# A Prognostic Risk Score Based on Hypoxia-, Immunity-, and Epithelialto-Mesenchymal Transition-Related Genes for the Prognosis and Immunotherapy Response of Lung Adenocarcinoma

**DOI:** 10.3389/fcell.2021.758777

**Published:** 2022-01-24

**Authors:** Wenhao Ouyang, Yupeng Jiang, Shiyi Bu, Tiantian Tang, Linjie Huang, Ming Chen, Yujie Tan, Qiyun Ou, Luhui Mao, Yingjie Mai, Herui Yao, Yunfang Yu, Xiaoling Lin

**Affiliations:** ^1^ Guangdong Provincial Key Laboratory of Malignant Tumor Epigenetics and Gene Regulation, Department of Medical Oncology, Breast Tumor Centre, Phase I Clinical Trial Centre, Sun Yat-Sen Memorial Hospital, Sun Yat-Sen University, Guangzhou, China; ^2^ Department of Pulmonary and Critical Care Medicine, Sun Yat-Sen Memorial Hospital, Sun Yat-Sen University, Guangzhou, China; ^3^ Department of Ultrasound in Medicine, Sun Yat-Sen Memorial Hospital, Sun Yat-Sen University, Guangzhou, China; ^4^ Artificial Intelligence and Digital Media Programme, Division of Science and Technology, Beijing Normal University-Hong Kong Baptist University United International College, Hong Kong Baptist University, Zhuhai, China

**Keywords:** lung adenocarcinoma, hypoxia, immune, EMT, gene signature, immunotherapy response

## Abstract

**Background:** Lung adenocarcinoma (LUAD), the most common subtype of non-small cell lung cancer (NSCLC), is associated with poor prognosis. However, current stage-based clinical methods are insufficient for survival prediction and decision-making. This study aimed to establish a novel model for evaluating the risk of LUAD based on hypoxia, immunity, and epithelial-mesenchymal transition (EMT) gene signatures.

**Methods:** In this study, we used data from TCGA-LUAD for the training cohort and GSE68465 and GSE72094 for the validation cohorts. Immunotherapy datasets GSE135222, GSE126044, and IMvigor210 were obtained from a previous study. Using bioinformatic and machine algorithms, we established a risk model based on hypoxia, immune, and EMT gene signatures, which was then used to divide patients into the high and low risk groups. We analyzed differences in enriched pathways between the two groups, following which we investigated whether the risk score was correlated with stemness scores, genes related to m^6^A, m^5^C, m^1^A and m^7^G modification, the immune microenvironment, immunotherapy response, and multiple anti-cancer drug sensitivity.

**Results:** Overall survival differed significantly between the high-risk and low-risk groups (HR = 4.26). The AUCs for predicting 1-, 3-, and 5-year survival were 0.763, 0.766, and 0.728, respectively. In the GSE68465 dataset, the HR was 2.03, while the AUCs for predicting 1-, 3-, and 5-year survival were 0.69, 0.651, and 0.618, respectively. The corresponding values in the GSE72094 dataset were an HR of 2.36 and AUCs of 0.653, 0.662, and 0.749, respectively. The risk score model could independently predict OS in patients with LUAD, and highly correlated with stemness scores and numerous m^6^A, m^5^C, m^1^A and m^7^G modification-related genes. Furthermore, the risk model was significantly correlated with multiple immune microenvironment characteristics. In the GSE135222 dataset, the HR was 4.26 and the AUC was 0.702. Evaluation of the GSE126044 and IMvigor210 cohorts indicated that PD-1/PD-LI inhibitor treatment may be indicated in patients with low risk scores, while anti-cancer therapy with various drugs may be indicated in patients with high risk scores.

**Conclusion:** Our novel risk model developed based on hypoxia, immune, and EMT gene signatures can aid in predicting clinical prognosis and guiding treatment in patients with LUAD.

## 1 Introduction

Lung adenocarcinoma (LUAD) is the most common subtype of non-small cell lung cancer (NSCLC) (Hyuna et al., 2021). Despite advances in standard treatment strategies based on clinical stage, the survival rate remains poor among patients with LUAD (Bi et al., 2020; Siegel et al., 2021), and the associated tumors are highly heterogeneous. Thus, developing a method for accurately stratifying risk and guiding treatment is essential.

Hypoxic conditions in the tumor microenvironment (TME) and immune microenvironment play a crucial role and are regarded as the major drivers of malignancy in LUAD. Further, both environments are strongly associated with malignant progression, therapeutic resistance, and poor prognosis (Wang D. D. et al., 2021; Wu et al., 2021; Zhang Y. et al., 2021). Several studies have recently shown that a hypoxic stimulus can alter the TME, decreasing the proportion of immune cells and increasing the expression of immunosuppressive cytokines (Zeng et al., 2021). Thus, hypoxia is considered the major immunosuppressive mechanism during cancer development (Labiano et al., 2015). Moreover, hypoxic stimulation can activate epithelial-mesenchymal transition (EMT), a key link in cancer progression (Jiang et al., 2011). Despite these findings, reliable prognostic signatures based on the fundamental combination of hypoxia, immunity, and EMT gene signatures have yet to be established.

Hence, to aid in improving clinical management strategies, the present study aimed to establish a novel model for evaluating LUAD risk based on genes related to hypoxia, immunity, and EMT.

## 2 Materials and Methods

### 2.1 Data Acquisition

Gene expression data, clinical survival information, and gene mutation information for patients with LUAD were downloaded from The Cancer Genome Atlas (TCGA) database (TCGA-LUAD) (Schabath et al., 2016) and the Gene Expression Omnibus (GEO) database (GSE68465, GSE72094) (Zhang A. et al., 2021). The TCGA-LUAD data were used for the training cohort, while those for GSE68465 and GSE72094 were used for the validation cohorts. The TCGA-LUAD dataset was delivered via an Illumina HiSeq 2000 microarray, the GSE68465 dataset was delivered via the Affymetrix Human Genome U133A Array, and the GSE72094 dataset was delivered via the Rosetta/Merck Human RSTA Custom Affymetrix 2.0 microarray. The “sva” package of R software was used to correct the batch effect between different datasets using the “combat” algorithm.

Hypoxia- and EMT-related genes were extracted from the hallmark gene set in the Molecular Signatures Database v7.0(MSigDB, www.gsea-msigdb.org), which includes 200 hypoxia genes and 200 EMT-related genes; 2,498 immune-related genes were acquired in the ImmPort (http://www.immport.org/). This study was approved by the Ethics and Research Committees of Sun Yat-Sen Memorial Hospital and Sun Yat-Sen University.

### 2.2 Screening of Differentially Expressed Hypoxia-, Immunity-, and EMT-Related Genes

Information regarding the expression of 200 hypoxia-, 2,498 immune-, and 200 EMT-related genes was collected from the TCGA-LUAD database. Differentially expressed genes (DEGs) between LUAD and normal lung tissue were then identified using the Wilcoxon test according to |Log2FC| > 1 and *p* < 0.05 would considered as DEGs. Log2FC > 1 indicating upregulated genes and Log2FC < −1 indicating downregulated genes, respectively. Heat and volcano maps were then generated to show the expression of different genes.

### 2.3 Functional Exploration of DEGs

An R software package (clusterprofiler, version 3.12) was used to perform Gene Ontology (GO) and Kyoto Encyclopedia of Genes and Genomes (KEGG) pathway enrichment analysis. Using Fisher’s exact test, those with false discovery rate (FDR)-corrected *p* values less than 0.05 were regarded as significant indicators.

### 2.4 Construction and Verification of the Risk Model

First, RNA expression in the TCGA-LUAD, GSE68465, and GSE72094 datasets was cross-checked to identify co-expressed and differentially expressed hypoxia-, immunity-, and EMT-related genes. Consequently, univariate Cox analysis of overall survival (OS) was performed to screen for hypoxia-, immunity-, and EMT-related genes with prognostic values. Next, least absolute shrinkage and selection operator (LASSO) regression with 10-fold cross-validation was performed, and 1,000 cycles were run via the R software package “glmnet.” For each cycle, 1,000 random simulations were performed. Based on the optimal lambda value, the best possible gene was selected to construct the model, and a risk formula was established.

The risk scores were calculated according to the expression of each gene and its corresponding regression coefficients using the following equation: risk score = ∑genes Cox coefficient × gene expression. The patients were then categorized into high-risk and low-risk groups based on the optimal cutoff value, which was computed using the “surv_cutpoint” function in the “survminer” R package. Receiver operating characteristic curves were drawn via the R Package “survivalROC” to estimate the predictive sensitivity of the formula. Model effectiveness was evaluated in the validation set using the same coefficients and cutoff values used in the training set. We then evaluated whether the risk score formula exhibited independent prognostic value when combined with clinical variables via multiple regression analysis.

### 2.5 Selection of m^6^A, m^5^C, m^1^A and m^7^G Genes

The expression matrices of m6A genes were including (METTL14, METTL3, RBM15, RBM15B, WTAP, CBLL1, ZC3H13, ALKBH5, FTO, YTHDC1, YTHDF1, YTHDC2, YTHDF2, IGF2BP1, YTHDF3, FMR1, HNRNPC, HNRNPA2B1, ELAVL1, and LRPPRC). The expression of m5C genes including (NSUN7, ALYREF, NSUN1, NSUN6, NSUN3, NSUN4, NSUN2 and NSUN5); The expression of m1A genes including (ALKBH3, ALKBH1 and YTHDF2); The expression of m7G genes including (METTL1, BUD23 and RNMT).

### 2.6 Differential Analysis of Immune Cell Infiltration, Immune Function, and Immune Checkpoint Function and the Validation of Immunotherapeutic Responses

Immune cell infiltration was identified using timer 2.0 (cistrome.shinyapps.io/timer/) *via* the Timer, QUANTISEQ, CIBERSORT, CIBERSORT-ABS, XCELL, MCPCOUNTER, and EPIC algorithms. The “gsva” R package was used to process the single-sample gene set of the enrichment analysis (ssGSEA) to calculate the activity status of 13 immune-related pathways. The selection of immune-checkpoint genes was based on the findings of a previous study (Isomura et al., 2021). The ESTIMATE algorithm was used to calculate the stromal score, immune score, and ESTIMATE score of TCGA-LUAD samples.

Given the lack of information on immune therapy in the TCGA-LUAD cohort, the predictive capability of the risk score formula was evaluated using the GSE135222 (NSCLC), GSE126044 (NSCLC), and IMvigor210 (metastatic urothelial cancer) cohorts (Charoentong et al., 2017; Mariathasan et al., 2018; Jung et al., 2019; Yu et al., 2019; Yu et al., 2020a; Cho et al., 2020).

### 2.7 Predicting Anti-Cancer Drug Response

To evaluate the ability of the risk score to predict the chemotherapeutic response, the half-maximal inhibitory concentration (IC50) of common chemotherapeutic drugs was first calculated in the TCGA-LUAD training group, using the “pRRophetic” package in R software. The Wilcoxon rank test was then used to compare the difference in IC50 between the low- and high-risk groups. Finally, the R package “ggplot” was used to visualize the data.

### 2.8 Statistical Analysis

DEGs were screened using the Wilcoxon test. Univariate Cox analysis of overall survival (OS) was performed to screen relevant genes with prognostic values. Kaplan–Meier survival curves were generated and compared between the two groups using the log-rank test. The associations between the risk score determined using the prognostic model and the stromal score, stemness score, and immune score were assessed using Spearman correlation analysis. All statistical analyses were performed using R version 4.0.0 (R-project.org) and its adequate packages. Statistical significance was set at *p* < 0.05.

## 3 Results

**FIGURE 1:**
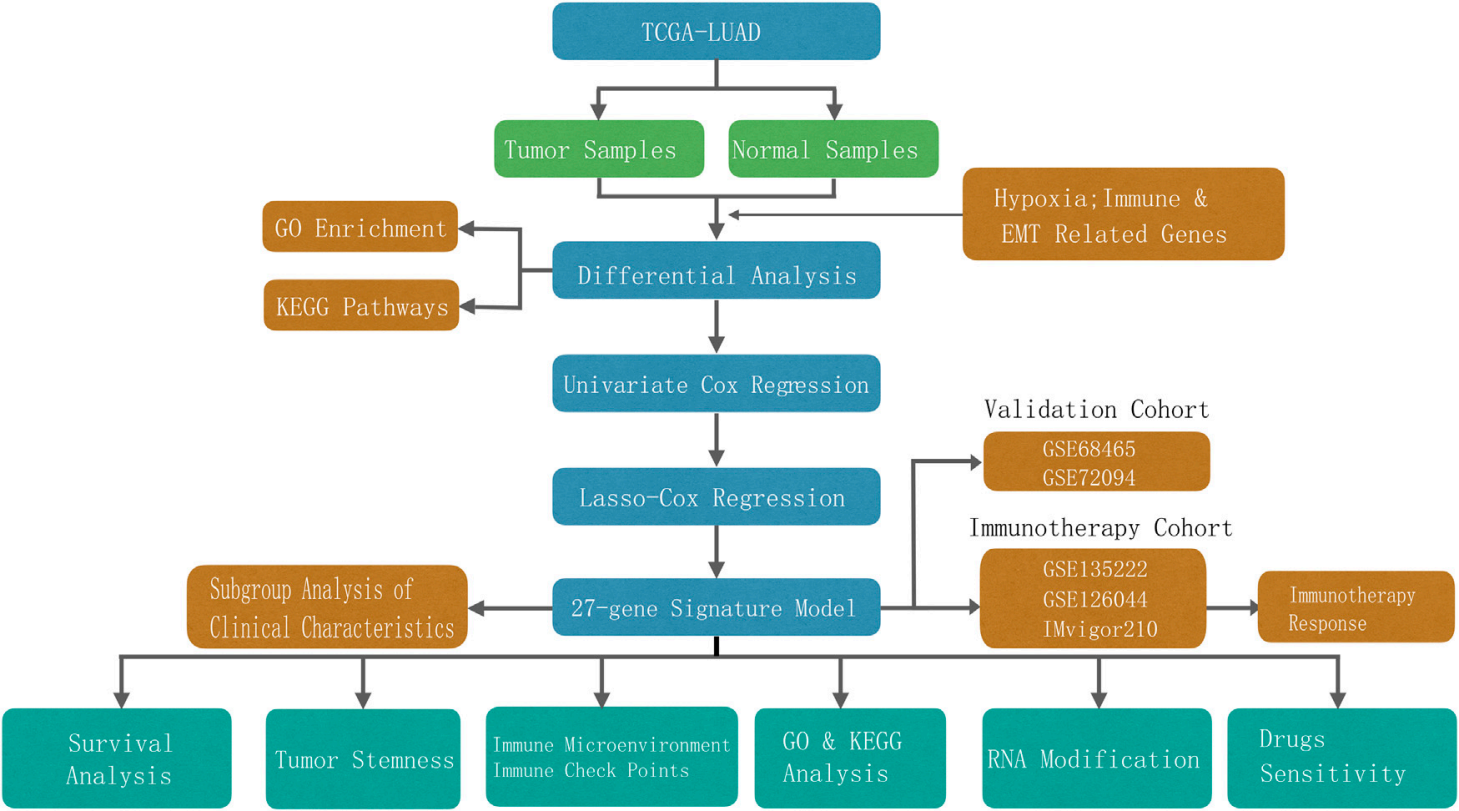
Flow chart of this study.

Totals of 500 and 840 patients with LUAD were selected from the training and validation sets, respectively. The detailed clinical features of these patients are summarized in Supplementary Table S1. The flowchart of the study is shown in Figure 1.

### 3.1 Differentially Expressed Hypoxia-, Immune-, and EMT-Related Genes

**FIGURE 2:**
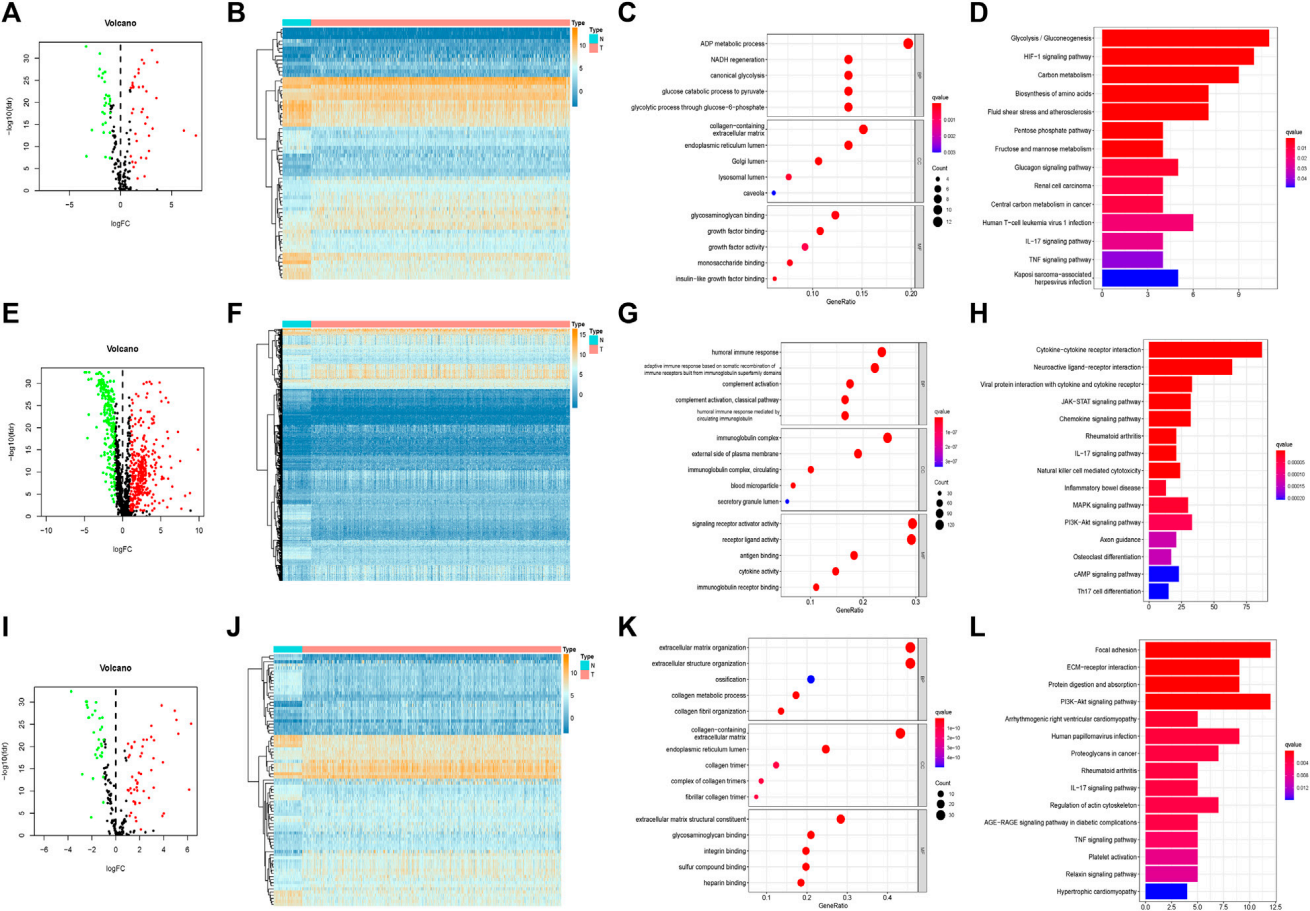
Separativelly screening of differentially expressed hypoxia, immunity and EMT related genes. **(A)** Volcano plots showing the hypoxia-related DEGs. **(B)** Heatmaps of differentially expressed hypoxia-related mRNAs. **(C)** GO enrichment of hypoxia-related DEGs. **(D)** KEGG pathways of hypoxia-related DEGs. **(E)** Volcano plots showing the immune-related DEGs. **(F)** Heatmaps of differentially expressed immune-related mRNAs. **(G)** GO enrichment of immune-related DEGs. **(H)** KEGG pathways of immune-related DEGs. **(I)** Volcano plots showing the EMT-related DEGs. **(J)** Heatmaps of differentially expressed EMT-related mRNAs. **(K)** GO enrichment of EMT-related DEGs. **(L)** KEGG pathways of EMT-related DEGs.

**FIGURE 3:**
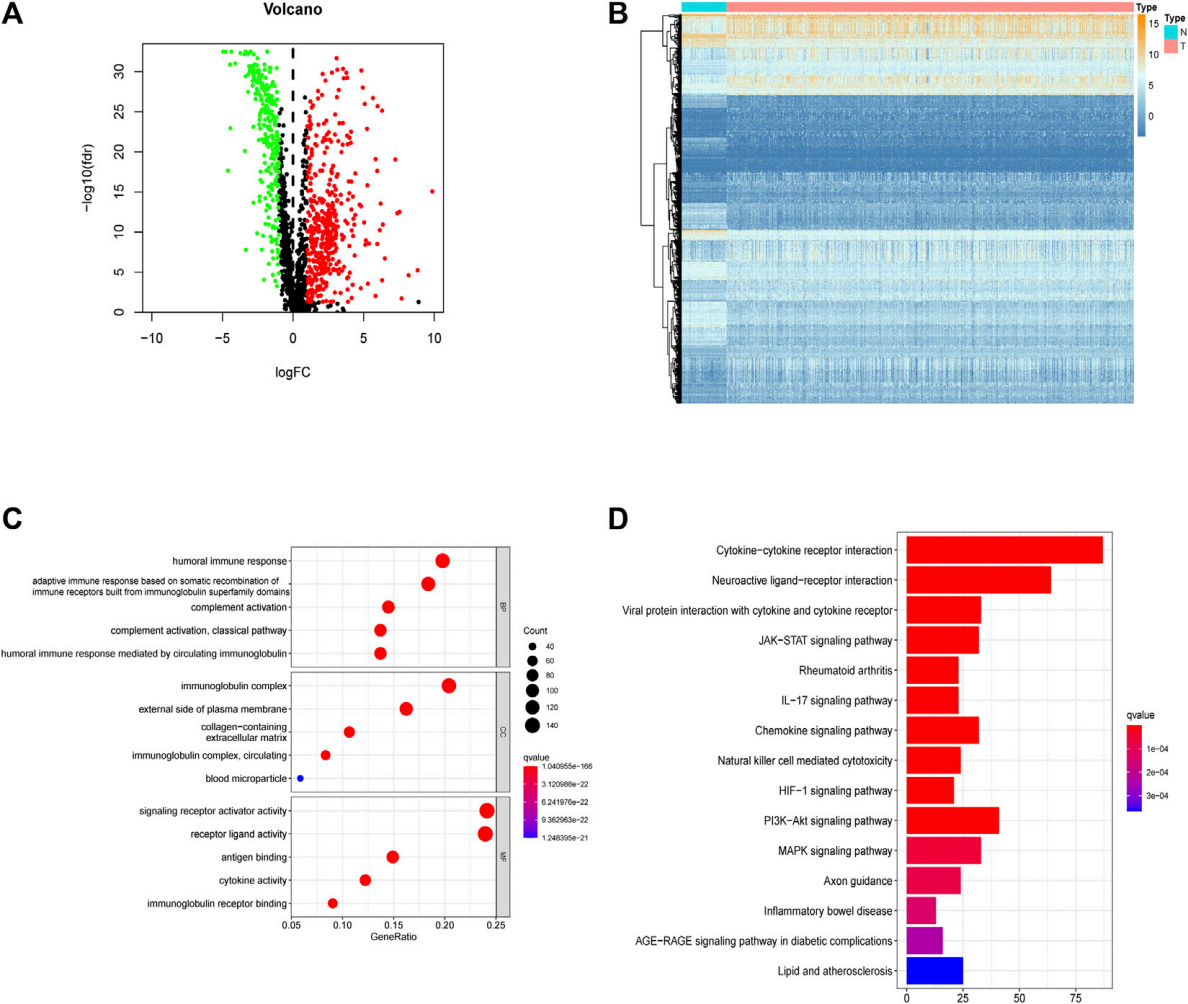
Integrally screening of differentially expressed hypoxia, immunity and EMT related genes. **(A)** Volcano plots of the integrated DEGs. **(B)** Heatmaps of the integrated mRNAs. **(C)** GO enrichment based of integrated DEGs. **(D)** KEGG pathways of integrated DEGs.

In the training set, 66 of 169 hypoxia-related genes, 556 of 1,214 immune-related genes, and 81 of 177 EMT-related genes were differentially expressed between LUAD and adjacent normal tissues. Of these, 37 hypoxia-related genes, 345 immune-related genes, and 50 EMT-related genes were upregulated, while 29 hypoxia-related genes, 211 immune-related genes, and 31 EMT-related genes were downregulated (Figures 2A,B,E,F,I,J). In total, there were 703 of 1,560 DEGS, 432 and 271 of which were upregulated and downregulated, respectively (Figures 3A,B).

### 3.2 Functional Analysis of Hypoxia-, Immune-, and EMT-Related DEGs

In the GO enrichment analysis, we identified the top 5 GO categories with significant enrichment of genes related to hypoxia, immunity, or EMT. The most significantly altered hypoxia-, immune-, and EMT-related genes were involved in the metabolic processing of ADP, in signaling receptor activator activity, and in extracellular matrix organization, respectively (Figures 2C,G,K; Supplementary Tables S2, S4, S6). We then performed KEGG analysis and identified the top 15 KEGG categories with significant enrichment of hypoxia-, immune-, and EMT-related genes. The altered hypoxia-related genes were mostly associated with glycolysis, while the altered immune-related genes were mostly associated with cytokine–cytokine receptor reactions. The EMT-related genes exhibiting the most significant alterations were involved in focal adhesion (Figures 2D,H,L) (detailed in Supplementary Tables S3, S5, S7). Further, when these gene signatures were combined, the most correlated GO and KEGG categories were signaling receptor activator activity cytokine–cytokine receptor reactions, respectively (Figures 3C,D) (Supplementary Tables S8, S9).

### 3.3 Predictive Ability of the Risk Score

**FIGURE 4:**
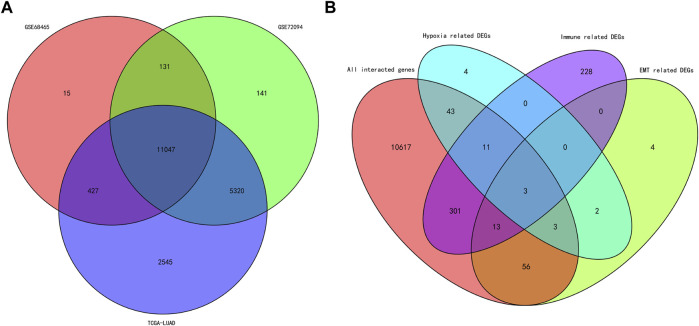
Venn diagram of the intersected genes and DEGs. **(A)** Venn diagram of the intersected genes among the cohorts of TCGA, GSE68465 and GSE72094. **(B)** Venn diagram of the intersected hypoxia, immune and EMT related DEGs.

**FIGURE 5:**
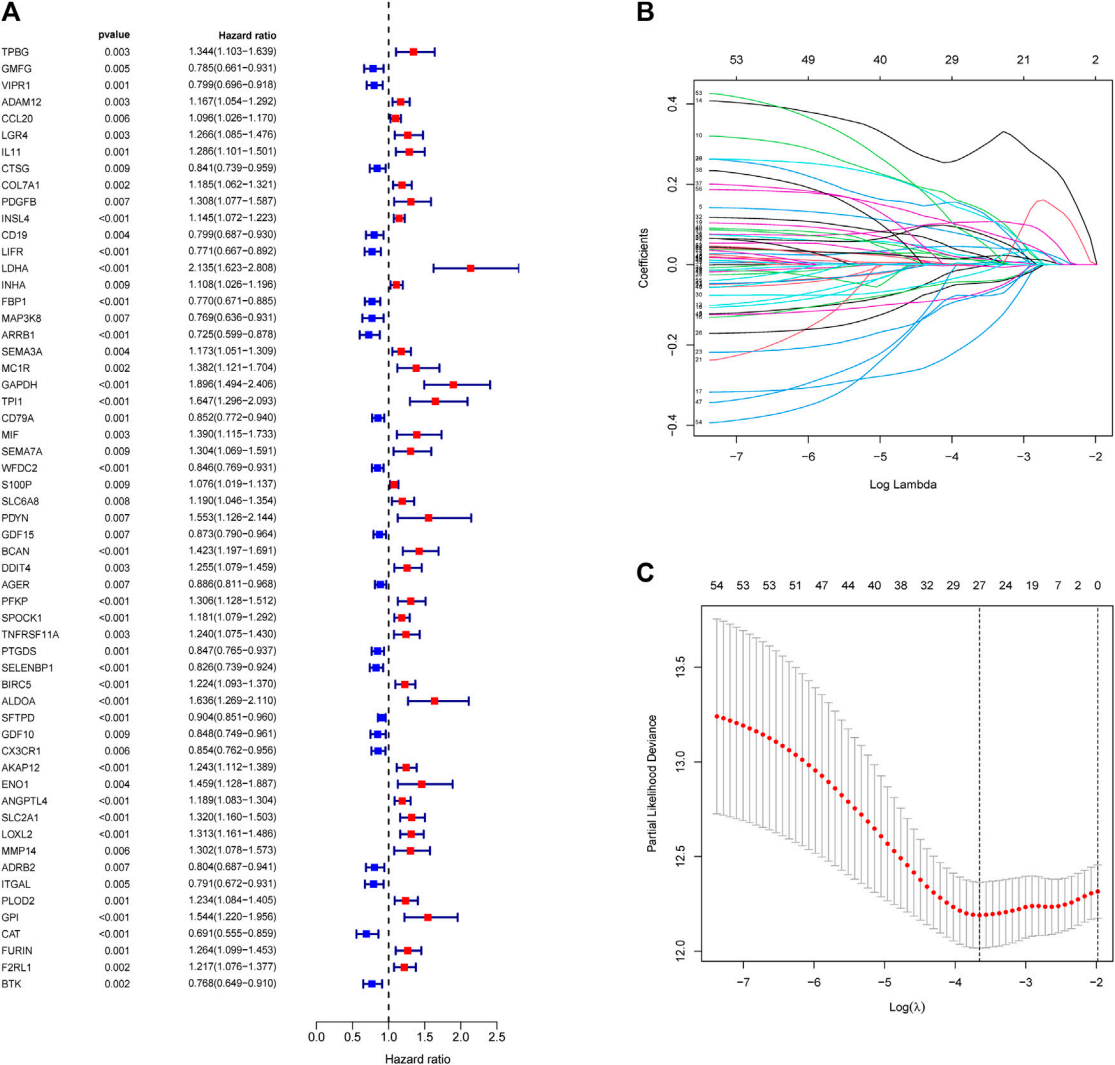
Construction of risk score formula. **(A)** Forest plots showing the results of survival related gene via univariate Cox regression analysis between interacted genes and OS. **(B)** LASSO coefficient profile plots of the 57 prognostic related genes showing that the variations in the size of the coefficients of parameters shrink with an increasing value of the k penalty. **(C)** Penalty plot for the LASSO model for the 57 prognostic genes with error bars denoting the standard errors.

**FIGURE 6:**
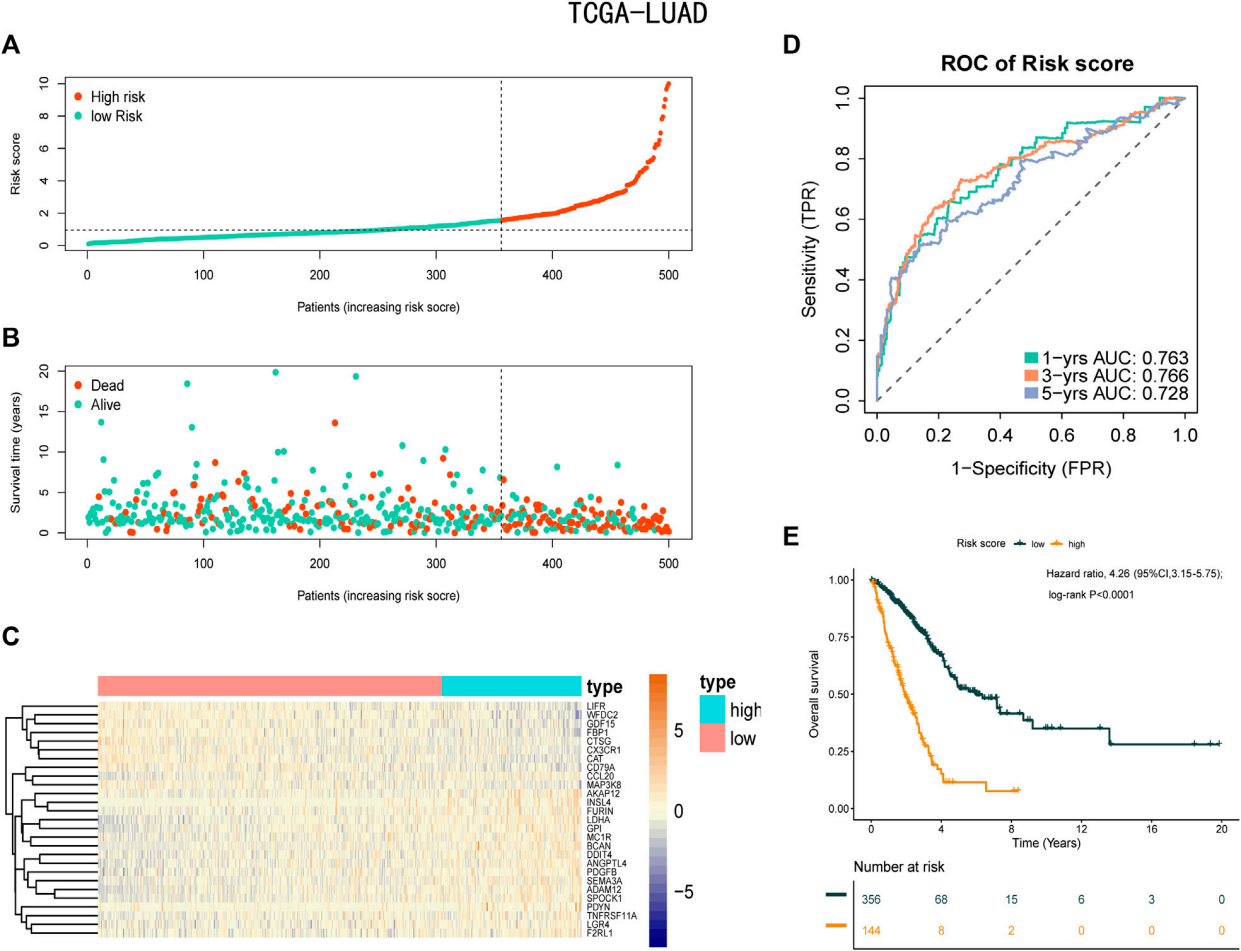
Prognostic analysis of the risk score formula in the training set. **(A)** Distribution of risk score for the training set. **(B)** Patterns of the survival time and survival status between the high-risk and low-risk groups for training set. **(C)** Heatmaps of the 27 prognostic genes for each patient in training set. **(D)** Time-related ROC analysis proved the prognostic performance of the risk score in the training set. **(E)** Kaplan-Meier survival curve of the patients in the high-risk and low-risk groups for OS in the training set.

A total of 11,074 genes were co-expressed; among them, 430 of 668 hypoxia-, immune-, and EMT-related DEGs were selected (Figures 4A,B). Then, these 430 genes were used in the univariate Cox regression analysis. A total of 57 prognostic genes were identified (Figure 5A). To avoid overfitting the prognostic model, LASSO regression analysis was performed. Finally, 27 genes were selected and included in the risk score formula, as follows: Risk score = ADAM12 × 0.0537 + CCL20 × 0.1149 + LGR4 × 0.0481 − CTSG × 0.0435 + PDGFB × 0.2173 + INSL4×0.0526 + LIFR×0.0033 + LDHA × 0.1794 − FBP1 × 0.0417 − MAP3K8×0.3235 + SEMA3A × 0.0329 + MC1R × 0.1367 − CD79A × 0.1300 − WFDC2 × 0.0577 + PDYN × 0.2017 − GDF15 × 0.0710 + BCAN × 0.1043 + DDIT4 × 0.0715 − SPOCK1 × 0.0148 + TNFRSF11A × 0.1982 − CX3CR1×0.1238 − AKAP12 × 0.0061 + ANGPTL4 × 0.0227 + GPI × 0.1924 − CAT × 0.0789 + FURIN × 0.0187 + F2RL1 × 0.1408 (Figures 5B,C). Based on am optimistic cut off, 144 and 356 patients were categorized into the high-risk and low-risk groups, respectively (Figures 6A–C). Kaplan–Meier survival analysis revealed that OS was lower in the high-risk group than in the low-risk group (Figure 6E). The area under the curve (AUC) values for predicting 1-, 3-, and 5-year OS were 0.763, 0.766, and 0.728, respectively (HR = 4.26; 95% CI 3.15–5.75; *p* < 0.0001; Figure 6D). These results show that the risk model based on the 27 genes listed above had high accuracy in predicting the OS of patients with LUAD. Besides, we also proved the novel risk score independently predict the OS of LUAD (Supplementary Figure S1).

### 3.4 Stability of the Risk Score Formula Constructed Using Hypoxia-Related Genes

**FIGURE 7:**
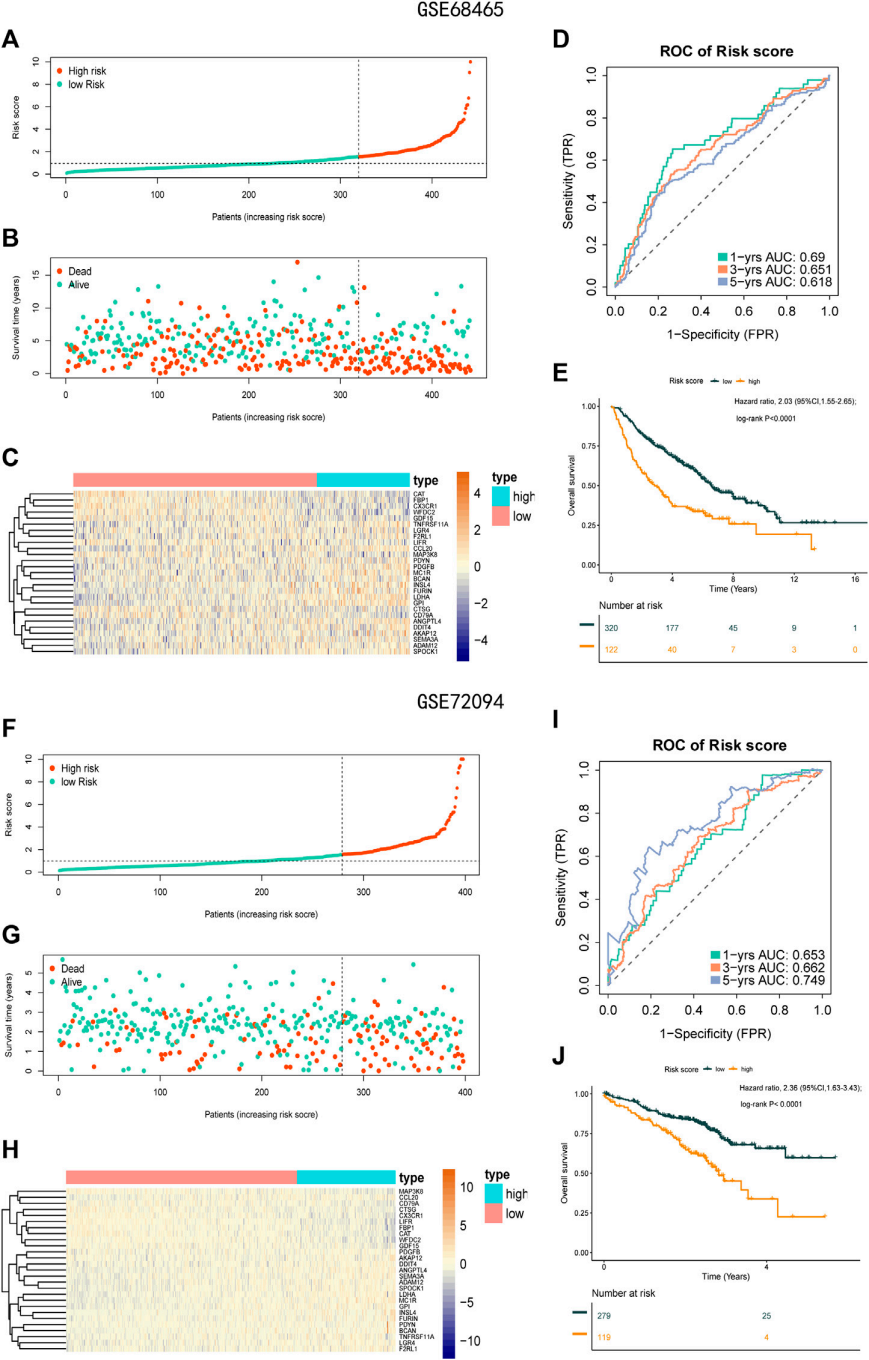
Prognostic analysis of the 27-gene signature model in the validation sets GSE68465 and GSE72094. **(A)** Distribution of risk score for the GSE68465. **(B)** Patterns of the survival time and survival status between the high-risk and low-risk groups for GSE68465. **(C)** Heatmaps of the 27 prognostic genes for each patient in GSE68465. **(D)** Time-related ROC analysis proved the prognostic performance of the risk score in the GSE68465. **(E)** Kaplan-Meier survival curve of the patients in the high risk score and low risk score groups for OS in the GSE68465. **(F)** Distribution of risk score for the GSE72094. **(G)** Patterns of the survival time and survival status between the high-risk and low-risk groups for GSE72094. **(H)** Heatmaps of the 27 prognostic genes for each patient in GSE72094. **(I)** Time-related ROC analysis proved the prognostic performance of the risk score in the GSE72094. **(J)** Kaplan-Meier survival curve of the patients in the high-risk and low-risk groups for OS in the GSE72094.

To check the stability of the model developed from the training set, patients in the validation sets (GSE68465 and GSE72094) were also divided into a high-risk group and a low-risk group according to the same cut-off value and risk formula as those in TCGA cohort (Figures 7A–C,F–H). The results indicated that OS was markedly lower in the high-risk group than in the low-risk group (Figures 7E,J). In the GSE68465 set, the AUCs for predicting 1-, 3-, and 5-year OS were 0.69, 0.651, and 0.618, respectively (HR = 2.03; 95% CI = 1.55–2.65; *p* < 0.0001). In the GSE72094 cohort, the corresponding AUCs were 0.653, 0.662, and 0.749, respectively (HR = 2.36; 95% CI = 1.63–3.43; *p* < 0.001; Figures 7D,I).

### 3.5 Subgroup Analysis Using the Risk Score Formula

**FIGURE 8:**
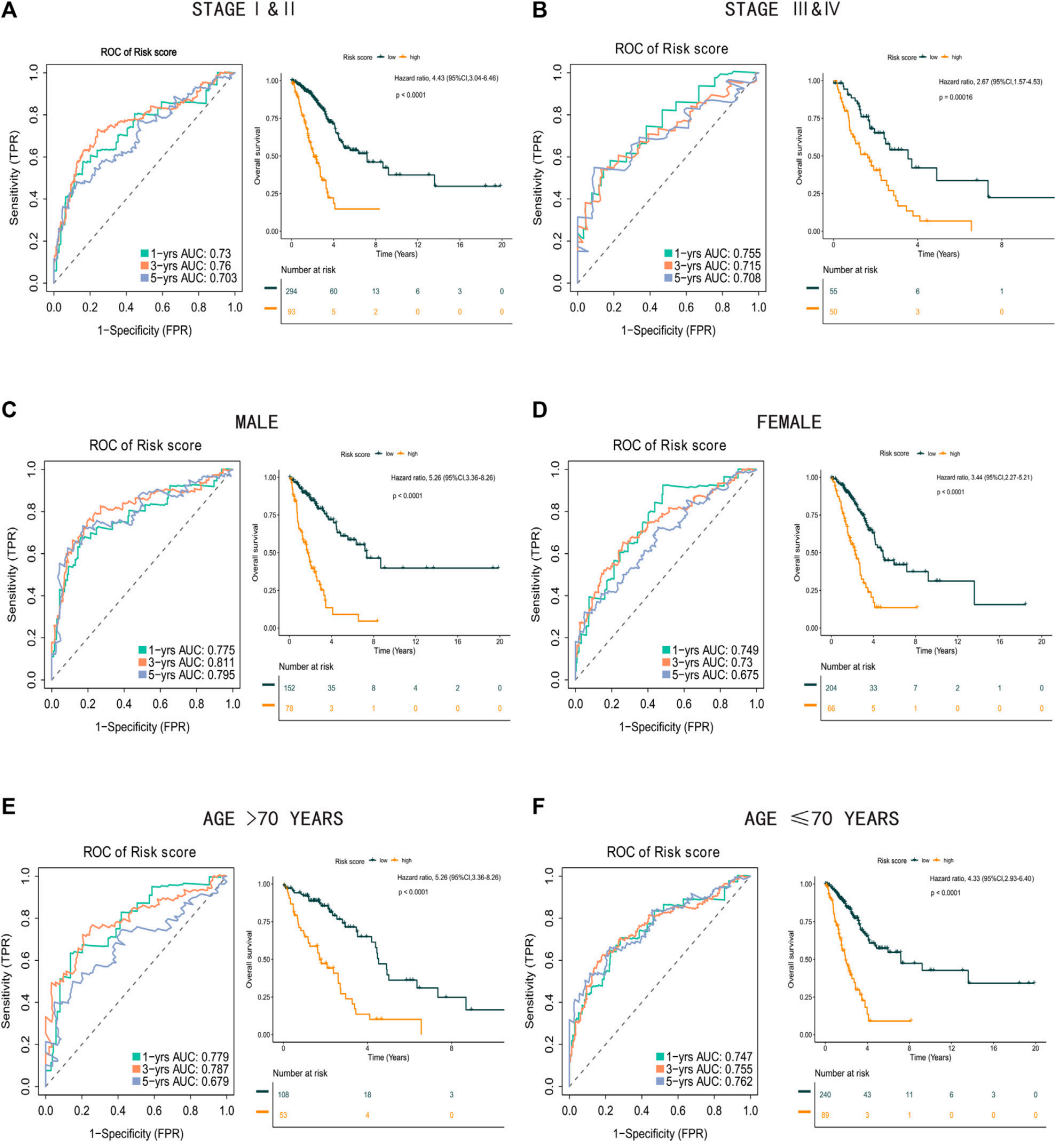
Clinical subgroups analysis between high risk score group and low risk score group. **(A)** Time-related ROC analysis and Kaplan-Meier survival curve of the patients in stage I and stage II. **(B)** Time-related ROC analysis and Kaplan-Meier survival curve of the patients in stage III and stage IV. **(C)** Time-related ROC analysis and Kaplan-Meier survival curve of the male patients. **(D)** Time-related ROC analysis and Kaplan-Meier survival curve of the female patients. **(E)** Time-related ROC analysis and Kaplan-Meier survival curve of the patients more than 70 years old. **(F)** Time-related ROC analysis and Kaplan-Meier survival curve of the patients less than or equal to 70 years old.

Next, we analyzed the association between clinical features (including stage, age, and sex) and the risk score in the TCGA-LUAD database. The risk score remained significantly effective across all subgroups based on tumor stage, sex, and age (Figure 8), supporting the reliability of the risk score formula. Moreover, in the univariate and multivariate Cox regression analysis, the risk score formula was identified as an independent prognostic indicator of poor outcomes in patients with LUAD (Supplementary Figures S1A,B).

### 3.6 Functional Analysis

**FIGURE 9:**
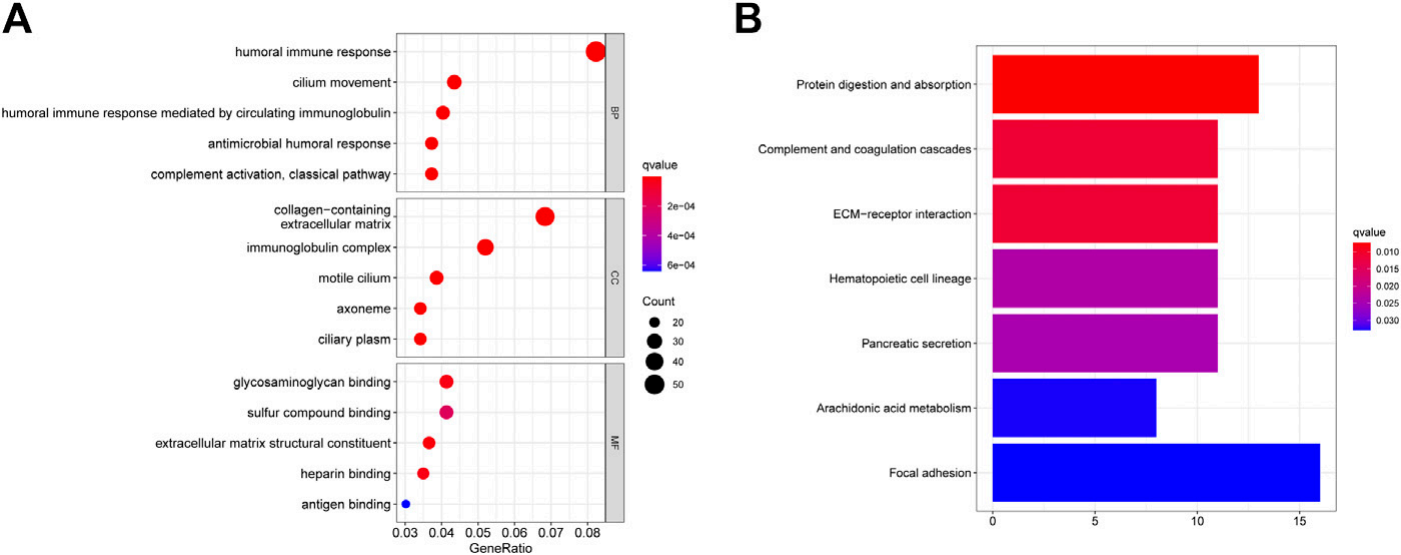
GO and KEGG enrichment analysis between high risk score group and low risk score group. **(A)** GO enrichment between the high-risk patients and low-risk patients in TCGA cohort. **(B)** KEGG pathways between the high-risk patients and low-risk patients in TCGA cohort.

Further analysis of the differences in enrichment pathways between the low-risk and high-risk groups showed that the most different pathways were related to the humoral immune response, collagen-containing extracellular matrix, and focal adhesion (Figures 9A,B; Supplementary Tables S10, S11). This may explain why OS was lower in the high-risk group than in the low-risk group.

### 3.7 Tumor Stemness Analysis and Gene Mutation Landscape

**FIGURE 10:**
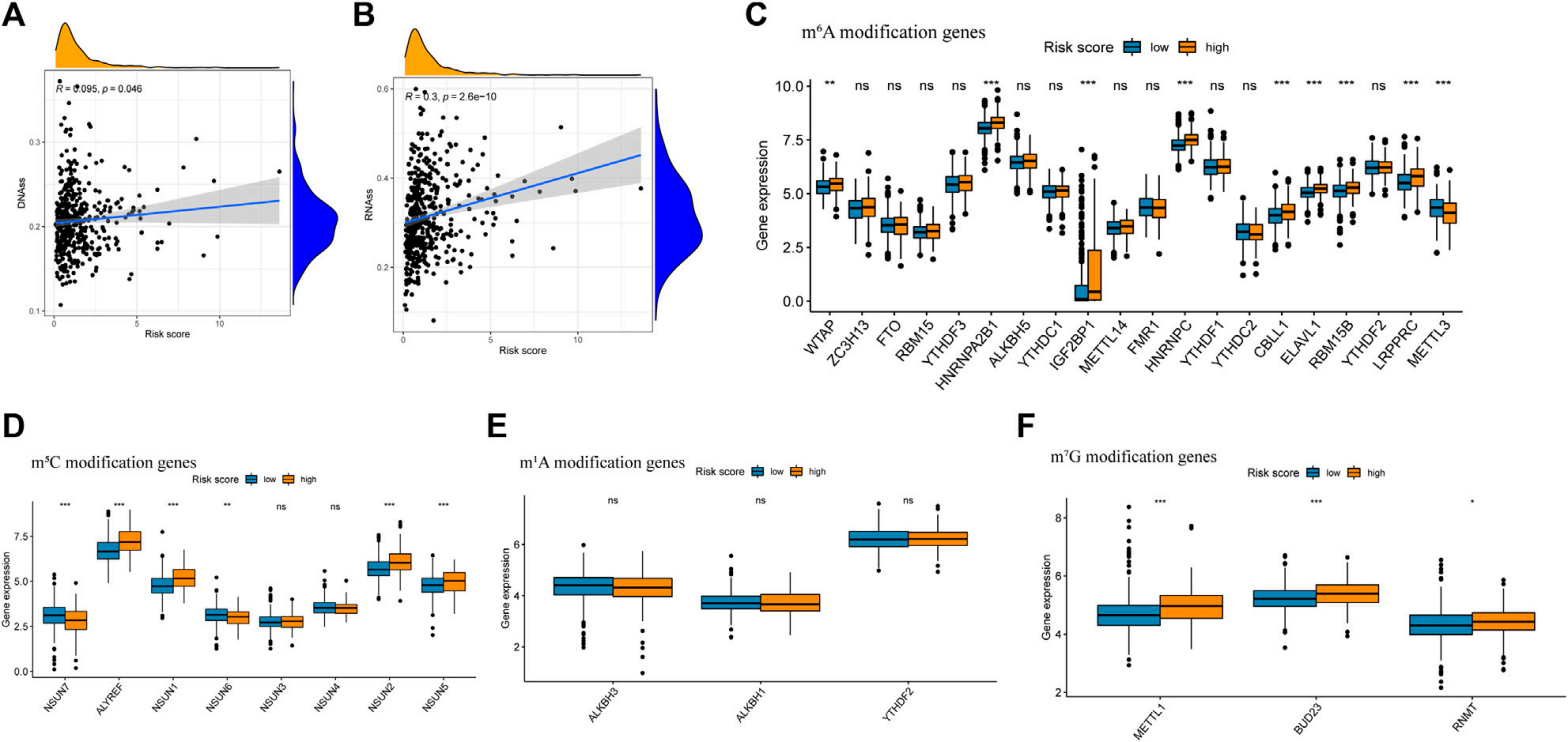
Analysis of risk score with tumor stem cell score and Differential expression of m^6^A related gene between high risk score group and low risk score group. **(A)** The relationship between risk score and RNAss. **(B)** The relationship between risk score and DNAss. **(C)** Differential expression analysis of m^6^A related gene between high risk score group and low risk score group. **(D)** Differential expression analysis of m^5^C related gene between high risk score group and low risk score group. **(E)** Differential expression analysis of m^1^A related gene between high risk score group and low risk score group. **(F)** Differential expression analysis of m^7^G related gene between high risk score group and low risk score group.

Growing evidence indicates that increased expression of stemness-related biomarkers in tumor cells is highly correlated with drug resistance, cancer recurrence, and tumor proliferation (Luo and Vögeli, 2020). Hence, we assessed the correlations of the DNA stemness score (DNAss) and RNA stemness score (RNAss) with the risk score. The results indicate that the risk score was significantly positively correlated with the DNAss and RNAss (Figures 10A,B). Besides, this study also compared the gene mutation landscape between high and low risk score group. We found in high risk score group, the mutation frequency of TP53, TTN and KEAP were obviously higher than low risk score group (Supplementary Figure S2).

### 3.8 Expression of m^6^A, m^5^C, m^1^A and m^7^G Modification-Related Genes

Previous research has indicated that m^6^A, m^5^C, m^1^A and m^7^G modification, which were reversible epigenetic RNA process, significantly involved in the proliferation and migration of cancer cells (Dib et al., 2017; Barbieri and Kouzarides, 2020). In this study, the expression of m^6^A modification genes WTAP, HNRNPA2B1, IGF2BP2, HNRNPC, CBLL1, ELAVL1, RBM15B, LRPPRC, and ELAVL1, the expression of m^5^C modification gene ALYREF, NSUN1 and NSUN2 and the expression of m7G modification gene METTL1, BUD23 and RNMT were significantly higher in the high risk group, while the expression of m^6^A modification gene METTL3, the expression of m^5^C modification gene NSUN7 and NSUN6 were significantly higher in the low-risk group (Figures 10C–F).

### 3.9 Analysis of Immune Status

**FIGURE 11:**
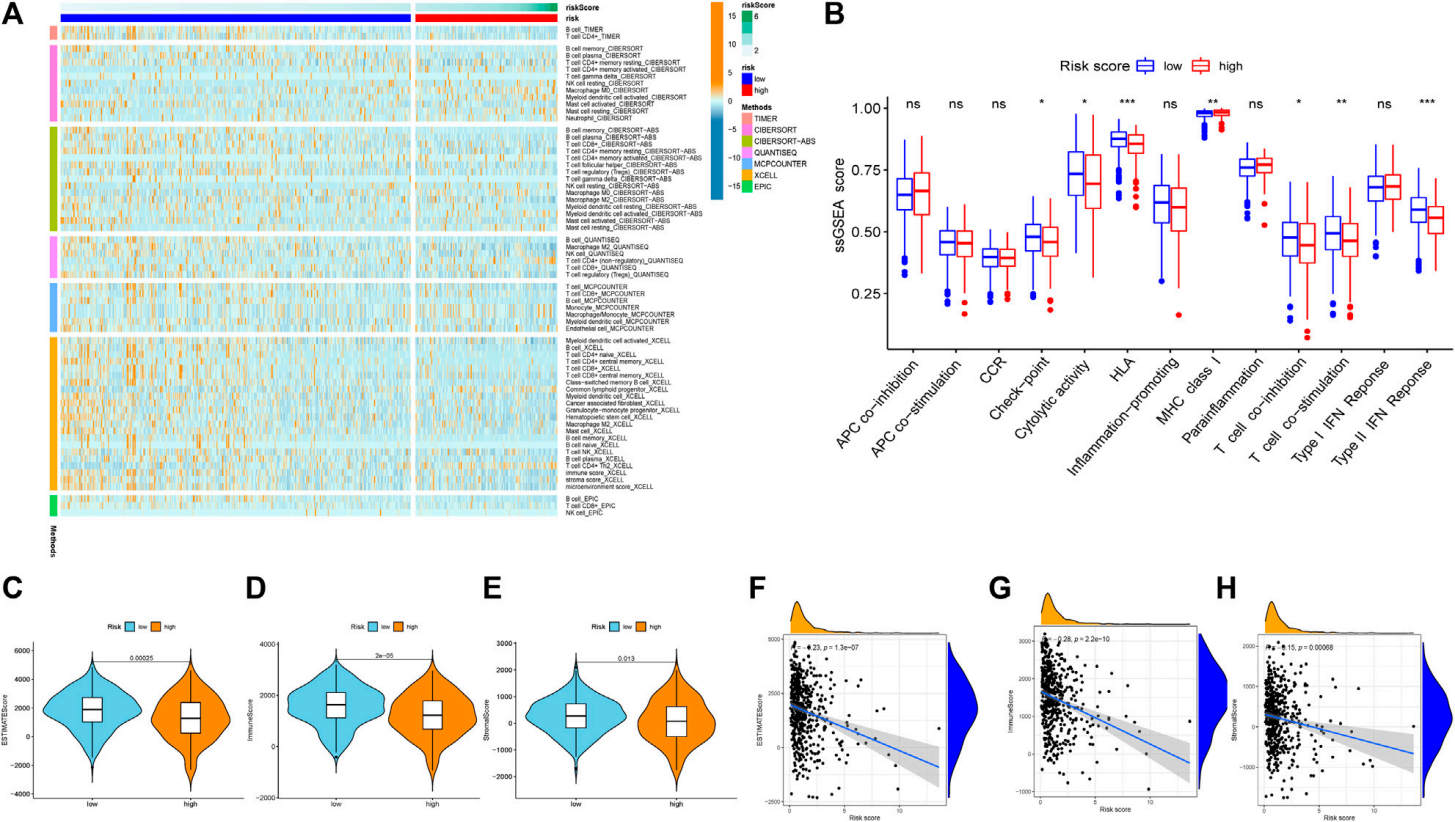
Exploration of tumor immune microenvironment between high risk score group and low risk score group. **(A)** Heatmap for immune responses based on CIBERSORT, TIMER, CIBERSORT-ABS, QUANTISEQ, MCPCOUNTER, XCELL, EPIC algorithms among high risk score group and low risk score group. **(B)** ssGSEA for the association between immune cell subpopulations and related functions. **(C)** ESTIMATE score. **(D)** Immune score. **(E)** Stromal score. **(F)** The relationship between risk score and the ESTIMATE score. **(G)** The relationship between risk score and the Immune score. (H)The relationship between risk score and the Stromal score. *p < 0.05; **p < 0.01; ***p < 0.001, ns, not significant.

The relationship between the risk score and the immune status of the patients in the TCGA cohort is shown in Figures 11A,B. There were significant alterations in immune checkpoint genes. Thus, we further compared the expression of immune checkpoint-related genes between the high-risk group and the low-risk group. The tumor immune microenvironment was also assessed using the immune score, ESTIMATE score, and stromal score (Yoshida et al., 2021). All three scores were negatively correlated with the risk score (Figures 11C–H), indicating stronger tumor immune activity in low-risk patients than in high-risk patients.

### 3.10 Analysis of Anti-Cancer Treatment Sensitivity

**FIGURE 12:**
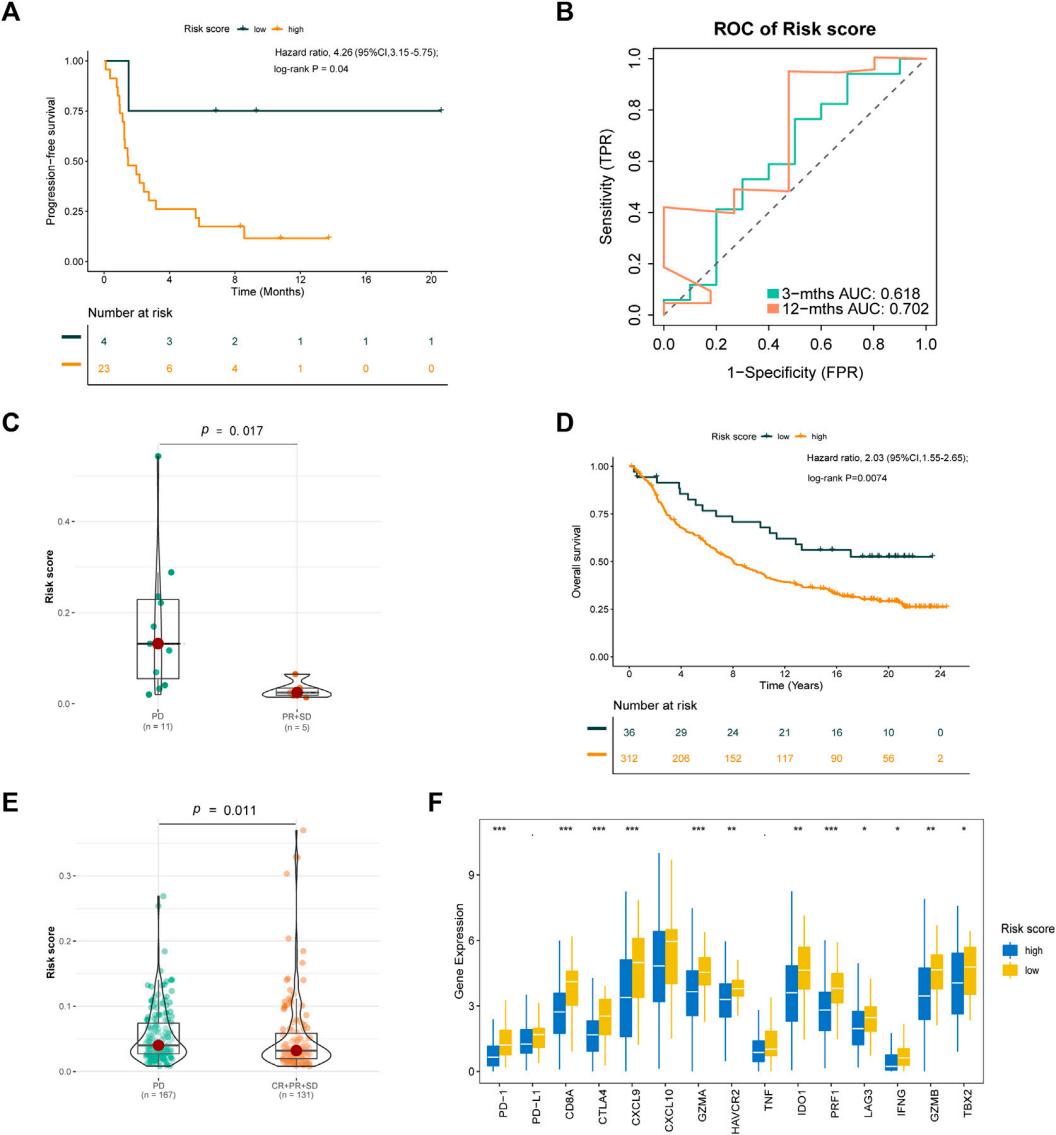
Validation of the risk score formula for immunotherapy. **(A)** Kaplan-Meier survival curve of GSE135222 cohort for PFS. **(B)** Time-related ROC analysis proved the prognostic performance of the risk score in the GSE135222. **(C)** The difference of risk score in the subgroup of PD-1 treatment response in GSE126044. **(D)** Kaplan-Meier survival curve of IMvigor210 cohort for OS. **(E)** The difference of Risk score in the subgroup of PD-L1 treatment response. **(F)** The expression of immue-related checkpoints among high and low risk groups in IMvigor210 cohort. *p < 0.1; *p < 0.05; **p < 0.01; ***p < 0.001.

**FIGURE 13:**
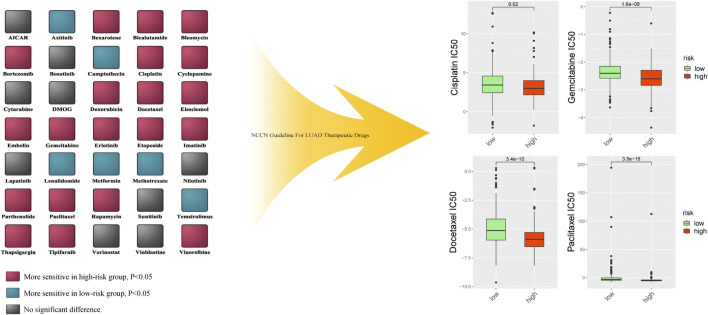
The difference of multiple anti-cancer drugs sensitivity between high risk score group and low risk score group.

To verify the prognostic value of the risk score formula for immunotherapy sensitivity, we selected three immunotherapy datasets from patients with NSCLC and metastatic urothelial cancer. The risk score formula was associated with progression-free survival (PFS) in patients with NSCLC undergoing anti-PD-1/PD-L1 therapy in the GSE135222 cohort (HR = 4.26; 95% CI = 3.15–5.75; *p* = 0.04) (Figure 12A), and the AUC value for predicting the 12-month PFS was 0.702 (Figure 12B). In the GSE126044 cohort, the risk score was higher in patients with NSCLC who had experienced no benefit (disease progression [PD]) from nivolumab or pembrolizumab than in those who had experienced a benefit (partial response [PR] + stable disease [SD]) (*p* = 0.017) (Figure 12C). Furthermore, the risk score was associated with worse immunotherapy response in patients with metastatic urothelial cancer (Figures 12D,F). The risk score was also significantly correlated with several immune checkpoint-related genes: *PD-1, CD8A, CTLA4, CXCL9, GZMA, HAVCR2, IDO1, PRF1, LAG3, IFNG, GZMB*, and *TBX2* (Figure 13G). Our analysis further revealed that a high risk score was associated with high sensitivity to common NCCN (National Comprehensive Cancer Network, https://www.nccn.org) recommended anti-LUAD drugs such as cisplatin, docetaxel, paclitaxel and gemcitabine (Figure 13). These results show that the risk score can be used as a potential predictor of chemosensitivity and that immunotherapy may be more appropriate for low-risk patients, while chemotherapy may be more appropriate for high-risk patients.

## 4 Discussion

The current era of precision medicine highlights an urgent need to establish a more precise method for evaluating prognosis and guiding treatment in patients with LUAD. Hypoxia, the immune microenvironment, and EMT play crucial roles in tumorigenesis, progression, and drug resistance in LUAD (Isomura et al., 2021; Wu et al., 2021; Yoshida et al., 2021).

Numerous studies have identified the existence of a hypoxic area as one of the key characteristics of cancer growth (Shen et al., 2015). Indeed, hypoxia promotes cancer metastasis and reduces the survival rate in patients with cancer (Nobre et al., 2018), and the expression of hypoxia genes has been shown to increase metabolism in lung adenocarcinoma cells (Smolle et al., 2020). At the same time, the hypoxic microenvironment of the tumor suppresses the ability of immune cells to detect and kill tumor cells (Vito et al., 2020). Additional studies have reported that the hypoxic microenvironment influences the effect of tumor chemotherapy and immune checkpoint inhibitors, which explain the increasing mortality rate among patients with LUAD (Ando et al., 2019; Daniel et al., 2019; Gao et al., 2019). Hypoxia can also contribute to EMT in patients with LUAD (Ando et al., 2019). Moreover, the immune system plays a vital role in the development and progression of malignant tumors (Lin et al., 2021). Immunotherapy is a novel treatment for LUAD that has achieved multiple satisfactory results (Li et al., 2020). EMT has also been shown to play a critical role in tumor development from initiation to metastasis (Taki et al., 2021). After EMT, LUAD cells can produce more extracellular matrix, which can hasten tumor metastasis, aggravate immune evasion, and induce drug resistance (Pastushenko and Cédric, 2019; Cui et al., 2020; Taki et al., 2021). Based on these findings, the present study combined hypoxia-, immunity-, and EMT-related gene signatures to construct a prognostic model for LUAD risk. To our best knowledge, this is the first study to combine these gene signatures to predict prognosis in patients with LUAD.

Using the LASSO Cox algorithm, 200 hypoxia-related genes, 2,498 immune-related genes, and 200 EMT-related genes were used to identify the most robust biomarkers and establish a novel risk score. In total, 27 related genes were included in the risk formula for LUAD prognosis. Using this formula, we classified patients with LUAD into the high- and low-risk groups. The formula had AUCs of 0.763, 0.766, and 0.728 for predicting 1-, 3-, and 5-year OS, respectively, indicating that it has high accuracy and reliability. Further, OS was significantly lower in the high-risk group than in the low-risk group, and the risk score exhibited high predictive capability in the GSE68465 and GSE72094 validation sets.

Subsequent subgroup analysis by sex, age, and stage indicated that the formula exhibited good predictive capability across all categories. Prognosis was accurately predicted in male and female patients and in patients aged >70 years or <70 years. Importantly, patients in the high-risk group also had significantly lower OS than patients in the low-risk group, regardless of disease stage, indicating the need for a gene-based classification for clinical use. Functional analysis between each group revealed strong associations between a high risk score and genes related to the humoral immune response, collagen-containing extracellular matrix, and focal adhesion. All of these are highly correlated with the anti-tumor response, tumor metastasis, drug resistance, and tumor progression (Murphy et al., 2012; Lovitt et al., 2018; Kosibaty et al., 2021).

Stemness-related biomarkers in tumor cells are highly correlated with drug resistance, cancer recurrence, and tumor proliferation (Liu et al., 2021). In this study, stemness-related biomarkers were positively associated with the risk score, demonstrating the prognostic value of the formula. Modification of m^6^A, m^5^C, m^1^A, and m^7^G are the common type of modification in RNA and plays critical roles in cancer development (Teng et al., 2021; Xu F. et al., 2021; Xu R. et al., 2021). And RNA methylation highly interconnected with hypoxia, immune response and EMT(Lin et al., 2019; Chao et al., 2020; Wang E. et al., 2021). In previous study, researcher identified hypoxia can induced the sumolytion of m^6^A enzyme(Hou et al., 2021), hypoxia-inducible factor-1 alpha (HIF-1α) can drive m^5^C modification to promote tumorigenesis(Wang J. Z. et al., 2021), m^1^A and m^6^A modification can significantly affect the infiltration of immune cells(Cai et al., 2021; Gao et al., 2021), m^7^G modification can drives immune evasion (Devarkar et al., 2016), and m^6^A, m^7^G modification can induce EMT in cancer development (Yu X. et al., 2020; Xia et al., 2021). Hence we investigate the correlation of RNA methylation with this risk score. In this study, the expression of WTAP, HNRNPA2B1, IGF2BP2, HNRNPC, CBLL1, ELAVL1, RBM15B, LRPPRC, ELAVL1, ALYREF, NSUN1, NSUN2, METTL1, BUD23, RNMT, METTL3, NSUN7 and NSUN6 differed significantly between the high-risk and low-risk groups. These results further support the value of our risk score formula.

Immune cells in the TME are associated with the development of cancer (Bruni et al., 2020). Our risk formula was highly correlated with markers of the immune microenvironment. Previous studies have reported that the characteristics of the immune microenvironment can predict sensitivity to immune checkpoint inhibitor treatment in patients with LUAD (Yu et al., 2020b; Park et al., 2020). In this study, patients in the low-risk group had higher immune activity. To validate the prognostic value of the risk score for immunotherapy sensitivity, we used two external datasets (GSE135222, GSE126044) containing information from patients with NSCLC treated with anti-PD-1/PD-L1 therapy. Our findings indicated that the risk score formula was associated with PFS and treatment responses in patients with NSCLC undergoing anti-PD-1/PD-L1 therapy. The IMvigor210 study examined the effect of PD-L1 inhibitor treatment in patients with metastatic urothelial cancer (Mariathasan et al., 2018; Wang S. et al., 2021). Because relatively few patients were enrolled in the GSE135222 and GSE126044 cohorts, we further evaluated the formula in the IMvigor210 cohort. Our results suggest that the risk score formula is not only suitable for predicting the effect of anti-PD-1/PD-L1 treatment in lung cancer, but that it may also be applicable in patients with other cancer types. Hence, a low-risk score may be an indicator for immunotherapy.

Previously, Sun et al. (2020) reported that the hypoxia-related signature could aid in predicting OS in patients with early-stage LUAD. However, whether hypoxia-related gene signatures can be used to develop a simple predictive formula for late-stage LUAD outcomes or immunotherapy sensitivity remains unknown. Other studies have also attempted to use immune- or EMT-related gene signatures to establish a prognostic model for LUAD (Tang et al., 2020; Wang et al., 2020). However, few of these studies used an authentic immune therapy cohort for validation. Given that multiple factors can have a substantial effect on the prognosis of LUAD and the intimate interconnections among hypoxia, immune responses, and EMT function, we aimed to establish a novel prognostic model based on the integration of multiple gene signatures. Our analysis indicated that the prognostic formula developed in this study exhibits precision for both early- and late-stage LUAD and is valid for predicting sensitivity to immunotherapy based on findings from a clinical cohort.

Currently, there are only a few methods for evaluating tumor sensitivity to chemotherapy (Rochigneux et al., 2020). In this study, the risk score was positively associated with drug sensitivity to cisplatin, docetaxel, paclitaxel and gemcitabine. This indicates that the risk score can be used to determine the appropriateness and benefit of chemotherapy in patients with LUAD, which may aid in the development of individualized treatment strategies.

This study also had some limitations. As the study was based on information within public databases, real-world prospective cohort studies are required to validate the risk score formula. The sequencing methods of the cohort included in this study were different, this may also affect the accuracy of this formula. Furthermore, most patients were Caucasian, highlighting the need to evaluate the predictive ability of the risk score in patients of other races.

## 5 Conclusion

In summary, this study established a novel 27-gene prognostic risk score for LUAD. The risk score was independently associated with OS in patients with LUAD and with functional analysis, tumor stemness, RNA methylation analysis, the immune microenvironment, and treatment response. Further, it accurately predicted prognosis in subgroups according to age, sex, and disease stage. These findings indicate that molecular risk stratification may be useful for predicting prognosis and guiding treatment in patients with LUAD.

## Data Availability

The datasets presented in this study can be found in online repositories. The names of the repository/repositories and accession number(s) can be found in the article/Supplementary Material.
