# Supplementary material for: A Prognostic Risk Score Based on Hypoxia-, Immunity-, and Epithelialto-Mesenchymal Transition-Related Genes for the Prognosis and Immunotherapy Response of Lung Adenocarcinoma
Source: Front Cell Dev Biol. 2022 Jan 24;9:758777. doi: 10.3389/fcell.2021.758777 (PMC8819669; doi:10.3389/fcell.2021.758777)
Supplement: Supplementary file 1 [file Table1.DOCX]

| **Supplementary Table 1 \| Clinical information of patients with LUAD in this study** | | | |
| --- | --- | --- | --- |
| Corhort | TCGA-LUAD | GSE68465 | GSE72094 |
| Number of patients | n=500 | n=442 | n=398 |
| Age(Mean±SD) | 65.26±10.05 | 64.39±10.09 | 69.36±9.45 |
| Follow up time (Mean±SD)(days) | 907.93±896.64 | 1600.05±1102.46 | 791.91±402.69 |
| Follow up status | |  |  |
| Alive | 318(63.6%) | 206(46.61%) | 285(71.61%) |
| Dead | 182(36.4%) | 236(53.39%) | 113(28.39%) |
| Gender |  |  |  |
| Male | 230(46%) | 223(50.45%) | 176(44.22%) |
| Female | 270(54%) | 219(49.55%) | 222(55.78%) |
| clinical Stage | |  |  |
| Stage I | 268(53.6%) | - | 254(63.82%) |
| Stage II | 119(23.8%) | - | 67(16.83%) |
| Stage III | 80(16%) | - | 57(14.32%) |
| Stage IV | 25(5%) | - | 15(3.77%) |
| Unknown | 8(1.6%) | - | 5(1.26%) |
| T stage |  |  |  |
| T1 | 167(33.4%) | - | - |
| T2 | 267(53.4%) | - | - |
| T3 | 45(9%) | - | - |
| T4 | 18(3.6%) | - | - |
| Unknown | 3(0.6%) | - | - |
| M stage |  |  |  |
| M0 | 332(66.4%) | - | - |
| M1 | 24(4.8%) | - | - |
| Unknown | 144(28.8%) | - | - |
| N stage |  |  |  |
| N0 | 324(64.8%) | - | - |
| N1 | 94(18.8%) | - | - |
| N2 | 69(13.8%) | - | - |
| N3 | 2(0.4%) | - | - |
| Unknown | 11(2.2%) | - | - |
| Smoking history | |  |  |
| Smoking up to now | | 32(7.24%) | - |
| Used to smoke | | 268(60.63%) 300(75.38%) | |
| No smoking history | | 49(11.09%) | 31(7.79%) |
| Unknown |  | 93(21.04%) | 67(16.83%) |
| Chemotherapy | |  |  |
| No |  | 340(76.92%) - | |
| Yes |  | 89(20.14%) | - |
| Unknown |  | 13(2.94%) | - |
| Histological grading | |  |  |
| Medium differentiation | | 209(47.29%) - | |
| High differentiation | | 60(13.57%) | - |
| Low differentiation | | 166(37.56%) - | |
| Unknown |  | 7(1.58%) | - |

LUAD: Lung Adenocarcinoma
