# Supplementary material for: A Prognostic Risk Score Based on Hypoxia-, Immunity-, and Epithelialto-Mesenchymal Transition-Related Genes for the Prognosis and Immunotherapy Response of Lung Adenocarcinoma
Source: Front Cell Dev Biol. 2022 Jan 24;9:758777. doi: 10.3389/fcell.2021.758777 (PMC8819669; doi:10.3389/fcell.2021.758777)
Supplement: Supplementary file 2 [file Table8.DOCX]

| **Supplementary Table 8 \| GO enrichment analysis of integrated-DEGs** | | | | |
| --- | --- | --- | --- | --- |
| Category | ID | Description | Count | qvalue |
| BP | GO:0006958 | complement activation, classical pathway | 88 | 2.27E-90 |
| BP | GO:0006959 | humoral immune response | 127 | 2.16E-89 |
| BP | GO:0006956 | complement activation | 93 | 5.25E-86 |
| BP | GO:0002455 | humoral immune response mediated by circulating immunoglobulin | 88 | 1.11E-85 |
| BP | GO:0002460 | adaptive immune response based on somatic recombination of immune receptors built from immunoglobulin superfamily domains | 118 | 7.43E-80 |
| BP | GO:0019724 | B cell mediated immunity | 97 | 1.34E-78 |
| BP | GO:0016064 | immunoglobulin mediated immune response | 96 | 5.90E-78 |
| BP | GO:0002449 | lymphocyte mediated immunity | 113 | 1.09E-74 |
| BP | GO:0006909 | phagocytosis | 114 | 1.33E-73 |
| BP | GO:0002377 | immunoglobulin production | 89 | 2.95E-72 |
| BP | GO:0002440 | production of molecular mediator of immune response | 101 | 1.11E-69 |
| BP | GO:0002429 | immune response-activating cell surface receptor signaling pathway | 109 | 1.05E-56 |
| BP | GO:0002757 | immune response-activating signal transduction | 109 | 1.05E-56 |
| BP | GO:0030449 | regulation of complement activation | 61 | 1.65E-56 |
| BP | GO:0006910 | phagocytosis, recognition | 54 | 1.09E-54 |
| BP | GO:0002920 | regulation of humoral immune response | 62 | 1.06E-52 |
| BP | GO:0002696 | positive regulation of leukocyte activation | 97 | 2.00E-52 |
| BP | GO:0050867 | positive regulation of cell activation | 98 | 6.67E-52 |
| BP | GO:0042742 | defense response to bacterium | 90 | 1.18E-51 |
| BP | GO:0002431 | Fc receptor mediated stimulatory signaling pathway | 63 | 1.52E-51 |
| BP | GO:0050871 | positive regulation of B cell activation | 64 | 1.52E-51 |
| BP | GO:0038094 | Fc-gamma receptor signaling pathway | 62 | 6.97E-51 |
| BP | GO:0050864 | regulation of B cell activation | 70 | 1.25E-50 |
| BP | GO:0006911 | phagocytosis, engulfment | 59 | 1.25E-50 |
| BP | GO:0002433 | immune response-regulating cell surface receptor signaling pathway involved in phagocytosis | 61 | 2.83E-50 |
| BP | GO:0038096 | Fc-gamma receptor signaling pathway involved in phagocytosis | 61 | 2.83E-50 |
| BP | GO:0002697 | regulation of immune effector process | 101 | 2.88E-50 |
| BP | GO:0051251 | positive regulation of lymphocyte activation | 89 | 9.42E-50 |
| BP | GO:0099024 | plasma membrane invagination | 59 | 1.72E-48 |
| BP | GO:0010324 | membrane invagination | 59 | 1.06E-46 |
| BP | GO:0050853 | B cell receptor signaling pathway | 57 | 8.86E-46 |
| BP | GO:0038095 | Fc-epsilon receptor signaling pathway | 62 | 1.92E-45 |
| BP | GO:0038093 | Fc receptor signaling pathway | 69 | 1.63E-42 |
| BP | GO:0008037 | cell recognition | 67 | 2.45E-42 |
| BP | GO:0006898 | receptor-mediated endocytosis | 78 | 8.72E-42 |
| BP | GO:0042113 | B cell activation | 77 | 8.40E-41 |
| BP | GO:0050851 | antigen receptor-mediated signaling pathway | 64 | 7.14E-29 |
| BP | GO:0060326 | cell chemotaxis | 62 | 2.94E-28 |
| BP | GO:0097529 | myeloid leukocyte migration | 50 | 3.56E-25 |
| BP | GO:0030198 | extracellular matrix organization | 61 | 1.38E-21 |
| BP | GO:0043062 | extracellular structure organization | 61 | 1.55E-21 |
| BP | GO:0030595 | leukocyte chemotaxis | 47 | 1.55E-21 |
| BP | GO:1990868 | response to chemokine | 31 | 2.73E-20 |
| BP | GO:1990869 | cellular response to chemokine | 31 | 2.73E-20 |
| BP | GO:0050920 | regulation of chemotaxis | 45 | 4.75E-20 |
| BP | GO:0070371 | ERK1 and ERK2 cascade | 53 | 8.41E-20 |
| BP | GO:0070374 | positive regulation of ERK1 and ERK2 cascade | 43 | 1.85E-19 |
| BP | GO:0070098 | chemokine-mediated signaling pathway | 29 | 2.08E-19 |
| BP | GO:0030593 | neutrophil chemotaxis | 30 | 2.34E-18 |
| BP | GO:0097530 | granulocyte migration | 35 | 4.87E-18 |
| BP | GO:0070372 | regulation of ERK1 and ERK2 cascade | 49 | 6.04E-18 |
| BP | GO:0007187 | G protein-coupled receptor signaling pathway, coupled to cyclic nucleotide second messenger | 45 | 1.34E-17 |
| BP | GO:0071621 | granulocyte chemotaxis | 32 | 1.50E-17 |
| BP | GO:1990266 | neutrophil migration | 31 | 4.13E-17 |
| BP | GO:0071674 | mononuclear cell migration | 28 | 4.92E-17 |
| BP | GO:0002548 | monocyte chemotaxis | 24 | 5.11E-17 |
| BP | GO:0007188 | adenylate cyclase-modulating G protein-coupled receptor signaling pathway | 41 | 1.78E-16 |
| BP | GO:0019730 | antimicrobial humoral response | 31 | 1.42E-15 |
| BP | GO:0050673 | epithelial cell proliferation | 56 | 2.54E-15 |
| BP | GO:0002685 | regulation of leukocyte migration | 37 | 1.01E-14 |
| BP | GO:0050727 | regulation of inflammatory response | 53 | 1.21E-14 |
| BP | GO:0001667 | ameboidal-type cell migration | 56 | 3.55E-14 |
| BP | GO:0050921 | positive regulation of chemotaxis | 30 | 4.83E-14 |
| BP | GO:0019932 | second-messenger-mediated signaling | 54 | 5.62E-14 |
| BP | GO:0001819 | positive regulation of cytokine production | 53 | 9.79E-14 |
| BP | GO:0032102 | negative regulation of response to external stimulus | 52 | 1.06E-13 |
| BP | GO:0032496 | response to lipopolysaccharide | 45 | 1.24E-13 |
| BP | GO:0001935 | endothelial cell proliferation | 34 | 2.91E-13 |
| BP | GO:0050679 | positive regulation of epithelial cell proliferation | 35 | 2.91E-13 |
| BP | GO:0002237 | response to molecule of bacterial origin | 45 | 1.27E-12 |
| BP | GO:0008217 | regulation of blood pressure | 32 | 1.69E-12 |
| BP | GO:0018108 | peptidyl-tyrosine phosphorylation | 46 | 1.76E-12 |
| BP | GO:0018212 | peptidyl-tyrosine modification | 46 | 2.33E-12 |
| BP | GO:0001938 | positive regulation of endothelial cell proliferation | 25 | 2.40E-12 |
| BP | GO:0048771 | tissue remodeling | 31 | 2.45E-12 |
| BP | GO:0040013 | negative regulation of locomotion | 47 | 3.67E-12 |
| BP | GO:0050729 | positive regulation of inflammatory response | 29 | 3.84E-12 |
| BP | GO:0051047 | positive regulation of secretion | 43 | 4.27E-12 |
| BP | GO:0051271 | negative regulation of cellular component movement | 47 | 4.67E-12 |
| BP | GO:0070661 | leukocyte proliferation | 41 | 4.85E-12 |
| BP | GO:0048660 | regulation of smooth muscle cell proliferation | 30 | 6.52E-12 |
| BP | GO:0019935 | cyclic-nucleotide-mediated signaling | 34 | 8.65E-12 |
| BP | GO:0048659 | smooth muscle cell proliferation | 30 | 8.70E-12 |
| BP | GO:0060389 | pathway-restricted SMAD protein phosphorylation | 19 | 1.01E-11 |
| BP | GO:0031349 | positive regulation of defense response | 45 | 1.77E-11 |
| BP | GO:0050918 | positive chemotaxis | 19 | 1.82E-11 |
| BP | GO:1903532 | positive regulation of secretion by cell | 40 | 2.01E-11 |
| BP | GO:0050731 | positive regulation of peptidyl-tyrosine phosphorylation | 31 | 2.65E-11 |
| BP | GO:0001936 | regulation of endothelial cell proliferation | 30 | 3.14E-11 |
| BP | GO:0050730 | regulation of peptidyl-tyrosine phosphorylation | 36 | 3.71E-11 |
| BP | GO:0050678 | regulation of epithelial cell proliferation | 45 | 4.10E-11 |
| BP | GO:0019933 | cAMP-mediated signaling | 31 | 4.41E-11 |
| BP | GO:0007204 | positive regulation of cytosolic calcium ion concentration | 40 | 4.74E-11 |
| BP | GO:0050919 | negative chemotaxis | 16 | 4.91E-11 |
| BP | GO:0002687 | positive regulation of leukocyte migration | 26 | 6.34E-11 |
| BP | GO:0010862 | positive regulation of pathway-restricted SMAD protein phosphorylation | 16 | 6.99E-11 |
| BP | GO:0051897 | positive regulation of protein kinase B signaling | 29 | 7.07E-11 |
| BP | GO:0055074 | calcium ion homeostasis | 49 | 8.68E-11 |
| BP | GO:0033002 | muscle cell proliferation | 34 | 8.69E-11 |
| BP | GO:0050922 | negative regulation of chemotaxis | 18 | 1.34E-10 |
| BP | GO:0007189 | adenylate cyclase-activating G protein-coupled receptor signaling pathway | 26 | 1.36E-10 |
| BP | GO:0007159 | leukocyte cell-cell adhesion | 42 | 1.39E-10 |
| BP | GO:0045785 | positive regulation of cell adhesion | 46 | 1.57E-10 |
| BP | GO:1901342 | regulation of vasculature development | 47 | 1.57E-10 |
| BP | GO:0001906 | cell killing | 28 | 1.60E-10 |
| BP | GO:0007631 | feeding behavior | 22 | 1.75E-10 |
| BP | GO:0048846 | axon extension involved in axon guidance | 14 | 2.03E-10 |
| BP | GO:1902284 | neuron projection extension involved in neuron projection guidance | 14 | 2.03E-10 |
| BP | GO:0070663 | regulation of leukocyte proliferation | 33 | 2.40E-10 |
| BP | GO:0045926 | negative regulation of growth | 34 | 2.46E-10 |
| BP | GO:0045765 | regulation of angiogenesis | 44 | 2.56E-10 |
| BP | GO:0051480 | regulation of cytosolic calcium ion concentration | 41 | 2.66E-10 |
| BP | GO:0001558 | regulation of cell growth | 45 | 2.76E-10 |
| BP | GO:0060393 | regulation of pathway-restricted SMAD protein phosphorylation | 17 | 4.24E-10 |
| BP | GO:0070482 | response to oxygen levels | 43 | 5.13E-10 |
| BP | GO:0001503 | ossification | 44 | 5.13E-10 |
| BP | GO:0032963 | collagen metabolic process | 22 | 7.35E-10 |
| BP | GO:0002688 | regulation of leukocyte chemotaxis | 23 | 7.35E-10 |
| BP | GO:0048247 | lymphocyte chemotaxis | 17 | 9.32E-10 |
| BP | GO:0050886 | endocrine process | 19 | 1.04E-09 |
| BP | GO:0061844 | antimicrobial humoral immune response mediated by antimicrobial peptide | 18 | 1.17E-09 |
| BP | GO:0006874 | cellular calcium ion homeostasis | 46 | 1.19E-09 |
| BP | GO:0016049 | cell growth | 48 | 1.23E-09 |
| BP | GO:0072676 | lymphocyte migration | 22 | 1.43E-09 |
| BP | GO:0003018 | vascular process in circulatory system | 28 | 1.53E-09 |
| BP | GO:0030308 | negative regulation of cell growth | 28 | 1.53E-09 |
| BP | GO:0010631 | epithelial cell migration | 40 | 1.80E-09 |
| BP | GO:0090132 | epithelium migration | 40 | 2.30E-09 |
| BP | GO:0045766 | positive regulation of angiogenesis | 29 | 2.68E-09 |
| BP | GO:0002699 | positive regulation of immune effector process | 30 | 3.12E-09 |
| BP | GO:0090130 | tissue migration | 40 | 3.67E-09 |
| BP | GO:0071356 | cellular response to tumor necrosis factor | 35 | 3.76E-09 |
| BP | GO:0072503 | cellular divalent inorganic cation homeostasis | 47 | 4.26E-09 |
| BP | GO:1903037 | regulation of leukocyte cell-cell adhesion | 37 | 4.26E-09 |
| BP | GO:0048841 | regulation of axon extension involved in axon guidance | 12 | 5.91E-09 |
| BP | GO:0002683 | negative regulation of immune system process | 45 | 5.91E-09 |
| BP | GO:0034341 | response to interferon-gamma | 28 | 6.08E-09 |
| BP | GO:0043434 | response to peptide hormone | 44 | 6.18E-09 |
| BP | GO:0031640 | killing of cells of other organism | 16 | 8.10E-09 |
| BP | GO:0071526 | semaphorin-plexin signaling pathway | 13 | 8.18E-09 |
| BP | GO:0036293 | response to decreased oxygen levels | 39 | 9.64E-09 |
| BP | GO:1903039 | positive regulation of leukocyte cell-cell adhesion | 30 | 1.04E-08 |
| BP | GO:1904018 | positive regulation of vasculature development | 30 | 1.04E-08 |
| BP | GO:0048754 | branching morphogenesis of an epithelial tube | 24 | 1.12E-08 |
| BP | GO:0035296 | regulation of tube diameter | 23 | 1.17E-08 |
| BP | GO:0097746 | regulation of blood vessel diameter | 23 | 1.17E-08 |
| BP | GO:0071346 | cellular response to interferon-gamma | 26 | 1.27E-08 |
| BP | GO:0001666 | response to hypoxia | 38 | 1.27E-08 |
| BP | GO:0035150 | regulation of tube size | 23 | 1.32E-08 |
| BP | GO:0046651 | lymphocyte proliferation | 33 | 1.46E-08 |
| BP | GO:0003044 | regulation of systemic arterial blood pressure mediated by a chemical signal | 14 | 1.48E-08 |
| BP | GO:1902668 | negative regulation of axon guidance | 11 | 1.65E-08 |
| BP | GO:0032943 | mononuclear cell proliferation | 33 | 1.88E-08 |
| BP | GO:2000146 | negative regulation of cell motility | 38 | 1.95E-08 |
| BP | GO:0009755 | hormone-mediated signaling pathway | 27 | 2.04E-08 |
| BP | GO:0050870 | positive regulation of T cell activation | 28 | 2.05E-08 |
| BP | GO:0030336 | negative regulation of cell migration | 37 | 2.09E-08 |
| BP | GO:0061138 | morphogenesis of a branching epithelium | 26 | 2.15E-08 |
| BP | GO:0034612 | response to tumor necrosis factor | 35 | 2.38E-08 |
| BP | GO:0034103 | regulation of tissue remodeling | 18 | 2.62E-08 |
| BP | GO:1902667 | regulation of axon guidance | 13 | 2.71E-08 |
| BP | GO:0048661 | positive regulation of smooth muscle cell proliferation | 19 | 3.13E-08 |
| BP | GO:0050670 | regulation of lymphocyte proliferation | 28 | 3.34E-08 |
| BP | GO:0032609 | interferon-gamma production | 20 | 3.40E-08 |
| BP | GO:0007200 | phospholipase C-activating G protein-coupled receptor signaling pathway | 19 | 3.64E-08 |
| BP | GO:0043406 | positive regulation of MAP kinase activity | 31 | 3.67E-08 |
| BP | GO:0032944 | regulation of mononuclear cell proliferation | 28 | 4.02E-08 |
| BP | GO:0002793 | positive regulation of peptide secretion | 26 | 4.02E-08 |
| BP | GO:0009612 | response to mechanical stimulus | 27 | 5.00E-08 |
| BP | GO:0003073 | regulation of systemic arterial blood pressure | 18 | 5.11E-08 |
| BP | GO:0002690 | positive regulation of leukocyte chemotaxis | 18 | 6.05E-08 |
| BP | GO:0010632 | regulation of epithelial cell migration | 33 | 6.18E-08 |
| BP | GO:0048762 | mesenchymal cell differentiation | 28 | 8.67E-08 |
| BP | GO:0140353 | lipid export from cell | 12 | 8.90E-08 |
| BP | GO:0060485 | mesenchyme development | 32 | 8.93E-08 |
| BP | GO:1902105 | regulation of leukocyte differentiation | 32 | 8.93E-08 |
| BP | GO:0090100 | positive regulation of transmembrane receptor protein serine/threonine kinase signaling pathway | 19 | 9.01E-08 |
| BP | GO:0001763 | morphogenesis of a branching structure | 26 | 9.09E-08 |
| BP | GO:0022407 | regulation of cell-cell adhesion | 41 | 9.21E-08 |
| BP | GO:0048843 | negative regulation of axon extension involved in axon guidance | 10 | 1.07E-07 |
| BP | GO:0060395 | SMAD protein signal transduction | 16 | 1.09E-07 |
| BP | GO:1903708 | positive regulation of hemopoiesis | 26 | 1.22E-07 |
| BP | GO:0022409 | positive regulation of cell-cell adhesion | 31 | 1.27E-07 |
| BP | GO:0046849 | bone remodeling | 17 | 1.63E-07 |
| BP | GO:0097755 | positive regulation of blood vessel diameter | 14 | 1.63E-07 |
| BP | GO:0032147 | activation of protein kinase activity | 34 | 1.75E-07 |
| BP | GO:0001823 | mesonephros development | 18 | 1.78E-07 |
| BP | GO:0071222 | cellular response to lipopolysaccharide | 26 | 1.78E-07 |
| BP | GO:0048640 | negative regulation of developmental growth | 19 | 1.81E-07 |
| BP | GO:0002822 | regulation of adaptive immune response based on somatic recombination of immune receptors built from immunoglobulin superfamily domains | 22 | 1.82E-07 |
| BP | GO:0051896 | regulation of protein kinase B signaling | 29 | 1.83E-07 |
| BP | GO:0050863 | regulation of T cell activation | 34 | 1.83E-07 |
| BP | GO:0043270 | positive regulation of ion transport | 31 | 1.83E-07 |
| BP | GO:0032649 | regulation of interferon-gamma production | 18 | 2.01E-07 |
| BP | GO:0098801 | regulation of renal system process | 11 | 2.02E-07 |
| BP | GO:0002819 | regulation of adaptive immune response | 23 | 2.13E-07 |
| BP | GO:0022617 | extracellular matrix disassembly | 16 | 2.15E-07 |
| BP | GO:0048638 | regulation of developmental growth | 35 | 2.48E-07 |
| BP | GO:0044060 | regulation of endocrine process | 12 | 2.59E-07 |
| BP | GO:0032613 | interleukin-10 production | 14 | 2.96E-07 |
| BP | GO:0046660 | female sex differentiation | 19 | 3.03E-07 |
| BP | GO:1902107 | positive regulation of leukocyte differentiation | 22 | 3.51E-07 |
| BP | GO:0043405 | regulation of MAP kinase activity | 34 | 3.64E-07 |
| BP | GO:0007178 | transmembrane receptor protein serine/threonine kinase signaling pathway | 35 | 3.70E-07 |
| BP | GO:0043491 | protein kinase B signaling | 30 | 3.86E-07 |
| BP | GO:0042110 | T cell activation | 42 | 4.13E-07 |
| BP | GO:0042509 | regulation of tyrosine phosphorylation of STAT protein | 16 | 4.18E-07 |
| BP | GO:0001657 | ureteric bud development | 17 | 4.59E-07 |
| BP | GO:0072163 | mesonephric epithelium development | 17 | 5.31E-07 |
| BP | GO:0072164 | mesonephric tubule development | 17 | 5.31E-07 |
| BP | GO:0060560 | developmental growth involved in morphogenesis | 27 | 5.54E-07 |
| BP | GO:0070665 | positive regulation of leukocyte proliferation | 21 | 5.65E-07 |
| BP | GO:0032755 | positive regulation of interleukin-6 production | 16 | 5.71E-07 |
| BP | GO:0048588 | developmental cell growth | 27 | 5.97E-07 |
| BP | GO:0071219 | cellular response to molecule of bacterial origin | 26 | 6.02E-07 |
| BP | GO:0061041 | regulation of wound healing | 21 | 6.24E-07 |
| BP | GO:0007260 | tyrosine phosphorylation of STAT protein | 16 | 6.62E-07 |
| BP | GO:0043542 | endothelial cell migration | 30 | 6.89E-07 |
| BP | GO:0002573 | myeloid leukocyte differentiation | 25 | 7.78E-07 |
| BP | GO:0044706 | multi-multicellular organism process | 26 | 8.44E-07 |
| BP | GO:0001990 | regulation of systemic arterial blood pressure by hormone | 11 | 8.44E-07 |
| BP | GO:1903706 | regulation of hemopoiesis | 42 | 9.04E-07 |
| BP | GO:0051216 | cartilage development | 24 | 9.04E-07 |
| BP | GO:1903034 | regulation of response to wounding | 23 | 9.47E-07 |
| BP | GO:0007171 | activation of transmembrane receptor protein tyrosine kinase activity | 7 | 9.95E-07 |
| BP | GO:0001894 | tissue homeostasis | 28 | 1.16E-06 |
| BP | GO:1905952 | regulation of lipid localization | 22 | 1.18E-06 |
| BP | GO:1904894 | positive regulation of receptor signaling pathway via STAT | 16 | 1.20E-06 |
| BP | GO:0046545 | development of primary female sexual characteristics | 17 | 1.20E-06 |
| BP | GO:0071216 | cellular response to biotic stimulus | 27 | 1.21E-06 |
| BP | GO:0061448 | connective tissue development | 28 | 1.23E-06 |
| BP | GO:0042531 | positive regulation of tyrosine phosphorylation of STAT protein | 14 | 1.28E-06 |
| BP | GO:0071902 | positive regulation of protein serine/threonine kinase activity | 33 | 1.28E-06 |
| BP | GO:0002285 | lymphocyte activation involved in immune response | 23 | 1.35E-06 |
| BP | GO:0046879 | hormone secretion | 31 | 1.53E-06 |
| BP | GO:0097696 | receptor signaling pathway via STAT | 22 | 1.55E-06 |
| BP | GO:0010634 | positive regulation of epithelial cell migration | 22 | 1.90E-06 |
| BP | GO:0002825 | regulation of T-helper 1 type immune response | 9 | 1.98E-06 |
| BP | GO:0006735 | NADH regeneration | 9 | 1.98E-06 |
| BP | GO:0061621 | canonical glycolysis | 9 | 1.98E-06 |
| BP | GO:0061718 | glucose catabolic process to pyruvate | 9 | 1.98E-06 |
| BP | GO:0002286 | T cell activation involved in immune response | 17 | 1.99E-06 |
| BP | GO:0110110 | positive regulation of animal organ morphogenesis | 15 | 2.00E-06 |
| BP | GO:0090092 | regulation of transmembrane receptor protein serine/threonine kinase signaling pathway | 27 | 2.20E-06 |
| BP | GO:0050671 | positive regulation of lymphocyte proliferation | 19 | 2.24E-06 |
| BP | GO:0010771 | negative regulation of cell morphogenesis involved in differentiation | 16 | 2.36E-06 |
| BP | GO:0032946 | positive regulation of mononuclear cell proliferation | 19 | 2.49E-06 |
| BP | GO:0002526 | acute inflammatory response | 17 | 2.54E-06 |
| BP | GO:1904892 | regulation of receptor signaling pathway via STAT | 20 | 2.56E-06 |
| BP | GO:0042554 | superoxide anion generation | 10 | 2.70E-06 |
| BP | GO:0009914 | hormone transport | 31 | 2.70E-06 |
| BP | GO:0061620 | glycolytic process through glucose-6-phosphate | 9 | 2.71E-06 |
| BP | GO:0035813 | regulation of renal sodium excretion | 8 | 2.85E-06 |
| BP | GO:0002702 | positive regulation of production of molecular mediator of immune response | 16 | 3.04E-06 |
| BP | GO:0008585 | female gonad development | 16 | 3.04E-06 |
| BP | GO:0042129 | regulation of T cell proliferation | 21 | 3.04E-06 |
| BP | GO:0001818 | negative regulation of cytokine production | 33 | 3.13E-06 |
| BP | GO:0010721 | negative regulation of cell development | 32 | 3.19E-06 |
| BP | GO:0042116 | macrophage activation | 16 | 3.44E-06 |
| BP | GO:0001755 | neural crest cell migration | 12 | 3.62E-06 |
| BP | GO:0061615 | glycolytic process through fructose-6-phosphate | 9 | 3.68E-06 |
| BP | GO:0046427 | positive regulation of receptor signaling pathway via JAK-STAT | 15 | 4.04E-06 |
| BP | GO:0046425 | regulation of receptor signaling pathway via JAK-STAT | 19 | 4.15E-06 |
| BP | GO:0060986 | endocrine hormone secretion | 11 | 4.29E-06 |
| BP | GO:0030282 | bone mineralization | 17 | 5.16E-06 |
| BP | GO:0097305 | response to alcohol | 25 | 5.22E-06 |
| BP | GO:0014032 | neural crest cell development | 14 | 5.25E-06 |
| BP | GO:0031341 | regulation of cell killing | 15 | 5.31E-06 |
| BP | GO:0042698 | ovulation cycle | 13 | 5.51E-06 |
| BP | GO:0050777 | negative regulation of immune response | 20 | 5.51E-06 |
| BP | GO:0007162 | negative regulation of cell adhesion | 29 | 5.66E-06 |
| BP | GO:0007190 | activation of adenylate cyclase activity | 10 | 5.67E-06 |
| BP | GO:0035812 | renal sodium excretion | 8 | 5.99E-06 |
| BP | GO:0050829 | defense response to Gram-negative bacterium | 14 | 5.99E-06 |
| BP | GO:0019731 | antibacterial humoral response | 12 | 6.24E-06 |
| BP | GO:0050771 | negative regulation of axonogenesis | 13 | 6.40E-06 |
| BP | GO:0002686 | negative regulation of leukocyte migration | 11 | 6.42E-06 |
| BP | GO:0051024 | positive regulation of immunoglobulin secretion | 6 | 6.64E-06 |
| BP | GO:0072593 | reactive oxygen species metabolic process | 28 | 7.20E-06 |
| BP | GO:0032653 | regulation of interleukin-10 production | 12 | 7.43E-06 |
| BP | GO:0019674 | NAD metabolic process | 11 | 7.84E-06 |
| BP | GO:0046850 | regulation of bone remodeling | 11 | 7.84E-06 |
| BP | GO:0030099 | myeloid cell differentiation | 36 | 7.88E-06 |
| BP | GO:0032368 | regulation of lipid transport | 18 | 8.20E-06 |
| BP | GO:0044062 | regulation of excretion | 8 | 8.36E-06 |
| BP | GO:0034694 | response to prostaglandin | 9 | 8.55E-06 |
| BP | GO:0007259 | receptor signaling pathway via JAK-STAT | 20 | 8.55E-06 |
| BP | GO:0050663 | cytokine secretion | 13 | 8.61E-06 |
| BP | GO:0006027 | glycosaminoglycan catabolic process | 12 | 8.69E-06 |
| BP | GO:0014031 | mesenchymal cell development | 14 | 8.94E-06 |
| BP | GO:0048864 | stem cell development | 14 | 8.94E-06 |
| BP | GO:0042098 | T cell proliferation | 22 | 9.21E-06 |
| BP | GO:0071675 | regulation of mononuclear cell migration | 11 | 9.33E-06 |
| BP | GO:0002703 | regulation of leukocyte mediated immunity | 23 | 9.50E-06 |
| BP | GO:0070555 | response to interleukin-1 | 23 | 9.50E-06 |
| BP | GO:0031667 | response to nutrient levels | 38 | 9.64E-06 |
| BP | GO:0035710 | CD4-positive, alpha-beta T cell activation | 15 | 9.64E-06 |
| BP | GO:0042100 | B cell proliferation | 15 | 9.64E-06 |
| BP | GO:0002791 | regulation of peptide secretion | 33 | 9.72E-06 |
| BP | GO:0007565 | female pregnancy | 22 | 9.78E-06 |
| BP | GO:0008361 | regulation of cell size | 21 | 9.97E-06 |
| BP | GO:0061387 | regulation of extent of cell growth | 16 | 1.10E-05 |
| BP | GO:1903557 | positive regulation of tumor necrosis factor superfamily cytokine production | 11 | 1.11E-05 |
| BP | GO:0050927 | positive regulation of positive chemotaxis | 8 | 1.12E-05 |
| BP | GO:0009615 | response to virus | 31 | 1.19E-05 |
| BP | GO:0030098 | lymphocyte differentiation | 32 | 1.27E-05 |
| BP | GO:0022604 | regulation of cell morphogenesis | 39 | 1.33E-05 |
| BP | GO:0030517 | negative regulation of axon extension | 10 | 1.33E-05 |
| BP | GO:0032964 | collagen biosynthetic process | 11 | 1.33E-05 |
| BP | GO:1903555 | regulation of tumor necrosis factor superfamily cytokine production | 16 | 1.37E-05 |
| BP | GO:0050770 | regulation of axonogenesis | 21 | 1.51E-05 |
| BP | GO:0050926 | regulation of positive chemotaxis | 8 | 1.53E-05 |
| BP | GO:0046883 | regulation of hormone secretion | 26 | 1.55E-05 |
| BP | GO:0042102 | positive regulation of T cell proliferation | 15 | 1.55E-05 |
| BP | GO:0045639 | positive regulation of myeloid cell differentiation | 15 | 1.55E-05 |
| BP | GO:0006968 | cellular defense response | 11 | 1.58E-05 |
| BP | GO:0009620 | response to fungus | 11 | 1.58E-05 |
| BP | GO:0002831 | regulation of response to biotic stimulus | 34 | 1.59E-05 |
| BP | GO:0042088 | T-helper 1 type immune response | 10 | 1.62E-05 |
| BP | GO:0050714 | positive regulation of protein secretion | 20 | 1.63E-05 |
| BP | GO:1990138 | neuron projection extension | 20 | 1.63E-05 |
| BP | GO:0014033 | neural crest cell differentiation | 14 | 1.66E-05 |
| BP | GO:0031345 | negative regulation of cell projection organization | 21 | 1.73E-05 |
| BP | GO:0032735 | positive regulation of interleukin-12 production | 9 | 1.74E-05 |
| BP | GO:0002700 | regulation of production of molecular mediator of immune response | 18 | 1.86E-05 |
| BP | GO:0030199 | collagen fibril organization | 11 | 1.86E-05 |
| BP | GO:0002021 | response to dietary excess | 8 | 2.00E-05 |
| BP | GO:0045672 | positive regulation of osteoclast differentiation | 8 | 2.00E-05 |
| BP | GO:0046887 | positive regulation of hormone secretion | 17 | 2.09E-05 |
| BP | GO:0002367 | cytokine production involved in immune response | 15 | 2.15E-05 |
| BP | GO:0006026 | aminoglycan catabolic process | 12 | 2.17E-05 |
| BP | GO:0035265 | organ growth | 21 | 2.17E-05 |
| BP | GO:0006007 | glucose catabolic process | 9 | 2.19E-05 |
| BP | GO:0002824 | positive regulation of adaptive immune response based on somatic recombination of immune receptors built from immunoglobulin superfamily domains | 15 | 2.41E-05 |
| BP | GO:0032303 | regulation of icosanoid secretion | 7 | 2.45E-05 |
| BP | GO:0071706 | tumor necrosis factor superfamily cytokine production | 16 | 2.53E-05 |
| BP | GO:0010769 | regulation of cell morphogenesis involved in differentiation | 28 | 2.56E-05 |
| BP | GO:0031214 | biomineral tissue development | 19 | 2.69E-05 |
| BP | GO:0110148 | biomineralization | 19 | 2.69E-05 |
| BP | GO:0061097 | regulation of protein tyrosine kinase activity | 14 | 2.70E-05 |
| BP | GO:0006953 | acute-phase response | 10 | 2.87E-05 |
| BP | GO:0022602 | ovulation cycle process | 10 | 2.87E-05 |
| BP | GO:0007599 | hemostasis | 30 | 2.90E-05 |
| BP | GO:0002763 | positive regulation of myeloid leukocyte differentiation | 11 | 3.06E-05 |
| BP | GO:0035815 | positive regulation of renal sodium excretion | 6 | 3.19E-05 |
| BP | GO:0009306 | protein secretion | 36 | 3.23E-05 |
| BP | GO:0048608 | reproductive structure development | 35 | 3.24E-05 |
| BP | GO:0002062 | chondrocyte differentiation | 15 | 3.30E-05 |
| BP | GO:0007229 | integrin-mediated signaling pathway | 15 | 3.30E-05 |
| BP | GO:0045619 | regulation of lymphocyte differentiation | 20 | 3.31E-05 |
| BP | GO:0035592 | establishment of protein localization to extracellular region | 36 | 3.34E-05 |
| BP | GO:0016051 | carbohydrate biosynthetic process | 22 | 3.35E-05 |
| BP | GO:0032733 | positive regulation of interleukin-10 production | 9 | 3.36E-05 |
| BP | GO:0033138 | positive regulation of peptidyl-serine phosphorylation | 15 | 3.65E-05 |
| BP | GO:1900046 | regulation of hemostasis | 13 | 3.70E-05 |
| BP | GO:0048675 | axon extension | 16 | 3.71E-05 |
| BP | GO:0001649 | osteoblast differentiation | 23 | 3.73E-05 |
| BP | GO:0002292 | T cell differentiation involved in immune response | 12 | 3.78E-05 |
| BP | GO:0061458 | reproductive system development | 35 | 3.81E-05 |
| BP | GO:0071347 | cellular response to interleukin-1 | 20 | 3.81E-05 |
| BP | GO:0002705 | positive regulation of leukocyte mediated immunity | 17 | 3.92E-05 |
| BP | GO:0071774 | response to fibroblast growth factor | 18 | 3.99E-05 |
| BP | GO:0002821 | positive regulation of adaptive immune response | 15 | 3.99E-05 |
| BP | GO:0032680 | regulation of tumor necrosis factor production | 15 | 3.99E-05 |
| BP | GO:0043312 | neutrophil degranulation | 37 | 4.02E-05 |
| BP | GO:0061098 | positive regulation of protein tyrosine kinase activity | 11 | 4.07E-05 |
| BP | GO:0051961 | negative regulation of nervous system development | 28 | 4.07E-05 |
| BP | GO:0032660 | regulation of interleukin-17 production | 9 | 4.07E-05 |
| BP | GO:1901654 | response to ketone | 21 | 4.08E-05 |
| BP | GO:0050764 | regulation of phagocytosis | 14 | 4.12E-05 |
| BP | GO:0033688 | regulation of osteoblast proliferation | 8 | 4.26E-05 |
| BP | GO:0010977 | negative regulation of neuron projection development | 18 | 4.28E-05 |
| BP | GO:0097485 | neuron projection guidance | 26 | 4.35E-05 |
| BP | GO:0003014 | renal system process | 16 | 4.36E-05 |
| BP | GO:0071692 | protein localization to extracellular region | 36 | 4.39E-05 |
| BP | GO:0002283 | neutrophil activation involved in immune response | 37 | 4.49E-05 |
| BP | GO:0055093 | response to hyperoxia | 7 | 4.51E-05 |
| BP | GO:1990845 | adaptive thermogenesis | 18 | 4.61E-05 |
| BP | GO:0031343 | positive regulation of cell killing | 11 | 4.65E-05 |
| BP | GO:0001774 | microglial cell activation | 10 | 4.65E-05 |
| BP | GO:0002269 | leukocyte activation involved in inflammatory response | 10 | 4.65E-05 |
| BP | GO:0032760 | positive regulation of tumor necrosis factor production | 10 | 4.65E-05 |
| BP | GO:0048511 | rhythmic process | 27 | 4.91E-05 |
| BP | GO:0000187 | activation of MAPK activity | 18 | 4.97E-05 |
| BP | GO:0050830 | defense response to Gram-positive bacterium | 14 | 5.06E-05 |
| BP | GO:0098868 | bone growth | 8 | 5.36E-05 |
| BP | GO:0002294 | CD4-positive, alpha-beta T cell differentiation involved in immune response | 11 | 5.37E-05 |
| BP | GO:0072073 | kidney epithelium development | 17 | 5.39E-05 |
| BP | GO:0045088 | regulation of innate immune response | 27 | 5.46E-05 |
| BP | GO:0010466 | negative regulation of peptidase activity | 25 | 5.52E-05 |
| BP | GO:0010594 | regulation of endothelial cell migration | 23 | 5.60E-05 |
| BP | GO:0030203 | glycosaminoglycan metabolic process | 18 | 5.83E-05 |
| BP | GO:0006734 | NADH metabolic process | 9 | 5.93E-05 |
| BP | GO:0001659 | temperature homeostasis | 19 | 6.07E-05 |
| BP | GO:0031100 | animal organ regeneration | 12 | 6.13E-05 |
| BP | GO:0002287 | alpha-beta T cell activation involved in immune response | 11 | 6.13E-05 |
| BP | GO:0002293 | alpha-beta T cell differentiation involved in immune response | 11 | 6.13E-05 |
| BP | GO:2000514 | regulation of CD4-positive, alpha-beta T cell activation | 11 | 6.13E-05 |
| BP | GO:0032535 | regulation of cellular component size | 31 | 6.22E-05 |
| BP | GO:0048545 | response to steroid hormone | 29 | 6.23E-05 |
| BP | GO:0032675 | regulation of interleukin-6 production | 18 | 6.23E-05 |
| BP | GO:0014068 | positive regulation of phosphatidylinositol 3-kinase signaling | 13 | 6.34E-05 |
| BP | GO:0071559 | response to transforming growth factor beta | 24 | 6.63E-05 |
| BP | GO:0032640 | tumor necrosis factor production | 15 | 6.97E-05 |
| BP | GO:0045453 | bone resorption | 11 | 7.06E-05 |
| BP | GO:0032308 | positive regulation of prostaglandin secretion | 5 | 7.38E-05 |
| BP | GO:0050768 | negative regulation of neurogenesis | 26 | 7.41E-05 |
| BP | GO:0044344 | cellular response to fibroblast growth factor stimulus | 17 | 7.46E-05 |
| BP | GO:0150076 | neuroinflammatory response | 12 | 7.88E-05 |
| BP | GO:0070542 | response to fatty acid | 13 | 7.99E-05 |
| BP | GO:0007588 | excretion | 11 | 8.10E-05 |
| BP | GO:0032729 | positive regulation of interferon-gamma production | 11 | 8.10E-05 |
| BP | GO:0002456 | T cell mediated immunity | 14 | 8.44E-05 |
| BP | GO:0007548 | sex differentiation | 25 | 8.60E-05 |
| BP | GO:0045637 | regulation of myeloid cell differentiation | 24 | 8.88E-05 |
| BP | GO:0042310 | vasoconstriction | 12 | 8.88E-05 |
| BP | GO:0030278 | regulation of ossification | 21 | 8.93E-05 |
| BP | GO:0031960 | response to corticosteroid | 18 | 9.19E-05 |
| BP | GO:0050728 | negative regulation of inflammatory response | 20 | 9.20E-05 |
| BP | GO:0031649 | heat generation | 6 | 9.86E-05 |
| BP | GO:0032305 | positive regulation of icosanoid secretion | 6 | 9.86E-05 |
| BP | GO:0032620 | interleukin-17 production | 9 | 0.000101956 |
| BP | GO:0002720 | positive regulation of cytokine production involved in immune response | 10 | 0.000102361 |
| BP | GO:0032655 | regulation of interleukin-12 production | 10 | 0.000102361 |
| BP | GO:0050715 | positive regulation of cytokine secretion | 8 | 0.000102361 |
| BP | GO:0007411 | axon guidance | 25 | 0.000106645 |
| BP | GO:0010959 | regulation of metal ion transport | 31 | 0.000112687 |
| BP | GO:0031589 | cell-substrate adhesion | 29 | 0.000117362 |
| BP | GO:0070997 | neuron death | 29 | 0.000123345 |
| BP | GO:1905954 | positive regulation of lipid localization | 13 | 0.000123376 |
| BP | GO:0032306 | regulation of prostaglandin secretion | 5 | 0.000123801 |
| BP | GO:0031348 | negative regulation of defense response | 24 | 0.000124324 |
| BP | GO:0001909 | leukocyte mediated cytotoxicity | 14 | 0.000124562 |
| BP | GO:0071887 | leukocyte apoptotic process | 14 | 0.000124562 |
| BP | GO:0033687 | osteoblast proliferation | 8 | 0.000125802 |
| BP | GO:0071560 | cellular response to transforming growth factor beta stimulus | 23 | 0.000127615 |
| BP | GO:0031099 | regeneration | 20 | 0.000127903 |
| BP | GO:0002761 | regulation of myeloid leukocyte differentiation | 15 | 0.000131495 |
| BP | GO:0007596 | blood coagulation | 28 | 0.000131821 |
| BP | GO:0003081 | regulation of systemic arterial blood pressure by renin-angiotensin | 7 | 0.00013219 |
| BP | GO:0090025 | regulation of monocyte chemotaxis | 7 | 0.00013219 |
| BP | GO:0048662 | negative regulation of smooth muscle cell proliferation | 11 | 0.000135024 |
| BP | GO:0001541 | ovarian follicle development | 10 | 0.000135024 |
| BP | GO:0032615 | interleukin-12 production | 10 | 0.000135024 |
| BP | GO:1905517 | macrophage migration | 10 | 0.000135024 |
| BP | GO:0032740 | positive regulation of interleukin-17 production | 6 | 0.000135024 |
| BP | GO:0051023 | regulation of immunoglobulin secretion | 6 | 0.000135024 |
| BP | GO:2000831 | regulation of steroid hormone secretion | 6 | 0.000135024 |
| BP | GO:0002706 | regulation of lymphocyte mediated immunity | 17 | 0.000137464 |
| BP | GO:0030193 | regulation of blood coagulation | 12 | 0.000138322 |
| BP | GO:0032635 | interleukin-6 production | 18 | 0.000139492 |
| BP | GO:0030509 | BMP signaling pathway | 17 | 0.000148621 |
| BP | GO:0009595 | detection of biotic stimulus | 8 | 0.000150391 |
| BP | GO:2000516 | positive regulation of CD4-positive, alpha-beta T cell activation | 8 | 0.000150391 |
| BP | GO:0032370 | positive regulation of lipid transport | 11 | 0.000151954 |
| BP | GO:0002718 | regulation of cytokine production involved in immune response | 12 | 0.00015471 |
| BP | GO:0090287 | regulation of cellular response to growth factor stimulus | 26 | 0.000157111 |
| BP | GO:0072001 | renal system development | 25 | 0.000158786 |
| BP | GO:0006022 | aminoglycan metabolic process | 18 | 0.000160632 |
| BP | GO:0030516 | regulation of axon extension | 13 | 0.000162565 |
| BP | GO:0030574 | collagen catabolic process | 9 | 0.000163428 |
| BP | GO:0045776 | negative regulation of blood pressure | 9 | 0.000163428 |
| BP | GO:0030072 | peptide hormone secretion | 23 | 0.000164467 |
| BP | GO:0050817 | coagulation | 28 | 0.000170752 |
| BP | GO:0051607 | defense response to virus | 23 | 0.000174166 |
| BP | GO:0052547 | regulation of peptidase activity | 34 | 0.00017617 |
| BP | GO:0050707 | regulation of cytokine secretion | 10 | 0.000176667 |
| BP | GO:0030316 | osteoclast differentiation | 13 | 0.000178586 |
| BP | GO:0051048 | negative regulation of secretion | 19 | 0.000178845 |
| BP | GO:2000273 | positive regulation of signaling receptor activity | 8 | 0.000179524 |
| BP | GO:0014066 | regulation of phosphatidylinositol 3-kinase signaling | 15 | 0.000179524 |
| BP | GO:0050866 | negative regulation of cell activation | 20 | 0.000181616 |
| BP | GO:0030168 | platelet activation | 17 | 0.000182254 |
| BP | GO:0002024 | diet induced thermogenesis | 5 | 0.000187177 |
| BP | GO:0033690 | positive regulation of osteoblast proliferation | 5 | 0.000187177 |
| BP | GO:0014911 | positive regulation of smooth muscle cell migration | 9 | 0.000189394 |
| BP | GO:0045665 | negative regulation of neuron differentiation | 21 | 0.000191778 |
| BP | GO:0019229 | regulation of vasoconstriction | 10 | 0.000199633 |
| BP | GO:0032732 | positive regulation of interleukin-1 production | 10 | 0.000199633 |
| BP | GO:0042093 | T-helper cell differentiation | 10 | 0.000199633 |
| BP | GO:0061900 | glial cell activation | 10 | 0.000199633 |
| BP | GO:2000191 | regulation of fatty acid transport | 7 | 0.000205175 |
| BP | GO:0014910 | regulation of smooth muscle cell migration | 12 | 0.000209287 |
| BP | GO:0070664 | negative regulation of leukocyte proliferation | 12 | 0.000209287 |
| BP | GO:0097756 | negative regulation of blood vessel diameter | 12 | 0.000209287 |
| BP | GO:0032689 | negative regulation of interferon-gamma production | 8 | 0.0002134 |
| BP | GO:0045807 | positive regulation of endocytosis | 13 | 0.0002134 |
| BP | GO:0002673 | regulation of acute inflammatory response | 9 | 0.000219096 |
| BP | GO:0010712 | regulation of collagen metabolic process | 9 | 0.000219096 |
| BP | GO:0033135 | regulation of peptidyl-serine phosphorylation | 16 | 0.00022035 |
| BP | GO:0060419 | heart growth | 14 | 0.000225443 |
| BP | GO:1904705 | regulation of vascular associated smooth muscle cell proliferation | 12 | 0.000231561 |
| BP | GO:1990874 | vascular associated smooth muscle cell proliferation | 12 | 0.000231561 |
| BP | GO:0007568 | aging | 26 | 0.000236941 |
| BP | GO:0046631 | alpha-beta T cell activation | 16 | 0.000237404 |
| BP | GO:0006801 | superoxide metabolic process | 11 | 0.000238082 |
| BP | GO:0045137 | development of primary sexual characteristics | 21 | 0.000238313 |
| BP | GO:0051767 | nitric-oxide synthase biosynthetic process | 6 | 0.000238313 |
| BP | GO:0051769 | regulation of nitric-oxide synthase biosynthetic process | 6 | 0.000238313 |
| BP | GO:2000193 | positive regulation of fatty acid transport | 6 | 0.000238313 |
| BP | GO:1901215 | negative regulation of neuron death | 20 | 0.000240938 |
| BP | GO:0001822 | kidney development | 24 | 0.000244451 |
| BP | GO:0036296 | response to increased oxygen levels | 7 | 0.000250506 |
| BP | GO:0071624 | positive regulation of granulocyte chemotaxis | 7 | 0.000250506 |
| BP | GO:0007160 | cell-matrix adhesion | 21 | 0.000250506 |
| BP | GO:1904707 | positive regulation of vascular associated smooth muscle cell proliferation | 9 | 0.000250506 |
| BP | GO:0050818 | regulation of coagulation | 12 | 0.000252783 |
| BP | GO:0055024 | regulation of cardiac muscle tissue development | 13 | 0.000252889 |
| BP | GO:0030888 | regulation of B cell proliferation | 10 | 0.000253087 |
| BP | GO:1901653 | cellular response to peptide | 30 | 0.000261635 |
| BP | GO:0001910 | regulation of leukocyte mediated cytotoxicity | 11 | 0.000262407 |
| BP | GO:1904019 | epithelial cell apoptotic process | 14 | 0.000262407 |
| BP | GO:1902893 | regulation of pri-miRNA transcription by RNA polymerase II | 9 | 0.000291082 |
| BP | GO:0010876 | lipid localization | 32 | 0.000291393 |
| BP | GO:0043524 | negative regulation of neuron apoptotic process | 16 | 0.000292746 |
| BP | GO:1903524 | positive regulation of blood circulation | 11 | 0.000294875 |
| BP | GO:0032892 | positive regulation of organic acid transport | 8 | 0.000297098 |
| BP | GO:1902895 | positive regulation of pri-miRNA transcription by RNA polymerase II | 8 | 0.000297098 |
| BP | GO:0002833 | positive regulation of response to biotic stimulus | 22 | 0.000299897 |
| BP | GO:0035929 | steroid hormone secretion | 6 | 0.000310416 |
| BP | GO:0048305 | immunoglobulin secretion | 6 | 0.000310416 |
| BP | GO:0061213 | positive regulation of mesonephros development | 6 | 0.000310416 |
| BP | GO:0042063 | gliogenesis | 25 | 0.000318308 |
| BP | GO:0045621 | positive regulation of lymphocyte differentiation | 13 | 0.000333271 |
| BP | GO:0071622 | regulation of granulocyte chemotaxis | 9 | 0.000333271 |
| BP | GO:0051924 | regulation of calcium ion transport | 22 | 0.000333271 |
| BP | GO:0006094 | gluconeogenesis | 12 | 0.00034052 |
| BP | GO:0002695 | negative regulation of leukocyte activation | 18 | 0.000342732 |
| BP | GO:0050832 | defense response to fungus | 8 | 0.000350773 |
| BP | GO:0071772 | response to BMP | 17 | 0.000354701 |
| BP | GO:0071773 | cellular response to BMP stimulus | 17 | 0.000354701 |
| BP | GO:0009135 | purine nucleoside diphosphate metabolic process | 15 | 0.000354701 |
| BP | GO:0009179 | purine ribonucleoside diphosphate metabolic process | 15 | 0.000354701 |
| BP | GO:0002708 | positive regulation of lymphocyte mediated immunity | 13 | 0.000360176 |
| BP | GO:0007218 | neuropeptide signaling pathway | 13 | 0.000360176 |
| BP | GO:2000379 | positive regulation of reactive oxygen species metabolic process | 13 | 0.000360176 |
| BP | GO:0043367 | CD4-positive, alpha-beta T cell differentiation | 11 | 0.000361901 |
| BP | GO:0061614 | pri-miRNA transcription by RNA polymerase II | 9 | 0.000380547 |
| BP | GO:0001655 | urogenital system development | 26 | 0.00038288 |
| BP | GO:0045834 | positive regulation of lipid metabolic process | 16 | 0.000384902 |
| BP | GO:0032310 | prostaglandin secretion | 5 | 0.000391057 |
| BP | GO:0050930 | induction of positive chemotaxis | 5 | 0.000391057 |
| BP | GO:0060732 | positive regulation of inositol phosphate biosynthetic process | 5 | 0.000391057 |
| BP | GO:0055017 | cardiac muscle tissue growth | 13 | 0.000391617 |
| BP | GO:0051928 | positive regulation of calcium ion transport | 14 | 0.000392292 |
| BP | GO:0035809 | regulation of urine volume | 6 | 0.000396372 |
| BP | GO:0060562 | epithelial tube morphogenesis | 26 | 0.000396616 |
| BP | GO:0060348 | bone development | 19 | 0.000404269 |
| BP | GO:0048246 | macrophage chemotaxis | 8 | 0.000404269 |
| BP | GO:0014909 | smooth muscle cell migration | 12 | 0.000404269 |
| BP | GO:1903035 | negative regulation of response to wounding | 12 | 0.000404269 |
| BP | GO:0014065 | phosphatidylinositol 3-kinase signaling | 16 | 0.000406491 |
| BP | GO:0051250 | negative regulation of lymphocyte activation | 16 | 0.000406491 |
| BP | GO:0006024 | glycosaminoglycan biosynthetic process | 13 | 0.000423817 |
| BP | GO:0007584 | response to nutrient | 17 | 0.000424616 |
| BP | GO:0050708 | regulation of protein secretion | 27 | 0.000426592 |
| BP | GO:0009185 | ribonucleoside diphosphate metabolic process | 15 | 0.000436465 |
| BP | GO:0008406 | gonad development | 20 | 0.000440454 |
| BP | GO:0019319 | hexose biosynthetic process | 12 | 0.000441331 |
| BP | GO:0045778 | positive regulation of ossification | 12 | 0.000441331 |
| BP | GO:1904035 | regulation of epithelial cell apoptotic process | 12 | 0.000441331 |
| BP | GO:0002063 | chondrocyte development | 7 | 0.000442814 |
| BP | GO:0010743 | regulation of macrophage derived foam cell differentiation | 7 | 0.000442814 |
| BP | GO:0061036 | positive regulation of cartilage development | 7 | 0.000442814 |
| BP | GO:0045123 | cellular extravasation | 10 | 0.00045066 |
| BP | GO:0045670 | regulation of osteoclast differentiation | 10 | 0.00045066 |
| BP | GO:0046031 | ADP metabolic process | 14 | 0.000450696 |
| BP | GO:0051346 | negative regulation of hydrolase activity | 33 | 0.000451426 |
| BP | GO:0006939 | smooth muscle contraction | 13 | 0.000453419 |
| BP | GO:2000278 | regulation of DNA biosynthetic process | 13 | 0.000453419 |
| BP | GO:0045124 | regulation of bone resorption | 8 | 0.000464511 |
| BP | GO:1901343 | negative regulation of vasculature development | 19 | 0.000472701 |
| BP | GO:0072677 | eosinophil migration | 6 | 0.000496371 |
| BP | GO:0032355 | response to estradiol | 15 | 0.000498686 |
| BP | GO:0050766 | positive regulation of phagocytosis | 10 | 0.00050447 |
| BP | GO:0002691 | regulation of cellular extravasation | 7 | 0.000532452 |
| BP | GO:0043552 | positive regulation of phosphatidylinositol 3-kinase activity | 7 | 0.000532452 |
| BP | GO:0090022 | regulation of neutrophil chemotaxis | 7 | 0.000532452 |
| BP | GO:2000846 | regulation of corticosteroid hormone secretion | 5 | 0.000534742 |
| BP | GO:0048015 | phosphatidylinositol-mediated signaling | 18 | 0.000541421 |
| BP | GO:0032965 | regulation of collagen biosynthetic process | 8 | 0.000541508 |
| BP | GO:0019320 | hexose catabolic process | 9 | 0.000547316 |
| BP | GO:0031295 | T cell costimulation | 9 | 0.000547316 |
| BP | GO:0060688 | regulation of morphogenesis of a branching structure | 9 | 0.000547316 |
| BP | GO:0046634 | regulation of alpha-beta T cell activation | 12 | 0.000575907 |
| BP | GO:1901214 | regulation of neuron death | 25 | 0.000580546 |
| BP | GO:0048708 | astrocyte differentiation | 11 | 0.000589262 |
| BP | GO:1903522 | regulation of blood circulation | 24 | 0.000609382 |
| BP | GO:0106106 | cold-induced thermogenesis | 15 | 0.000616013 |
| BP | GO:0120161 | regulation of cold-induced thermogenesis | 15 | 0.000616013 |
| BP | GO:0007409 | axonogenesis | 33 | 0.000619558 |
| BP | GO:0003094 | glomerular filtration | 6 | 0.000620165 |
| BP | GO:0032753 | positive regulation of interleukin-4 production | 6 | 0.000620165 |
| BP | GO:0034695 | response to prostaglandin E | 6 | 0.000620165 |
| BP | GO:0002639 | positive regulation of immunoglobulin production | 8 | 0.000622687 |
| BP | GO:0034105 | positive regulation of tissue remodeling | 8 | 0.000622687 |
| BP | GO:0006879 | cellular iron ion homeostasis | 10 | 0.000622687 |
| BP | GO:0050795 | regulation of behavior | 10 | 0.000622687 |
| BP | GO:0010001 | glial cell differentiation | 20 | 0.000626069 |
| BP | GO:0002888 | positive regulation of myeloid leukocyte mediated immunity | 7 | 0.000626069 |
| BP | GO:0032814 | regulation of natural killer cell activation | 7 | 0.000626069 |
| BP | GO:0045089 | positive regulation of innate immune response | 19 | 0.00065577 |
| BP | GO:0048738 | cardiac muscle tissue development | 20 | 0.000661034 |
| BP | GO:0048017 | inositol lipid-mediated signaling | 18 | 0.000674935 |
| BP | GO:0060759 | regulation of response to cytokine stimulus | 18 | 0.000674935 |
| BP | GO:0006006 | glucose metabolic process | 19 | 0.000692736 |
| BP | GO:0031294 | lymphocyte costimulation | 9 | 0.000694457 |
| BP | GO:0043551 | regulation of phosphatidylinositol 3-kinase activity | 9 | 0.000694457 |
| BP | GO:1903793 | positive regulation of anion transport | 9 | 0.000694457 |
| BP | GO:0030101 | natural killer cell activation | 11 | 0.000704886 |
| BP | GO:0010919 | regulation of inositol phosphate biosynthetic process | 5 | 0.000714156 |
| BP | GO:0035930 | corticosteroid hormone secretion | 5 | 0.000714156 |
| BP | GO:0051770 | positive regulation of nitric-oxide synthase biosynthetic process | 5 | 0.000714156 |
| BP | GO:0006023 | aminoglycan biosynthetic process | 13 | 0.00072912 |
| BP | GO:0006096 | glycolytic process | 13 | 0.00072912 |
| BP | GO:0046364 | monosaccharide biosynthetic process | 12 | 0.000734694 |
| BP | GO:0016486 | peptide hormone processing | 7 | 0.000738921 |
| BP | GO:0140448 | signaling receptor ligand precursor processing | 7 | 0.000738921 |
| BP | GO:0002092 | positive regulation of receptor internalization | 6 | 0.000755779 |
| BP | GO:0003071 | renal system process involved in regulation of systemic arterial blood pressure | 6 | 0.000755779 |
| BP | GO:0060259 | regulation of feeding behavior | 6 | 0.000755779 |
| BP | GO:0061217 | regulation of mesonephros development | 6 | 0.000755779 |
| BP | GO:0097205 | renal filtration | 6 | 0.000755779 |
| BP | GO:0061035 | regulation of cartilage development | 10 | 0.000758921 |
| BP | GO:0007517 | muscle organ development | 29 | 0.000761311 |
| BP | GO:0007193 | adenylate cyclase-inhibiting G protein-coupled receptor signaling pathway | 11 | 0.000761396 |
| BP | GO:0050772 | positive regulation of axonogenesis | 11 | 0.000761396 |
| BP | GO:2000106 | regulation of leukocyte apoptotic process | 11 | 0.000761396 |
| BP | GO:0001658 | branching involved in ureteric bud morphogenesis | 9 | 0.000769794 |
| BP | GO:0006757 | ATP generation from ADP | 13 | 0.000777148 |
| BP | GO:0030217 | T cell differentiation | 21 | 0.000782957 |
| BP | GO:0015908 | fatty acid transport | 12 | 0.000786733 |
| BP | GO:0060191 | regulation of lipase activity | 12 | 0.000786733 |
| BP | GO:0010595 | positive regulation of endothelial cell migration | 14 | 0.00079106 |
| BP | GO:0000302 | response to reactive oxygen species | 20 | 0.000791573 |
| BP | GO:0010810 | regulation of cell-substrate adhesion | 19 | 0.000791581 |
| BP | GO:2000027 | regulation of animal organ morphogenesis | 21 | 0.000819177 |
| BP | GO:2000377 | regulation of reactive oxygen species metabolic process | 18 | 0.000825486 |
| BP | GO:0060420 | regulation of heart growth | 11 | 0.000830389 |
| BP | GO:0002709 | regulation of T cell mediated immunity | 10 | 0.000830389 |
| BP | GO:0010517 | regulation of phospholipase activity | 10 | 0.000830389 |
| BP | GO:0001959 | regulation of cytokine-mediated signaling pathway | 17 | 0.000843472 |
| BP | GO:0006165 | nucleoside diphosphate phosphorylation | 14 | 0.000845068 |
| BP | GO:1905332 | positive regulation of morphogenesis of an epithelium | 7 | 0.000854657 |
| BP | GO:0030900 | forebrain development | 28 | 0.000876965 |
| BP | GO:0045580 | regulation of T cell differentiation | 15 | 0.000887982 |
| BP | GO:1904036 | negative regulation of epithelial cell apoptotic process | 8 | 0.000916205 |
| BP | GO:0051873 | killing by host of symbiont cells | 6 | 0.000916205 |
| BP | GO:0090023 | positive regulation of neutrophil chemotaxis | 6 | 0.000916205 |
| BP | GO:0015732 | prostaglandin transport | 5 | 0.000927515 |
| BP | GO:0006869 | lipid transport | 28 | 0.000945341 |
| BP | GO:0046939 | nucleotide phosphorylation | 14 | 0.000975738 |
| BP | GO:0010951 | negative regulation of endopeptidase activity | 21 | 0.000991044 |
| BP | GO:0045730 | respiratory burst | 7 | 0.00100626 |
| BP | GO:0090218 | positive regulation of lipid kinase activity | 7 | 0.00100626 |
| BP | GO:0006090 | pyruvate metabolic process | 15 | 0.001012069 |
| BP | GO:0032602 | chemokine production | 10 | 0.001015497 |
| BP | GO:0043627 | response to estrogen | 10 | 0.001015497 |
| BP | GO:0061383 | trabecula morphogenesis | 8 | 0.001044537 |
| BP | GO:2000107 | negative regulation of leukocyte apoptotic process | 8 | 0.001044537 |
| BP | GO:0006979 | response to oxidative stress | 31 | 0.001078599 |
| BP | GO:0010518 | positive regulation of phospholipase activity | 9 | 0.001078599 |
| BP | GO:0002524 | hypersensitivity | 4 | 0.001085641 |
| BP | GO:0030388 | fructose 1,6-bisphosphate metabolic process | 4 | 0.001085641 |
| BP | GO:0042756 | drinking behavior | 4 | 0.001085641 |
| BP | GO:0003416 | endochondral bone growth | 6 | 0.001115133 |
| BP | GO:0033555 | multicellular organismal response to stress | 10 | 0.001119018 |
| BP | GO:0032612 | interleukin-1 production | 13 | 0.001119703 |
| BP | GO:0010742 | macrophage derived foam cell differentiation | 7 | 0.001170844 |
| BP | GO:0090077 | foam cell differentiation | 7 | 0.001170844 |
| BP | GO:0014812 | muscle cell migration | 12 | 0.001171138 |
| BP | GO:0019233 | sensory perception of pain | 12 | 0.001171138 |
| BP | GO:0046632 | alpha-beta T cell differentiation | 12 | 0.001171138 |
| BP | GO:0007369 | gastrulation | 17 | 0.001180095 |
| BP | GO:0010718 | positive regulation of epithelial to mesenchymal transition | 8 | 0.001180095 |
| BP | GO:0046330 | positive regulation of JNK cascade | 14 | 0.001184716 |
| BP | GO:0046635 | positive regulation of alpha-beta T cell activation | 9 | 0.001193921 |
| BP | GO:0032656 | regulation of interleukin-13 production | 5 | 0.001195137 |
| BP | GO:0090190 | positive regulation of branching involved in ureteric bud morphogenesis | 5 | 0.001195137 |
| BP | GO:0009132 | nucleoside diphosphate metabolic process | 15 | 0.001209258 |
| BP | GO:0030500 | regulation of bone mineralization | 10 | 0.001219567 |
| BP | GO:0062197 | cellular response to chemical stress | 26 | 0.001219567 |
| BP | GO:0051051 | negative regulation of transport | 32 | 0.001219567 |
| BP | GO:0051402 | neuron apoptotic process | 20 | 0.001276219 |
| BP | GO:0045582 | positive regulation of T cell differentiation | 11 | 0.001276219 |
| BP | GO:0048732 | gland development | 30 | 0.001293485 |
| BP | GO:0006936 | muscle contraction | 26 | 0.001320075 |
| BP | GO:0002437 | inflammatory response to antigenic stimulus | 9 | 0.001326469 |
| BP | GO:1900047 | negative regulation of hemostasis | 8 | 0.001326959 |
| BP | GO:0032958 | inositol phosphate biosynthetic process | 6 | 0.001326959 |
| BP | GO:0051883 | killing of cells in other organism involved in symbiotic interaction | 6 | 0.001326959 |
| BP | GO:1902932 | positive regulation of alcohol biosynthetic process | 6 | 0.001326959 |
| BP | GO:0061045 | negative regulation of wound healing | 10 | 0.001336046 |
| BP | GO:2000279 | negative regulation of DNA biosynthetic process | 7 | 0.001337631 |
| BP | GO:0034764 | positive regulation of transmembrane transport | 18 | 0.001384485 |
| BP | GO:0051235 | maintenance of location | 24 | 0.001405341 |
| BP | GO:0003012 | muscle system process | 31 | 0.001433357 |
| BP | GO:0007586 | digestion | 14 | 0.001433952 |
| BP | GO:0060675 | ureteric bud morphogenesis | 9 | 0.001466953 |
| BP | GO:0006809 | nitric oxide biosynthetic process | 10 | 0.001466953 |
| BP | GO:0071260 | cellular response to mechanical stimulus | 10 | 0.001466953 |
| BP | GO:0001912 | positive regulation of leukocyte mediated cytotoxicity | 8 | 0.001491987 |
| BP | GO:0003179 | heart valve morphogenesis | 8 | 0.001491987 |
| BP | GO:0048260 | positive regulation of receptor-mediated endocytosis | 8 | 0.001491987 |
| BP | GO:0055078 | sodium ion homeostasis | 8 | 0.001491987 |
| BP | GO:0051591 | response to cAMP | 11 | 0.001493959 |
| BP | GO:0060993 | kidney morphogenesis | 11 | 0.001493959 |
| BP | GO:0032674 | regulation of interleukin-5 production | 5 | 0.001505837 |
| BP | GO:0033189 | response to vitamin A | 5 | 0.001505837 |
| BP | GO:0048245 | eosinophil chemotaxis | 5 | 0.001505837 |
| BP | GO:0061081 | positive regulation of myeloid leukocyte cytokine production involved in immune response | 5 | 0.001505837 |
| BP | GO:0090184 | positive regulation of kidney development | 7 | 0.001534531 |
| BP | GO:0010739 | positive regulation of protein kinase A signaling | 4 | 0.001544062 |
| BP | GO:0051712 | positive regulation of killing of cells of other organism | 4 | 0.001544062 |
| BP | GO:2000318 | positive regulation of T-helper 17 type immune response | 4 | 0.001544062 |
| BP | GO:0055021 | regulation of cardiac muscle tissue growth | 10 | 0.001592277 |
| BP | GO:0046365 | monosaccharide catabolic process | 9 | 0.001608344 |
| BP | GO:0072171 | mesonephric tubule morphogenesis | 9 | 0.001608344 |
| BP | GO:0060537 | muscle tissue development | 28 | 0.001635593 |
| BP | GO:0032731 | positive regulation of interleukin-1 beta production | 8 | 0.001670069 |
| BP | GO:0001704 | formation of primary germ layer | 13 | 0.00167037 |
| BP | GO:0032652 | regulation of interleukin-1 production | 12 | 0.001677248 |
| BP | GO:0045861 | negative regulation of proteolysis | 26 | 0.001682196 |
| BP | GO:0002040 | sprouting angiogenesis | 17 | 0.001684108 |
| BP | GO:0019216 | regulation of lipid metabolic process | 29 | 0.001705968 |
| BP | GO:0034767 | positive regulation of ion transmembrane transport | 15 | 0.001706218 |
| BP | GO:0071375 | cellular response to peptide hormone stimulus | 24 | 0.001756271 |
| BP | GO:0032890 | regulation of organic acid transport | 9 | 0.001773154 |
| BP | GO:0043550 | regulation of lipid kinase activity | 9 | 0.001773154 |
| BP | GO:0072678 | T cell migration | 9 | 0.001773154 |
| BP | GO:1903409 | reactive oxygen species biosynthetic process | 13 | 0.001777978 |
| BP | GO:0043372 | positive regulation of CD4-positive, alpha-beta T cell differentiation | 6 | 0.001863462 |
| BP | GO:0061082 | myeloid leukocyte cytokine production | 6 | 0.001863462 |
| BP | GO:0032757 | positive regulation of interleukin-8 production | 8 | 0.001866645 |
| BP | GO:0002689 | negative regulation of leukocyte chemotaxis | 5 | 0.001878653 |
| BP | GO:0002827 | positive regulation of T-helper 1 type immune response | 5 | 0.001878653 |
| BP | GO:0032634 | interleukin-5 production | 5 | 0.001878653 |
| BP | GO:0043950 | positive regulation of cAMP-mediated signaling | 5 | 0.001878653 |
| BP | GO:0120162 | positive regulation of cold-induced thermogenesis | 11 | 0.001878653 |
| BP | GO:0002576 | platelet degranulation | 13 | 0.001893784 |
| BP | GO:1903510 | mucopolysaccharide metabolic process | 12 | 0.001925707 |
| BP | GO:0032642 | regulation of chemokine production | 9 | 0.001950951 |
| BP | GO:1902622 | regulation of neutrophil migration | 7 | 0.002006328 |
| BP | GO:0051384 | response to glucocorticoid | 14 | 0.002055853 |
| BP | GO:1904064 | positive regulation of cation transmembrane transport | 14 | 0.002055853 |
| BP | GO:0048872 | homeostasis of number of cells | 20 | 0.00206308 |
| BP | GO:0090288 | negative regulation of cellular response to growth factor stimulus | 16 | 0.00206308 |
| BP | GO:0001706 | endoderm formation | 8 | 0.002075021 |
| BP | GO:0051353 | positive regulation of oxidoreductase activity | 8 | 0.002075021 |
| BP | GO:0060350 | endochondral bone morphogenesis | 8 | 0.002075021 |
| BP | GO:0030208 | dermatan sulfate biosynthetic process | 4 | 0.002140175 |
| BP | GO:0032736 | positive regulation of interleukin-13 production | 4 | 0.002140175 |
| BP | GO:0044650 | adhesion of symbiont to host cell | 4 | 0.002140175 |
| BP | GO:0003206 | cardiac chamber morphogenesis | 13 | 0.002155361 |
| BP | GO:0002675 | positive regulation of acute inflammatory response | 6 | 0.002176594 |
| BP | GO:1902624 | positive regulation of neutrophil migration | 6 | 0.002176594 |
| BP | GO:0046209 | nitric oxide metabolic process | 10 | 0.002236724 |
| BP | GO:0007179 | transforming growth factor beta receptor signaling pathway | 17 | 0.002275053 |
| BP | GO:0090183 | regulation of kidney development | 8 | 0.002318909 |
| BP | GO:1904645 | response to amyloid-beta | 8 | 0.002318909 |
| BP | GO:0032616 | interleukin-13 production | 5 | 0.002323976 |
| BP | GO:0046641 | positive regulation of alpha-beta T cell proliferation | 5 | 0.002323976 |
| BP | GO:0019722 | calcium-mediated signaling | 18 | 0.002453201 |
| BP | GO:0032673 | regulation of interleukin-4 production | 6 | 0.002565323 |
| BP | GO:0035767 | endothelial cell chemotaxis | 6 | 0.002565323 |
| BP | GO:0045589 | regulation of regulatory T cell differentiation | 6 | 0.002565323 |
| BP | GO:0050869 | negative regulation of B cell activation | 6 | 0.002565323 |
| BP | GO:0071398 | cellular response to fatty acid | 8 | 0.002591121 |
| BP | GO:0001913 | T cell mediated cytotoxicity | 7 | 0.002596321 |
| BP | GO:0048639 | positive regulation of developmental growth | 16 | 0.002691841 |
| BP | GO:0060840 | artery development | 11 | 0.002768375 |
| BP | GO:0002224 | toll-like receptor signaling pathway | 14 | 0.002773519 |
| BP | GO:0032874 | positive regulation of stress-activated MAPK cascade | 15 | 0.002841188 |
| BP | GO:0006816 | calcium ion transport | 28 | 0.002860156 |
| BP | GO:0033005 | positive regulation of mast cell activation | 5 | 0.002860156 |
| BP | GO:0034104 | negative regulation of tissue remodeling | 5 | 0.002860156 |
| BP | GO:0045624 | positive regulation of T-helper cell differentiation | 5 | 0.002860156 |
| BP | GO:0071379 | cellular response to prostaglandin stimulus | 5 | 0.002860156 |
| BP | GO:0090189 | regulation of branching involved in ureteric bud morphogenesis | 5 | 0.002860156 |
| BP | GO:0055072 | iron ion homeostasis | 10 | 0.002865358 |
| BP | GO:2001057 | reactive nitrogen species metabolic process | 10 | 0.002865358 |
| BP | GO:0002886 | regulation of myeloid leukocyte mediated immunity | 8 | 0.002865358 |
| BP | GO:0030205 | dermatan sulfate metabolic process | 4 | 0.002878503 |
| BP | GO:0031650 | regulation of heat generation | 4 | 0.002878503 |
| BP | GO:0032725 | positive regulation of granulocyte macrophage colony-stimulating factor production | 4 | 0.002878503 |
| BP | GO:0033605 | positive regulation of catecholamine secretion | 4 | 0.002878503 |
| BP | GO:0045741 | positive regulation of epidermal growth factor-activated receptor activity | 4 | 0.002878503 |
| BP | GO:0060249 | anatomical structure homeostasis | 30 | 0.002881997 |
| BP | GO:0016202 | regulation of striated muscle tissue development | 14 | 0.002895273 |
| BP | GO:0001974 | blood vessel remodeling | 7 | 0.002895273 |
| BP | GO:0014002 | astrocyte development | 7 | 0.002895273 |
| BP | GO:0032722 | positive regulation of chemokine production | 7 | 0.002895273 |
| BP | GO:0060412 | ventricular septum morphogenesis | 7 | 0.002895273 |
| BP | GO:0002861 | regulation of inflammatory response to antigenic stimulus | 6 | 0.002931565 |
| BP | GO:0003338 | metanephros morphogenesis | 6 | 0.002931565 |
| BP | GO:0042755 | eating behavior | 6 | 0.002931565 |
| BP | GO:0046640 | regulation of alpha-beta T cell proliferation | 6 | 0.002931565 |
| BP | GO:0062013 | positive regulation of small molecule metabolic process | 14 | 0.003053663 |
| BP | GO:0072078 | nephron tubule morphogenesis | 9 | 0.003070909 |
| BP | GO:0070304 | positive regulation of stress-activated protein kinase signaling cascade | 15 | 0.003098141 |
| BP | GO:0043030 | regulation of macrophage activation | 8 | 0.003141374 |
| BP | GO:0032309 | icosanoid secretion | 7 | 0.003274889 |
| BP | GO:0035987 | endodermal cell differentiation | 7 | 0.003274889 |
| BP | GO:0014706 | striated muscle tissue development | 26 | 0.003316738 |
| BP | GO:0044070 | regulation of anion transport | 11 | 0.003383761 |
| BP | GO:0015850 | organic hydroxy compound transport | 20 | 0.003391231 |
| BP | GO:0016525 | negative regulation of angiogenesis | 16 | 0.003403084 |
| BP | GO:0042311 | vasodilation | 6 | 0.003403084 |
| BP | GO:0061384 | heart trabecula morphogenesis | 6 | 0.003403084 |
| BP | GO:2000352 | negative regulation of endothelial cell apoptotic process | 6 | 0.003403084 |
| BP | GO:1901861 | regulation of muscle tissue development | 14 | 0.00340882 |
| BP | GO:0032693 | negative regulation of interleukin-10 production | 5 | 0.00340882 |
| BP | GO:0015718 | monocarboxylic acid transport | 15 | 0.003432915 |
| BP | GO:0043393 | regulation of protein binding | 17 | 0.003458278 |
| BP | GO:0045471 | response to ethanol | 12 | 0.00348877 |
| BP | GO:0048634 | regulation of muscle organ development | 14 | 0.003608406 |
| BP | GO:0032611 | interleukin-1 beta production | 11 | 0.003608406 |
| BP | GO:0072088 | nephron epithelium morphogenesis | 9 | 0.003654352 |
| BP | GO:0051091 | positive regulation of DNA-binding transcription factor activity | 20 | 0.003654856 |
| BP | GO:0045933 | positive regulation of muscle contraction | 7 | 0.003654856 |
| BP | GO:0046638 | positive regulation of alpha-beta T cell differentiation | 7 | 0.003654856 |
| BP | GO:0018105 | peptidyl-serine phosphorylation | 22 | 0.003665738 |
| BP | GO:2000181 | negative regulation of blood vessel morphogenesis | 16 | 0.003732282 |
| BP | GO:0030207 | chondroitin sulfate catabolic process | 4 | 0.003732282 |
| BP | GO:0035810 | positive regulation of urine volume | 4 | 0.003732282 |
| BP | GO:0051709 | regulation of killing of cells of other organism | 4 | 0.003732282 |
| BP | GO:0072216 | positive regulation of metanephros development | 4 | 0.003732282 |
| BP | GO:0003170 | heart valve development | 8 | 0.003798281 |
| BP | GO:0045576 | mast cell activation | 8 | 0.003798281 |
| BP | GO:0033273 | response to vitamin | 10 | 0.003841007 |
| BP | GO:0001914 | regulation of T cell mediated cytotoxicity | 6 | 0.003875693 |
| BP | GO:0030513 | positive regulation of BMP signaling pathway | 6 | 0.003875693 |
| BP | GO:0032633 | interleukin-4 production | 6 | 0.003875693 |
| BP | GO:0045066 | regulatory T cell differentiation | 6 | 0.003875693 |
| BP | GO:0010633 | negative regulation of epithelial cell migration | 12 | 0.003926661 |
| BP | GO:0043523 | regulation of neuron apoptotic process | 17 | 0.003926661 |
| BP | GO:0060193 | positive regulation of lipase activity | 9 | 0.003926661 |
| BP | GO:0060415 | muscle tissue morphogenesis | 9 | 0.003926661 |
| BP | GO:0030324 | lung development | 15 | 0.003959185 |
| BP | GO:0051092 | positive regulation of NF-kappaB transcription factor activity | 14 | 0.003964561 |
| BP | GO:0018209 | peptidyl-serine modification | 23 | 0.003997786 |
| BP | GO:0002438 | acute inflammatory response to antigenic stimulus | 5 | 0.004022995 |
| BP | GO:0003181 | atrioventricular valve morphogenesis | 5 | 0.004022995 |
| BP | GO:0010869 | regulation of receptor biosynthetic process | 5 | 0.004022995 |
| BP | GO:0098581 | detection of external biotic stimulus | 5 | 0.004022995 |
| BP | GO:0043370 | regulation of CD4-positive, alpha-beta T cell differentiation | 7 | 0.00403247 |
| BP | GO:0030879 | mammary gland development | 13 | 0.00409541 |
| BP | GO:0019318 | hexose metabolic process | 19 | 0.004170256 |
| BP | GO:0042493 | response to drug | 26 | 0.004211307 |
| BP | GO:0045927 | positive regulation of growth | 20 | 0.004214595 |
| BP | GO:0071695 | anatomical structure maturation | 18 | 0.004234104 |
| BP | GO:0060411 | cardiac septum morphogenesis | 9 | 0.004234104 |
| BP | GO:0061333 | renal tubule morphogenesis | 9 | 0.004234104 |
| BP | GO:0072028 | nephron morphogenesis | 9 | 0.004234104 |
| BP | GO:0072006 | nephron development | 13 | 0.0043275 |
| BP | GO:0001569 | branching involved in blood vessel morphogenesis | 6 | 0.004382225 |
| BP | GO:0010543 | regulation of platelet activation | 6 | 0.004382225 |
| BP | GO:0045622 | regulation of T-helper cell differentiation | 6 | 0.004382225 |
| BP | GO:0045742 | positive regulation of epidermal growth factor receptor signaling pathway | 6 | 0.004382225 |
| BP | GO:0046633 | alpha-beta T cell proliferation | 6 | 0.004382225 |
| BP | GO:0043112 | receptor metabolic process | 16 | 0.004428596 |
| BP | GO:0031279 | regulation of cyclase activity | 7 | 0.004483931 |
| BP | GO:0043900 | regulation of multi-organism process | 9 | 0.004596524 |
| BP | GO:0060349 | bone morphogenesis | 10 | 0.004697717 |
| BP | GO:0002281 | macrophage activation involved in immune response | 4 | 0.004697717 |
| BP | GO:0002864 | regulation of acute inflammatory response to antigenic stimulus | 4 | 0.004697717 |
| BP | GO:0032645 | regulation of granulocyte macrophage colony-stimulating factor production | 4 | 0.004697717 |
| BP | GO:0035635 | entry of bacterium into host cell | 4 | 0.004697717 |
| BP | GO:0042976 | activation of Janus kinase activity | 4 | 0.004697717 |
| BP | GO:0044406 | adhesion of symbiont to host | 4 | 0.004697717 |
| BP | GO:0050651 | dermatan sulfate proteoglycan biosynthetic process | 4 | 0.004697717 |
| BP | GO:0090594 | inflammatory response to wounding | 4 | 0.004697717 |
| BP | GO:0010738 | regulation of protein kinase A signaling | 5 | 0.004716411 |
| BP | GO:0014829 | vascular associated smooth muscle contraction | 5 | 0.004716411 |
| BP | GO:0071677 | positive regulation of mononuclear cell migration | 5 | 0.004716411 |
| BP | GO:2001026 | regulation of endothelial cell chemotaxis | 5 | 0.004716411 |
| BP | GO:0030323 | respiratory tube development | 15 | 0.004743217 |
| BP | GO:0046637 | regulation of alpha-beta T cell differentiation | 8 | 0.00492547 |
| BP | GO:2000401 | regulation of lymphocyte migration | 8 | 0.00492547 |
| BP | GO:0050672 | negative regulation of lymphocyte proliferation | 9 | 0.004931849 |
| BP | GO:0002711 | positive regulation of T cell mediated immunity | 7 | 0.004931849 |
| BP | GO:0030195 | negative regulation of blood coagulation | 7 | 0.004931849 |
| BP | GO:0071715 | icosanoid transport | 7 | 0.004931849 |
| BP | GO:1901571 | fatty acid derivative transport | 7 | 0.004931849 |
| BP | GO:1903727 | positive regulation of phospholipid metabolic process | 7 | 0.004931849 |
| BP | GO:1904994 | regulation of leukocyte adhesion to vascular endothelial cell | 6 | 0.004932928 |
| BP | GO:0070167 | regulation of biomineral tissue development | 10 | 0.005001544 |
| BP | GO:0110149 | regulation of biomineralization | 10 | 0.005001544 |
| BP | GO:0031098 | stress-activated protein kinase signaling cascade | 21 | 0.00518283 |
| BP | GO:0032945 | negative regulation of mononuclear cell proliferation | 9 | 0.00533799 |
| BP | GO:0048146 | positive regulation of fibroblast proliferation | 7 | 0.005514236 |
| BP | GO:0070838 | divalent metal ion transport | 29 | 0.005532491 |
| BP | GO:0002507 | tolerance induction | 5 | 0.005532491 |
| BP | GO:0003171 | atrioventricular valve development | 5 | 0.005532491 |
| BP | GO:0021884 | forebrain neuron development | 5 | 0.005532491 |
| BP | GO:0032967 | positive regulation of collagen biosynthetic process | 5 | 0.005532491 |
| BP | GO:0042104 | positive regulation of activated T cell proliferation | 5 | 0.005532491 |
| BP | GO:0060343 | trabecula formation | 5 | 0.005532491 |
| BP | GO:1905523 | positive regulation of macrophage migration | 5 | 0.005532491 |
| BP | GO:1903531 | negative regulation of secretion by cell | 14 | 0.005578506 |
| BP | GO:0051930 | regulation of sensory perception of pain | 6 | 0.005578506 |
| BP | GO:0051954 | positive regulation of amine transport | 6 | 0.005578506 |
| BP | GO:1901186 | positive regulation of ERBB signaling pathway | 6 | 0.005578506 |
| BP | GO:2000403 | positive regulation of lymphocyte migration | 6 | 0.005578506 |
| BP | GO:0060541 | respiratory system development | 16 | 0.00574343 |
| BP | GO:0032651 | regulation of interleukin-1 beta production | 10 | 0.005743448 |
| BP | GO:0045667 | regulation of osteoblast differentiation | 12 | 0.005816141 |
| BP | GO:0045669 | positive regulation of osteoblast differentiation | 8 | 0.005816141 |
| BP | GO:0007567 | parturition | 4 | 0.005816141 |
| BP | GO:0032604 | granulocyte macrophage colony-stimulating factor production | 4 | 0.005816141 |
| BP | GO:0033033 | negative regulation of myeloid cell apoptotic process | 4 | 0.005816141 |
| BP | GO:0034374 | low-density lipoprotein particle remodeling | 4 | 0.005816141 |
| BP | GO:0045342 | MHC class II biosynthetic process | 4 | 0.005816141 |
| BP | GO:0050655 | dermatan sulfate proteoglycan metabolic process | 4 | 0.005816141 |
| BP | GO:0051147 | regulation of muscle cell differentiation | 15 | 0.005956736 |
| BP | GO:0051339 | regulation of lyase activity | 7 | 0.006012908 |
| BP | GO:2001238 | positive regulation of extrinsic apoptotic signaling pathway | 7 | 0.006012908 |
| BP | GO:0051098 | regulation of binding | 24 | 0.006038067 |
| BP | GO:0071496 | cellular response to external stimulus | 22 | 0.006225324 |
| BP | GO:0010737 | protein kinase A signaling | 6 | 0.006281007 |
| BP | GO:0042092 | type 2 immune response | 6 | 0.006281007 |
| BP | GO:0051931 | regulation of sensory perception | 6 | 0.006281007 |
| BP | GO:0003148 | outflow tract septum morphogenesis | 5 | 0.006409619 |
| BP | GO:0010714 | positive regulation of collagen metabolic process | 5 | 0.006409619 |
| BP | GO:0048143 | astrocyte activation | 5 | 0.006409619 |
| BP | GO:1901623 | regulation of lymphocyte chemotaxis | 5 | 0.006409619 |
| BP | GO:0051090 | regulation of DNA-binding transcription factor activity | 28 | 0.006413514 |
| BP | GO:0051403 | stress-activated MAPK cascade | 20 | 0.006434838 |
| BP | GO:0010717 | regulation of epithelial to mesenchymal transition | 10 | 0.006518043 |
| BP | GO:0043255 | regulation of carbohydrate biosynthetic process | 10 | 0.006518043 |
| BP | GO:0090277 | positive regulation of peptide hormone secretion | 10 | 0.006518043 |
| BP | GO:0046620 | regulation of organ growth | 11 | 0.00658319 |
| BP | GO:0072511 | divalent inorganic cation transport | 29 | 0.00658319 |
| BP | GO:0048145 | regulation of fibroblast proliferation | 9 | 0.00658319 |
| BP | GO:0048644 | muscle organ morphogenesis | 9 | 0.00658319 |
| BP | GO:0061756 | leukocyte adhesion to vascular endothelial cell | 7 | 0.006591792 |
| BP | GO:0021700 | developmental maturation | 20 | 0.006638807 |
| BP | GO:2000241 | regulation of reproductive process | 14 | 0.006705999 |
| BP | GO:0043534 | blood vessel endothelial cell migration | 15 | 0.006788083 |
| BP | GO:0001837 | epithelial to mesenchymal transition | 13 | 0.006810563 |
| BP | GO:0002637 | regulation of immunoglobulin production | 8 | 0.006810563 |
| BP | GO:0045428 | regulation of nitric oxide biosynthetic process | 8 | 0.006810563 |
| BP | GO:0072577 | endothelial cell apoptotic process | 8 | 0.006810563 |
| BP | GO:0051341 | regulation of oxidoreductase activity | 11 | 0.006974104 |
| BP | GO:0030501 | positive regulation of bone mineralization | 6 | 0.007005541 |
| BP | GO:0030307 | positive regulation of cell growth | 14 | 0.007022234 |
| BP | GO:0015844 | monoamine transport | 9 | 0.007050298 |
| BP | GO:0045913 | positive regulation of carbohydrate metabolic process | 9 | 0.007050298 |
| BP | GO:0048144 | fibroblast proliferation | 9 | 0.007050298 |
| BP | GO:0010715 | regulation of extracellular matrix disassembly | 4 | 0.00711475 |
| BP | GO:0030889 | negative regulation of B cell proliferation | 4 | 0.00711475 |
| BP | GO:0034599 | cellular response to oxidative stress | 21 | 0.007204439 |
| BP | GO:0010524 | positive regulation of calcium ion transport into cytosol | 7 | 0.007216751 |
| BP | GO:0032623 | interleukin-2 production | 7 | 0.007216751 |
| BP | GO:0006109 | regulation of carbohydrate metabolic process | 16 | 0.007310764 |
| BP | GO:0032800 | receptor biosynthetic process | 5 | 0.007341662 |
| BP | GO:1990776 | response to angiotensin | 5 | 0.007341662 |
| BP | GO:0008543 | fibroblast growth factor receptor signaling pathway | 11 | 0.007351937 |
| BP | GO:0097191 | extrinsic apoptotic signaling pathway | 17 | 0.007433772 |
| BP | GO:0007618 | mating | 6 | 0.007825359 |
| BP | GO:0030890 | positive regulation of B cell proliferation | 6 | 0.007825359 |
| BP | GO:0042596 | fear response | 6 | 0.007825359 |
| BP | GO:0048286 | lung alveolus development | 6 | 0.007825359 |
| BP | GO:0022600 | digestive system process | 10 | 0.007862151 |
| BP | GO:0050433 | regulation of catecholamine secretion | 7 | 0.007931528 |
| BP | GO:0050819 | negative regulation of coagulation | 7 | 0.007931528 |
| BP | GO:0030183 | B cell differentiation | 12 | 0.007957057 |
| BP | GO:0003205 | cardiac chamber development | 14 | 0.00805879 |
| BP | GO:0002717 | positive regulation of natural killer cell mediated immunity | 5 | 0.008493194 |
| BP | GO:0019835 | cytolysis | 5 | 0.008493194 |
| BP | GO:0043032 | positive regulation of macrophage activation | 5 | 0.008493194 |
| BP | GO:0060351 | cartilage development involved in endochondral bone morphogenesis | 5 | 0.008493194 |
| BP | GO:0035313 | wound healing, spreading of epidermal cells | 4 | 0.008693481 |
| BP | GO:0060457 | negative regulation of digestive system process | 4 | 0.008693481 |
| BP | GO:0051781 | positive regulation of cell division | 9 | 0.008741032 |
| BP | GO:0010770 | positive regulation of cell morphogenesis involved in differentiation | 13 | 0.008742153 |
| BP | GO:1901136 | carbohydrate derivative catabolic process | 15 | 0.008816507 |
| BP | GO:0005996 | monosaccharide metabolic process | 20 | 0.009084084 |
| BP | GO:0071897 | DNA biosynthetic process | 15 | 0.009237548 |
| BP | GO:0003015 | heart process | 20 | 0.009420244 |
| BP | GO:0051651 | maintenance of location in cell | 16 | 0.009422058 |
| BP | GO:0008016 | regulation of heart contraction | 18 | 0.009422058 |
| BP | GO:0050432 | catecholamine secretion | 7 | 0.009579544 |
| BP | GO:0007176 | regulation of epidermal growth factor-activated receptor activity | 5 | 0.009785365 |
| BP | GO:0002369 | T cell cytokine production | 6 | 0.009785365 |
| BP | GO:0045823 | positive regulation of heart contraction | 6 | 0.009785365 |
| BP | GO:0003007 | heart morphogenesis | 18 | 0.0101811 |
| BP | GO:0051145 | smooth muscle cell differentiation | 8 | 0.010185936 |
| BP | GO:0070227 | lymphocyte apoptotic process | 8 | 0.010185936 |
| BP | GO:0022612 | gland morphogenesis | 11 | 0.010408704 |
| BP | GO:0002043 | blood vessel endothelial cell proliferation involved in sprouting angiogenesis | 7 | 0.010408704 |
| BP | GO:0007566 | embryo implantation | 7 | 0.010408704 |
| BP | GO:0001991 | regulation of systemic arterial blood pressure by circulatory renin-angiotensin | 4 | 0.010408704 |
| BP | GO:0010744 | positive regulation of macrophage derived foam cell differentiation | 4 | 0.010408704 |
| BP | GO:0032930 | positive regulation of superoxide anion generation | 4 | 0.010408704 |
| BP | GO:0034393 | positive regulation of smooth muscle cell apoptotic process | 4 | 0.010408704 |
| BP | GO:0035988 | chondrocyte proliferation | 4 | 0.010408704 |
| BP | GO:0045063 | T-helper 1 cell differentiation | 4 | 0.010408704 |
| BP | GO:0045780 | positive regulation of bone resorption | 4 | 0.010408704 |
| BP | GO:0046852 | positive regulation of bone remodeling | 4 | 0.010408704 |
| BP | GO:0090026 | positive regulation of monocyte chemotaxis | 4 | 0.010408704 |
| BP | GO:0098543 | detection of other organism | 4 | 0.010408704 |
| BP | GO:1900747 | negative regulation of vascular endothelial growth factor signaling pathway | 4 | 0.010408704 |
| BP | GO:0021537 | telencephalon development | 18 | 0.010426045 |
| BP | GO:0010596 | negative regulation of endothelial cell migration | 10 | 0.010700995 |
| BP | GO:0033003 | regulation of mast cell activation | 6 | 0.010770386 |
| BP | GO:1903053 | regulation of extracellular matrix organization | 6 | 0.010770386 |
| BP | GO:1905521 | regulation of macrophage migration | 6 | 0.010770386 |
| BP | GO:1903036 | positive regulation of response to wounding | 8 | 0.010868577 |
| BP | GO:0001952 | regulation of cell-matrix adhesion | 11 | 0.010928711 |
| BP | GO:0002323 | natural killer cell activation involved in immune response | 5 | 0.011026049 |
| BP | GO:0002828 | regulation of type 2 immune response | 5 | 0.011026049 |
| BP | GO:0072538 | T-helper 17 type immune response | 5 | 0.011026049 |
| BP | GO:2001236 | regulation of extrinsic apoptotic signaling pathway | 13 | 0.011027857 |
| BP | GO:0060043 | regulation of cardiac muscle cell proliferation | 7 | 0.011293719 |
| BP | GO:0032526 | response to retinoic acid | 10 | 0.011319543 |
| BP | GO:0001656 | metanephros development | 9 | 0.011353058 |
| BP | GO:0097306 | cellular response to alcohol | 9 | 0.011353058 |
| BP | GO:0042445 | hormone metabolic process | 16 | 0.011358994 |
| BP | GO:0030858 | positive regulation of epithelial cell differentiation | 8 | 0.011646414 |
| BP | GO:0045685 | regulation of glial cell differentiation | 8 | 0.011646414 |
| BP | GO:0048844 | artery morphogenesis | 8 | 0.011646414 |
| BP | GO:0001660 | fever generation | 3 | 0.011646414 |
| BP | GO:0032815 | negative regulation of natural killer cell activation | 3 | 0.011646414 |
| BP | GO:0032817 | regulation of natural killer cell proliferation | 3 | 0.011646414 |
| BP | GO:0043084 | penile erection | 3 | 0.011646414 |
| BP | GO:0043380 | regulation of memory T cell differentiation | 3 | 0.011646414 |
| BP | GO:0045625 | regulation of T-helper 1 cell differentiation | 3 | 0.011646414 |
| BP | GO:0045713 | low-density lipoprotein particle receptor biosynthetic process | 3 | 0.011646414 |
| BP | GO:0070099 | regulation of chemokine-mediated signaling pathway | 3 | 0.011646414 |
| BP | GO:0062012 | regulation of small molecule metabolic process | 27 | 0.011655068 |
| BP | GO:0042692 | muscle cell differentiation | 24 | 0.01170535 |
| BP | GO:0052548 | regulation of endopeptidase activity | 26 | 0.01170535 |
| BP | GO:0002715 | regulation of natural killer cell mediated immunity | 6 | 0.01170535 |
| BP | GO:0003197 | endocardial cushion development | 6 | 0.01170535 |
| BP | GO:0032924 | activin receptor signaling pathway | 6 | 0.01170535 |
| BP | GO:0043114 | regulation of vascular permeability | 6 | 0.01170535 |
| BP | GO:0045429 | positive regulation of nitric oxide biosynthetic process | 6 | 0.01170535 |
| BP | GO:0060443 | mammary gland morphogenesis | 6 | 0.01170535 |
| BP | GO:2001222 | regulation of neuron migration | 6 | 0.01170535 |
| BP | GO:0035904 | aorta development | 7 | 0.012108445 |
| BP | GO:0003085 | negative regulation of systemic arterial blood pressure | 4 | 0.012132721 |
| BP | GO:0007194 | negative regulation of adenylate cyclase activity | 4 | 0.012132721 |
| BP | GO:0007620 | copulation | 4 | 0.012132721 |
| BP | GO:0032816 | positive regulation of natural killer cell activation | 4 | 0.012132721 |
| BP | GO:0034138 | toll-like receptor 3 signaling pathway | 4 | 0.012132721 |
| BP | GO:1905939 | regulation of gonad development | 4 | 0.012132721 |
| BP | GO:0003176 | aortic valve development | 5 | 0.01227209 |
| BP | GO:0045761 | regulation of adenylate cyclase activity | 5 | 0.01227209 |
| BP | GO:0046328 | regulation of JNK cascade | 14 | 0.01230321 |
| BP | GO:0003281 | ventricular septum development | 8 | 0.012306363 |
| BP | GO:0042542 | response to hydrogen peroxide | 12 | 0.012308175 |
| BP | GO:0072009 | nephron epithelium development | 10 | 0.012455541 |
| BP | GO:0030510 | regulation of BMP signaling pathway | 9 | 0.012641815 |
| BP | GO:0032677 | regulation of interleukin-8 production | 9 | 0.012641815 |
| BP | GO:0051702 | interaction with symbiont | 9 | 0.012641815 |
| BP | GO:0072080 | nephron tubule development | 9 | 0.012641815 |
| BP | GO:0010863 | positive regulation of phospholipase C activity | 6 | 0.012847156 |
| BP | GO:1904407 | positive regulation of nitric oxide metabolic process | 6 | 0.012847156 |
| BP | GO:0046888 | negative regulation of hormone secretion | 7 | 0.013035518 |
| BP | GO:0090303 | positive regulation of wound healing | 7 | 0.013035518 |
| BP | GO:0060047 | heart contraction | 19 | 0.013095506 |
| BP | GO:0010675 | regulation of cellular carbohydrate metabolic process | 12 | 0.013581164 |
| BP | GO:0021988 | olfactory lobe development | 5 | 0.013828513 |
| BP | GO:0033028 | myeloid cell apoptotic process | 5 | 0.013828513 |
| BP | GO:0044319 | wound healing, spreading of cells | 5 | 0.013828513 |
| BP | GO:0090505 | epiboly involved in wound healing | 5 | 0.013828513 |
| BP | GO:0055025 | positive regulation of cardiac muscle tissue development | 7 | 0.014143718 |
| BP | GO:2000351 | regulation of endothelial cell apoptotic process | 7 | 0.014143718 |
| BP | GO:0030225 | macrophage differentiation | 6 | 0.014143718 |
| BP | GO:0043303 | mast cell degranulation | 6 | 0.014143718 |
| BP | GO:0051057 | positive regulation of small GTPase mediated signal transduction | 8 | 0.014143718 |
| BP | GO:0014074 | response to purine-containing compound | 12 | 0.014143718 |
| BP | GO:0002029 | desensitization of G protein-coupled receptor signaling pathway | 4 | 0.014143718 |
| BP | GO:0003177 | pulmonary valve development | 4 | 0.014143718 |
| BP | GO:0014821 | phasic smooth muscle contraction | 4 | 0.014143718 |
| BP | GO:0022401 | negative adaptation of signaling pathway | 4 | 0.014143718 |
| BP | GO:0090280 | positive regulation of calcium ion import | 4 | 0.014143718 |
| BP | GO:0140131 | positive regulation of lymphocyte chemotaxis | 4 | 0.014143718 |
| BP | GO:1902548 | negative regulation of cellular response to vascular endothelial growth factor stimulus | 4 | 0.014143718 |
| BP | GO:0007589 | body fluid secretion | 9 | 0.014201147 |
| BP | GO:0061326 | renal tubule development | 9 | 0.014201147 |
| BP | GO:1903725 | regulation of phospholipid metabolic process | 9 | 0.014201147 |
| BP | GO:0051222 | positive regulation of protein transport | 22 | 0.014216878 |
| BP | GO:0048705 | skeletal system morphogenesis | 16 | 0.014395123 |
| BP | GO:0003231 | cardiac ventricle development | 11 | 0.01450182 |
| BP | GO:0001787 | natural killer cell proliferation | 3 | 0.014618392 |
| BP | GO:0003093 | regulation of glomerular filtration | 3 | 0.014618392 |
| BP | GO:0014820 | tonic smooth muscle contraction | 3 | 0.014618392 |
| BP | GO:0031652 | positive regulation of heat generation | 3 | 0.014618392 |
| BP | GO:0032276 | regulation of gonadotropin secretion | 3 | 0.014618392 |
| BP | GO:0032754 | positive regulation of interleukin-5 production | 3 | 0.014618392 |
| BP | GO:0033197 | response to vitamin E | 3 | 0.014618392 |
| BP | GO:0033860 | regulation of NAD(P)H oxidase activity | 3 | 0.014618392 |
| BP | GO:0035747 | natural killer cell chemotaxis | 3 | 0.014618392 |
| BP | GO:0040015 | negative regulation of multicellular organism growth | 3 | 0.014618392 |
| BP | GO:0043379 | memory T cell differentiation | 3 | 0.014618392 |
| BP | GO:0043589 | skin morphogenesis | 3 | 0.014618392 |
| BP | GO:0090715 | immunological memory formation process | 3 | 0.014618392 |
| BP | GO:0098917 | retrograde trans-synaptic signaling | 3 | 0.014618392 |
| BP | GO:0006826 | iron ion transport | 8 | 0.014865305 |
| BP | GO:0007492 | endoderm development | 8 | 0.014865305 |
| BP | GO:0055008 | cardiac muscle tissue morphogenesis | 7 | 0.015004109 |
| BP | GO:0007202 | activation of phospholipase C activity | 5 | 0.015152972 |
| BP | GO:0010922 | positive regulation of phosphatase activity | 5 | 0.015152972 |
| BP | GO:0051482 | positive regulation of cytosolic calcium ion concentration involved in phospholipase C-activating G protein-coupled signaling pathway | 5 | 0.015152972 |
| BP | GO:0090504 | epiboly | 5 | 0.015152972 |
| BP | GO:0002279 | mast cell activation involved in immune response | 6 | 0.015152972 |
| BP | GO:0070169 | positive regulation of biomineral tissue development | 6 | 0.015152972 |
| BP | GO:0110151 | positive regulation of biomineralization | 6 | 0.015152972 |
| BP | GO:1900274 | regulation of phospholipase C activity | 6 | 0.015152972 |
| BP | GO:0003279 | cardiac septum development | 10 | 0.01517916 |
| BP | GO:0030856 | regulation of epithelial cell differentiation | 13 | 0.015822111 |
| BP | GO:1901655 | cellular response to ketone | 9 | 0.015822111 |
| BP | GO:1901224 | positive regulation of NIK/NF-kappaB signaling | 8 | 0.015851007 |
| BP | GO:0022408 | negative regulation of cell-cell adhesion | 14 | 0.016048494 |
| BP | GO:0031623 | receptor internalization | 10 | 0.016061201 |
| BP | GO:0023058 | adaptation of signaling pathway | 4 | 0.016198381 |
| BP | GO:0032700 | negative regulation of interleukin-17 production | 4 | 0.016198381 |
| BP | GO:0032928 | regulation of superoxide anion generation | 4 | 0.016198381 |
| BP | GO:0044342 | type B pancreatic cell proliferation | 4 | 0.016198381 |
| BP | GO:2000316 | regulation of T-helper 17 type immune response | 4 | 0.016198381 |
| BP | GO:0046683 | response to organophosphorus | 11 | 0.016587804 |
| BP | GO:0002448 | mast cell mediated immunity | 6 | 0.016587804 |
| BP | GO:0032956 | regulation of actin cytoskeleton organization | 22 | 0.016612085 |
| BP | GO:0001960 | negative regulation of cytokine-mediated signaling pathway | 8 | 0.016877956 |
| BP | GO:0019915 | lipid storage | 8 | 0.016877956 |
| BP | GO:0046916 | cellular transition metal ion homeostasis | 10 | 0.016877956 |
| BP | GO:0003203 | endocardial cushion morphogenesis | 5 | 0.016877956 |
| BP | GO:0034390 | smooth muscle cell apoptotic process | 5 | 0.016877956 |
| BP | GO:0034391 | regulation of smooth muscle cell apoptotic process | 5 | 0.016877956 |
| BP | GO:0009743 | response to carbohydrate | 16 | 0.016924311 |
| BP | GO:0033209 | tumor necrosis factor-mediated signaling pathway | 13 | 0.0171027 |
| BP | GO:0007254 | JNK cascade | 15 | 0.017172916 |
| BP | GO:0090276 | regulation of peptide hormone secretion | 15 | 0.017172916 |
| BP | GO:0006940 | regulation of smooth muscle contraction | 7 | 0.017297769 |
| BP | GO:0015837 | amine transport | 9 | 0.017692863 |
| BP | GO:0010469 | regulation of signaling receptor activity | 13 | 0.017848948 |
| BP | GO:0008038 | neuron recognition | 6 | 0.018063128 |
| BP | GO:2000725 | regulation of cardiac muscle cell differentiation | 6 | 0.018063128 |
| BP | GO:0045444 | fat cell differentiation | 16 | 0.018160838 |
| BP | GO:0001867 | complement activation, lectin pathway | 3 | 0.018160838 |
| BP | GO:0002863 | positive regulation of inflammatory response to antigenic stimulus | 3 | 0.018160838 |
| BP | GO:0030157 | pancreatic juice secretion | 3 | 0.018160838 |
| BP | GO:0051549 | positive regulation of keratinocyte migration | 3 | 0.018160838 |
| BP | GO:0060453 | regulation of gastric acid secretion | 3 | 0.018160838 |
| BP | GO:0070278 | extracellular matrix constituent secretion | 3 | 0.018160838 |
| BP | GO:0072540 | T-helper 17 cell lineage commitment | 3 | 0.018160838 |
| BP | GO:1902287 | semaphorin-plexin signaling pathway involved in axon guidance | 3 | 0.018160838 |
| BP | GO:1905941 | positive regulation of gonad development | 3 | 0.018160838 |
| BP | GO:2001223 | negative regulation of neuron migration | 3 | 0.018160838 |
| BP | GO:0002228 | natural killer cell mediated immunity | 7 | 0.018447961 |
| BP | GO:0007585 | respiratory gaseous exchange by respiratory system | 7 | 0.018447961 |
| BP | GO:0034394 | protein localization to cell surface | 7 | 0.018447961 |
| BP | GO:0010226 | response to lithium ion | 4 | 0.018447961 |
| BP | GO:0031280 | negative regulation of cyclase activity | 4 | 0.018447961 |
| BP | GO:0032703 | negative regulation of interleukin-2 production | 4 | 0.018447961 |
| BP | GO:0035743 | CD4-positive, alpha-beta T cell cytokine production | 4 | 0.018447961 |
| BP | GO:0051043 | regulation of membrane protein ectodomain proteolysis | 4 | 0.018447961 |
| BP | GO:0072215 | regulation of metanephros development | 4 | 0.018447961 |
| BP | GO:1900017 | positive regulation of cytokine production involved in inflammatory response | 4 | 0.018447961 |
| BP | GO:0010092 | specification of animal organ identity | 5 | 0.018554197 |
| BP | GO:1903038 | negative regulation of leukocyte cell-cell adhesion | 11 | 0.018850909 |
| BP | GO:0001570 | vasculogenesis | 8 | 0.018883896 |
| BP | GO:0014855 | striated muscle cell proliferation | 8 | 0.018883896 |
| BP | GO:0048041 | focal adhesion assembly | 8 | 0.018883896 |
| BP | GO:0099177 | regulation of trans-synaptic signaling | 26 | 0.018914953 |
| BP | GO:0032872 | regulation of stress-activated MAPK cascade | 16 | 0.019207225 |
| BP | GO:0010038 | response to metal ion | 22 | 0.019264166 |
| BP | GO:0038084 | vascular endothelial growth factor signaling pathway | 6 | 0.019386726 |
| BP | GO:0051153 | regulation of striated muscle cell differentiation | 10 | 0.019397234 |
| BP | GO:0010522 | regulation of calcium ion transport into cytosol | 9 | 0.019496503 |
| BP | GO:0032637 | interleukin-8 production | 9 | 0.019496503 |
| BP | GO:0050890 | cognition | 19 | 0.020149586 |
| BP | GO:0060402 | calcium ion transport into cytosol | 12 | 0.02046932 |
| BP | GO:2001233 | regulation of apoptotic signaling pathway | 24 | 0.020535258 |
| BP | GO:0007611 | learning or memory | 17 | 0.020547306 |
| BP | GO:0030212 | hyaluronan metabolic process | 5 | 0.020547306 |
| BP | GO:0090322 | regulation of superoxide metabolic process | 5 | 0.020547306 |
| BP | GO:1905314 | semi-lunar valve development | 5 | 0.020547306 |
| BP | GO:0060401 | cytosolic calcium ion transport | 13 | 0.020673027 |
| BP | GO:0010883 | regulation of lipid storage | 6 | 0.021044418 |
| BP | GO:0032330 | regulation of chondrocyte differentiation | 6 | 0.021044418 |
| BP | GO:0060421 | positive regulation of heart growth | 6 | 0.021044418 |
| BP | GO:0001759 | organ induction | 4 | 0.021044418 |
| BP | GO:0033622 | integrin activation | 4 | 0.021044418 |
| BP | GO:0044321 | response to leptin | 4 | 0.021044418 |
| BP | GO:0045954 | positive regulation of natural killer cell mediated cytotoxicity | 4 | 0.021044418 |
| BP | GO:1903429 | regulation of cell maturation | 4 | 0.021044418 |
| BP | GO:0010812 | negative regulation of cell-substrate adhesion | 7 | 0.021062009 |
| BP | GO:0040014 | regulation of multicellular organism growth | 7 | 0.021062009 |
| BP | GO:0070302 | regulation of stress-activated protein kinase signaling cascade | 16 | 0.021119461 |
| BP | GO:1904951 | positive regulation of establishment of protein localization | 22 | 0.021302645 |
| BP | GO:1901890 | positive regulation of cell junction assembly | 9 | 0.021692273 |
| BP | GO:0042326 | negative regulation of phosphorylation | 27 | 0.021731637 |
| BP | GO:1904062 | regulation of cation transmembrane transport | 21 | 0.021860105 |
| BP | GO:0016052 | carbohydrate catabolic process | 14 | 0.021860105 |
| BP | GO:1901888 | regulation of cell junction assembly | 14 | 0.021860105 |
| BP | GO:0002430 | complement receptor mediated signaling pathway | 3 | 0.021860105 |
| BP | GO:0008228 | opsonization | 3 | 0.021860105 |
| BP | GO:0010745 | negative regulation of macrophage derived foam cell differentiation | 3 | 0.021860105 |
| BP | GO:0016264 | gap junction assembly | 3 | 0.021860105 |
| BP | GO:0031953 | negative regulation of protein autophosphorylation | 3 | 0.021860105 |
| BP | GO:0060100 | positive regulation of phagocytosis, engulfment | 3 | 0.021860105 |
| BP | GO:0060340 | positive regulation of type I interferon-mediated signaling pathway | 3 | 0.021860105 |
| BP | GO:0061469 | regulation of type B pancreatic cell proliferation | 3 | 0.021860105 |
| BP | GO:1900272 | negative regulation of long-term synaptic potentiation | 3 | 0.021860105 |
| BP | GO:1902285 | semaphorin-plexin signaling pathway involved in neuron projection guidance | 3 | 0.021860105 |
| BP | GO:1903236 | regulation of leukocyte tethering or rolling | 3 | 0.021860105 |
| BP | GO:1904995 | negative regulation of leukocyte adhesion to vascular endothelial cell | 3 | 0.021860105 |
| BP | GO:1905155 | positive regulation of membrane invagination | 3 | 0.021860105 |
| BP | GO:2000194 | regulation of female gonad development | 3 | 0.021860105 |
| BP | GO:0010811 | positive regulation of cell-substrate adhesion | 10 | 0.022173463 |
| BP | GO:0001889 | liver development | 11 | 0.02222205 |
| BP | GO:0060038 | cardiac muscle cell proliferation | 7 | 0.02222205 |
| BP | GO:0003298 | physiological muscle hypertrophy | 5 | 0.02222205 |
| BP | GO:0003301 | physiological cardiac muscle hypertrophy | 5 | 0.02222205 |
| BP | GO:0010661 | positive regulation of muscle cell apoptotic process | 5 | 0.02222205 |
| BP | GO:0030224 | monocyte differentiation | 5 | 0.02222205 |
| BP | GO:0061049 | cell growth involved in cardiac muscle cell development | 5 | 0.02222205 |
| BP | GO:0071276 | cellular response to cadmium ion | 5 | 0.02222205 |
| BP | GO:1903131 | mononuclear cell differentiation | 5 | 0.02222205 |
| BP | GO:0043949 | regulation of cAMP-mediated signaling | 6 | 0.022335013 |
| BP | GO:0002221 | pattern recognition receptor signaling pathway | 14 | 0.022555306 |
| BP | GO:0048863 | stem cell differentiation | 17 | 0.022855094 |
| BP | GO:0007249 | I-kappaB kinase/NF-kappaB signaling | 18 | 0.023287899 |
| BP | GO:0043302 | positive regulation of leukocyte degranulation | 4 | 0.023583134 |
| BP | GO:1904385 | cellular response to angiotensin | 4 | 0.023583134 |
| BP | GO:0030522 | intracellular receptor signaling pathway | 17 | 0.023583134 |
| BP | GO:0060761 | negative regulation of response to cytokine stimulus | 8 | 0.023583134 |
| BP | GO:0031532 | actin cytoskeleton reorganization | 9 | 0.023742778 |
| BP | GO:0002698 | negative regulation of immune effector process | 10 | 0.024347657 |
| BP | GO:0001662 | behavioral fear response | 5 | 0.024518358 |
| BP | GO:0010837 | regulation of keratinocyte proliferation | 5 | 0.024518358 |
| BP | GO:2000249 | regulation of actin cytoskeleton reorganization | 5 | 0.024518358 |
| BP | GO:0001933 | negative regulation of protein phosphorylation | 25 | 0.024518358 |
| BP | GO:0061008 | hepaticobiliary system development | 11 | 0.025346036 |
| BP | GO:0014823 | response to activity | 7 | 0.025465693 |
| BP | GO:0046579 | positive regulation of Ras protein signal transduction | 7 | 0.025465693 |
| BP | GO:0043535 | regulation of blood vessel endothelial cell migration | 12 | 0.025700654 |
| BP | GO:0045620 | negative regulation of lymphocyte differentiation | 6 | 0.02623975 |
| BP | GO:1903426 | regulation of reactive oxygen species biosynthetic process | 9 | 0.02623975 |
| BP | GO:0003413 | chondrocyte differentiation involved in endochondral bone morphogenesis | 3 | 0.02623975 |
| BP | GO:0010838 | positive regulation of keratinocyte proliferation | 3 | 0.02623975 |
| BP | GO:0030213 | hyaluronan biosynthetic process | 3 | 0.02623975 |
| BP | GO:0035437 | maintenance of protein localization in endoplasmic reticulum | 3 | 0.02623975 |
| BP | GO:0036295 | cellular response to increased oxygen levels | 3 | 0.02623975 |
| BP | GO:0038166 | angiotensin-activated signaling pathway | 3 | 0.02623975 |
| BP | GO:0045472 | response to ether | 3 | 0.02623975 |
| BP | GO:0050872 | white fat cell differentiation | 3 | 0.02623975 |
| BP | GO:0051547 | regulation of keratinocyte migration | 3 | 0.02623975 |
| BP | GO:0071285 | cellular response to lithium ion | 3 | 0.02623975 |
| BP | GO:0090713 | immunological memory process | 3 | 0.02623975 |
| BP | GO:2001257 | regulation of cation channel activity | 13 | 0.026427218 |
| BP | GO:0030206 | chondroitin sulfate biosynthetic process | 4 | 0.026504755 |
| BP | GO:0035902 | response to immobilization stress | 4 | 0.026504755 |
| BP | GO:0051350 | negative regulation of lyase activity | 4 | 0.026504755 |
| BP | GO:0072202 | cell differentiation involved in metanephros development | 4 | 0.026504755 |
| BP | GO:1904754 | positive regulation of vascular associated smooth muscle cell migration | 4 | 0.026504755 |
| BP | GO:0002209 | behavioral defense response | 5 | 0.02662416 |
| BP | GO:0002714 | positive regulation of B cell mediated immunity | 5 | 0.02662416 |
| BP | GO:0002891 | positive regulation of immunoglobulin mediated immune response | 5 | 0.02662416 |
| BP | GO:0050691 | regulation of defense response to virus by host | 5 | 0.02662416 |
| BP | GO:0051281 | positive regulation of release of sequestered calcium ion into cytosol | 5 | 0.02662416 |
| BP | GO:0072210 | metanephric nephron development | 5 | 0.02662416 |
| BP | GO:0043647 | inositol phosphate metabolic process | 7 | 0.026799489 |
| BP | GO:0051937 | catecholamine transport | 7 | 0.026799489 |
| BP | GO:0006029 | proteoglycan metabolic process | 8 | 0.027794423 |
| BP | GO:0070228 | regulation of lymphocyte apoptotic process | 6 | 0.027958246 |
| BP | GO:0032970 | regulation of actin filament-based process | 23 | 0.028539828 |
| BP | GO:0051155 | positive regulation of striated muscle cell differentiation | 7 | 0.028707941 |
| BP | GO:0021761 | limbic system development | 9 | 0.028959309 |
| BP | GO:0014013 | regulation of gliogenesis | 10 | 0.029069365 |
| BP | GO:0044262 | cellular carbohydrate metabolic process | 18 | 0.029172738 |
| BP | GO:0033574 | response to testosterone | 5 | 0.029217414 |
| BP | GO:0042269 | regulation of natural killer cell mediated cytotoxicity | 5 | 0.029217414 |
| BP | GO:2000826 | regulation of heart morphogenesis | 5 | 0.029217414 |
| BP | GO:0051952 | regulation of amine transport | 8 | 0.02933458 |
| BP | GO:0060333 | interferon-gamma-mediated signaling pathway | 8 | 0.02933458 |
| BP | GO:0043123 | positive regulation of I-kappaB kinase/NF-kappaB signaling | 13 | 0.02933458 |
| BP | GO:0031644 | regulation of nervous system process | 11 | 0.029632663 |
| BP | GO:0051495 | positive regulation of cytoskeleton organization | 15 | 0.029632663 |
| BP | GO:0002026 | regulation of the force of heart contraction | 4 | 0.029681438 |
| BP | GO:0048520 | positive regulation of behavior | 4 | 0.029681438 |
| BP | GO:0072539 | T-helper 17 cell differentiation | 4 | 0.029681438 |
| BP | GO:0002090 | regulation of receptor internalization | 6 | 0.029879977 |
| BP | GO:0045840 | positive regulation of mitotic nuclear division | 6 | 0.029879977 |
| BP | GO:0048016 | inositol phosphate-mediated signaling | 6 | 0.029879977 |
| BP | GO:0060760 | positive regulation of response to cytokine stimulus | 6 | 0.029879977 |
| BP | GO:0061005 | cell differentiation involved in kidney development | 6 | 0.029879977 |
| BP | GO:1904659 | glucose transmembrane transport | 9 | 0.030170375 |
| BP | GO:0006367 | transcription initiation from RNA polymerase II promoter | 13 | 0.030274138 |
| BP | GO:0034121 | regulation of toll-like receptor signaling pathway | 7 | 0.030281676 |
| BP | GO:0050804 | modulation of chemical synaptic transmission | 25 | 0.030311245 |
| BP | GO:0030100 | regulation of endocytosis | 14 | 0.030505429 |
| BP | GO:0001946 | lymphangiogenesis | 3 | 0.030614138 |
| BP | GO:0002467 | germinal center formation | 3 | 0.030614138 |
| BP | GO:0010455 | positive regulation of cell fate commitment | 3 | 0.030614138 |
| BP | GO:0020027 | hemoglobin metabolic process | 3 | 0.030614138 |
| BP | GO:0032274 | gonadotropin secretion | 3 | 0.030614138 |
| BP | GO:0034349 | glial cell apoptotic process | 3 | 0.030614138 |
| BP | GO:0044849 | estrous cycle | 3 | 0.030614138 |
| BP | GO:0045346 | regulation of MHC class II biosynthetic process | 3 | 0.030614138 |
| BP | GO:0060099 | regulation of phagocytosis, engulfment | 3 | 0.030614138 |
| BP | GO:0060347 | heart trabecula formation | 3 | 0.030614138 |
| BP | GO:0060841 | venous blood vessel development | 3 | 0.030614138 |
| BP | GO:0072567 | chemokine (C-X-C motif) ligand 2 production | 3 | 0.030614138 |
| BP | GO:2000341 | regulation of chemokine (C-X-C motif) ligand 2 production | 3 | 0.030614138 |
| BP | GO:2001028 | positive regulation of endothelial cell chemotaxis | 3 | 0.030614138 |
| BP | GO:0051149 | positive regulation of muscle cell differentiation | 9 | 0.031329362 |
| BP | GO:0006509 | membrane protein ectodomain proteolysis | 5 | 0.031329362 |
| BP | GO:0044058 | regulation of digestive system process | 5 | 0.031329362 |
| BP | GO:0046006 | regulation of activated T cell proliferation | 5 | 0.031329362 |
| BP | GO:0090279 | regulation of calcium ion import | 5 | 0.031329362 |
| BP | GO:1900371 | regulation of purine nucleotide biosynthetic process | 5 | 0.031329362 |
| BP | GO:0034614 | cellular response to reactive oxygen species | 12 | 0.031550612 |
| BP | GO:0015909 | long-chain fatty acid transport | 7 | 0.031834479 |
| BP | GO:0035924 | cellular response to vascular endothelial growth factor stimulus | 7 | 0.031834479 |
| BP | GO:0001916 | positive regulation of T cell mediated cytotoxicity | 4 | 0.032773394 |
| BP | GO:0003180 | aortic valve morphogenesis | 4 | 0.032773394 |
| BP | GO:0010575 | positive regulation of vascular endothelial growth factor production | 4 | 0.032773394 |
| BP | GO:0043901 | negative regulation of multi-organism process | 4 | 0.032773394 |
| BP | GO:0090257 | regulation of muscle system process | 16 | 0.033723046 |
| BP | GO:0110053 | regulation of actin filament organization | 17 | 0.03393095 |
| BP | GO:0010660 | regulation of muscle cell apoptotic process | 8 | 0.033982262 |
| BP | GO:0031663 | lipopolysaccharide-mediated signaling pathway | 6 | 0.034107101 |
| BP | GO:0002823 | negative regulation of adaptive immune response based on somatic recombination of immune receptors built from immunoglobulin superfamily domains | 5 | 0.034122944 |
| BP | GO:0030808 | regulation of nucleotide biosynthetic process | 5 | 0.034122944 |
| BP | GO:0045773 | positive regulation of axon extension | 5 | 0.034122944 |
| BP | GO:0035821 | modulation of process of other organism | 9 | 0.034460618 |
| BP | GO:0006937 | regulation of muscle contraction | 12 | 0.035396363 |
| BP | GO:0050769 | positive regulation of neurogenesis | 26 | 0.035490082 |
| BP | GO:0060337 | type I interferon signaling pathway | 8 | 0.035678763 |
| BP | GO:0071357 | cellular response to type I interferon | 8 | 0.035678763 |
| BP | GO:0014015 | positive regulation of gliogenesis | 7 | 0.035678763 |
| BP | GO:0001977 | renal system process involved in regulation of blood volume | 3 | 0.035678763 |
| BP | GO:0002295 | T-helper cell lineage commitment | 3 | 0.035678763 |
| BP | GO:0002830 | positive regulation of type 2 immune response | 3 | 0.035678763 |
| BP | GO:0014061 | regulation of norepinephrine secretion | 3 | 0.035678763 |
| BP | GO:0033008 | positive regulation of mast cell activation involved in immune response | 3 | 0.035678763 |
| BP | GO:0043116 | negative regulation of vascular permeability | 3 | 0.035678763 |
| BP | GO:0043306 | positive regulation of mast cell degranulation | 3 | 0.035678763 |
| BP | GO:0048521 | negative regulation of behavior | 3 | 0.035678763 |
| BP | GO:0071380 | cellular response to prostaglandin E stimulus | 3 | 0.035678763 |
| BP | GO:0072224 | metanephric glomerulus development | 3 | 0.035678763 |
| BP | GO:1900451 | positive regulation of glutamate receptor signaling pathway | 3 | 0.035678763 |
| BP | GO:1905153 | regulation of membrane invagination | 3 | 0.035678763 |
| BP | GO:2001224 | positive regulation of neuron migration | 3 | 0.035678763 |
| BP | GO:0008645 | hexose transmembrane transport | 9 | 0.03581493 |
| BP | GO:0046777 | protein autophosphorylation | 15 | 0.036063223 |
| BP | GO:1903428 | positive regulation of reactive oxygen species biosynthetic process | 6 | 0.036063223 |
| BP | GO:2000242 | negative regulation of reproductive process | 6 | 0.036063223 |
| BP | GO:0007263 | nitric oxide mediated signal transduction | 4 | 0.036063223 |
| BP | GO:0033032 | regulation of myeloid cell apoptotic process | 4 | 0.036063223 |
| BP | GO:0045577 | regulation of B cell differentiation | 4 | 0.036063223 |
| BP | GO:0050901 | leukocyte tethering or rolling | 4 | 0.036063223 |
| BP | GO:2000727 | positive regulation of cardiac muscle cell differentiation | 4 | 0.036063223 |
| BP | GO:0051099 | positive regulation of binding | 12 | 0.036154476 |
| BP | GO:0031670 | cellular response to nutrient | 5 | 0.03656984 |
| BP | GO:0045687 | positive regulation of glial cell differentiation | 5 | 0.03656984 |
| BP | GO:2000404 | regulation of T cell migration | 5 | 0.03656984 |
| BP | GO:0001895 | retina homeostasis | 7 | 0.037612111 |
| BP | GO:0046942 | carboxylic acid transport | 20 | 0.038233343 |
| BP | GO:0003158 | endothelium development | 10 | 0.038233343 |
| BP | GO:0032868 | response to insulin | 17 | 0.038474023 |
| BP | GO:0021872 | forebrain generation of neurons | 6 | 0.038474023 |
| BP | GO:0033619 | membrane protein proteolysis | 6 | 0.038474023 |
| BP | GO:0042267 | natural killer cell mediated cytotoxicity | 6 | 0.038474023 |
| BP | GO:0045843 | negative regulation of striated muscle tissue development | 6 | 0.038474023 |
| BP | GO:0007623 | circadian rhythm | 14 | 0.038767363 |
| BP | GO:0015749 | monosaccharide transmembrane transport | 9 | 0.039074715 |
| BP | GO:0008360 | regulation of cell shape | 11 | 0.039644323 |
| BP | GO:0055026 | negative regulation of cardiac muscle tissue development | 5 | 0.039644323 |
| BP | GO:1904646 | cellular response to amyloid-beta | 5 | 0.039644323 |
| BP | GO:0010827 | regulation of glucose transmembrane transport | 7 | 0.039691507 |
| BP | GO:0070373 | negative regulation of ERK1 and ERK2 cascade | 7 | 0.039691507 |
| BP | GO:0001516 | prostaglandin biosynthetic process | 4 | 0.039815092 |
| BP | GO:0010758 | regulation of macrophage chemotaxis | 4 | 0.039815092 |
| BP | GO:0046457 | prostanoid biosynthetic process | 4 | 0.039815092 |
| BP | GO:0120033 | negative regulation of plasma membrane bounded cell projection assembly | 4 | 0.039815092 |
| BP | GO:1900746 | regulation of vascular endothelial growth factor signaling pathway | 4 | 0.039815092 |
| BP | GO:0045446 | endothelial cell differentiation | 9 | 0.040774874 |
| BP | GO:0015849 | organic acid transport | 20 | 0.040854486 |
| BP | GO:0010657 | muscle cell apoptotic process | 8 | 0.040854486 |
| BP | GO:0048635 | negative regulation of muscle organ development | 6 | 0.040854486 |
| BP | GO:0043271 | negative regulation of ion transport | 11 | 0.040854486 |
| BP | GO:0002643 | regulation of tolerance induction | 3 | 0.040854486 |
| BP | GO:0003184 | pulmonary valve morphogenesis | 3 | 0.040854486 |
| BP | GO:0016045 | detection of bacterium | 3 | 0.040854486 |
| BP | GO:0032966 | negative regulation of collagen biosynthetic process | 3 | 0.040854486 |
| BP | GO:0046851 | negative regulation of bone remodeling | 3 | 0.040854486 |
| BP | GO:0048243 | norepinephrine secretion | 3 | 0.040854486 |
| BP | GO:0060263 | regulation of respiratory burst | 3 | 0.040854486 |
| BP | GO:1903209 | positive regulation of oxidative stress-induced cell death | 3 | 0.040854486 |
| BP | GO:0003151 | outflow tract morphogenesis | 7 | 0.041690224 |
| BP | GO:1903707 | negative regulation of hemopoiesis | 11 | 0.042370233 |
| BP | GO:0034219 | carbohydrate transmembrane transport | 9 | 0.042370233 |
| BP | GO:0051209 | release of sequestered calcium ion into cytosol | 9 | 0.042370233 |
| BP | GO:0050798 | activated T cell proliferation | 5 | 0.042498198 |
| BP | GO:0007044 | cell-substrate junction assembly | 8 | 0.042710526 |
| BP | GO:0034340 | response to type I interferon | 8 | 0.042710526 |
| BP | GO:0055076 | transition metal ion homeostasis | 10 | 0.042710526 |
| BP | GO:0048469 | cell maturation | 12 | 0.042754841 |
| BP | GO:0046622 | positive regulation of organ growth | 6 | 0.043326555 |
| BP | GO:0051893 | regulation of focal adhesion assembly | 6 | 0.043326555 |
| BP | GO:0090109 | regulation of cell-substrate junction assembly | 6 | 0.043326555 |
| BP | GO:0021772 | olfactory bulb development | 4 | 0.043384468 |
| BP | GO:0032770 | positive regulation of monooxygenase activity | 4 | 0.043384468 |
| BP | GO:0034368 | protein-lipid complex remodeling | 4 | 0.043384468 |
| BP | GO:0034369 | plasma lipoprotein particle remodeling | 4 | 0.043384468 |
| BP | GO:0045940 | positive regulation of steroid metabolic process | 4 | 0.043384468 |
| BP | GO:0045987 | positive regulation of smooth muscle contraction | 4 | 0.043384468 |
| BP | GO:0048710 | regulation of astrocyte differentiation | 4 | 0.043384468 |
| BP | GO:0050650 | chondroitin sulfate proteoglycan biosynthetic process | 4 | 0.043384468 |
| BP | GO:0050974 | detection of mechanical stimulus involved in sensory perception | 4 | 0.043384468 |
| BP | GO:0070229 | negative regulation of lymphocyte apoptotic process | 4 | 0.043384468 |
| BP | GO:2000406 | positive regulation of T cell migration | 4 | 0.043384468 |
| BP | GO:0001937 | negative regulation of endothelial cell proliferation | 7 | 0.043623401 |
| BP | GO:0051283 | negative regulation of sequestering of calcium ion | 9 | 0.043848865 |
| BP | GO:0035850 | epithelial cell differentiation involved in kidney development | 5 | 0.045519371 |
| BP | GO:0043300 | regulation of leukocyte degranulation | 5 | 0.045519371 |
| BP | GO:0048483 | autonomic nervous system development | 5 | 0.045519371 |
| BP | GO:0030166 | proteoglycan biosynthetic process | 6 | 0.04573243 |
| BP | GO:0032507 | maintenance of protein location in cell | 6 | 0.04573243 |
| BP | GO:0032835 | glomerulus development | 6 | 0.04573243 |
| BP | GO:0042130 | negative regulation of T cell proliferation | 6 | 0.04573243 |
| BP | GO:0046173 | polyol biosynthetic process | 6 | 0.04573243 |
| BP | GO:0046686 | response to cadmium ion | 6 | 0.04573243 |
| BP | GO:1901862 | negative regulation of muscle tissue development | 6 | 0.04573243 |
| BP | GO:0021954 | central nervous system neuron development | 7 | 0.045925248 |
| BP | GO:1902930 | regulation of alcohol biosynthetic process | 7 | 0.045925248 |
| BP | GO:0010713 | negative regulation of collagen metabolic process | 3 | 0.046412183 |
| BP | GO:0034755 | iron ion transmembrane transport | 3 | 0.046412183 |
| BP | GO:0035994 | response to muscle stretch | 3 | 0.046412183 |
| BP | GO:0061298 | retina vasculature development in camera-type eye | 3 | 0.046412183 |
| BP | GO:0090036 | regulation of protein kinase C signaling | 3 | 0.046412183 |
| BP | GO:0034367 | protein-containing complex remodeling | 4 | 0.04738157 |
| BP | GO:0043304 | regulation of mast cell degranulation | 4 | 0.04738157 |
| BP | GO:0051385 | response to mineralocorticoid | 4 | 0.04738157 |
| BP | GO:0060317 | cardiac epithelial to mesenchymal transition | 4 | 0.04738157 |
| BP | GO:0070528 | protein kinase C signaling | 4 | 0.04738157 |
| BP | GO:0071295 | cellular response to vitamin | 4 | 0.04738157 |
| BP | GO:0097421 | liver regeneration | 4 | 0.04738157 |
| BP | GO:1902547 | regulation of cellular response to vascular endothelial growth factor stimulus | 4 | 0.04738157 |
| BP | GO:0051282 | regulation of sequestering of calcium ion | 9 | 0.047386529 |
| BP | GO:1900542 | regulation of purine nucleotide metabolic process | 9 | 0.047386529 |
| BP | GO:2000243 | positive regulation of reproductive process | 7 | 0.048199079 |
| BP | GO:0030177 | positive regulation of Wnt signaling pathway | 12 | 0.048215067 |
| BP | GO:2001235 | positive regulation of apoptotic signaling pathway | 12 | 0.048215067 |
| BP | GO:0048645 | animal organ formation | 6 | 0.048215067 |
| BP | GO:0098586 | cellular response to virus | 6 | 0.048215067 |
| BP | GO:0110020 | regulation of actomyosin structure organization | 8 | 0.048215067 |
| BP | GO:0150115 | cell-substrate junction organization | 8 | 0.048215067 |
| BP | GO:0002820 | negative regulation of adaptive immune response | 5 | 0.048215067 |
| BP | GO:0009409 | response to cold | 5 | 0.048215067 |
| BP | GO:0031952 | regulation of protein autophosphorylation | 5 | 0.048215067 |
| BP | GO:0055010 | ventricular cardiac muscle tissue morphogenesis | 5 | 0.048215067 |
| BP | GO:0055023 | positive regulation of cardiac muscle tissue growth | 5 | 0.048215067 |
| BP | GO:0021782 | glial cell development | 9 | 0.049236564 |
| BP | GO:0006352 | DNA-templated transcription, initiation | 15 | 0.049618956 |
| CC | GO:0019814 | immunoglobulin complex | 132 | 1.04E-166 |
| CC | GO:0042571 | immunoglobulin complex, circulating | 54 | 7.11E-61 |
| CC | GO:0009897 | external side of plasma membrane | 105 | 7.11E-61 |
| CC | GO:0062023 | collagen-containing extracellular matrix | 69 | 1.84E-26 |
| CC | GO:0072562 | blood microparticle | 38 | 1.25E-21 |
| CC | GO:0034774 | secretory granule lumen | 35 | 3.73E-08 |
| CC | GO:0005788 | endoplasmic reticulum lumen | 34 | 3.73E-08 |
| CC | GO:0060205 | cytoplasmic vesicle lumen | 35 | 3.73E-08 |
| CC | GO:0005796 | Golgi lumen | 19 | 3.73E-08 |
| CC | GO:0031983 | vesicle lumen | 35 | 3.73E-08 |
| CC | GO:0005581 | collagen trimer | 17 | 9.22E-08 |
| CC | GO:0005583 | fibrillar collagen trimer | 6 | 2.46E-05 |
| CC | GO:0098643 | banded collagen fibril | 6 | 2.46E-05 |
| CC | GO:0098644 | complex of collagen trimers | 7 | 7.46E-05 |
| CC | GO:0045121 | membrane raft | 28 | 0.000111256 |
| CC | GO:0098857 | membrane microdomain | 28 | 0.000111256 |
| CC | GO:0098589 | membrane region | 28 | 0.00021609 |
| CC | GO:0031091 | platelet alpha granule | 11 | 0.003646344 |
| CC | GO:0042581 | specific granule | 15 | 0.004776554 |
| CC | GO:0031093 | platelet alpha granule lumen | 9 | 0.00555216 |
| CC | GO:0005901 | caveola | 10 | 0.005605242 |
| CC | GO:0043020 | NADPH oxidase complex | 4 | 0.006931553 |
| CC | GO:0035580 | specific granule lumen | 8 | 0.013410456 |
| CC | GO:0070820 | tertiary granule | 14 | 0.015100843 |
| CC | GO:0044853 | plasma membrane raft | 11 | 0.016787838 |
| CC | GO:0005775 | vacuolar lumen | 14 | 0.023088896 |
| CC | GO:0045178 | basal part of cell | 8 | 0.023088896 |
| CC | GO:0030136 | clathrin-coated vesicle | 15 | 0.023610567 |
| CC | GO:1904724 | tertiary granule lumen | 7 | 0.023749172 |
| CC | GO:0005916 | fascia adherens | 3 | 0.038879129 |
| MF | GO:0030546 | signaling receptor activator activity | 144 | 2.75E-96 |
| MF | GO:0048018 | receptor ligand activity | 143 | 4.41E-96 |
| MF | GO:0003823 | antigen binding | 89 | 1.35E-85 |
| MF | GO:0034987 | immunoglobulin receptor binding | 54 | 1.66E-59 |
| MF | GO:0005125 | cytokine activity | 73 | 2.37E-49 |
| MF | GO:0008083 | growth factor activity | 50 | 2.87E-33 |
| MF | GO:0005179 | hormone activity | 43 | 2.57E-31 |
| MF | GO:0005126 | cytokine receptor binding | 55 | 1.62E-26 |
| MF | GO:0008528 | G protein-coupled peptide receptor activity | 38 | 4.99E-22 |
| MF | GO:0005539 | glycosaminoglycan binding | 46 | 1.08E-21 |
| MF | GO:0001653 | peptide receptor activity | 38 | 1.16E-21 |
| MF | GO:0001664 | G protein-coupled receptor binding | 51 | 1.76E-21 |
| MF | GO:0019955 | cytokine binding | 35 | 1.46E-20 |
| MF | GO:0008009 | chemokine activity | 22 | 8.84E-19 |
| MF | GO:0042379 | chemokine receptor binding | 23 | 5.07E-16 |
| MF | GO:0140375 | immune receptor activity | 30 | 1.22E-15 |
| MF | GO:0004896 | cytokine receptor activity | 25 | 1.16E-14 |
| MF | GO:0019838 | growth factor binding | 29 | 1.18E-14 |
| MF | GO:0008201 | heparin binding | 32 | 1.28E-14 |
| MF | GO:0005201 | extracellular matrix structural constituent | 29 | 4.12E-12 |
| MF | GO:0042562 | hormone binding | 21 | 4.13E-12 |
| MF | GO:1901681 | sulfur compound binding | 35 | 2.55E-11 |
| MF | GO:0042277 | peptide binding | 38 | 3.20E-11 |
| MF | GO:0048020 | CCR chemokine receptor binding | 15 | 2.16E-10 |
| MF | GO:0017046 | peptide hormone binding | 15 | 7.79E-10 |
| MF | GO:0070851 | growth factor receptor binding | 23 | 2.95E-09 |
| MF | GO:0033218 | amide binding | 39 | 4.22E-09 |
| MF | GO:0005178 | integrin binding | 23 | 4.22E-09 |
| MF | GO:0045499 | chemorepellent activity | 11 | 7.84E-09 |
| MF | GO:0005184 | neuropeptide hormone activity | 10 | 2.99E-07 |
| MF | GO:0030215 | semaphorin receptor binding | 9 | 2.99E-07 |
| MF | GO:0061134 | peptidase regulator activity | 26 | 4.50E-07 |
| MF | GO:0019199 | transmembrane receptor protein kinase activity | 15 | 5.13E-07 |
| MF | GO:0030414 | peptidase inhibitor activity | 23 | 7.31E-07 |
| MF | GO:0005518 | collagen binding | 13 | 4.40E-06 |
| MF | GO:0050786 | RAGE receptor binding | 6 | 5.73E-06 |
| MF | GO:0048306 | calcium-dependent protein binding | 14 | 6.94E-06 |
| MF | GO:0004879 | nuclear receptor activity | 11 | 8.06E-06 |
| MF | GO:0098531 | ligand-activated transcription factor activity | 11 | 8.06E-06 |
| MF | GO:0071855 | neuropeptide receptor binding | 9 | 8.18E-06 |
| MF | GO:0016500 | protein-hormone receptor activity | 7 | 9.17E-06 |
| MF | GO:0045236 | CXCR chemokine receptor binding | 7 | 9.17E-06 |
| MF | GO:0005024 | transforming growth factor beta-activated receptor activity | 6 | 1.69E-05 |
| MF | GO:0004866 | endopeptidase inhibitor activity | 20 | 2.29E-05 |
| MF | GO:0061135 | endopeptidase regulator activity | 20 | 4.70E-05 |
| MF | GO:0038187 | pattern recognition receptor activity | 7 | 5.59E-05 |
| MF | GO:0019956 | chemokine binding | 8 | 7.65E-05 |
| MF | GO:0002020 | protease binding | 16 | 9.22E-05 |
| MF | GO:0048029 | monosaccharide binding | 11 | 0.00011742 |
| MF | GO:0048407 | platelet-derived growth factor binding | 5 | 0.000124959 |
| MF | GO:0005501 | retinoid binding | 8 | 0.000138195 |
| MF | GO:0043394 | proteoglycan binding | 8 | 0.000138195 |
| MF | GO:0004867 | serine-type endopeptidase inhibitor activity | 13 | 0.000143176 |
| MF | GO:0019840 | isoprenoid binding | 8 | 0.000161989 |
| MF | GO:0042056 | chemoattractant activity | 8 | 0.000161989 |
| MF | GO:0004675 | transmembrane receptor protein serine/threonine kinase activity | 6 | 0.000173176 |
| MF | GO:0004857 | enzyme inhibitor activity | 29 | 0.000185312 |
| MF | GO:0033612 | receptor serine/threonine kinase binding | 7 | 0.00023835 |
| MF | GO:0016175 | superoxide-generating NAD(P)H oxidase activity | 5 | 0.000279425 |
| MF | GO:0030020 | extracellular matrix structural constituent conferring tensile strength | 8 | 0.000327702 |
| MF | GO:0017134 | fibroblast growth factor binding | 6 | 0.000520785 |
| MF | GO:0050431 | transforming growth factor beta binding | 6 | 0.000520785 |
| MF | GO:0050840 | extracellular matrix binding | 9 | 0.000604216 |
| MF | GO:0019841 | retinol binding | 5 | 0.000793673 |
| MF | GO:0043395 | heparan sulfate proteoglycan binding | 5 | 0.000793673 |
| MF | GO:0005104 | fibroblast growth factor receptor binding | 6 | 0.000801238 |
| MF | GO:0038024 | cargo receptor activity | 10 | 0.000801238 |
| MF | GO:0004714 | transmembrane receptor protein tyrosine kinase activity | 9 | 0.000961446 |
| MF | GO:0042923 | neuropeptide binding | 6 | 0.00098213 |
| MF | GO:0030246 | carbohydrate binding | 21 | 0.001167495 |
| MF | GO:0001968 | fibronectin binding | 6 | 0.001193674 |
| MF | GO:0042834 | peptidoglycan binding | 5 | 0.001331545 |
| MF | GO:0046332 | SMAD binding | 10 | 0.001432355 |
| MF | GO:0030296 | protein tyrosine kinase activator activity | 5 | 0.001688633 |
| MF | GO:0050664 | oxidoreductase activity, acting on NAD(P)H, oxygen as acceptor | 5 | 0.001688633 |
| MF | GO:0005520 | insulin-like growth factor binding | 6 | 0.001693253 |
| MF | GO:0036122 | BMP binding | 4 | 0.00352579 |
| MF | GO:0061783 | peptidoglycan muralytic activity | 4 | 0.00352579 |
| MF | GO:0070700 | BMP receptor binding | 4 | 0.00352579 |
| MF | GO:0005160 | transforming growth factor beta receptor binding | 5 | 0.004934761 |
| MF | GO:0019957 | C-C chemokine binding | 5 | 0.004934761 |
| MF | GO:0070696 | transmembrane receptor protein serine/threonine kinase binding | 5 | 0.004934761 |
| MF | GO:0048185 | activin binding | 4 | 0.006082989 |
| MF | GO:0004252 | serine-type endopeptidase activity | 14 | 0.006778734 |
| MF | GO:1990782 | protein tyrosine kinase binding | 10 | 0.006822088 |
| MF | GO:0001637 | G protein-coupled chemoattractant receptor activity | 5 | 0.006822088 |
| MF | GO:0004950 | chemokine receptor activity | 5 | 0.006822088 |
| MF | GO:0003953 | NAD+ nucleosidase activity | 4 | 0.007288754 |
| MF | GO:0050135 | NAD(P)+ nucleosidase activity | 4 | 0.007288754 |
| MF | GO:0061809 | NAD+ nucleotidase, cyclic ADP-ribose generating | 4 | 0.007288754 |
| MF | GO:0004713 | protein tyrosine kinase activity | 12 | 0.007482357 |
| MF | GO:0050661 | NADP binding | 7 | 0.008446539 |
| MF | GO:0016918 | retinal binding | 4 | 0.008988628 |
| MF | GO:0030971 | receptor tyrosine kinase binding | 8 | 0.010850529 |
| MF | GO:0005164 | tumor necrosis factor receptor binding | 5 | 0.014117205 |
| MF | GO:0008188 | neuropeptide receptor activity | 6 | 0.014806861 |
| MF | GO:0008236 | serine-type peptidase activity | 14 | 0.01505355 |
| MF | GO:0004955 | prostaglandin receptor activity | 3 | 0.015387831 |
| MF | GO:0005031 | tumor necrosis factor-activated receptor activity | 3 | 0.015387831 |
| MF | GO:0017002 | activin-activated receptor activity | 3 | 0.015387831 |
| MF | GO:0001972 | retinoic acid binding | 4 | 0.015594661 |
| MF | GO:0017171 | serine hydrolase activity | 14 | 0.017225929 |
| MF | GO:0005044 | scavenger receptor activity | 6 | 0.017225929 |
| MF | GO:0051428 | peptide hormone receptor binding | 4 | 0.018233177 |
| MF | GO:0032813 | tumor necrosis factor receptor superfamily binding | 6 | 0.018791961 |
| MF | GO:0005154 | epidermal growth factor receptor binding | 5 | 0.019120807 |
| MF | GO:0004954 | prostanoid receptor activity | 3 | 0.019120807 |
| MF | GO:0019864 | IgG binding | 3 | 0.019120807 |
| MF | GO:0005540 | hyaluronic acid binding | 4 | 0.020723391 |
| MF | GO:0016860 | intramolecular oxidoreductase activity | 6 | 0.021995732 |
| MF | GO:0016493 | C-C chemokine receptor activity | 4 | 0.023573926 |
| MF | GO:0004875 | complement receptor activity | 3 | 0.023573926 |
| MF | GO:0005035 | death receptor activity | 3 | 0.023573926 |
| MF | GO:0017154 | semaphorin receptor activity | 3 | 0.023573926 |
| MF | GO:0019865 | immunoglobulin binding | 4 | 0.027106193 |
| MF | GO:0045309 | protein phosphorylated amino acid binding | 6 | 0.027771936 |
| MF | GO:0008179 | adenylate cyclase binding | 3 | 0.029146107 |
| MF | GO:0033293 | monocarboxylic acid binding | 7 | 0.03323006 |
| MF | GO:0003707 | steroid hormone receptor activity | 4 | 0.035016206 |
| MF | GO:0044548 | S100 protein binding | 3 | 0.035304105 |
| MF | GO:0031406 | carboxylic acid binding | 14 | 0.035694799 |
| MF | GO:0001784 | phosphotyrosine residue binding | 5 | 0.041093196 |
| MF | GO:0004953 | icosanoid receptor activity | 3 | 0.042027591 |
| MF | GO:0004869 | cysteine-type endopeptidase inhibitor activity | 6 | 0.04333379 |
| MF | GO:0001540 | amyloid-beta binding | 7 | 0.044613781 |
| MF | GO:0051427 | hormone receptor binding | 12 | 0.046540609 |
| MF | GO:0001223 | transcription coactivator binding | 4 | 0.04777653 |
| MF | GO:0043236 | laminin binding | 4 | 0.04777653 |
| MF | GO:0008191 | metalloendopeptidase inhibitor activity | 3 | 0.04777653 |
| MF | GO:0019211 | phosphatase activator activity | 3 | 0.04777653 |
