# Supplementary material for: A Prognostic Risk Score Based on Hypoxia-, Immunity-, and Epithelialto-Mesenchymal Transition-Related Genes for the Prognosis and Immunotherapy Response of Lung Adenocarcinoma
Source: Front Cell Dev Biol. 2022 Jan 24;9:758777. doi: 10.3389/fcell.2021.758777 (PMC8819669; doi:10.3389/fcell.2021.758777)
Supplement: Supplementary file 3 [file Table7.DOCX]

| **Supplementary Table 7 \| KEGG pathway enrichment analysis of EMT-DEGs** | | | |
| --- | --- | --- | --- |
| ID | Description | Count | qvalue |
| hsa04510 | Focal adhesion | 12 | 1.15E-07 |
| hsa04512 | ECM-receptor interaction | 9 | 1.15E-07 |
| hsa04974 | Protein digestion and absorption | 9 | 3.15E-07 |
| hsa04151 | PI3K-Akt signaling pathway | 12 | 2.47E-05 |
| hsa05412 | Arrhythmogenic right ventricular cardiomyopathy | 5 | 0.002159 |
| hsa05165 | Human papillomavirus infection | 9 | 0.002639 |
| hsa05205 | Proteoglycans in cancer | 7 | 0.003085 |
| hsa05323 | Rheumatoid arthritis | 5 | 0.003085 |
| hsa04657 | IL-17 signaling pathway | 5 | 0.003085 |
| hsa04810 | Regulation of actin cytoskeleton | 7 | 0.00337 |
| hsa04933 | AGE-RAGE signaling pathway in diabetic complications | 5 | 0.00337 |
| hsa04668 | TNF signaling pathway | 5 | 0.005214 |
| hsa04611 | Platelet activation | 5 | 0.007651 |
| hsa04926 | Relaxin signaling pathway | 5 | 0.008491 |
| hsa05410 | Hypertrophic cardiomyopathy | 4 | 0.01586 |
| hsa05146 | Amoebiasis | 4 | 0.022685 |
| hsa05144 | Malaria | 3 | 0.022685 |
| hsa05169 | Epstein-Barr virus infection | 5 | 0.046039 |
