# Supplementary material for: A Prognostic Risk Score Based on Hypoxia-, Immunity-, and Epithelialto-Mesenchymal Transition-Related Genes for the Prognosis and Immunotherapy Response of Lung Adenocarcinoma
Source: Front Cell Dev Biol. 2022 Jan 24;9:758777. doi: 10.3389/fcell.2021.758777 (PMC8819669; doi:10.3389/fcell.2021.758777)
Supplement: Supplementary file 4 [file Image2.pdf]

Supplementary Figure 2 | The difference of frequently mutated genes between high- and low-risk group patients in TCGA cohort

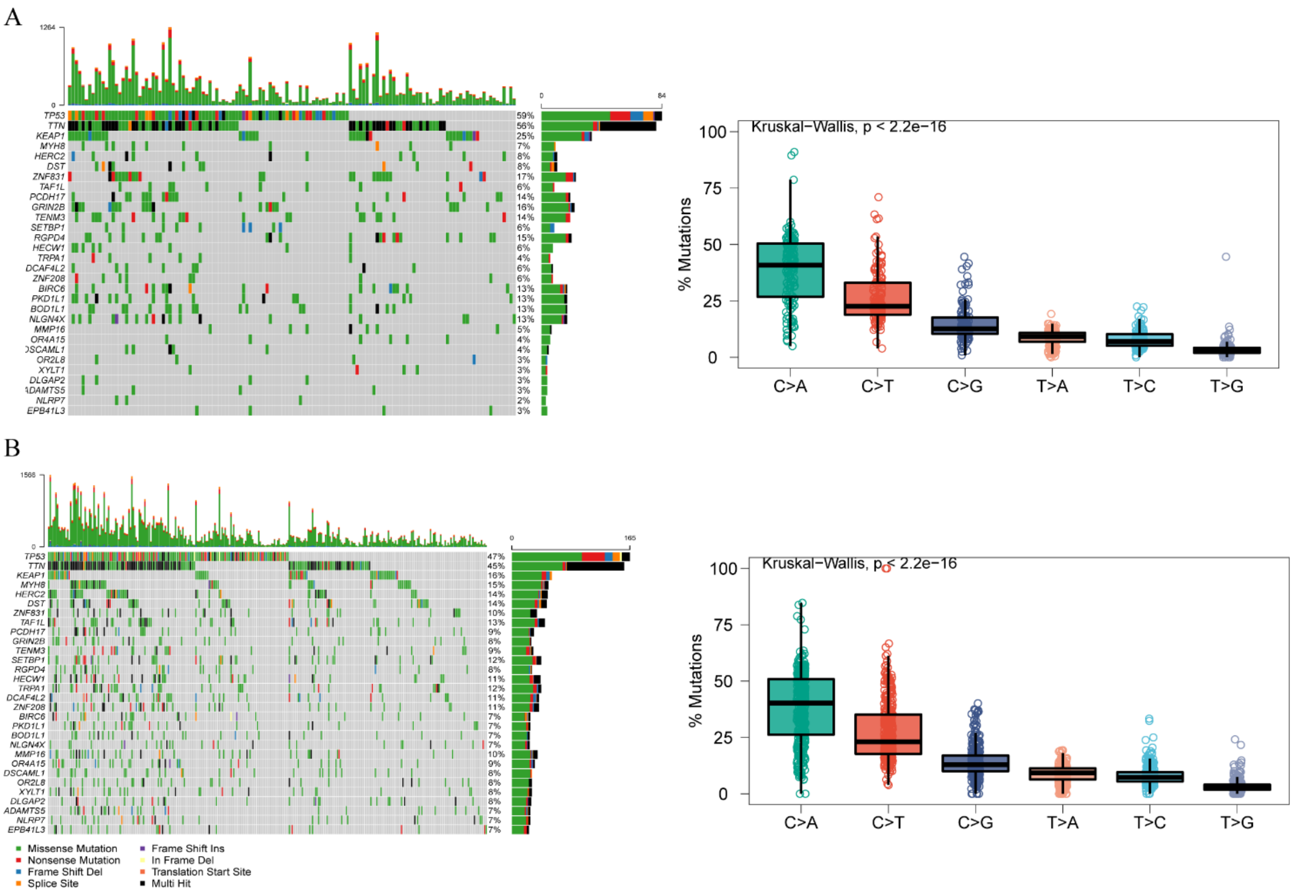

(A) Top 30 most frequently mutated genes were presented in high-risk group patients.

(B) Top 30 most frequently mutated genes were presented in low-risk group patients
