# Supplementary material for: A Prognostic Risk Score Based on Hypoxia-, Immunity-, and Epithelialto-Mesenchymal Transition-Related Genes for the Prognosis and Immunotherapy Response of Lung Adenocarcinoma
Source: Front Cell Dev Biol. 2022 Jan 24;9:758777. doi: 10.3389/fcell.2021.758777 (PMC8819669; doi:10.3389/fcell.2021.758777)
Supplement: Supplementary file 5 [file Table9.docx]

| **Supplementary Table 9 \| KEGG pathway enrichment analysis of integrated-DEGs** | | | |
| --- | --- | --- | --- |
| ID | Description | Count | qvalue |
| hsa04060 | Cytokine-cytokine receptor interaction | 87 | 6.89E-45 |
| hsa04080 | Neuroactive ligand-receptor interaction | 64 | 2.39E-20 |
| hsa04061 | Viral protein interaction with cytokine and cytokine receptor | 33 | 5.99E-18 |
| hsa04630 | JAK-STAT signaling pathway | 32 | 1.59E-10 |
| hsa05323 | Rheumatoid arthritis | 23 | 1.30E-09 |
| hsa04657 | IL-17 signaling pathway | 23 | 1.37E-09 |
| hsa04062 | Chemokine signaling pathway | 32 | 9.64E-09 |
| hsa04650 | Natural killer cell mediated cytotoxicity | 24 | 2.14E-07 |
| hsa04066 | HIF-1 signaling pathway | 21 | 6.56E-07 |
| hsa04151 | PI3K-Akt signaling pathway | 41 | 1.58E-06 |
| hsa04010 | MAPK signaling pathway | 33 | 5.68E-05 |
| hsa04360 | Axon guidance | 24 | 7.77E-05 |
| hsa05321 | Inflammatory bowel disease | 13 | 0.00013 |
| hsa04933 | AGE-RAGE signaling pathway in diabetic complications | 16 | 0.000232 |
| hsa05417 | Lipid and atherosclerosis | 25 | 0.000366 |
| hsa04145 | Phagosome | 20 | 0.000372 |
| hsa04510 | Focal adhesion | 23 | 0.000864 |
| hsa05144 | Malaria | 10 | 0.001023 |
| hsa04064 | NF-kappa B signaling pathway | 15 | 0.001033 |
| hsa04380 | Osteoclast differentiation | 17 | 0.001033 |
| hsa04926 | Relaxin signaling pathway | 17 | 0.001085 |
| hsa04659 | Th17 cell differentiation | 15 | 0.001284 |
| hsa04640 | Hematopoietic cell lineage | 14 | 0.001849 |
| hsa04668 | TNF signaling pathway | 15 | 0.001972 |
| hsa04014 | Ras signaling pathway | 24 | 0.001981 |
| hsa00010 | Glycolysis / Gluconeogenesis | 11 | 0.001981 |
| hsa04024 | cAMP signaling pathway | 23 | 0.001981 |
| hsa05146 | Amoebiasis | 14 | 0.002086 |
| hsa04015 | Rap1 signaling pathway | 22 | 0.00258 |
| hsa04350 | TGF-beta signaling pathway | 13 | 0.002942 |
| hsa05218 | Melanoma | 11 | 0.003261 |
| hsa05418 | Fluid shear stress and atherosclerosis | 16 | 0.005212 |
| hsa04020 | Calcium signaling pathway | 23 | 0.005898 |
| hsa04625 | C-type lectin receptor signaling pathway | 13 | 0.006659 |
| hsa04660 | T cell receptor signaling pathway | 13 | 0.006659 |
| hsa04924 | Renin secretion | 10 | 0.00756 |
| hsa04662 | B cell receptor signaling pathway | 11 | 0.008267 |
| hsa04672 | Intestinal immune network for IgA production | 8 | 0.009748 |
| hsa05167 | Kaposi sarcoma-associated herpesvirus infection | 19 | 0.009808 |
| hsa05332 | Graft-versus-host disease | 7 | 0.015498 |
| hsa05171 | Coronavirus disease - COVID-19 | 21 | 0.015498 |
| hsa04620 | Toll-like receptor signaling pathway | 12 | 0.016631 |
| hsa04614 | Renin-angiotensin system | 5 | 0.017057 |
| hsa01521 | EGFR tyrosine kinase inhibitor resistance | 10 | 0.017071 |
| hsa04658 | Th1 and Th2 cell differentiation | 11 | 0.017071 |
| hsa05150 | Staphylococcus aureus infection | 11 | 0.023134 |
| hsa05162 | Measles | 14 | 0.024871 |
| hsa04012 | ErbB signaling pathway | 10 | 0.026604 |
| hsa04810 | Regulation of actin cytoskeleton | 19 | 0.029644 |
| hsa05205 | Proteoglycans in cancer | 18 | 0.032786 |
| hsa05224 | Breast cancer | 14 | 0.037003 |
| hsa05140 | Leishmaniasis | 9 | 0.037125 |
| hsa04270 | Vascular smooth muscle contraction | 13 | 0.037125 |
| hsa04928 | Parathyroid hormone synthesis, secretion and action | 11 | 0.040746 |
| hsa05320 | Autoimmune thyroid disease | 7 | 0.040766 |
| hsa05219 | Bladder cancer | 6 | 0.040766 |
