# Supplementary material for: A Prognostic Risk Score Based on Hypoxia-, Immunity-, and Epithelialto-Mesenchymal Transition-Related Genes for the Prognosis and Immunotherapy Response of Lung Adenocarcinoma
Source: Front Cell Dev Biol. 2022 Jan 24;9:758777. doi: 10.3389/fcell.2021.758777 (PMC8819669; doi:10.3389/fcell.2021.758777)
Supplement: Supplementary file 6 [file Table2.DOCX]

|  |
| --- |

| **Supplementary Table 2 \| GO enrichment analysis of hypoxia-DEGs** | | | | |
| --- | --- | --- | --- | --- |
| Category | ID | Description | Count | qvalue |
| BP | GO:0006735 | NADH regeneration | 9 | 1.02E-13 |
| BP | GO:0061621 | canonical glycolysis | 9 | 1.02E-13 |
| BP | GO:0061718 | glucose catabolic process to pyruvate | 9 | 1.02E-13 |
| BP | GO:0061620 | glycolytic process through glucose-6-phosphate | 9 | 1.06E-13 |
| BP | GO:0046031 | ADP metabolic process | 13 | 1.06E-13 |
| BP | GO:0061615 | glycolytic process through fructose-6-phosphate | 9 | 1.08E-13 |
| BP | GO:0006165 | nucleoside diphosphate phosphorylation | 13 | 1.74E-13 |
| BP | GO:0046939 | nucleotide phosphorylation | 13 | 1.74E-13 |
| BP | GO:0009135 | purine nucleoside diphosphate metabolic process | 13 | 1.74E-13 |
| BP | GO:0009179 | purine ribonucleoside diphosphate metabolic process | 13 | 1.74E-13 |
| BP | GO:0019674 | NAD metabolic process | 10 | 1.74E-13 |
| BP | GO:0009185 | ribonucleoside diphosphate metabolic process | 13 | 2.01E-13 |
| BP | GO:0006007 | glucose catabolic process | 9 | 4.61E-13 |
| BP | GO:0006090 | pyruvate metabolic process | 13 | 5.59E-13 |
| BP | GO:0006096 | glycolytic process | 12 | 5.62E-13 |
| BP | GO:0006757 | ATP generation from ADP | 12 | 5.86E-13 |
| BP | GO:0009132 | nucleoside diphosphate metabolic process | 13 | 5.95E-13 |
| BP | GO:0006734 | NADH metabolic process | 9 | 1.22E-12 |
| BP | GO:0016052 | carbohydrate catabolic process | 13 | 1.46E-11 |
| BP | GO:0019320 | hexose catabolic process | 9 | 2.29E-11 |
| BP | GO:0016051 | carbohydrate biosynthetic process | 13 | 2.98E-11 |
| BP | GO:0006006 | glucose metabolic process | 13 | 3.02E-11 |
| BP | GO:0046365 | monosaccharide catabolic process | 9 | 9.45E-11 |
| BP | GO:0005996 | monosaccharide metabolic process | 14 | 9.65E-11 |
| BP | GO:0019318 | hexose metabolic process | 13 | 2.35E-10 |
| BP | GO:0006094 | gluconeogenesis | 9 | 1.65E-09 |
| BP | GO:0036293 | response to decreased oxygen levels | 14 | 1.78E-09 |
| BP | GO:0019319 | hexose biosynthetic process | 9 | 2.07E-09 |
| BP | GO:0046034 | ATP metabolic process | 13 | 2.57E-09 |
| BP | GO:0046364 | monosaccharide biosynthetic process | 9 | 3.39E-09 |
| BP | GO:0070482 | response to oxygen levels | 14 | 3.67E-09 |
| BP | GO:0009150 | purine ribonucleotide metabolic process | 14 | 4.19E-09 |
| BP | GO:0006024 | glycosaminoglycan biosynthetic process | 9 | 6.21E-09 |
| BP | GO:0009259 | ribonucleotide metabolic process | 14 | 6.40E-09 |
| BP | GO:0019693 | ribose phosphate metabolic process | 14 | 8.75E-09 |
| BP | GO:0006023 | aminoglycan biosynthetic process | 9 | 1.00E-08 |
| BP | GO:0001666 | response to hypoxia | 13 | 1.19E-08 |
| BP | GO:0006163 | purine nucleotide metabolic process | 14 | 1.27E-08 |
| BP | GO:0072521 | purine-containing compound metabolic process | 14 | 2.90E-08 |
| BP | GO:0030203 | glycosaminoglycan metabolic process | 9 | 1.53E-07 |
| BP | GO:0006022 | aminoglycan metabolic process | 9 | 3.15E-07 |
| BP | GO:0030198 | extracellular matrix organization | 12 | 3.92E-07 |
| BP | GO:0043062 | extracellular structure organization | 12 | 3.94E-07 |
| BP | GO:0062197 | cellular response to chemical stress | 10 | 1.62E-05 |
| BP | GO:0042542 | response to hydrogen peroxide | 7 | 2.62E-05 |
| BP | GO:0044262 | cellular carbohydrate metabolic process | 9 | 2.74E-05 |
| BP | GO:0000302 | response to reactive oxygen species | 8 | 5.21E-05 |
| BP | GO:0006027 | glycosaminoglycan catabolic process | 5 | 7.38E-05 |
| BP | GO:1903510 | mucopolysaccharide metabolic process | 6 | 8.63E-05 |
| BP | GO:0050673 | epithelial cell proliferation | 10 | 0.000111 |
| BP | GO:0006026 | aminoglycan catabolic process | 5 | 0.000111 |
| BP | GO:0006979 | response to oxidative stress | 10 | 0.000119 |
| BP | GO:1900542 | regulation of purine nucleotide metabolic process | 6 | 0.000119 |
| BP | GO:0006140 | regulation of nucleotide metabolic process | 6 | 0.000128 |
| BP | GO:0030388 | fructose 1,6-bisphosphate metabolic process | 3 | 0.000135 |
| BP | GO:0097193 | intrinsic apoptotic signaling pathway | 8 | 0.000207 |
| BP | GO:0030208 | dermatan sulfate biosynthetic process | 3 | 0.000238 |
| BP | GO:0070372 | regulation of ERK1 and ERK2 cascade | 8 | 0.000293 |
| BP | GO:1902895 | positive regulation of pri-miRNA transcription by RNA polymerase II | 4 | 0.000293 |
| BP | GO:0030205 | dermatan sulfate metabolic process | 3 | 0.000293 |
| BP | GO:0034599 | cellular response to oxidative stress | 8 | 0.000308 |
| BP | GO:0030207 | chondroitin sulfate catabolic process | 3 | 0.00036 |
| BP | GO:0070371 | ERK1 and ERK2 cascade | 8 | 0.00042 |
| BP | GO:0050651 | dermatan sulfate proteoglycan biosynthetic process | 3 | 0.000435 |
| BP | GO:1904035 | regulation of epithelial cell apoptotic process | 5 | 0.00046 |
| BP | GO:0050655 | dermatan sulfate proteoglycan metabolic process | 3 | 0.000518 |
| BP | GO:1901342 | regulation of vasculature development | 9 | 0.000528 |
| BP | GO:0031960 | response to corticosteroid | 6 | 0.000528 |
| BP | GO:0044282 | small molecule catabolic process | 9 | 0.000593 |
| BP | GO:0062012 | regulation of small molecule metabolic process | 9 | 0.000626 |
| BP | GO:1902893 | regulation of pri-miRNA transcription by RNA polymerase II | 4 | 0.000656 |
| BP | GO:0048872 | homeostasis of number of cells | 7 | 0.000658 |
| BP | GO:0048660 | regulation of smooth muscle cell proliferation | 6 | 0.000664 |
| BP | GO:0048659 | smooth muscle cell proliferation | 6 | 0.000699 |
| BP | GO:0061614 | pri-miRNA transcription by RNA polymerase II | 4 | 0.000723 |
| BP | GO:0006754 | ATP biosynthetic process | 4 | 0.000769 |
| BP | GO:0046394 | carboxylic acid biosynthetic process | 8 | 0.000818 |
| BP | GO:0016053 | organic acid biosynthetic process | 8 | 0.000823 |
| BP | GO:0001667 | ameboidal-type cell migration | 9 | 0.000841 |
| BP | GO:0042326 | negative regulation of phosphorylation | 9 | 0.000871 |
| BP | GO:1904019 | epithelial cell apoptotic process | 5 | 0.00106 |
| BP | GO:0050678 | regulation of epithelial cell proliferation | 8 | 0.001287 |
| BP | GO:0002688 | regulation of leukocyte chemotaxis | 5 | 0.001346 |
| BP | GO:0034976 | response to endoplasmic reticulum stress | 7 | 0.001346 |
| BP | GO:0030166 | proteoglycan biosynthetic process | 4 | 0.001346 |
| BP | GO:1903578 | regulation of ATP metabolic process | 5 | 0.001368 |
| BP | GO:0045765 | regulation of angiogenesis | 8 | 0.001381 |
| BP | GO:0009206 | purine ribonucleoside triphosphate biosynthetic process | 4 | 0.001381 |
| BP | GO:0009145 | purine nucleoside triphosphate biosynthetic process | 4 | 0.00145 |
| BP | GO:0006109 | regulation of carbohydrate metabolic process | 6 | 0.001582 |
| BP | GO:0002685 | regulation of leukocyte migration | 6 | 0.001648 |
| BP | GO:0030206 | chondroitin sulfate biosynthetic process | 3 | 0.001684 |
| BP | GO:0060326 | cell chemotaxis | 7 | 0.001737 |
| BP | GO:0009201 | ribonucleoside triphosphate biosynthetic process | 4 | 0.001827 |
| BP | GO:0007568 | aging | 7 | 0.001991 |
| BP | GO:0097529 | myeloid leukocyte migration | 6 | 0.002012 |
| BP | GO:0001889 | liver development | 5 | 0.002141 |
| BP | GO:0050920 | regulation of chemotaxis | 6 | 0.002315 |
| BP | GO:0061008 | hepaticobiliary system development | 5 | 0.002315 |
| BP | GO:0048732 | gland development | 8 | 0.002334 |
| BP | GO:0030595 | leukocyte chemotaxis | 6 | 0.002431 |
| BP | GO:0032496 | response to lipopolysaccharide | 7 | 0.002451 |
| BP | GO:0051384 | response to glucocorticoid | 5 | 0.002451 |
| BP | GO:0009205 | purine ribonucleoside triphosphate metabolic process | 4 | 0.002495 |
| BP | GO:0071453 | cellular response to oxygen levels | 6 | 0.002495 |
| BP | GO:0050650 | chondroitin sulfate proteoglycan biosynthetic process | 3 | 0.002495 |
| BP | GO:0009896 | positive regulation of catabolic process | 8 | 0.002579 |
| BP | GO:0009142 | nucleoside triphosphate biosynthetic process | 4 | 0.002781 |
| BP | GO:0048545 | response to steroid hormone | 7 | 0.002851 |
| BP | GO:0002262 | myeloid cell homeostasis | 5 | 0.002851 |
| BP | GO:0033002 | muscle cell proliferation | 6 | 0.002907 |
| BP | GO:0072330 | monocarboxylic acid biosynthetic process | 6 | 0.00301 |
| BP | GO:0009199 | ribonucleoside triphosphate metabolic process | 4 | 0.003053 |
| BP | GO:0007519 | skeletal muscle tissue development | 5 | 0.003099 |
| BP | GO:0034637 | cellular carbohydrate biosynthetic process | 4 | 0.003138 |
| BP | GO:0002237 | response to molecule of bacterial origin | 7 | 0.003199 |
| BP | GO:0009144 | purine nucleoside triphosphate metabolic process | 4 | 0.003225 |
| BP | GO:2001236 | regulation of extrinsic apoptotic signaling pathway | 5 | 0.003362 |
| BP | GO:0006029 | proteoglycan metabolic process | 4 | 0.003611 |
| BP | GO:0010038 | response to metal ion | 7 | 0.003658 |
| BP | GO:0060538 | skeletal muscle organ development | 5 | 0.003878 |
| BP | GO:0071276 | cellular response to cadmium ion | 3 | 0.003998 |
| BP | GO:0034614 | cellular response to reactive oxygen species | 5 | 0.004029 |
| BP | GO:0051591 | response to cAMP | 4 | 0.004093 |
| BP | GO:0030204 | chondroitin sulfate metabolic process | 3 | 0.004216 |
| BP | GO:0009152 | purine ribonucleotide biosynthetic process | 5 | 0.004262 |
| BP | GO:0010634 | positive regulation of epithelial cell migration | 5 | 0.004576 |
| BP | GO:0031331 | positive regulation of cellular catabolic process | 7 | 0.005027 |
| BP | GO:1900371 | regulation of purine nucleotide biosynthetic process | 3 | 0.005094 |
| BP | GO:0042116 | macrophage activation | 4 | 0.005132 |
| BP | GO:0030808 | regulation of nucleotide biosynthetic process | 3 | 0.005379 |
| BP | GO:0001936 | regulation of endothelial cell proliferation | 5 | 0.005395 |
| BP | GO:0048661 | positive regulation of smooth muscle cell proliferation | 4 | 0.005403 |
| BP | GO:0009260 | ribonucleotide biosynthetic process | 5 | 0.005583 |
| BP | GO:0050654 | chondroitin sulfate proteoglycan metabolic process | 3 | 0.005587 |
| BP | GO:0044272 | sulfur compound biosynthetic process | 5 | 0.006355 |
| BP | GO:2001233 | regulation of apoptotic signaling pathway | 7 | 0.006565 |
| BP | GO:0046390 | ribose phosphate biosynthetic process | 5 | 0.006565 |
| BP | GO:0045933 | positive regulation of muscle contraction | 3 | 0.006576 |
| BP | GO:0009141 | nucleoside triphosphate metabolic process | 4 | 0.006576 |
| BP | GO:1901136 | carbohydrate derivative catabolic process | 5 | 0.006576 |
| BP | GO:0006164 | purine nucleotide biosynthetic process | 5 | 0.006728 |
| BP | GO:0031952 | regulation of protein autophosphorylation | 3 | 0.006728 |
| BP | GO:1904036 | negative regulation of epithelial cell apoptotic process | 3 | 0.006728 |
| BP | GO:0010632 | regulation of epithelial cell migration | 6 | 0.006728 |
| BP | GO:0005976 | polysaccharide metabolic process | 4 | 0.006728 |
| BP | GO:0005984 | disaccharide metabolic process | 2 | 0.006728 |
| BP | GO:0010739 | positive regulation of protein kinase A signaling | 2 | 0.006728 |
| BP | GO:0051024 | positive regulation of immunoglobulin secretion | 2 | 0.006728 |
| BP | GO:0001935 | endothelial cell proliferation | 5 | 0.006752 |
| BP | GO:0001938 | positive regulation of endothelial cell proliferation | 4 | 0.006752 |
| BP | GO:0002686 | negative regulation of leukocyte migration | 3 | 0.006818 |
| BP | GO:0031099 | regeneration | 5 | 0.006951 |
| BP | GO:0048146 | positive regulation of fibroblast proliferation | 3 | 0.007569 |
| BP | GO:0006002 | fructose 6-phosphate metabolic process | 2 | 0.007743 |
| BP | GO:2001238 | positive regulation of extrinsic apoptotic signaling pathway | 3 | 0.007852 |
| BP | GO:0071456 | cellular response to hypoxia | 5 | 0.007852 |
| BP | GO:0072522 | purine-containing compound biosynthetic process | 5 | 0.007852 |
| BP | GO:0009612 | response to mechanical stimulus | 5 | 0.007973 |
| BP | GO:0032964 | collagen biosynthetic process | 3 | 0.008154 |
| BP | GO:0050679 | positive regulation of epithelial cell proliferation | 5 | 0.008217 |
| BP | GO:0001933 | negative regulation of protein phosphorylation | 7 | 0.008549 |
| BP | GO:0031953 | negative regulation of protein autophosphorylation | 2 | 0.008682 |
| BP | GO:0070374 | positive regulation of ERK1 and ERK2 cascade | 5 | 0.008775 |
| BP | GO:0030968 | endoplasmic reticulum unfolded protein response | 4 | 0.009014 |
| BP | GO:0036294 | cellular response to decreased oxygen levels | 5 | 0.009164 |
| BP | GO:0044273 | sulfur compound catabolic process | 3 | 0.009164 |
| BP | GO:0042113 | B cell activation | 6 | 0.009164 |
| BP | GO:0030213 | hyaluronan biosynthetic process | 2 | 0.009748 |
| BP | GO:0018209 | peptidyl-serine modification | 6 | 0.009778 |
| BP | GO:0034101 | erythrocyte homeostasis | 4 | 0.009778 |
| BP | GO:0044706 | multi-multicellular organism process | 5 | 0.01045 |
| BP | GO:0010595 | positive regulation of endothelial cell migration | 4 | 0.010524 |
| BP | GO:0006000 | fructose metabolic process | 2 | 0.010715 |
| BP | GO:0044849 | estrous cycle | 2 | 0.010715 |
| BP | GO:0045779 | negative regulation of bone resorption | 2 | 0.010715 |
| BP | GO:0045820 | negative regulation of glycolytic process | 2 | 0.010715 |
| BP | GO:0046683 | response to organophosphorus | 4 | 0.010814 |
| BP | GO:0097191 | extrinsic apoptotic signaling pathway | 5 | 0.010853 |
| BP | GO:1902041 | regulation of extrinsic apoptotic signaling pathway via death domain receptors | 3 | 0.010915 |
| BP | GO:0031667 | response to nutrient levels | 7 | 0.011038 |
| BP | GO:0030183 | B cell differentiation | 4 | 0.011477 |
| BP | GO:1904018 | positive regulation of vasculature development | 5 | 0.011669 |
| BP | GO:0030336 | negative regulation of cell migration | 6 | 0.011669 |
| BP | GO:0046686 | response to cadmium ion | 3 | 0.012148 |
| BP | GO:0010594 | regulation of endothelial cell migration | 5 | 0.012148 |
| BP | GO:0007178 | transmembrane receptor protein serine/threonine kinase signaling pathway | 6 | 0.01287 |
| BP | GO:0009312 | oligosaccharide biosynthetic process | 2 | 0.01287 |
| BP | GO:0010715 | regulation of extracellular matrix disassembly | 2 | 0.01287 |
| BP | GO:0046851 | negative regulation of bone remodeling | 2 | 0.01287 |
| BP | GO:0070997 | neuron death | 6 | 0.012971 |
| BP | GO:0002700 | regulation of production of molecular mediator of immune response | 4 | 0.012999 |
| BP | GO:0050921 | positive regulation of chemotaxis | 4 | 0.012999 |
| BP | GO:0033135 | regulation of peptidyl-serine phosphorylation | 4 | 0.013116 |
| BP | GO:0002548 | monocyte chemotaxis | 3 | 0.013116 |
| BP | GO:0050918 | positive chemotaxis | 3 | 0.013116 |
| BP | GO:0043687 | post-translational protein modification | 6 | 0.013116 |
| BP | GO:0034620 | cellular response to unfolded protein | 4 | 0.013283 |
| BP | GO:0010631 | epithelial cell migration | 6 | 0.013283 |
| BP | GO:2000146 | negative regulation of cell motility | 6 | 0.013283 |
| BP | GO:0051592 | response to calcium ion | 4 | 0.013461 |
| BP | GO:0035994 | response to muscle stretch | 2 | 0.013521 |
| BP | GO:0051023 | regulation of immunoglobulin secretion | 2 | 0.013521 |
| BP | GO:0090132 | epithelium migration | 6 | 0.01353 |
| BP | GO:0010675 | regulation of cellular carbohydrate metabolic process | 4 | 0.01353 |
| BP | GO:0035914 | skeletal muscle cell differentiation | 3 | 0.013546 |
| BP | GO:0048662 | negative regulation of smooth muscle cell proliferation | 3 | 0.013546 |
| BP | GO:0014074 | response to purine-containing compound | 4 | 0.013667 |
| BP | GO:0031668 | cellular response to extracellular stimulus | 5 | 0.014134 |
| BP | GO:0090130 | tissue migration | 6 | 0.014302 |
| BP | GO:0002689 | negative regulation of leukocyte chemotaxis | 2 | 0.015783 |
| BP | GO:0034138 | toll-like receptor 3 signaling pathway | 2 | 0.015783 |
| BP | GO:0070262 | peptidyl-serine dephosphorylation | 2 | 0.015783 |
| BP | GO:0071243 | cellular response to arsenic-containing substance | 2 | 0.015783 |
| BP | GO:2001169 | regulation of ATP biosynthetic process | 2 | 0.015783 |
| BP | GO:0045637 | regulation of myeloid cell differentiation | 5 | 0.016188 |
| BP | GO:0031100 | animal organ regeneration | 3 | 0.016331 |
| BP | GO:0043627 | response to estrogen | 3 | 0.016331 |
| BP | GO:0009165 | nucleotide biosynthetic process | 5 | 0.016485 |
| BP | GO:1903524 | positive regulation of blood circulation | 3 | 0.016485 |
| BP | GO:0036499 | PERK-mediated unfolded protein response | 2 | 0.016485 |
| BP | GO:0048305 | immunoglobulin secretion | 2 | 0.016485 |
| BP | GO:0072111 | cell proliferation involved in kidney development | 2 | 0.016485 |
| BP | GO:0072574 | hepatocyte proliferation | 2 | 0.016485 |
| BP | GO:0072575 | epithelial cell proliferation involved in liver morphogenesis | 2 | 0.016485 |
| BP | GO:0140467 | integrated stress response signaling | 2 | 0.016485 |
| BP | GO:1900543 | negative regulation of purine nucleotide metabolic process | 2 | 0.016485 |
| BP | GO:0043467 | regulation of generation of precursor metabolites and energy | 4 | 0.016834 |
| BP | GO:1901293 | nucleoside phosphate biosynthetic process | 5 | 0.016834 |
| BP | GO:0000271 | polysaccharide biosynthetic process | 3 | 0.017235 |
| BP | GO:0060395 | SMAD protein signal transduction | 3 | 0.017235 |
| BP | GO:0040013 | negative regulation of locomotion | 6 | 0.017367 |
| BP | GO:0042493 | response to drug | 6 | 0.017367 |
| BP | GO:0034104 | negative regulation of tissue remodeling | 2 | 0.017402 |
| BP | GO:0045980 | negative regulation of nucleotide metabolic process | 2 | 0.017402 |
| BP | GO:0072576 | liver morphogenesis | 2 | 0.017402 |
| BP | GO:0043536 | positive regulation of blood vessel endothelial cell migration | 3 | 0.017418 |
| BP | GO:0051271 | negative regulation of cellular component movement | 6 | 0.017604 |
| BP | GO:0035967 | cellular response to topologically incorrect protein | 4 | 0.017604 |
| BP | GO:0006110 | regulation of glycolytic process | 3 | 0.017826 |
| BP | GO:0051100 | negative regulation of binding | 4 | 0.018469 |
| BP | GO:0032703 | negative regulation of interleukin-2 production | 2 | 0.018469 |
| BP | GO:1904996 | positive regulation of leukocyte adhesion to vascular endothelial cell | 2 | 0.018469 |
| BP | GO:0002718 | regulation of cytokine production involved in immune response | 3 | 0.019384 |
| BP | GO:0048145 | regulation of fibroblast proliferation | 3 | 0.019384 |
| BP | GO:0050714 | positive regulation of protein secretion | 4 | 0.019433 |
| BP | GO:0006937 | regulation of muscle contraction | 4 | 0.019697 |
| BP | GO:2000637 | positive regulation of gene silencing by miRNA | 2 | 0.019697 |
| BP | GO:0048144 | fibroblast proliferation | 3 | 0.01973 |
| BP | GO:0043542 | endothelial cell migration | 5 | 0.020064 |
| BP | GO:0072593 | reactive oxygen species metabolic process | 5 | 0.020588 |
| BP | GO:0001776 | leukocyte homeostasis | 3 | 0.020835 |
| BP | GO:0010738 | regulation of protein kinase A signaling | 2 | 0.020857 |
| BP | GO:0060148 | positive regulation of posttranscriptional gene silencing | 2 | 0.020857 |
| BP | GO:1904705 | regulation of vascular associated smooth muscle cell proliferation | 3 | 0.021189 |
| BP | GO:1990874 | vascular associated smooth muscle cell proliferation | 3 | 0.021189 |
| BP | GO:0000188 | inactivation of MAPK activity | 2 | 0.022195 |
| BP | GO:0031664 | regulation of lipopolysaccharide-mediated signaling pathway | 2 | 0.022195 |
| BP | GO:0008625 | extrinsic apoptotic signaling pathway via death domain receptors | 3 | 0.022328 |
| BP | GO:0006986 | response to unfolded protein | 4 | 0.022865 |
| BP | GO:0043470 | regulation of carbohydrate catabolic process | 3 | 0.022865 |
| BP | GO:0030099 | myeloid cell differentiation | 6 | 0.023131 |
| BP | GO:0051899 | membrane depolarization | 3 | 0.023285 |
| BP | GO:0002026 | regulation of the force of heart contraction | 2 | 0.023285 |
| BP | GO:0032897 | negative regulation of viral transcription | 2 | 0.023285 |
| BP | GO:0008217 | regulation of blood pressure | 4 | 0.024218 |
| BP | GO:1903579 | negative regulation of ATP metabolic process | 2 | 0.024833 |
| BP | GO:0003018 | vascular process in circulatory system | 4 | 0.025432 |
| BP | GO:0003073 | regulation of systemic arterial blood pressure | 3 | 0.025816 |
| BP | GO:0018105 | peptidyl-serine phosphorylation | 5 | 0.026144 |
| BP | GO:0001782 | B cell homeostasis | 2 | 0.026219 |
| BP | GO:0002690 | positive regulation of leukocyte chemotaxis | 3 | 0.02629 |
| BP | GO:0002793 | positive regulation of peptide secretion | 4 | 0.026291 |
| BP | GO:0071248 | cellular response to metal ion | 4 | 0.026291 |
| BP | GO:1903532 | positive regulation of secretion by cell | 5 | 0.026573 |
| BP | GO:0071674 | mononuclear cell migration | 3 | 0.026573 |
| BP | GO:1901216 | positive regulation of neuron death | 3 | 0.026573 |
| BP | GO:0050864 | regulation of B cell activation | 4 | 0.026783 |
| BP | GO:0001516 | prostaglandin biosynthetic process | 2 | 0.026783 |
| BP | GO:0033137 | negative regulation of peptidyl-serine phosphorylation | 2 | 0.026783 |
| BP | GO:0046457 | prostanoid biosynthetic process | 2 | 0.026783 |
| BP | GO:0060055 | angiogenesis involved in wound healing | 2 | 0.026783 |
| BP | GO:0061082 | myeloid leukocyte cytokine production | 2 | 0.026783 |
| BP | GO:0007565 | female pregnancy | 4 | 0.026783 |
| BP | GO:0051090 | regulation of DNA-binding transcription factor activity | 6 | 0.027634 |
| BP | GO:0002702 | positive regulation of production of molecular mediator of immune response | 3 | 0.027919 |
| BP | GO:0070301 | cellular response to hydrogen peroxide | 3 | 0.027919 |
| BP | GO:1901654 | response to ketone | 4 | 0.028356 |
| BP | GO:0044264 | cellular polysaccharide metabolic process | 3 | 0.028406 |
| BP | GO:0045639 | positive regulation of myeloid cell differentiation | 3 | 0.028406 |
| BP | GO:0010952 | positive regulation of peptidase activity | 4 | 0.028559 |
| BP | GO:0009746 | response to hexose | 4 | 0.028837 |
| BP | GO:0009306 | protein secretion | 6 | 0.028837 |
| BP | GO:0002683 | negative regulation of immune system process | 6 | 0.028837 |
| BP | GO:0035592 | establishment of protein localization to extracellular region | 6 | 0.028837 |
| BP | GO:0050869 | negative regulation of B cell activation | 2 | 0.028837 |
| BP | GO:0051385 | response to mineralocorticoid | 2 | 0.028837 |
| BP | GO:0070528 | protein kinase C signaling | 2 | 0.028837 |
| BP | GO:0097421 | liver regeneration | 2 | 0.028837 |
| BP | GO:0008630 | intrinsic apoptotic signaling pathway in response to DNA damage | 3 | 0.028949 |
| BP | GO:0071496 | cellular response to external stimulus | 5 | 0.028949 |
| BP | GO:0052547 | regulation of peptidase activity | 6 | 0.029238 |
| BP | GO:0035966 | response to topologically incorrect protein | 4 | 0.029295 |
| BP | GO:0003012 | muscle system process | 6 | 0.029295 |
| BP | GO:0002367 | cytokine production involved in immune response | 3 | 0.029295 |
| BP | GO:0042594 | response to starvation | 4 | 0.029526 |
| BP | GO:0033028 | myeloid cell apoptotic process | 2 | 0.029526 |
| BP | GO:0044319 | wound healing, spreading of cells | 2 | 0.029526 |
| BP | GO:0090505 | epiboly involved in wound healing | 2 | 0.029526 |
| BP | GO:0071692 | protein localization to extracellular region | 6 | 0.029526 |
| BP | GO:0034284 | response to monosaccharide | 4 | 0.029526 |
| BP | GO:0050866 | negative regulation of cell activation | 4 | 0.029526 |
| BP | GO:0045766 | positive regulation of angiogenesis | 4 | 0.029836 |
| BP | GO:0071222 | cellular response to lipopolysaccharide | 4 | 0.029836 |
| BP | GO:0002573 | myeloid leukocyte differentiation | 4 | 0.030339 |
| BP | GO:0010762 | regulation of fibroblast migration | 2 | 0.030339 |
| BP | GO:0046685 | response to arsenic-containing substance | 2 | 0.030339 |
| BP | GO:0050715 | positive regulation of cytokine secretion | 2 | 0.030339 |
| BP | GO:0090504 | epiboly | 2 | 0.030339 |
| BP | GO:2000352 | negative regulation of endothelial cell apoptotic process | 2 | 0.030339 |
| BP | GO:0033138 | positive regulation of peptidyl-serine phosphorylation | 3 | 0.031596 |
| BP | GO:0043618 | regulation of transcription from RNA polymerase II promoter in response to stress | 3 | 0.031596 |
| BP | GO:0030513 | positive regulation of BMP signaling pathway | 2 | 0.031807 |
| BP | GO:0051047 | positive regulation of secretion | 5 | 0.031906 |
| BP | GO:0032680 | regulation of tumor necrosis factor production | 3 | 0.031906 |
| BP | GO:0090100 | positive regulation of transmembrane receptor protein serine/threonine kinase signaling pathway | 3 | 0.031906 |
| BP | GO:1904659 | glucose transmembrane transport | 3 | 0.031906 |
| BP | GO:0032963 | collagen metabolic process | 3 | 0.034149 |
| BP | GO:1903555 | regulation of tumor necrosis factor superfamily cytokine production | 3 | 0.034149 |
| BP | GO:0007599 | hemostasis | 5 | 0.034482 |
| BP | GO:0030212 | hyaluronan metabolic process | 2 | 0.034482 |
| BP | GO:1904994 | regulation of leukocyte adhesion to vascular endothelial cell | 2 | 0.034482 |
| BP | GO:0008645 | hexose transmembrane transport | 3 | 0.034518 |
| BP | GO:0031669 | cellular response to nutrient levels | 4 | 0.034518 |
| BP | GO:0071241 | cellular response to inorganic substance | 4 | 0.034518 |
| BP | GO:0071219 | cellular response to molecule of bacterial origin | 4 | 0.034956 |
| BP | GO:0043620 | regulation of DNA-templated transcription in response to stress | 3 | 0.034983 |
| BP | GO:0002699 | positive regulation of immune effector process | 4 | 0.035197 |
| BP | GO:0050708 | regulation of protein secretion | 5 | 0.035197 |
| BP | GO:0045880 | positive regulation of smoothened signaling pathway | 2 | 0.035197 |
| BP | GO:1905898 | positive regulation of response to endoplasmic reticulum stress | 2 | 0.035197 |
| BP | GO:0015749 | monosaccharide transmembrane transport | 3 | 0.035197 |
| BP | GO:0032640 | tumor necrosis factor production | 3 | 0.035197 |
| BP | GO:0051341 | regulation of oxidoreductase activity | 3 | 0.035929 |
| BP | GO:0034219 | carbohydrate transmembrane transport | 3 | 0.036648 |
| BP | GO:0010737 | protein kinase A signaling | 2 | 0.036648 |
| BP | GO:0071706 | tumor necrosis factor superfamily cytokine production | 3 | 0.037308 |
| BP | GO:0006936 | muscle contraction | 5 | 0.038531 |
| BP | GO:0043903 | regulation of symbiotic process | 4 | 0.038585 |
| BP | GO:0010906 | regulation of glucose metabolic process | 3 | 0.038599 |
| BP | GO:0030218 | erythrocyte differentiation | 3 | 0.038599 |
| BP | GO:0009266 | response to temperature stimulus | 4 | 0.03918 |
| BP | GO:0009743 | response to carbohydrate | 4 | 0.03918 |
| BP | GO:2001234 | negative regulation of apoptotic signaling pathway | 4 | 0.03918 |
| BP | GO:0051354 | negative regulation of oxidoreductase activity | 2 | 0.039239 |
| BP | GO:1902042 | negative regulation of extrinsic apoptotic signaling pathway via death domain receptors | 2 | 0.039239 |
| BP | GO:0007050 | cell cycle arrest | 4 | 0.039424 |
| BP | GO:0002761 | regulation of myeloid leukocyte differentiation | 3 | 0.039573 |
| BP | GO:0022612 | gland morphogenesis | 3 | 0.040341 |
| BP | GO:0048246 | macrophage chemotaxis | 2 | 0.040586 |
| BP | GO:0045862 | positive regulation of proteolysis | 5 | 0.040586 |
| BP | GO:0006790 | sulfur compound metabolic process | 5 | 0.040918 |
| BP | GO:0045124 | regulation of bone resorption | 2 | 0.042072 |
| BP | GO:0045823 | positive regulation of heart contraction | 2 | 0.042072 |
| BP | GO:0014066 | regulation of phosphatidylinositol 3-kinase signaling | 3 | 0.042338 |
| BP | GO:0032965 | regulation of collagen biosynthetic process | 2 | 0.043618 |
| BP | GO:1903053 | regulation of extracellular matrix organization | 2 | 0.043618 |
| BP | GO:0002791 | regulation of peptide secretion | 5 | 0.044803 |
| BP | GO:0071216 | cellular response to biotic stimulus | 4 | 0.044812 |
| BP | GO:0002639 | positive regulation of immunoglobulin production | 2 | 0.044812 |
| BP | GO:0010677 | negative regulation of cellular carbohydrate metabolic process | 2 | 0.044812 |
| BP | GO:0032722 | positive regulation of chemokine production | 2 | 0.044812 |
| BP | GO:0042771 | intrinsic apoptotic signaling pathway in response to DNA damage by p53 class mediator | 2 | 0.044812 |
| BP | GO:0010761 | fibroblast migration | 2 | 0.046371 |
| BP | GO:0032570 | response to progesterone | 2 | 0.046371 |
| BP | GO:0044275 | cellular carbohydrate catabolic process | 2 | 0.046371 |
| BP | GO:0014706 | striated muscle tissue development | 5 | 0.047529 |
| BP | GO:0044088 | regulation of vacuole organization | 2 | 0.048068 |
| BP | GO:0090092 | regulation of transmembrane receptor protein serine/threonine kinase signaling pathway | 4 | 0.049056 |
| CC | GO:0005796 | Golgi lumen | 7 | 4.88E-06 |
| CC | GO:0005788 | endoplasmic reticulum lumen | 9 | 3.95E-05 |
| CC | GO:0062023 | collagen-containing extracellular matrix | 10 | 4.62E-05 |
| CC | GO:0043202 | lysosomal lumen | 5 | 0.0004 |
| CC | GO:0005901 | caveola | 4 | 0.003001 |
| CC | GO:0005775 | vacuolar lumen | 5 | 0.004351 |
| CC | GO:0044853 | plasma membrane raft | 4 | 0.007298 |
| CC | GO:0031430 | M band | 2 | 0.035368 |
| CC | GO:0005938 | cell cortex | 5 | 0.036047 |
| MF | GO:0019838 | growth factor binding | 7 | 8.13E-05 |
| MF | GO:0005539 | glycosaminoglycan binding | 8 | 0.000125 |
| MF | GO:0005520 | insulin-like growth factor binding | 4 | 0.000169 |
| MF | GO:0048029 | monosaccharide binding | 5 | 0.000179 |
| MF | GO:0008083 | growth factor activity | 6 | 0.000736 |
| MF | GO:0016860 | intramolecular oxidoreductase activity | 4 | 0.000774 |
| MF | GO:0030246 | carbohydrate binding | 7 | 0.000981 |
| MF | GO:0042056 | chemoattractant activity | 3 | 0.004809 |
| MF | GO:0048018 | receptor ligand activity | 8 | 0.004809 |
| MF | GO:0008201 | heparin binding | 5 | 0.004809 |
| MF | GO:0030546 | signaling receptor activator activity | 8 | 0.004809 |
| MF | GO:0048407 | platelet-derived growth factor binding | 2 | 0.008815 |
| MF | GO:0072542 | protein phosphatase activator activity | 2 | 0.011075 |
| MF | GO:0051287 | NAD binding | 3 | 0.011075 |
| MF | GO:0016208 | AMP binding | 2 | 0.011589 |
| MF | GO:0034483 | heparan sulfate sulfotransferase activity | 2 | 0.012507 |
| MF | GO:0019211 | phosphatase activator activity | 2 | 0.013422 |
| MF | GO:0005178 | integrin binding | 4 | 0.014999 |
| MF | GO:0016853 | isomerase activity | 4 | 0.017164 |
| MF | GO:0016835 | carbon-oxygen lyase activity | 3 | 0.017164 |
| MF | GO:1901681 | sulfur compound binding | 5 | 0.017164 |
| MF | GO:0005355 | glucose transmembrane transporter activity | 2 | 0.017164 |
| MF | GO:0015149 | hexose transmembrane transporter activity | 2 | 0.017164 |
| MF | GO:0005540 | hyaluronic acid binding | 2 | 0.017873 |
| MF | GO:0015145 | monosaccharide transmembrane transporter activity | 2 | 0.017873 |
| MF | GO:0070412 | R-SMAD binding | 2 | 0.017873 |
| MF | GO:0005201 | extracellular matrix structural constituent | 4 | 0.017873 |
| MF | GO:0051119 | sugar transmembrane transporter activity | 2 | 0.019959 |
| MF | GO:0001968 | fibronectin binding | 2 | 0.022444 |
| MF | GO:0016829 | lyase activity | 4 | 0.023964 |
| MF | GO:0071889 | 14-3-3 protein binding | 2 | 0.029334 |
| MF | GO:0016620 | oxidoreductase activity, acting on the aldehyde or oxo group of donors, NAD or NADP as acceptor | 2 | 0.033858 |
| MF | GO:0015144 | carbohydrate transmembrane transporter activity | 2 | 0.036583 |
| MF | GO:0005125 | cytokine activity | 4 | 0.045079 |
| MF | GO:0016903 | oxidoreductase activity, acting on the aldehyde or oxo group of donors | 2 | 0.046134 |
