# Supplementary material for: A Prognostic Risk Score Based on Hypoxia-, Immunity-, and Epithelialto-Mesenchymal Transition-Related Genes for the Prognosis and Immunotherapy Response of Lung Adenocarcinoma
Source: Front Cell Dev Biol. 2022 Jan 24;9:758777. doi: 10.3389/fcell.2021.758777 (PMC8819669; doi:10.3389/fcell.2021.758777)
Supplement: Supplementary file 7 [file Table5.DOCX]

| **Supplementary Table 5 \| KEGG pathway enrichment analysis of immune-DEGs** | | | |
| --- | --- | --- | --- |
| ID | Description | Count | qvalue |
| hsa04060 | Cytokine-cytokine receptor interaction | 87 | 3.63E-52 |
| hsa04080 | Neuroactive ligand-receptor interaction | 64 | 4.93E-25 |
| hsa04061 | Viral protein interaction with cytokine and cytokine receptor | 33 | 1.58E-20 |
| hsa04630 | JAK-STAT signaling pathway | 32 | 8.58E-13 |
| hsa04062 | Chemokine signaling pathway | 32 | 9.31E-11 |
| hsa05323 | Rheumatoid arthritis | 21 | 1.44E-09 |
| hsa04657 | IL-17 signaling pathway | 21 | 1.53E-09 |
| hsa04650 | Natural killer cell mediated cytotoxicity | 24 | 4.68E-09 |
| hsa05321 | Inflammatory bowel disease | 13 | 2.20E-05 |
| hsa04010 | MAPK signaling pathway | 30 | 2.65E-05 |
| hsa04151 | PI3K-Akt signaling pathway | 33 | 5.35E-05 |
| hsa04360 | Axon guidance | 21 | 0.000133 |
| hsa04380 | Osteoclast differentiation | 17 | 0.000138 |
| hsa04024 | cAMP signaling pathway | 23 | 0.000205 |
| hsa04659 | Th17 cell differentiation | 15 | 0.000206 |
| hsa04064 | NF-kappa B signaling pathway | 14 | 0.000582 |
| hsa04924 | Renin secretion | 11 | 0.000729 |
| hsa04145 | Phagosome | 17 | 0.000937 |
| hsa05417 | Lipid and atherosclerosis | 21 | 0.00102 |
| hsa04014 | Ras signaling pathway | 22 | 0.001033 |
| hsa04640 | Hematopoietic cell lineage | 13 | 0.001057 |
| hsa04933 | AGE-RAGE signaling pathway in diabetic complications | 13 | 0.001119 |
| hsa04020 | Calcium signaling pathway | 22 | 0.001469 |
| hsa04625 | C-type lectin receptor signaling pathway | 13 | 0.001469 |
| hsa04660 | T cell receptor signaling pathway | 13 | 0.001469 |
| hsa04015 | Rap1 signaling pathway | 20 | 0.001522 |
| hsa04350 | TGF-beta signaling pathway | 12 | 0.001942 |
| hsa04066 | HIF-1 signaling pathway | 13 | 0.002073 |
| hsa04662 | B cell receptor signaling pathway | 11 | 0.002073 |
| hsa04668 | TNF signaling pathway | 13 | 0.002559 |
| hsa05218 | Melanoma | 10 | 0.002698 |
| hsa04672 | Intestinal immune network for IgA production | 8 | 0.003146 |
| hsa05167 | Kaposi sarcoma-associated herpesvirus infection | 18 | 0.003146 |
| hsa05144 | Malaria | 8 | 0.003438 |
| hsa04620 | Toll-like receptor signaling pathway | 12 | 0.003807 |
| hsa04658 | Th1 and Th2 cell differentiation | 11 | 0.00451 |
| hsa01521 | EGFR tyrosine kinase inhibitor resistance | 10 | 0.004755 |
| hsa05332 | Graft-versus-host disease | 7 | 0.004923 |
| hsa05162 | Measles | 14 | 0.004923 |
| hsa05418 | Fluid shear stress and atherosclerosis | 14 | 0.004923 |
| hsa05150 | Staphylococcus aureus infection | 11 | 0.005646 |
| hsa04926 | Relaxin signaling pathway | 13 | 0.006895 |
| hsa04614 | Renin-angiotensin system | 5 | 0.006895 |
| hsa04012 | ErbB signaling pathway | 10 | 0.007045 |
| hsa05171 | Coronavirus disease - COVID-19 | 19 | 0.007804 |
| hsa05146 | Amoebiasis | 11 | 0.008237 |
| hsa05140 | Leishmaniasis | 9 | 0.011529 |
| hsa05320 | Autoimmune thyroid disease | 7 | 0.016122 |
| hsa05224 | Breast cancer | 13 | 0.018603 |
| hsa04917 | Prolactin signaling pathway | 8 | 0.020743 |
| hsa04270 | Vascular smooth muscle contraction | 12 | 0.020894 |
| hsa04923 | Regulation of lipolysis in adipocytes | 7 | 0.022315 |
| hsa05235 | PD-L1 expression and PD-1 checkpoint pathway in cancer | 9 | 0.026664 |
| hsa04928 | Parathyroid hormone synthesis, secretion and action | 10 | 0.028201 |
| hsa05207 | Chemical carcinogenesis - receptor activation | 16 | 0.030174 |
| hsa05133 | Pertussis | 8 | 0.030174 |
| hsa04621 | NOD-like receptor signaling pathway | 14 | 0.03699 |
| hsa05143 | African trypanosomiasis | 5 | 0.042151 |
| hsa05226 | Gastric cancer | 12 | 0.042691 |
| hsa05330 | Allograft rejection | 5 | 0.044697 |
| hsa05340 | Primary immunodeficiency | 5 | 0.044697 |
