# Supplementary material for: A Prognostic Risk Score Based on Hypoxia-, Immunity-, and Epithelialto-Mesenchymal Transition-Related Genes for the Prognosis and Immunotherapy Response of Lung Adenocarcinoma
Source: Front Cell Dev Biol. 2022 Jan 24;9:758777. doi: 10.3389/fcell.2021.758777 (PMC8819669; doi:10.3389/fcell.2021.758777)
Supplement: Supplementary file 8 [file Table3.DOCX]

| **Supplementary Table 3 \| KEGG pathway enrichment analysis of hypoxia-DEGs** | | | |
| --- | --- | --- | --- |
| ID | Description | Count | qvalue |
| hsa00010 | Glycolysis / Gluconeogenesis | 11 | 1.16E-11 |
| hsa04066 | HIF-1 signaling pathway | 10 | 3.39E-08 |
| hsa01200 | Carbon metabolism | 9 | 7.47E-07 |
| hsa01230 | Biosynthesis of amino acids | 7 | 7.42E-06 |
| hsa05418 | Fluid shear stress and atherosclerosis | 7 | 0.000383746 |
| hsa00030 | Pentose phosphate pathway | 4 | 0.000552555 |
| hsa00051 | Fructose and mannose metabolism | 4 | 0.000698032 |
| hsa04922 | Glucagon signaling pathway | 5 | 0.006222516 |
| hsa05211 | Renal cell carcinoma | 4 | 0.009325881 |
| hsa05230 | Central carbon metabolism in cancer | 4 | 0.009325881 |
| hsa05166 | Human T-cell leukemia virus 1 infection | 6 | 0.019636863 |
| hsa04657 | IL-17 signaling pathway | 4 | 0.023300165 |
| hsa04668 | TNF signaling pathway | 4 | 0.040531769 |
| hsa05167 | Kaposi sarcoma-associated herpesvirus infection | 5 | 0.048480321 |
