# Supplementary material for: A Prognostic Risk Score Based on Hypoxia-, Immunity-, and Epithelialto-Mesenchymal Transition-Related Genes for the Prognosis and Immunotherapy Response of Lung Adenocarcinoma
Source: Front Cell Dev Biol. 2022 Jan 24;9:758777. doi: 10.3389/fcell.2021.758777 (PMC8819669; doi:10.3389/fcell.2021.758777)
Supplement: Supplementary file 9 [file Table4.DOCX]

| **Supplementary Table 4 \| GO enrichment analysis of immune-DEGs** | | | | |
| --- | --- | --- | --- | --- |
| Category | ID | Description | Count | qvalue |
| BP | GO:0006958 | complement activation, classical pathway | 88 | 3.77E-98 |
| BP | GO:0006959 | humoral immune response | 125 | 6.86E-98 |
| BP | GO:0006956 | complement activation | 93 | 3.44E-94 |
| BP | GO:0002455 | humoral immune response mediated by circulating immunoglobulin | 88 | 2.00E-93 |
| BP | GO:0002460 | adaptive immune response based on somatic recombination of immune receptors built from immunoglobulin superfamily domains | 118 | 4.11E-90 |
| BP | GO:0019724 | B cell mediated immunity | 97 | 4.74E-87 |
| BP | GO:0016064 | immunoglobulin mediated immune response | 96 | 2.59E-86 |
| BP | GO:0002449 | lymphocyte mediated immunity | 112 | 3.79E-83 |
| BP | GO:0006909 | phagocytosis | 112 | 6.90E-81 |
| BP | GO:0002377 | immunoglobulin production | 89 | 5.91E-80 |
| BP | GO:0002440 | production of molecular mediator of immune response | 101 | 2.44E-78 |
| BP | GO:0002429 | immune response-activating cell surface receptor signaling pathway | 108 | 1.25E-64 |
| BP | GO:0002757 | immune response-activating signal transduction | 108 | 1.25E-64 |
| BP | GO:0030449 | regulation of complement activation | 61 | 9.76E-62 |
| BP | GO:0006910 | phagocytosis, recognition | 54 | 2.51E-59 |
| BP | GO:0042742 | defense response to bacterium | 90 | 5.12E-59 |
| BP | GO:0002920 | regulation of humoral immune response | 62 | 5.33E-58 |
| BP | GO:0002697 | regulation of immune effector process | 100 | 2.94E-57 |
| BP | GO:0002696 | positive regulation of leukocyte activation | 94 | 3.84E-57 |
| BP | GO:0002431 | Fc receptor mediated stimulatory signaling pathway | 63 | 7.29E-57 |
| BP | GO:0038094 | Fc-gamma receptor signaling pathway | 62 | 4.09E-56 |
| BP | GO:0050867 | positive regulation of cell activation | 94 | 1.11E-55 |
| BP | GO:0050871 | positive regulation of B cell activation | 63 | 1.50E-55 |
| BP | GO:0002433 | immune response-regulating cell surface receptor signaling pathway involved in phagocytosis | 61 | 2.01E-55 |
| BP | GO:0038096 | Fc-gamma receptor signaling pathway involved in phagocytosis | 61 | 2.01E-55 |
| BP | GO:0050864 | regulation of B cell activation | 69 | 3.34E-55 |
| BP | GO:0006911 | phagocytosis, engulfment | 58 | 3.64E-54 |
| BP | GO:0051251 | positive regulation of lymphocyte activation | 86 | 1.08E-53 |
| BP | GO:0099024 | plasma membrane invagination | 58 | 5.24E-52 |
| BP | GO:0038095 | Fc-epsilon receptor signaling pathway | 62 | 1.35E-50 |
| BP | GO:0050853 | B cell receptor signaling pathway | 57 | 1.41E-50 |
| BP | GO:0010324 | membrane invagination | 58 | 2.87E-50 |
| BP | GO:0038093 | Fc receptor signaling pathway | 69 | 3.55E-48 |
| BP | GO:0006898 | receptor-mediated endocytosis | 75 | 8.04E-45 |
| BP | GO:0042113 | B cell activation | 74 | 9.14E-44 |
| BP | GO:0008037 | cell recognition | 63 | 4.50E-43 |
| BP | GO:0050851 | antigen receptor-mediated signaling pathway | 63 | 7.34E-33 |
| BP | GO:0060326 | cell chemotaxis | 61 | 4.34E-32 |
| BP | GO:0097529 | myeloid leukocyte migration | 49 | 4.67E-28 |
| BP | GO:0030595 | leukocyte chemotaxis | 47 | 3.91E-25 |
| BP | GO:0050920 | regulation of chemotaxis | 44 | 1.71E-22 |
| BP | GO:0030593 | neutrophil chemotaxis | 31 | 6.81E-22 |
| BP | GO:0097530 | granulocyte migration | 36 | 8.95E-22 |
| BP | GO:0071621 | granulocyte chemotaxis | 33 | 4.20E-21 |
| BP | GO:0070374 | positive regulation of ERK1 and ERK2 cascade | 41 | 8.12E-21 |
| BP | GO:1990266 | neutrophil migration | 32 | 1.33E-20 |
| BP | GO:0070098 | chemokine-mediated signaling pathway | 28 | 1.63E-20 |
| BP | GO:1990868 | response to chemokine | 29 | 2.03E-20 |
| BP | GO:1990869 | cellular response to chemokine | 29 | 2.03E-20 |
| BP | GO:0007187 | G protein-coupled receptor signaling pathway, coupled to cyclic nucleotide second messenger | 44 | 4.73E-20 |
| BP | GO:0007188 | adenylate cyclase-modulating G protein-coupled receptor signaling pathway | 40 | 1.34E-18 |
| BP | GO:0019932 | second-messenger-mediated signaling | 55 | 2.78E-18 |
| BP | GO:0071674 | mononuclear cell migration | 27 | 4.19E-18 |
| BP | GO:0070371 | ERK1 and ERK2 cascade | 46 | 5.72E-18 |
| BP | GO:0002548 | monocyte chemotaxis | 23 | 1.10E-17 |
| BP | GO:0050921 | positive regulation of chemotaxis | 30 | 2.68E-16 |
| BP | GO:0019730 | antimicrobial humoral response | 29 | 5.99E-16 |
| BP | GO:0001819 | positive regulation of cytokine production | 51 | 6.68E-16 |
| BP | GO:0070372 | regulation of ERK1 and ERK2 cascade | 42 | 6.84E-16 |
| BP | GO:0050727 | regulation of inflammatory response | 49 | 1.88E-15 |
| BP | GO:0050679 | positive regulation of epithelial cell proliferation | 34 | 6.24E-15 |
| BP | GO:0070661 | leukocyte proliferation | 41 | 8.61E-15 |
| BP | GO:0018108 | peptidyl-tyrosine phosphorylation | 44 | 3.69E-14 |
| BP | GO:0019935 | cyclic-nucleotide-mediated signaling | 34 | 3.84E-14 |
| BP | GO:0002685 | regulation of leukocyte migration | 33 | 4.73E-14 |
| BP | GO:0018212 | peptidyl-tyrosine modification | 44 | 4.73E-14 |
| BP | GO:0050731 | positive regulation of peptidyl-tyrosine phosphorylation | 31 | 1.88E-13 |
| BP | GO:0001938 | positive regulation of endothelial cell proliferation | 24 | 3.02E-13 |
| BP | GO:0019933 | cAMP-mediated signaling | 31 | 3.26E-13 |
| BP | GO:0032102 | negative regulation of response to external stimulus | 46 | 3.46E-13 |
| BP | GO:0032496 | response to lipopolysaccharide | 40 | 3.86E-13 |
| BP | GO:0050673 | epithelial cell proliferation | 47 | 4.16E-13 |
| BP | GO:0051047 | positive regulation of secretion | 40 | 6.83E-13 |
| BP | GO:0051897 | positive regulation of protein kinase B signaling | 29 | 6.83E-13 |
| BP | GO:0070663 | regulation of leukocyte proliferation | 33 | 1.61E-12 |
| BP | GO:0050729 | positive regulation of inflammatory response | 27 | 1.61E-12 |
| BP | GO:0007189 | adenylate cyclase-activating G protein-coupled receptor signaling pathway | 26 | 2.03E-12 |
| BP | GO:0008217 | regulation of blood pressure | 29 | 2.42E-12 |
| BP | GO:0002237 | response to molecule of bacterial origin | 40 | 2.93E-12 |
| BP | GO:0050730 | regulation of peptidyl-tyrosine phosphorylation | 34 | 3.92E-12 |
| BP | GO:0060389 | pathway-restricted SMAD protein phosphorylation | 18 | 4.42E-12 |
| BP | GO:0007631 | feeding behavior | 22 | 4.51E-12 |
| BP | GO:0055074 | calcium ion homeostasis | 46 | 5.06E-12 |
| BP | GO:1903532 | positive regulation of secretion by cell | 37 | 5.06E-12 |
| BP | GO:0050918 | positive chemotaxis | 18 | 7.52E-12 |
| BP | GO:0031349 | positive regulation of defense response | 41 | 7.89E-12 |
| BP | GO:0001935 | endothelial cell proliferation | 29 | 1.11E-11 |
| BP | GO:0007204 | positive regulation of cytosolic calcium ion concentration | 37 | 1.16E-11 |
| BP | GO:0048660 | regulation of smooth muscle cell proliferation | 27 | 1.34E-11 |
| BP | GO:0048659 | smooth muscle cell proliferation | 27 | 1.74E-11 |
| BP | GO:0002688 | regulation of leukocyte chemotaxis | 23 | 1.74E-11 |
| BP | GO:0050919 | negative chemotaxis | 15 | 4.28E-11 |
| BP | GO:0050886 | endocrine process | 19 | 4.34E-11 |
| BP | GO:0002687 | positive regulation of leukocyte migration | 24 | 4.36E-11 |
| BP | GO:0001667 | ameboidal-type cell migration | 45 | 4.68E-11 |
| BP | GO:0048247 | lymphocyte chemotaxis | 17 | 5.01E-11 |
| BP | GO:0051480 | regulation of cytosolic calcium ion concentration | 38 | 5.48E-11 |
| BP | GO:0001936 | regulation of endothelial cell proliferation | 27 | 5.50E-11 |
| BP | GO:0010862 | positive regulation of pathway-restricted SMAD protein phosphorylation | 15 | 5.64E-11 |
| BP | GO:0033002 | muscle cell proliferation | 31 | 6.19E-11 |
| BP | GO:0050922 | negative regulation of chemotaxis | 17 | 6.25E-11 |
| BP | GO:0050678 | regulation of epithelial cell proliferation | 40 | 6.63E-11 |
| BP | GO:0001906 | cell killing | 26 | 6.71E-11 |
| BP | GO:0007159 | leukocyte cell-cell adhesion | 38 | 9.28E-11 |
| BP | GO:0006874 | cellular calcium ion homeostasis | 43 | 1.04E-10 |
| BP | GO:0046651 | lymphocyte proliferation | 33 | 1.24E-10 |
| BP | GO:0048771 | tissue remodeling | 26 | 1.44E-10 |
| BP | GO:0032943 | mononuclear cell proliferation | 33 | 1.63E-10 |
| BP | GO:0002699 | positive regulation of immune effector process | 29 | 1.64E-10 |
| BP | GO:0060393 | regulation of pathway-restricted SMAD protein phosphorylation | 16 | 2.47E-10 |
| BP | GO:0048846 | axon extension involved in axon guidance | 13 | 2.73E-10 |
| BP | GO:1902284 | neuron projection extension involved in neuron projection guidance | 13 | 2.73E-10 |
| BP | GO:0072676 | lymphocyte migration | 21 | 2.77E-10 |
| BP | GO:0072503 | cellular divalent inorganic cation homeostasis | 44 | 3.17E-10 |
| BP | GO:0002690 | positive regulation of leukocyte chemotaxis | 19 | 4.34E-10 |
| BP | GO:0034341 | response to interferon-gamma | 27 | 4.34E-10 |
| BP | GO:0061844 | antimicrobial humoral immune response mediated by antimicrobial peptide | 17 | 5.03E-10 |
| BP | GO:0050670 | regulation of lymphocyte proliferation | 28 | 5.30E-10 |
| BP | GO:0032944 | regulation of mononuclear cell proliferation | 28 | 6.53E-10 |
| BP | GO:0048841 | regulation of axon extension involved in axon guidance | 12 | 6.53E-10 |
| BP | GO:0071526 | semaphorin-plexin signaling pathway | 13 | 7.91E-10 |
| BP | GO:0071346 | cellular response to interferon-gamma | 25 | 1.28E-09 |
| BP | GO:0003044 | regulation of systemic arterial blood pressure mediated by a chemical signal | 14 | 1.32E-09 |
| BP | GO:0034103 | regulation of tissue remodeling | 18 | 1.39E-09 |
| BP | GO:0032609 | interferon-gamma production | 20 | 1.41E-09 |
| BP | GO:1903037 | regulation of leukocyte cell-cell adhesion | 34 | 1.43E-09 |
| BP | GO:0007200 | phospholipase C-activating G protein-coupled receptor signaling pathway | 19 | 1.72E-09 |
| BP | GO:0009755 | hormone-mediated signaling pathway | 26 | 1.75E-09 |
| BP | GO:0043406 | positive regulation of MAP kinase activity | 30 | 1.85E-09 |
| BP | GO:0002683 | negative regulation of immune system process | 41 | 1.93E-09 |
| BP | GO:1901342 | regulation of vasculature development | 40 | 1.95E-09 |
| BP | GO:1902668 | negative regulation of axon guidance | 11 | 2.22E-09 |
| BP | GO:1902667 | regulation of axon guidance | 13 | 2.80E-09 |
| BP | GO:0003018 | vascular process in circulatory system | 25 | 2.97E-09 |
| BP | GO:0051896 | regulation of protein kinase B signaling | 29 | 2.97E-09 |
| BP | GO:0040013 | negative regulation of locomotion | 37 | 3.74E-09 |
| BP | GO:0045766 | positive regulation of angiogenesis | 26 | 3.94E-09 |
| BP | GO:1902105 | regulation of leukocyte differentiation | 31 | 3.95E-09 |
| BP | GO:0051271 | negative regulation of cellular component movement | 37 | 4.53E-09 |
| BP | GO:0031640 | killing of cells of other organism | 15 | 4.87E-09 |
| BP | GO:0045765 | regulation of angiogenesis | 37 | 5.51E-09 |
| BP | GO:0043491 | protein kinase B signaling | 30 | 6.00E-09 |
| BP | GO:0032147 | activation of protein kinase activity | 33 | 6.19E-09 |
| BP | GO:0002822 | regulation of adaptive immune response based on somatic recombination of immune receptors built from immunoglobulin superfamily domains | 22 | 6.32E-09 |
| BP | GO:0002819 | regulation of adaptive immune response | 23 | 6.77E-09 |
| BP | GO:0071356 | cellular response to tumor necrosis factor | 31 | 6.78E-09 |
| BP | GO:0050870 | positive regulation of T cell activation | 26 | 6.90E-09 |
| BP | GO:0045785 | positive regulation of cell adhesion | 38 | 7.86E-09 |
| BP | GO:0048754 | branching morphogenesis of an epithelial tube | 22 | 7.88E-09 |
| BP | GO:0035296 | regulation of tube diameter | 21 | 1.02E-08 |
| BP | GO:0097746 | regulation of blood vessel diameter | 21 | 1.02E-08 |
| BP | GO:0061138 | morphogenesis of a branching epithelium | 24 | 1.02E-08 |
| BP | GO:0001990 | regulation of systemic arterial blood pressure by hormone | 12 | 1.02E-08 |
| BP | GO:0140353 | lipid export from cell | 12 | 1.02E-08 |
| BP | GO:1903039 | positive regulation of leukocyte cell-cell adhesion | 27 | 1.06E-08 |
| BP | GO:1904018 | positive regulation of vasculature development | 27 | 1.06E-08 |
| BP | GO:0032649 | regulation of interferon-gamma production | 18 | 1.10E-08 |
| BP | GO:0035150 | regulation of tube size | 21 | 1.12E-08 |
| BP | GO:0045926 | negative regulation of growth | 28 | 1.29E-08 |
| BP | GO:0048843 | negative regulation of axon extension involved in axon guidance | 10 | 1.68E-08 |
| BP | GO:0071222 | cellular response to lipopolysaccharide | 25 | 1.73E-08 |
| BP | GO:0010631 | epithelial cell migration | 34 | 1.77E-08 |
| BP | GO:0042110 | T cell activation | 40 | 1.91E-08 |
| BP | GO:0090132 | epithelium migration | 34 | 2.16E-08 |
| BP | GO:0070665 | positive regulation of leukocyte proliferation | 21 | 2.30E-08 |
| BP | GO:0050863 | regulation of T cell activation | 32 | 2.32E-08 |
| BP | GO:0032613 | interleukin-10 production | 14 | 2.75E-08 |
| BP | GO:0048638 | regulation of developmental growth | 33 | 2.75E-08 |
| BP | GO:0090130 | tissue migration | 34 | 3.19E-08 |
| BP | GO:0034612 | response to tumor necrosis factor | 31 | 3.63E-08 |
| BP | GO:0001763 | morphogenesis of a branching structure | 24 | 4.02E-08 |
| BP | GO:0032755 | positive regulation of interleukin-6 production | 16 | 4.26E-08 |
| BP | GO:0042116 | macrophage activation | 17 | 4.66E-08 |
| BP | GO:0002285 | lymphocyte activation involved in immune response | 23 | 4.74E-08 |
| BP | GO:0022407 | regulation of cell-cell adhesion | 37 | 4.74E-08 |
| BP | GO:1903708 | positive regulation of hemopoiesis | 24 | 5.24E-08 |
| BP | GO:0048640 | negative regulation of developmental growth | 18 | 5.24E-08 |
| BP | GO:0071902 | positive regulation of protein serine/threonine kinase activity | 32 | 5.58E-08 |
| BP | GO:0060395 | SMAD protein signal transduction | 15 | 5.73E-08 |
| BP | GO:0071219 | cellular response to molecule of bacterial origin | 25 | 6.02E-08 |
| BP | GO:0048661 | positive regulation of smooth muscle cell proliferation | 17 | 6.08E-08 |
| BP | GO:1902107 | positive regulation of leukocyte differentiation | 21 | 6.10E-08 |
| BP | GO:0030308 | negative regulation of cell growth | 23 | 6.19E-08 |
| BP | GO:0043434 | response to peptide hormone | 37 | 7.34E-08 |
| BP | GO:0002793 | positive regulation of peptide secretion | 23 | 8.29E-08 |
| BP | GO:0002573 | myeloid leukocyte differentiation | 24 | 8.91E-08 |
| BP | GO:1904894 | positive regulation of receptor signaling pathway via STAT | 16 | 9.06E-08 |
| BP | GO:0022409 | positive regulation of cell-cell adhesion | 28 | 9.09E-08 |
| BP | GO:0071216 | cellular response to biotic stimulus | 26 | 1.08E-07 |
| BP | GO:0010632 | regulation of epithelial cell migration | 29 | 1.22E-07 |
| BP | GO:0003073 | regulation of systemic arterial blood pressure | 16 | 1.22E-07 |
| BP | GO:0050671 | positive regulation of lymphocyte proliferation | 19 | 1.24E-07 |
| BP | GO:0042531 | positive regulation of tyrosine phosphorylation of STAT protein | 14 | 1.25E-07 |
| BP | GO:0097755 | positive regulation of blood vessel diameter | 13 | 1.27E-07 |
| BP | GO:0043270 | positive regulation of ion transport | 28 | 1.30E-07 |
| BP | GO:0002286 | T cell activation involved in immune response | 17 | 1.36E-07 |
| BP | GO:0032946 | positive regulation of mononuclear cell proliferation | 19 | 1.37E-07 |
| BP | GO:0042129 | regulation of T cell proliferation | 21 | 1.37E-07 |
| BP | GO:0001818 | negative regulation of cytokine production | 32 | 1.40E-07 |
| BP | GO:0001558 | regulation of cell growth | 35 | 1.49E-07 |
| BP | GO:0043405 | regulation of MAP kinase activity | 31 | 1.50E-07 |
| BP | GO:0002526 | acute inflammatory response | 17 | 1.76E-07 |
| BP | GO:0060560 | developmental growth involved in morphogenesis | 25 | 1.86E-07 |
| BP | GO:0048588 | developmental cell growth | 25 | 2.02E-07 |
| BP | GO:0042509 | regulation of tyrosine phosphorylation of STAT protein | 15 | 2.03E-07 |
| BP | GO:0002702 | positive regulation of production of molecular mediator of immune response | 16 | 2.42E-07 |
| BP | GO:0007171 | activation of transmembrane receptor protein tyrosine kinase activity | 7 | 2.55E-07 |
| BP | GO:0097696 | receptor signaling pathway via STAT | 21 | 2.71E-07 |
| BP | GO:0046879 | hormone secretion | 29 | 2.85E-07 |
| BP | GO:0007260 | tyrosine phosphorylation of STAT protein | 15 | 3.21E-07 |
| BP | GO:0044060 | regulation of endocrine process | 11 | 3.29E-07 |
| BP | GO:0098801 | regulation of renal system process | 10 | 3.49E-07 |
| BP | GO:1903706 | regulation of hemopoiesis | 38 | 3.53E-07 |
| BP | GO:0001823 | mesonephros development | 16 | 3.60E-07 |
| BP | GO:0046427 | positive regulation of receptor signaling pathway via JAK-STAT | 15 | 3.66E-07 |
| BP | GO:0002825 | regulation of T-helper 1 type immune response | 9 | 3.79E-07 |
| BP | GO:0042098 | T cell proliferation | 22 | 4.15E-07 |
| BP | GO:0048762 | mesenchymal cell differentiation | 24 | 4.16E-07 |
| BP | GO:0046849 | bone remodeling | 15 | 4.20E-07 |
| BP | GO:0042554 | superoxide anion generation | 10 | 4.55E-07 |
| BP | GO:0046660 | female sex differentiation | 17 | 4.66E-07 |
| BP | GO:0001755 | neural crest cell migration | 12 | 4.71E-07 |
| BP | GO:0009914 | hormone transport | 29 | 4.97E-07 |
| BP | GO:0008361 | regulation of cell size | 21 | 5.05E-07 |
| BP | GO:0014032 | neural crest cell development | 14 | 5.33E-07 |
| BP | GO:1904892 | regulation of receptor signaling pathway via STAT | 19 | 5.97E-07 |
| BP | GO:0097305 | response to alcohol | 24 | 6.05E-07 |
| BP | GO:0050829 | defense response to Gram-negative bacterium | 14 | 6.20E-07 |
| BP | GO:0035813 | regulation of renal sodium excretion | 8 | 6.37E-07 |
| BP | GO:0016049 | cell growth | 37 | 6.55E-07 |
| BP | GO:0060485 | mesenchyme development | 27 | 6.70E-07 |
| BP | GO:0002831 | regulation of response to biotic stimulus | 33 | 7.37E-07 |
| BP | GO:0019731 | antibacterial humoral response | 12 | 8.34E-07 |
| BP | GO:0090100 | positive regulation of transmembrane receptor protein serine/threonine kinase signaling pathway | 16 | 8.54E-07 |
| BP | GO:1905952 | regulation of lipid localization | 20 | 8.83E-07 |
| BP | GO:0035710 | CD4-positive, alpha-beta T cell activation | 15 | 9.37E-07 |
| BP | GO:0042100 | B cell proliferation | 15 | 9.37E-07 |
| BP | GO:0061387 | regulation of extent of cell growth | 16 | 9.49E-07 |
| BP | GO:0014031 | mesenchymal cell development | 14 | 9.49E-07 |
| BP | GO:0048864 | stem cell development | 14 | 9.49E-07 |
| BP | GO:0007190 | activation of adenylate cyclase activity | 10 | 9.62E-07 |
| BP | GO:0032653 | regulation of interleukin-10 production | 12 | 9.78E-07 |
| BP | GO:0001657 | ureteric bud development | 15 | 1.05E-06 |
| BP | GO:0110110 | positive regulation of animal organ morphogenesis | 14 | 1.09E-06 |
| BP | GO:0046425 | regulation of receptor signaling pathway via JAK-STAT | 18 | 1.10E-06 |
| BP | GO:0001894 | tissue homeostasis | 25 | 1.13E-06 |
| BP | GO:0046850 | regulation of bone remodeling | 11 | 1.17E-06 |
| BP | GO:0072163 | mesonephric epithelium development | 15 | 1.18E-06 |
| BP | GO:0072164 | mesonephric tubule development | 15 | 1.18E-06 |
| BP | GO:0050777 | negative regulation of immune response | 19 | 1.24E-06 |
| BP | GO:0035812 | renal sodium excretion | 8 | 1.33E-06 |
| BP | GO:0010634 | positive regulation of epithelial cell migration | 20 | 1.33E-06 |
| BP | GO:0002700 | regulation of production of molecular mediator of immune response | 18 | 1.33E-06 |
| BP | GO:0002703 | regulation of leukocyte mediated immunity | 22 | 1.46E-06 |
| BP | GO:0042102 | positive regulation of T cell proliferation | 15 | 1.51E-06 |
| BP | GO:0034694 | response to prostaglandin | 9 | 1.69E-06 |
| BP | GO:0043542 | endothelial cell migration | 26 | 1.70E-06 |
| BP | GO:1903557 | positive regulation of tumor necrosis factor superfamily cytokine production | 11 | 1.73E-06 |
| BP | GO:0014033 | neural crest cell differentiation | 14 | 1.86E-06 |
| BP | GO:0044062 | regulation of excretion | 8 | 1.88E-06 |
| BP | GO:0007259 | receptor signaling pathway via JAK-STAT | 19 | 1.95E-06 |
| BP | GO:0051024 | positive regulation of immunoglobulin secretion | 6 | 2.00E-06 |
| BP | GO:0002367 | cytokine production involved in immune response | 15 | 2.17E-06 |
| BP | GO:0009615 | response to virus | 29 | 2.22E-06 |
| BP | GO:0030517 | negative regulation of axon extension | 10 | 2.38E-06 |
| BP | GO:0002824 | positive regulation of adaptive immune response based on somatic recombination of immune receptors built from immunoglobulin superfamily domains | 15 | 2.43E-06 |
| BP | GO:0046545 | development of primary female sexual characteristics | 15 | 2.43E-06 |
| BP | GO:0006968 | cellular defense response | 11 | 2.50E-06 |
| BP | GO:0032368 | regulation of lipid transport | 17 | 2.50E-06 |
| BP | GO:0042088 | T-helper 1 type immune response | 10 | 2.95E-06 |
| BP | GO:0032735 | positive regulation of interleukin-12 production | 9 | 3.72E-06 |
| BP | GO:0000187 | activation of MAPK activity | 18 | 4.17E-06 |
| BP | GO:0002821 | positive regulation of adaptive immune response | 15 | 4.42E-06 |
| BP | GO:0002021 | response to dietary excess | 8 | 4.99E-06 |
| BP | GO:0045672 | positive regulation of osteoclast differentiation | 8 | 4.99E-06 |
| BP | GO:0050771 | negative regulation of axonogenesis | 12 | 5.05E-06 |
| BP | GO:0002763 | positive regulation of myeloid leukocyte differentiation | 11 | 5.21E-06 |
| BP | GO:0032675 | regulation of interleukin-6 production | 18 | 5.43E-06 |
| BP | GO:0006953 | acute-phase response | 10 | 5.51E-06 |
| BP | GO:0060986 | endocrine hormone secretion | 10 | 5.51E-06 |
| BP | GO:0070555 | response to interleukin-1 | 21 | 5.51E-06 |
| BP | GO:0046883 | regulation of hormone secretion | 24 | 5.69E-06 |
| BP | GO:0002292 | T cell differentiation involved in immune response | 12 | 5.78E-06 |
| BP | GO:1903555 | regulation of tumor necrosis factor superfamily cytokine production | 15 | 6.05E-06 |
| BP | GO:0045088 | regulation of innate immune response | 26 | 6.07E-06 |
| BP | GO:0030099 | myeloid cell differentiation | 32 | 6.13E-06 |
| BP | GO:0030098 | lymphocyte differentiation | 29 | 6.15E-06 |
| BP | GO:0050830 | defense response to Gram-positive bacterium | 14 | 6.44E-06 |
| BP | GO:0001503 | ossification | 31 | 6.83E-06 |
| BP | GO:0032303 | regulation of icosanoid secretion | 7 | 6.93E-06 |
| BP | GO:0030336 | negative regulation of cell migration | 28 | 7.00E-06 |
| BP | GO:0061098 | positive regulation of protein tyrosine kinase activity | 11 | 7.07E-06 |
| BP | GO:0008585 | female gonad development | 14 | 7.17E-06 |
| BP | GO:0032733 | positive regulation of interleukin-10 production | 9 | 7.35E-06 |
| BP | GO:0046887 | positive regulation of hormone secretion | 16 | 7.80E-06 |
| BP | GO:0002686 | negative regulation of leukocyte migration | 10 | 7.86E-06 |
| BP | GO:0045619 | regulation of lymphocyte differentiation | 19 | 7.97E-06 |
| BP | GO:0045639 | positive regulation of myeloid cell differentiation | 14 | 7.97E-06 |
| BP | GO:0032660 | regulation of interleukin-17 production | 9 | 9.17E-06 |
| BP | GO:0071347 | cellular response to interleukin-1 | 19 | 9.34E-06 |
| BP | GO:0001774 | microglial cell activation | 10 | 9.36E-06 |
| BP | GO:0002269 | leukocyte activation involved in inflammatory response | 10 | 9.36E-06 |
| BP | GO:0032760 | positive regulation of tumor necrosis factor production | 10 | 9.36E-06 |
| BP | GO:0002294 | CD4-positive, alpha-beta T cell differentiation involved in immune response | 11 | 9.57E-06 |
| BP | GO:0031100 | animal organ regeneration | 12 | 9.88E-06 |
| BP | GO:0035815 | positive regulation of renal sodium excretion | 6 | 1.04E-05 |
| BP | GO:0043312 | neutrophil degranulation | 34 | 1.04E-05 |
| BP | GO:0071706 | tumor necrosis factor superfamily cytokine production | 15 | 1.09E-05 |
| BP | GO:0002456 | T cell mediated immunity | 14 | 1.10E-05 |
| BP | GO:0002287 | alpha-beta T cell activation involved in immune response | 11 | 1.10E-05 |
| BP | GO:0002293 | alpha-beta T cell differentiation involved in immune response | 11 | 1.10E-05 |
| BP | GO:2000514 | regulation of CD4-positive, alpha-beta T cell activation | 11 | 1.10E-05 |
| BP | GO:0071675 | regulation of mononuclear cell migration | 10 | 1.10E-05 |
| BP | GO:0050770 | regulation of axonogenesis | 19 | 1.14E-05 |
| BP | GO:0002283 | neutrophil activation involved in immune response | 34 | 1.16E-05 |
| BP | GO:0032535 | regulation of cellular component size | 29 | 1.26E-05 |
| BP | GO:0032635 | interleukin-6 production | 18 | 1.27E-05 |
| BP | GO:0045453 | bone resorption | 11 | 1.27E-05 |
| BP | GO:0002705 | positive regulation of leukocyte mediated immunity | 16 | 1.45E-05 |
| BP | GO:0031341 | regulation of cell killing | 13 | 1.45E-05 |
| BP | GO:2000146 | negative regulation of cell motility | 28 | 1.45E-05 |
| BP | GO:0007588 | excretion | 11 | 1.47E-05 |
| BP | GO:0032729 | positive regulation of interferon-gamma production | 11 | 1.47E-05 |
| BP | GO:1990138 | neuron projection extension | 18 | 1.48E-05 |
| BP | GO:0002761 | regulation of myeloid leukocyte differentiation | 15 | 1.58E-05 |
| BP | GO:0048675 | axon extension | 15 | 1.58E-05 |
| BP | GO:0048246 | macrophage chemotaxis | 9 | 1.64E-05 |
| BP | GO:0071887 | leukocyte apoptotic process | 14 | 1.65E-05 |
| BP | GO:0001659 | temperature homeostasis | 18 | 1.72E-05 |
| BP | GO:0009620 | response to fungus | 10 | 1.81E-05 |
| BP | GO:0003014 | renal system process | 15 | 1.91E-05 |
| BP | GO:1901215 | negative regulation of neuron death | 20 | 1.99E-05 |
| BP | GO:0032680 | regulation of tumor necrosis factor production | 14 | 2.03E-05 |
| BP | GO:0002720 | positive regulation of cytokine production involved in immune response | 10 | 2.12E-05 |
| BP | GO:0032655 | regulation of interleukin-12 production | 10 | 2.12E-05 |
| BP | GO:0032620 | interleukin-17 production | 9 | 2.41E-05 |
| BP | GO:0030516 | regulation of axon extension | 13 | 2.53E-05 |
| BP | GO:0050764 | regulation of phagocytosis | 13 | 2.53E-05 |
| BP | GO:0090092 | regulation of transmembrane receptor protein serine/threonine kinase signaling pathway | 22 | 2.56E-05 |
| BP | GO:0002718 | regulation of cytokine production involved in immune response | 12 | 2.68E-05 |
| BP | GO:0010771 | negative regulation of cell morphogenesis involved in differentiation | 13 | 2.82E-05 |
| BP | GO:0046631 | alpha-beta T cell activation | 16 | 2.86E-05 |
| BP | GO:0032615 | interleukin-12 production | 10 | 2.90E-05 |
| BP | GO:1905517 | macrophage migration | 10 | 2.90E-05 |
| BP | GO:0032308 | positive regulation of prostaglandin secretion | 5 | 2.99E-05 |
| BP | GO:0002791 | regulation of peptide secretion | 28 | 3.09E-05 |
| BP | GO:0010594 | regulation of endothelial cell migration | 21 | 3.12E-05 |
| BP | GO:0050927 | positive regulation of positive chemotaxis | 7 | 3.17E-05 |
| BP | GO:0051607 | defense response to virus | 22 | 3.21E-05 |
| BP | GO:1903034 | regulation of response to wounding | 18 | 3.32E-05 |
| BP | GO:0031649 | heat generation | 6 | 3.46E-05 |
| BP | GO:0032305 | positive regulation of icosanoid secretion | 6 | 3.46E-05 |
| BP | GO:0002695 | negative regulation of leukocyte activation | 18 | 3.56E-05 |
| BP | GO:0032640 | tumor necrosis factor production | 14 | 3.60E-05 |
| BP | GO:0043524 | negative regulation of neuron apoptotic process | 16 | 3.60E-05 |
| BP | GO:0070664 | negative regulation of leukocyte proliferation | 12 | 3.74E-05 |
| BP | GO:0050663 | cytokine secretion | 11 | 3.76E-05 |
| BP | GO:0010721 | negative regulation of cell development | 26 | 3.79E-05 |
| BP | GO:0022602 | ovulation cycle process | 9 | 4.01E-05 |
| BP | GO:0045776 | negative regulation of blood pressure | 9 | 4.01E-05 |
| BP | GO:0022604 | regulation of cell morphogenesis | 33 | 4.05E-05 |
| BP | GO:0003081 | regulation of systemic arterial blood pressure by renin-angiotensin | 7 | 4.06E-05 |
| BP | GO:0050926 | regulation of positive chemotaxis | 7 | 4.06E-05 |
| BP | GO:2000516 | positive regulation of CD4-positive, alpha-beta T cell activation | 8 | 4.11E-05 |
| BP | GO:0061041 | regulation of wound healing | 16 | 4.16E-05 |
| BP | GO:0032732 | positive regulation of interleukin-1 production | 10 | 4.40E-05 |
| BP | GO:0042093 | T-helper cell differentiation | 10 | 4.40E-05 |
| BP | GO:0061900 | glial cell activation | 10 | 4.40E-05 |
| BP | GO:0048511 | rhythmic process | 24 | 4.50E-05 |
| BP | GO:0014068 | positive regulation of phosphatidylinositol 3-kinase signaling | 12 | 4.59E-05 |
| BP | GO:0050866 | negative regulation of cell activation | 19 | 4.73E-05 |
| BP | GO:0032740 | positive regulation of interleukin-17 production | 6 | 4.77E-05 |
| BP | GO:0051023 | regulation of immunoglobulin secretion | 6 | 4.77E-05 |
| BP | GO:2000831 | regulation of steroid hormone secretion | 6 | 4.77E-05 |
| BP | GO:0032306 | regulation of prostaglandin secretion | 5 | 4.92E-05 |
| BP | GO:2000273 | positive regulation of signaling receptor activity | 8 | 4.95E-05 |
| BP | GO:0002706 | regulation of lymphocyte mediated immunity | 16 | 5.18E-05 |
| BP | GO:0051250 | negative regulation of lymphocyte activation | 16 | 5.18E-05 |
| BP | GO:0051048 | negative regulation of secretion | 18 | 5.20E-05 |
| BP | GO:0050714 | positive regulation of protein secretion | 17 | 5.27E-05 |
| BP | GO:0002673 | regulation of acute inflammatory response | 9 | 5.43E-05 |
| BP | GO:1990845 | adaptive thermogenesis | 16 | 5.57E-05 |
| BP | GO:0070542 | response to fatty acid | 12 | 5.62E-05 |
| BP | GO:0010769 | regulation of cell morphogenesis involved in differentiation | 24 | 5.68E-05 |
| BP | GO:0031348 | negative regulation of defense response | 22 | 5.68E-05 |
| BP | GO:0030888 | regulation of B cell proliferation | 10 | 5.68E-05 |
| BP | GO:0032689 | negative regulation of interferon-gamma production | 8 | 5.94E-05 |
| BP | GO:0007218 | neuropeptide signaling pathway | 13 | 5.97E-05 |
| BP | GO:2000379 | positive regulation of reactive oxygen species metabolic process | 13 | 5.97E-05 |
| BP | GO:0002833 | positive regulation of response to biotic stimulus | 21 | 6.35E-05 |
| BP | GO:2000191 | regulation of fatty acid transport | 7 | 6.46E-05 |
| BP | GO:0150076 | neuroinflammatory response | 11 | 6.67E-05 |
| BP | GO:0030217 | T cell differentiation | 21 | 7.11E-05 |
| BP | GO:0001909 | leukocyte mediated cytotoxicity | 13 | 7.25E-05 |
| BP | GO:0043367 | CD4-positive, alpha-beta T cell differentiation | 11 | 7.51E-05 |
| BP | GO:0050728 | negative regulation of inflammatory response | 18 | 7.65E-05 |
| BP | GO:0061097 | regulation of protein tyrosine kinase activity | 12 | 7.65E-05 |
| BP | GO:0007178 | transmembrane receptor protein serine/threonine kinase signaling pathway | 26 | 7.67E-05 |
| BP | GO:0072073 | kidney epithelium development | 15 | 7.71E-05 |
| BP | GO:0002024 | diet induced thermogenesis | 5 | 7.72E-05 |
| BP | GO:0033138 | positive regulation of peptidyl-serine phosphorylation | 13 | 7.88E-05 |
| BP | GO:0070997 | neuron death | 26 | 7.97E-05 |
| BP | GO:0071624 | positive regulation of granulocyte chemotaxis | 7 | 8.06E-05 |
| BP | GO:1905954 | positive regulation of lipid localization | 12 | 8.40E-05 |
| BP | GO:0071622 | regulation of granulocyte chemotaxis | 9 | 8.48E-05 |
| BP | GO:0032892 | positive regulation of organic acid transport | 8 | 8.48E-05 |
| BP | GO:0051961 | negative regulation of nervous system development | 24 | 8.67E-05 |
| BP | GO:2000193 | positive regulation of fatty acid transport | 6 | 8.68E-05 |
| BP | GO:0030072 | peptide hormone secretion | 21 | 8.68E-05 |
| BP | GO:1901214 | regulation of neuron death | 24 | 9.55E-05 |
| BP | GO:0007548 | sex differentiation | 22 | 0.0001 |
| BP | GO:0045670 | regulation of osteoclast differentiation | 10 | 0.000108 |
| BP | GO:0046634 | regulation of alpha-beta T cell activation | 12 | 0.000114 |
| BP | GO:0019722 | calcium-mediated signaling | 19 | 0.000115 |
| BP | GO:0035929 | steroid hormone secretion | 6 | 0.000117 |
| BP | GO:0048305 | immunoglobulin secretion | 6 | 0.000117 |
| BP | GO:0045637 | regulation of myeloid cell differentiation | 21 | 0.000121 |
| BP | GO:0007411 | axon guidance | 22 | 0.000123 |
| BP | GO:0030316 | osteoclast differentiation | 12 | 0.000124 |
| BP | GO:0097485 | neuron projection guidance | 22 | 0.000129 |
| BP | GO:0051353 | positive regulation of oxidoreductase activity | 9 | 0.000131 |
| BP | GO:0010876 | lipid localization | 29 | 0.000137 |
| BP | GO:0042698 | ovulation cycle | 10 | 0.000137 |
| BP | GO:0048662 | negative regulation of smooth muscle cell proliferation | 10 | 0.000137 |
| BP | GO:0045580 | regulation of T cell differentiation | 15 | 0.00014 |
| BP | GO:0045124 | regulation of bone resorption | 8 | 0.000141 |
| BP | GO:0070482 | response to oxygen levels | 27 | 0.000143 |
| BP | GO:0072593 | reactive oxygen species metabolic process | 22 | 0.000149 |
| BP | GO:0060688 | regulation of morphogenesis of a branching structure | 9 | 0.000149 |
| BP | GO:0031345 | negative regulation of cell projection organization | 17 | 0.00015 |
| BP | GO:0035809 | regulation of urine volume | 6 | 0.000151 |
| BP | GO:0010743 | regulation of macrophage derived foam cell differentiation | 7 | 0.000151 |
| BP | GO:0032370 | positive regulation of lipid transport | 10 | 0.000153 |
| BP | GO:1901653 | cellular response to peptide | 27 | 0.000154 |
| BP | GO:0030101 | natural killer cell activation | 11 | 0.000157 |
| BP | GO:0001541 | ovarian follicle development | 9 | 0.00017 |
| BP | GO:0032310 | prostaglandin secretion | 5 | 0.00017 |
| BP | GO:0050930 | induction of positive chemotaxis | 5 | 0.00017 |
| BP | GO:0060732 | positive regulation of inositol phosphate biosynthetic process | 5 | 0.00017 |
| BP | GO:0045834 | positive regulation of lipid metabolic process | 15 | 0.000171 |
| BP | GO:0050772 | positive regulation of axonogenesis | 11 | 0.000172 |
| BP | GO:2000106 | regulation of leukocyte apoptotic process | 11 | 0.000172 |
| BP | GO:0055024 | regulation of cardiac muscle tissue development | 12 | 0.000177 |
| BP | GO:0072001 | renal system development | 22 | 0.000177 |
| BP | GO:0043552 | positive regulation of phosphatidylinositol 3-kinase activity | 7 | 0.000181 |
| BP | GO:0090022 | regulation of neutrophil chemotaxis | 7 | 0.000181 |
| BP | GO:0010977 | negative regulation of neuron projection development | 15 | 0.000181 |
| BP | GO:1904705 | regulation of vascular associated smooth muscle cell proliferation | 11 | 0.000189 |
| BP | GO:1990874 | vascular associated smooth muscle cell proliferation | 11 | 0.000189 |
| BP | GO:0002639 | positive regulation of immunoglobulin production | 8 | 0.000189 |
| BP | GO:0034105 | positive regulation of tissue remodeling | 8 | 0.000189 |
| BP | GO:0043551 | regulation of phosphatidylinositol 3-kinase activity | 9 | 0.000189 |
| BP | GO:1903793 | positive regulation of anion transport | 9 | 0.000189 |
| BP | GO:0031667 | response to nutrient levels | 30 | 0.000189 |
| BP | GO:0072677 | eosinophil migration | 6 | 0.000189 |
| BP | GO:0050768 | negative regulation of neurogenesis | 22 | 0.000201 |
| BP | GO:0045089 | positive regulation of innate immune response | 18 | 0.000204 |
| BP | GO:0002709 | regulation of T cell mediated immunity | 10 | 0.00021 |
| BP | GO:0032612 | interleukin-1 production | 13 | 0.000213 |
| BP | GO:0002888 | positive regulation of myeloid leukocyte mediated immunity | 7 | 0.000215 |
| BP | GO:0032814 | regulation of natural killer cell activation | 7 | 0.000215 |
| BP | GO:0045621 | positive regulation of lymphocyte differentiation | 12 | 0.000227 |
| BP | GO:0006801 | superoxide metabolic process | 10 | 0.000234 |
| BP | GO:2000846 | regulation of corticosteroid hormone secretion | 5 | 0.000235 |
| BP | GO:0032753 | positive regulation of interleukin-4 production | 6 | 0.00024 |
| BP | GO:0034695 | response to prostaglandin E | 6 | 0.00024 |
| BP | GO:0019229 | regulation of vasoconstriction | 9 | 0.000242 |
| BP | GO:0002708 | positive regulation of lymphocyte mediated immunity | 12 | 0.000246 |
| BP | GO:0046632 | alpha-beta T cell differentiation | 12 | 0.000246 |
| BP | GO:0016486 | peptide hormone processing | 7 | 0.000257 |
| BP | GO:0140448 | signaling receptor ligand precursor processing | 7 | 0.000257 |
| BP | GO:0001910 | regulation of leukocyte mediated cytotoxicity | 10 | 0.000258 |
| BP | GO:0032602 | chemokine production | 10 | 0.000258 |
| BP | GO:0007162 | negative regulation of cell adhesion | 22 | 0.000262 |
| BP | GO:0055017 | cardiac muscle tissue growth | 12 | 0.000266 |
| BP | GO:0031343 | positive regulation of cell killing | 9 | 0.000272 |
| BP | GO:1903522 | regulation of blood circulation | 22 | 0.000288 |
| BP | GO:1904036 | negative regulation of epithelial cell apoptotic process | 8 | 0.00029 |
| BP | GO:0009306 | protein secretion | 29 | 0.000292 |
| BP | GO:1901654 | response to ketone | 17 | 0.000296 |
| BP | GO:0006869 | lipid transport | 26 | 0.000298 |
| BP | GO:0060259 | regulation of feeding behavior | 6 | 0.000298 |
| BP | GO:0045582 | positive regulation of T cell differentiation | 11 | 0.000298 |
| BP | GO:0035592 | establishment of protein localization to extracellular region | 29 | 0.0003 |
| BP | GO:0001822 | kidney development | 21 | 0.000305 |
| BP | GO:0031099 | regeneration | 17 | 0.000311 |
| BP | GO:0010919 | regulation of inositol phosphate biosynthetic process | 5 | 0.000318 |
| BP | GO:0035930 | corticosteroid hormone secretion | 5 | 0.000318 |
| BP | GO:2000107 | negative regulation of leukocyte apoptotic process | 8 | 0.000331 |
| BP | GO:0010959 | regulation of metal ion transport | 26 | 0.000332 |
| BP | GO:0048608 | reproductive structure development | 28 | 0.000339 |
| BP | GO:0042063 | gliogenesis | 22 | 0.000339 |
| BP | GO:0046635 | positive regulation of alpha-beta T cell activation | 9 | 0.000341 |
| BP | GO:0042310 | vasoconstriction | 10 | 0.000348 |
| BP | GO:0001655 | urogenital system development | 23 | 0.000353 |
| BP | GO:0045730 | respiratory burst | 7 | 0.000355 |
| BP | GO:0090218 | positive regulation of lipid kinase activity | 7 | 0.000355 |
| BP | GO:0032652 | regulation of interleukin-1 production | 12 | 0.000365 |
| BP | GO:0090023 | positive regulation of neutrophil chemotaxis | 6 | 0.000365 |
| BP | GO:0090025 | regulation of monocyte chemotaxis | 6 | 0.000365 |
| BP | GO:0060562 | epithelial tube morphogenesis | 23 | 0.000365 |
| BP | GO:0051402 | neuron apoptotic process | 19 | 0.000367 |
| BP | GO:1904707 | positive regulation of vascular associated smooth muscle cell proliferation | 8 | 0.000373 |
| BP | GO:0071692 | protein localization to extracellular region | 29 | 0.000376 |
| BP | GO:0002437 | inflammatory response to antigenic stimulus | 9 | 0.000377 |
| BP | GO:0045665 | negative regulation of neuron differentiation | 18 | 0.000377 |
| BP | GO:0061458 | reproductive system development | 28 | 0.000383 |
| BP | GO:0044706 | multi-multicellular organism process | 18 | 0.000398 |
| BP | GO:0010742 | macrophage derived foam cell differentiation | 7 | 0.000413 |
| BP | GO:0090077 | foam cell differentiation | 7 | 0.000413 |
| BP | GO:0015732 | prostaglandin transport | 5 | 0.00042 |
| BP | GO:1902893 | regulation of pri-miRNA transcription by RNA polymerase II | 8 | 0.000424 |
| BP | GO:0001666 | response to hypoxia | 24 | 0.000457 |
| BP | GO:0045137 | development of primary sexual characteristics | 18 | 0.000465 |
| BP | GO:0010595 | positive regulation of endothelial cell migration | 13 | 0.00047 |
| BP | GO:0055078 | sodium ion homeostasis | 8 | 0.000485 |
| BP | GO:0010001 | glial cell differentiation | 18 | 0.000489 |
| BP | GO:0060419 | heart growth | 12 | 0.000496 |
| BP | GO:0019216 | regulation of lipid metabolic process | 27 | 0.000501 |
| BP | GO:0001649 | osteoblast differentiation | 18 | 0.000514 |
| BP | GO:0032890 | regulation of organic acid transport | 9 | 0.000525 |
| BP | GO:0043550 | regulation of lipid kinase activity | 9 | 0.000525 |
| BP | GO:0045123 | cellular extravasation | 9 | 0.000525 |
| BP | GO:0035265 | organ growth | 16 | 0.00053 |
| BP | GO:0032958 | inositol phosphate biosynthetic process | 6 | 0.000541 |
| BP | GO:1902932 | positive regulation of alcohol biosynthetic process | 6 | 0.000541 |
| BP | GO:0032731 | positive regulation of interleukin-1 beta production | 8 | 0.000545 |
| BP | GO:0061614 | pri-miRNA transcription by RNA polymerase II | 8 | 0.000545 |
| BP | GO:0032656 | regulation of interleukin-13 production | 5 | 0.000548 |
| BP | GO:0090190 | positive regulation of branching involved in ureteric bud morphogenesis | 5 | 0.000548 |
| BP | GO:0030278 | regulation of ossification | 17 | 0.00055 |
| BP | GO:0048708 | astrocyte differentiation | 10 | 0.00055 |
| BP | GO:1902895 | positive regulation of pri-miRNA transcription by RNA polymerase II | 7 | 0.000557 |
| BP | GO:0002524 | hypersensitivity | 4 | 0.000564 |
| BP | GO:0042756 | drinking behavior | 4 | 0.000564 |
| BP | GO:0030282 | bone mineralization | 12 | 0.000565 |
| BP | GO:1904019 | epithelial cell apoptotic process | 12 | 0.000565 |
| BP | GO:0032642 | regulation of chemokine production | 9 | 0.000573 |
| BP | GO:0050766 | positive regulation of phagocytosis | 9 | 0.000573 |
| BP | GO:0043523 | regulation of neuron apoptotic process | 17 | 0.000607 |
| BP | GO:0032757 | positive regulation of interleukin-8 production | 8 | 0.000609 |
| BP | GO:0071695 | anatomical structure maturation | 18 | 0.000613 |
| BP | GO:0015908 | fatty acid transport | 11 | 0.000623 |
| BP | GO:0060191 | regulation of lipase activity | 11 | 0.000623 |
| BP | GO:0048286 | lung alveolus development | 7 | 0.00064 |
| BP | GO:0050832 | defense response to fungus | 7 | 0.00064 |
| BP | GO:0002040 | sprouting angiogenesis | 16 | 0.000685 |
| BP | GO:0007565 | female pregnancy | 16 | 0.000685 |
| BP | GO:0032674 | regulation of interleukin-5 production | 5 | 0.000698 |
| BP | GO:0033189 | response to vitamin A | 5 | 0.000698 |
| BP | GO:0048245 | eosinophil chemotaxis | 5 | 0.000698 |
| BP | GO:0061081 | positive regulation of myeloid leukocyte cytokine production involved in immune response | 5 | 0.000698 |
| BP | GO:0036293 | response to decreased oxygen levels | 24 | 0.000699 |
| BP | GO:0006879 | cellular iron ion homeostasis | 9 | 0.000699 |
| BP | GO:0050795 | regulation of behavior | 9 | 0.000699 |
| BP | GO:0007193 | adenylate cyclase-inhibiting G protein-coupled receptor signaling pathway | 10 | 0.00071 |
| BP | GO:0097756 | negative regulation of blood vessel diameter | 10 | 0.00071 |
| BP | GO:0030198 | extracellular matrix organization | 25 | 0.000714 |
| BP | GO:1902622 | regulation of neutrophil migration | 7 | 0.000732 |
| BP | GO:0043062 | extracellular structure organization | 25 | 0.00074 |
| BP | GO:0043372 | positive regulation of CD4-positive, alpha-beta T cell differentiation | 6 | 0.000761 |
| BP | GO:0061082 | myeloid leukocyte cytokine production | 6 | 0.000761 |
| BP | GO:0031295 | T cell costimulation | 8 | 0.000761 |
| BP | GO:0045471 | response to ethanol | 12 | 0.000803 |
| BP | GO:2000318 | positive regulation of T-helper 17 type immune response | 4 | 0.000821 |
| BP | GO:0071375 | cellular response to peptide hormone stimulus | 22 | 0.000838 |
| BP | GO:0071398 | cellular response to fatty acid | 8 | 0.000858 |
| BP | GO:0034767 | positive regulation of ion transmembrane transport | 14 | 0.000875 |
| BP | GO:0002827 | positive regulation of T-helper 1 type immune response | 5 | 0.000875 |
| BP | GO:0032634 | interleukin-5 production | 5 | 0.000875 |
| BP | GO:0043950 | positive regulation of cAMP-mediated signaling | 5 | 0.000875 |
| BP | GO:0051767 | nitric-oxide synthase biosynthetic process | 5 | 0.000875 |
| BP | GO:0051769 | regulation of nitric-oxide synthase biosynthetic process | 5 | 0.000875 |
| BP | GO:0002675 | positive regulation of acute inflammatory response | 6 | 0.000901 |
| BP | GO:1902624 | positive regulation of neutrophil migration | 6 | 0.000901 |
| BP | GO:0019233 | sensory perception of pain | 11 | 0.000906 |
| BP | GO:0032611 | interleukin-1 beta production | 11 | 0.000906 |
| BP | GO:0010517 | regulation of phospholipase activity | 9 | 0.000928 |
| BP | GO:0008406 | gonad development | 17 | 0.000928 |
| BP | GO:0002886 | regulation of myeloid leukocyte mediated immunity | 8 | 0.000947 |
| BP | GO:0031294 | lymphocyte costimulation | 8 | 0.000947 |
| BP | GO:0001913 | T cell mediated cytotoxicity | 7 | 0.000951 |
| BP | GO:0106106 | cold-induced thermogenesis | 13 | 0.000997 |
| BP | GO:0120161 | regulation of cold-induced thermogenesis | 13 | 0.000997 |
| BP | GO:0015850 | organic hydroxy compound transport | 19 | 0.001017 |
| BP | GO:0001658 | branching involved in ureteric bud morphogenesis | 8 | 0.001051 |
| BP | GO:0043030 | regulation of macrophage activation | 8 | 0.001051 |
| BP | GO:0050707 | regulation of cytokine secretion | 8 | 0.001051 |
| BP | GO:0032673 | regulation of interleukin-4 production | 6 | 0.001051 |
| BP | GO:0035767 | endothelial cell chemotaxis | 6 | 0.001051 |
| BP | GO:0045589 | regulation of regulatory T cell differentiation | 6 | 0.001051 |
| BP | GO:0050869 | negative regulation of B cell activation | 6 | 0.001051 |
| BP | GO:0033135 | regulation of peptidyl-serine phosphorylation | 13 | 0.001052 |
| BP | GO:0001974 | blood vessel remodeling | 7 | 0.001072 |
| BP | GO:0014002 | astrocyte development | 7 | 0.001072 |
| BP | GO:0032722 | positive regulation of chemokine production | 7 | 0.001072 |
| BP | GO:0014821 | phasic smooth muscle contraction | 5 | 0.001072 |
| BP | GO:0032616 | interleukin-13 production | 5 | 0.001072 |
| BP | GO:0046641 | positive regulation of alpha-beta T cell proliferation | 5 | 0.001072 |
| BP | GO:0061213 | positive regulation of mesonephros development | 5 | 0.001072 |
| BP | GO:0051091 | positive regulation of DNA-binding transcription factor activity | 19 | 0.001088 |
| BP | GO:0014066 | regulation of phosphatidylinositol 3-kinase signaling | 12 | 0.001098 |
| BP | GO:2000278 | regulation of DNA biosynthetic process | 11 | 0.001109 |
| BP | GO:0032736 | positive regulation of interleukin-13 production | 4 | 0.001126 |
| BP | GO:0007409 | axonogenesis | 28 | 0.001133 |
| BP | GO:0051051 | negative regulation of transport | 28 | 0.00117 |
| BP | GO:1903409 | reactive oxygen species biosynthetic process | 12 | 0.001171 |
| BP | GO:1904064 | positive regulation of cation transmembrane transport | 13 | 0.001174 |
| BP | GO:0048639 | positive regulation of developmental growth | 15 | 0.001194 |
| BP | GO:0033555 | multicellular organismal response to stress | 9 | 0.001198 |
| BP | GO:1903524 | positive regulation of blood circulation | 9 | 0.001198 |
| BP | GO:0032309 | icosanoid secretion | 7 | 0.001203 |
| BP | GO:0002691 | regulation of cellular extravasation | 6 | 0.001206 |
| BP | GO:0002861 | regulation of inflammatory response to antigenic stimulus | 6 | 0.001206 |
| BP | GO:0042755 | eating behavior | 6 | 0.001206 |
| BP | GO:0046640 | regulation of alpha-beta T cell proliferation | 6 | 0.001206 |
| BP | GO:0009612 | response to mechanical stimulus | 16 | 0.001267 |
| BP | GO:0045576 | mast cell activation | 8 | 0.001271 |
| BP | GO:0033005 | positive regulation of mast cell activation | 5 | 0.001303 |
| BP | GO:0034104 | negative regulation of tissue remodeling | 5 | 0.001303 |
| BP | GO:0045624 | positive regulation of T-helper cell differentiation | 5 | 0.001303 |
| BP | GO:0055093 | response to hyperoxia | 5 | 0.001303 |
| BP | GO:0071379 | cellular response to prostaglandin stimulus | 5 | 0.001303 |
| BP | GO:0090189 | regulation of branching involved in ureteric bud morphogenesis | 5 | 0.001303 |
| BP | GO:0048738 | cardiac muscle tissue development | 17 | 0.001303 |
| BP | GO:0034764 | positive regulation of transmembrane transport | 16 | 0.001315 |
| BP | GO:0045778 | positive regulation of ossification | 10 | 0.001315 |
| BP | GO:1904035 | regulation of epithelial cell apoptotic process | 10 | 0.001315 |
| BP | GO:0051924 | regulation of calcium ion transport | 18 | 0.001315 |
| BP | GO:0046638 | positive regulation of alpha-beta T cell differentiation | 7 | 0.001341 |
| BP | GO:0010518 | positive regulation of phospholipase activity | 8 | 0.001387 |
| BP | GO:0042311 | vasodilation | 6 | 0.001387 |
| BP | GO:0050715 | positive regulation of cytokine secretion | 6 | 0.001387 |
| BP | GO:0061384 | heart trabecula morphogenesis | 6 | 0.001387 |
| BP | GO:2000352 | negative regulation of endothelial cell apoptotic process | 6 | 0.001387 |
| BP | GO:0031650 | regulation of heat generation | 4 | 0.001503 |
| BP | GO:0032725 | positive regulation of granulocyte macrophage colony-stimulating factor production | 4 | 0.001503 |
| BP | GO:0033605 | positive regulation of catecholamine secretion | 4 | 0.001503 |
| BP | GO:0045741 | positive regulation of epidermal growth factor-activated receptor activity | 4 | 0.001503 |
| BP | GO:0048015 | phosphatidylinositol-mediated signaling | 15 | 0.001503 |
| BP | GO:0014911 | positive regulation of smooth muscle cell migration | 7 | 0.001503 |
| BP | GO:0043370 | regulation of CD4-positive, alpha-beta T cell differentiation | 7 | 0.001503 |
| BP | GO:0006809 | nitric oxide biosynthetic process | 9 | 0.001525 |
| BP | GO:0050672 | negative regulation of lymphocyte proliferation | 9 | 0.001525 |
| BP | GO:0002224 | toll-like receptor signaling pathway | 13 | 0.001534 |
| BP | GO:0032693 | negative regulation of interleukin-10 production | 5 | 0.001572 |
| BP | GO:0001914 | regulation of T cell mediated cytotoxicity | 6 | 0.001599 |
| BP | GO:0032633 | interleukin-4 production | 6 | 0.001599 |
| BP | GO:0045066 | regulatory T cell differentiation | 6 | 0.001599 |
| BP | GO:0016202 | regulation of striated muscle tissue development | 13 | 0.001619 |
| BP | GO:0071774 | response to fibroblast growth factor | 13 | 0.001619 |
| BP | GO:0032651 | regulation of interleukin-1 beta production | 10 | 0.001635 |
| BP | GO:0032945 | negative regulation of mononuclear cell proliferation | 9 | 0.00165 |
| BP | GO:0055021 | regulation of cardiac muscle tissue growth | 9 | 0.00165 |
| BP | GO:0046637 | regulation of alpha-beta T cell differentiation | 8 | 0.001673 |
| BP | GO:0031279 | regulation of cyclase activity | 7 | 0.001673 |
| BP | GO:0061383 | trabecula morphogenesis | 7 | 0.001673 |
| BP | GO:0014065 | phosphatidylinositol 3-kinase signaling | 13 | 0.001706 |
| BP | GO:0050708 | regulation of protein secretion | 22 | 0.001761 |
| BP | GO:0030509 | BMP signaling pathway | 13 | 0.00181 |
| BP | GO:0048017 | inositol lipid-mediated signaling | 15 | 0.001814 |
| BP | GO:0060759 | regulation of response to cytokine stimulus | 15 | 0.001814 |
| BP | GO:0001569 | branching involved in blood vessel morphogenesis | 6 | 0.001821 |
| BP | GO:0009595 | detection of biotic stimulus | 6 | 0.001821 |
| BP | GO:0045622 | regulation of T-helper cell differentiation | 6 | 0.001821 |
| BP | GO:0045742 | positive regulation of epidermal growth factor receptor signaling pathway | 6 | 0.001821 |
| BP | GO:0046633 | alpha-beta T cell proliferation | 6 | 0.001821 |
| BP | GO:1905332 | positive regulation of morphogenesis of an epithelium | 6 | 0.001821 |
| BP | GO:0060675 | ureteric bud morphogenesis | 8 | 0.001825 |
| BP | GO:0051341 | regulation of oxidoreductase activity | 11 | 0.001846 |
| BP | GO:0002711 | positive regulation of T cell mediated immunity | 7 | 0.001848 |
| BP | GO:0071715 | icosanoid transport | 7 | 0.001848 |
| BP | GO:1901571 | fatty acid derivative transport | 7 | 0.001848 |
| BP | GO:1903727 | positive regulation of phospholipid metabolic process | 7 | 0.001848 |
| BP | GO:0002438 | acute inflammatory response to antigenic stimulus | 5 | 0.001848 |
| BP | GO:0003094 | glomerular filtration | 5 | 0.001848 |
| BP | GO:0010869 | regulation of receptor biosynthetic process | 5 | 0.001848 |
| BP | GO:0098581 | detection of external biotic stimulus | 5 | 0.001848 |
| BP | GO:1901861 | regulation of muscle tissue development | 13 | 0.001871 |
| BP | GO:0022617 | extracellular matrix disassembly | 9 | 0.001906 |
| BP | GO:0030193 | regulation of blood coagulation | 9 | 0.001906 |
| BP | GO:0035810 | positive regulation of urine volume | 4 | 0.001928 |
| BP | GO:0010770 | positive regulation of cell morphogenesis involved in differentiation | 13 | 0.001974 |
| BP | GO:0048634 | regulation of muscle organ development | 13 | 0.001974 |
| BP | GO:0072171 | mesonephric tubule morphogenesis | 8 | 0.001979 |
| BP | GO:0021700 | developmental maturation | 19 | 0.002038 |
| BP | GO:0090287 | regulation of cellular response to growth factor stimulus | 20 | 0.002038 |
| BP | GO:0048146 | positive regulation of fibroblast proliferation | 7 | 0.002051 |
| BP | GO:0046330 | positive regulation of JNK cascade | 12 | 0.002055 |
| BP | GO:0048145 | regulation of fibroblast proliferation | 9 | 0.002055 |
| BP | GO:1900046 | regulation of hemostasis | 9 | 0.002055 |
| BP | GO:0051090 | regulation of DNA-binding transcription factor activity | 26 | 0.002083 |
| BP | GO:0042445 | hormone metabolic process | 16 | 0.002093 |
| BP | GO:2000377 | regulation of reactive oxygen species metabolic process | 15 | 0.002139 |
| BP | GO:0072678 | T cell migration | 8 | 0.002161 |
| BP | GO:0051092 | positive regulation of NF-kappaB transcription factor activity | 13 | 0.002177 |
| BP | GO:0002092 | positive regulation of receptor internalization | 5 | 0.002177 |
| BP | GO:0003071 | renal system process involved in regulation of systemic arterial blood pressure | 5 | 0.002177 |
| BP | GO:0014829 | vascular associated smooth muscle contraction | 5 | 0.002177 |
| BP | GO:0061217 | regulation of mesonephros development | 5 | 0.002177 |
| BP | GO:0071677 | positive regulation of mononuclear cell migration | 5 | 0.002177 |
| BP | GO:0097205 | renal filtration | 5 | 0.002177 |
| BP | GO:2001026 | regulation of endothelial cell chemotaxis | 5 | 0.002177 |
| BP | GO:0015844 | monoamine transport | 9 | 0.002198 |
| BP | GO:0046209 | nitric oxide metabolic process | 9 | 0.002198 |
| BP | GO:0048144 | fibroblast proliferation | 9 | 0.002198 |
| BP | GO:0001912 | positive regulation of leukocyte mediated cytotoxicity | 7 | 0.002248 |
| BP | GO:0051339 | regulation of lyase activity | 7 | 0.002248 |
| BP | GO:0051930 | regulation of sensory perception of pain | 6 | 0.002309 |
| BP | GO:0051954 | positive regulation of amine transport | 6 | 0.002309 |
| BP | GO:1901186 | positive regulation of ERBB signaling pathway | 6 | 0.002309 |
| BP | GO:2000403 | positive regulation of lymphocyte migration | 6 | 0.002309 |
| BP | GO:0002637 | regulation of immunoglobulin production | 8 | 0.002326 |
| BP | GO:0072577 | endothelial cell apoptotic process | 8 | 0.002326 |
| BP | GO:0007586 | digestion | 12 | 0.002403 |
| BP | GO:0090288 | negative regulation of cellular response to growth factor stimulus | 14 | 0.002435 |
| BP | GO:0002281 | macrophage activation involved in immune response | 4 | 0.002435 |
| BP | GO:0002864 | regulation of acute inflammatory response to antigenic stimulus | 4 | 0.002435 |
| BP | GO:0032645 | regulation of granulocyte macrophage colony-stimulating factor production | 4 | 0.002435 |
| BP | GO:0042976 | activation of Janus kinase activity | 4 | 0.002435 |
| BP | GO:0051928 | positive regulation of calcium ion transport | 11 | 0.002447 |
| BP | GO:0014910 | regulation of smooth muscle cell migration | 9 | 0.002547 |
| BP | GO:0001959 | regulation of cytokine-mediated signaling pathway | 14 | 0.002547 |
| BP | GO:0002507 | tolerance induction | 5 | 0.002547 |
| BP | GO:0021884 | forebrain neuron development | 5 | 0.002547 |
| BP | GO:0042104 | positive regulation of activated T cell proliferation | 5 | 0.002547 |
| BP | GO:0051873 | killing by host of symbiont cells | 5 | 0.002547 |
| BP | GO:0051931 | regulation of sensory perception | 6 | 0.002606 |
| BP | GO:0031214 | biomineral tissue development | 13 | 0.002637 |
| BP | GO:0110148 | biomineralization | 13 | 0.002637 |
| BP | GO:0044070 | regulation of anion transport | 10 | 0.002731 |
| BP | GO:0051781 | positive regulation of cell division | 9 | 0.002731 |
| BP | GO:0055072 | iron ion homeostasis | 9 | 0.002731 |
| BP | GO:0060420 | regulation of heart growth | 9 | 0.002731 |
| BP | GO:2001057 | reactive nitrogen species metabolic process | 9 | 0.002731 |
| BP | GO:1901343 | negative regulation of vasculature development | 15 | 0.002879 |
| BP | GO:0090184 | positive regulation of kidney development | 6 | 0.002956 |
| BP | GO:0050818 | regulation of coagulation | 9 | 0.002956 |
| BP | GO:0003148 | outflow tract septum morphogenesis | 5 | 0.002996 |
| BP | GO:0048143 | astrocyte activation | 5 | 0.002996 |
| BP | GO:1901623 | regulation of lymphocyte chemotaxis | 5 | 0.002996 |
| BP | GO:0050433 | regulation of catecholamine secretion | 7 | 0.003027 |
| BP | GO:0060249 | anatomical structure homeostasis | 26 | 0.003029 |
| BP | GO:0048545 | response to steroid hormone | 21 | 0.003041 |
| BP | GO:1903531 | negative regulation of secretion by cell | 13 | 0.003048 |
| BP | GO:0032604 | granulocyte macrophage colony-stimulating factor production | 4 | 0.003048 |
| BP | GO:0033033 | negative regulation of myeloid cell apoptotic process | 4 | 0.003048 |
| BP | GO:0034374 | low-density lipoprotein particle remodeling | 4 | 0.003048 |
| BP | GO:0045342 | MHC class II biosynthetic process | 4 | 0.003048 |
| BP | GO:0051770 | positive regulation of nitric-oxide synthase biosynthetic process | 4 | 0.003048 |
| BP | GO:0044344 | cellular response to fibroblast growth factor stimulus | 12 | 0.003116 |
| BP | GO:2000027 | regulation of animal organ morphogenesis | 17 | 0.003183 |
| BP | GO:0030890 | positive regulation of B cell proliferation | 6 | 0.003311 |
| BP | GO:0090183 | regulation of kidney development | 7 | 0.003323 |
| BP | GO:0043534 | blood vessel endothelial cell migration | 14 | 0.003353 |
| BP | GO:0071772 | response to BMP | 13 | 0.003368 |
| BP | GO:0071773 | cellular response to BMP stimulus | 13 | 0.003368 |
| BP | GO:0050890 | cognition | 19 | 0.003403 |
| BP | GO:0048872 | homeostasis of number of cells | 17 | 0.003438 |
| BP | GO:0032800 | receptor biosynthetic process | 5 | 0.00347 |
| BP | GO:0051883 | killing of cells in other organism involved in symbiotic interaction | 5 | 0.00347 |
| BP | GO:0070227 | lymphocyte apoptotic process | 8 | 0.003503 |
| BP | GO:0072078 | nephron tubule morphogenesis | 8 | 0.003503 |
| BP | GO:0006939 | smooth muscle contraction | 10 | 0.003508 |
| BP | GO:0097306 | cellular response to alcohol | 9 | 0.003632 |
| BP | GO:0048016 | inositol phosphate-mediated signaling | 7 | 0.003635 |
| BP | GO:0050432 | catecholamine secretion | 7 | 0.003635 |
| BP | GO:0032874 | positive regulation of stress-activated MAPK cascade | 13 | 0.003702 |
| BP | GO:0030889 | negative regulation of B cell proliferation | 4 | 0.003801 |
| BP | GO:0007611 | learning or memory | 17 | 0.004014 |
| BP | GO:0002043 | blood vessel endothelial cell proliferation involved in sprouting angiogenesis | 7 | 0.004014 |
| BP | GO:0019835 | cytolysis | 5 | 0.004028 |
| BP | GO:0036296 | response to increased oxygen levels | 5 | 0.004028 |
| BP | GO:0043032 | positive regulation of macrophage activation | 5 | 0.004028 |
| BP | GO:0070304 | positive regulation of stress-activated protein kinase signaling cascade | 13 | 0.004083 |
| BP | GO:0072088 | nephron epithelium morphogenesis | 8 | 0.004117 |
| BP | GO:0002369 | T cell cytokine production | 6 | 0.004144 |
| BP | GO:0045823 | positive regulation of heart contraction | 6 | 0.004144 |
| BP | GO:0014909 | smooth muscle cell migration | 9 | 0.004156 |
| BP | GO:0032677 | regulation of interleukin-8 production | 9 | 0.004156 |
| BP | GO:1903035 | negative regulation of response to wounding | 9 | 0.004156 |
| BP | GO:0033209 | tumor necrosis factor-mediated signaling pathway | 13 | 0.004266 |
| BP | GO:0061448 | connective tissue development | 17 | 0.004291 |
| BP | GO:0060193 | positive regulation of lipase activity | 8 | 0.004444 |
| BP | GO:0060993 | kidney morphogenesis | 9 | 0.004456 |
| BP | GO:0018105 | peptidyl-serine phosphorylation | 19 | 0.004456 |
| BP | GO:0010469 | regulation of signaling receptor activity | 13 | 0.004461 |
| BP | GO:0015718 | monocarboxylic acid transport | 13 | 0.004461 |
| BP | GO:0032963 | collagen metabolic process | 10 | 0.004478 |
| BP | GO:0032965 | regulation of collagen biosynthetic process | 6 | 0.004593 |
| BP | GO:0033003 | regulation of mast cell activation | 6 | 0.004593 |
| BP | GO:0048863 | stem cell differentiation | 17 | 0.004593 |
| BP | GO:0001516 | prostaglandin biosynthetic process | 5 | 0.004593 |
| BP | GO:0007176 | regulation of epidermal growth factor-activated receptor activity | 5 | 0.004593 |
| BP | GO:0033688 | regulation of osteoblast proliferation | 5 | 0.004593 |
| BP | GO:0046457 | prostanoid biosynthetic process | 5 | 0.004593 |
| BP | GO:0060457 | negative regulation of digestive system process | 4 | 0.004612 |
| BP | GO:0062013 | positive regulation of small molecule metabolic process | 12 | 0.004661 |
| BP | GO:0051216 | cartilage development | 14 | 0.004715 |
| BP | GO:1903725 | regulation of phospholipid metabolic process | 9 | 0.004718 |
| BP | GO:0061333 | renal tubule morphogenesis | 8 | 0.004731 |
| BP | GO:0072028 | nephron morphogenesis | 8 | 0.004731 |
| BP | GO:0043112 | receptor metabolic process | 14 | 0.004922 |
| BP | GO:0030324 | lung development | 13 | 0.005084 |
| BP | GO:0003197 | endocardial cushion development | 6 | 0.005084 |
| BP | GO:0032924 | activin receptor signaling pathway | 6 | 0.005084 |
| BP | GO:0045429 | positive regulation of nitric oxide biosynthetic process | 6 | 0.005084 |
| BP | GO:0060412 | ventricular septum morphogenesis | 6 | 0.005084 |
| BP | GO:0006027 | glycosaminoglycan catabolic process | 7 | 0.005164 |
| BP | GO:0002323 | natural killer cell activation involved in immune response | 5 | 0.005246 |
| BP | GO:0072538 | T-helper 17 type immune response | 5 | 0.005246 |
| BP | GO:0098868 | bone growth | 5 | 0.005246 |
| BP | GO:0120162 | positive regulation of cold-induced thermogenesis | 9 | 0.005375 |
| BP | GO:0001991 | regulation of systemic arterial blood pressure by circulatory renin-angiotensin | 4 | 0.005539 |
| BP | GO:0010744 | positive regulation of macrophage derived foam cell differentiation | 4 | 0.005539 |
| BP | GO:0032930 | positive regulation of superoxide anion generation | 4 | 0.005539 |
| BP | GO:0034393 | positive regulation of smooth muscle cell apoptotic process | 4 | 0.005539 |
| BP | GO:0045063 | T-helper 1 cell differentiation | 4 | 0.005539 |
| BP | GO:0045780 | positive regulation of bone resorption | 4 | 0.005539 |
| BP | GO:0046852 | positive regulation of bone remodeling | 4 | 0.005539 |
| BP | GO:0090026 | positive regulation of monocyte chemotaxis | 4 | 0.005539 |
| BP | GO:0098543 | detection of other organism | 4 | 0.005539 |
| BP | GO:0030900 | forebrain development | 22 | 0.005544 |
| BP | GO:1903038 | negative regulation of leukocyte cell-cell adhesion | 11 | 0.005547 |
| BP | GO:0055025 | positive regulation of cardiac muscle tissue development | 7 | 0.005566 |
| BP | GO:2000351 | regulation of endothelial cell apoptotic process | 7 | 0.005566 |
| BP | GO:0010863 | positive regulation of phospholipase C activity | 6 | 0.005572 |
| BP | GO:1904407 | positive regulation of nitric oxide metabolic process | 6 | 0.005572 |
| BP | GO:0048732 | gland development | 24 | 0.005753 |
| BP | GO:1901224 | positive regulation of NIK/NF-kappaB signaling | 8 | 0.005852 |
| BP | GO:0007568 | aging | 19 | 0.005852 |
| BP | GO:0045761 | regulation of adenylate cyclase activity | 5 | 0.005922 |
| BP | GO:0061036 | positive regulation of cartilage development | 5 | 0.005922 |
| BP | GO:0030323 | respiratory tube development | 13 | 0.006005 |
| BP | GO:0015837 | amine transport | 9 | 0.00602 |
| BP | GO:0043255 | regulation of carbohydrate biosynthetic process | 9 | 0.00602 |
| BP | GO:0090277 | positive regulation of peptide hormone secretion | 9 | 0.00602 |
| BP | GO:0030225 | macrophage differentiation | 6 | 0.006156 |
| BP | GO:0043303 | mast cell degranulation | 6 | 0.006156 |
| BP | GO:0045807 | positive regulation of endocytosis | 9 | 0.006431 |
| BP | GO:0045927 | positive regulation of growth | 17 | 0.00644 |
| BP | GO:2000401 | regulation of lymphocyte migration | 7 | 0.006571 |
| BP | GO:0002689 | negative regulation of leukocyte chemotaxis | 4 | 0.006571 |
| BP | GO:0003085 | negative regulation of systemic arterial blood pressure | 4 | 0.006571 |
| BP | GO:0007194 | negative regulation of adenylate cyclase activity | 4 | 0.006571 |
| BP | GO:0032816 | positive regulation of natural killer cell activation | 4 | 0.006571 |
| BP | GO:1905939 | regulation of gonad development | 4 | 0.006571 |
| BP | GO:0003338 | metanephros morphogenesis | 5 | 0.006684 |
| BP | GO:0021988 | olfactory lobe development | 5 | 0.006684 |
| BP | GO:0033028 | myeloid cell apoptotic process | 5 | 0.006684 |
| BP | GO:0071560 | cellular response to transforming growth factor beta stimulus | 16 | 0.006756 |
| BP | GO:0002279 | mast cell activation involved in immune response | 6 | 0.006759 |
| BP | GO:1900274 | regulation of phospholipase C activity | 6 | 0.006759 |
| BP | GO:0032637 | interleukin-8 production | 9 | 0.006776 |
| BP | GO:0001660 | fever generation | 3 | 0.007065 |
| BP | GO:0032815 | negative regulation of natural killer cell activation | 3 | 0.007065 |
| BP | GO:0032817 | regulation of natural killer cell proliferation | 3 | 0.007065 |
| BP | GO:0043084 | penile erection | 3 | 0.007065 |
| BP | GO:0043380 | regulation of memory T cell differentiation | 3 | 0.007065 |
| BP | GO:0045625 | regulation of T-helper 1 cell differentiation | 3 | 0.007065 |
| BP | GO:0045713 | low-density lipoprotein particle receptor biosynthetic process | 3 | 0.007065 |
| BP | GO:0022600 | digestive system process | 9 | 0.007183 |
| BP | GO:0031960 | response to corticosteroid | 12 | 0.007329 |
| BP | GO:0051147 | regulation of muscle cell differentiation | 13 | 0.007369 |
| BP | GO:0002448 | mast cell mediated immunity | 6 | 0.007402 |
| BP | GO:0010712 | regulation of collagen metabolic process | 6 | 0.007402 |
| BP | GO:0072006 | nephron development | 11 | 0.007402 |
| BP | GO:0007202 | activation of phospholipase C activity | 5 | 0.00749 |
| BP | GO:0051482 | positive regulation of cytosolic calcium ion concentration involved in phospholipase C-activating G protein-coupled signaling pathway | 5 | 0.00749 |
| BP | GO:0045913 | positive regulation of carbohydrate metabolic process | 8 | 0.007618 |
| BP | GO:0002029 | desensitization of G protein-coupled receptor signaling pathway | 4 | 0.007683 |
| BP | GO:0003177 | pulmonary valve development | 4 | 0.007683 |
| BP | GO:0022401 | negative adaptation of signaling pathway | 4 | 0.007683 |
| BP | GO:0090280 | positive regulation of calcium ion import | 4 | 0.007683 |
| BP | GO:0140131 | positive regulation of lymphocyte chemotaxis | 4 | 0.007683 |
| BP | GO:0008016 | regulation of heart contraction | 16 | 0.007683 |
| BP | GO:0002698 | negative regulation of immune effector process | 10 | 0.008038 |
| BP | GO:0010633 | negative regulation of epithelial cell migration | 10 | 0.008038 |
| BP | GO:0022612 | gland morphogenesis | 10 | 0.008038 |
| BP | GO:2000725 | regulation of cardiac muscle cell differentiation | 6 | 0.008092 |
| BP | GO:0043393 | regulation of protein binding | 14 | 0.008095 |
| BP | GO:0006026 | aminoglycan catabolic process | 7 | 0.008173 |
| BP | GO:0071559 | response to transforming growth factor beta | 16 | 0.008235 |
| BP | GO:0003203 | endocardial cushion morphogenesis | 5 | 0.008366 |
| BP | GO:0033687 | osteoblast proliferation | 5 | 0.008366 |
| BP | GO:0034390 | smooth muscle cell apoptotic process | 5 | 0.008366 |
| BP | GO:0034391 | regulation of smooth muscle cell apoptotic process | 5 | 0.008366 |
| BP | GO:0040014 | regulation of multicellular organism growth | 7 | 0.008825 |
| BP | GO:0045428 | regulation of nitric oxide biosynthetic process | 7 | 0.008825 |
| BP | GO:0018209 | peptidyl-serine modification | 19 | 0.008826 |
| BP | GO:0022408 | negative regulation of cell-cell adhesion | 13 | 0.00898 |
| BP | GO:0023058 | adaptation of signaling pathway | 4 | 0.00898 |
| BP | GO:0032700 | negative regulation of interleukin-17 production | 4 | 0.00898 |
| BP | GO:0032928 | regulation of superoxide anion generation | 4 | 0.00898 |
| BP | GO:2000316 | regulation of T-helper 17 type immune response | 4 | 0.00898 |
| BP | GO:0014812 | muscle cell migration | 9 | 0.00898 |
| BP | GO:0001787 | natural killer cell proliferation | 3 | 0.009021 |
| BP | GO:0010739 | positive regulation of protein kinase A signaling | 3 | 0.009021 |
| BP | GO:0014820 | tonic smooth muscle contraction | 3 | 0.009021 |
| BP | GO:0031652 | positive regulation of heat generation | 3 | 0.009021 |
| BP | GO:0032754 | positive regulation of interleukin-5 production | 3 | 0.009021 |
| BP | GO:0033860 | regulation of NAD(P)H oxidase activity | 3 | 0.009021 |
| BP | GO:0035747 | natural killer cell chemotaxis | 3 | 0.009021 |
| BP | GO:0040015 | negative regulation of multicellular organism growth | 3 | 0.009021 |
| BP | GO:0043379 | memory T cell differentiation | 3 | 0.009021 |
| BP | GO:0051712 | positive regulation of killing of cells of other organism | 3 | 0.009021 |
| BP | GO:0090715 | immunological memory formation process | 3 | 0.009021 |
| BP | GO:0098917 | retrograde trans-synaptic signaling | 3 | 0.009021 |
| BP | GO:0032956 | regulation of actin cytoskeleton organization | 20 | 0.009215 |
| BP | GO:0010092 | specification of animal organ identity | 5 | 0.009244 |
| BP | GO:0010543 | regulation of platelet activation | 5 | 0.009244 |
| BP | GO:0010596 | negative regulation of endothelial cell migration | 9 | 0.009399 |
| BP | GO:0003179 | heart valve morphogenesis | 6 | 0.009522 |
| BP | GO:0048260 | positive regulation of receptor-mediated endocytosis | 6 | 0.009522 |
| BP | GO:0007584 | response to nutrient | 12 | 0.009731 |
| BP | GO:0030307 | positive regulation of cell growth | 12 | 0.009731 |
| BP | GO:0002062 | chondrocyte differentiation | 9 | 0.009939 |
| BP | GO:0007229 | integrin-mediated signaling pathway | 9 | 0.009939 |
| BP | GO:0030522 | intracellular receptor signaling pathway | 16 | 0.010289 |
| BP | GO:1904994 | regulation of leukocyte adhesion to vascular endothelial cell | 5 | 0.01031 |
| BP | GO:0031280 | negative regulation of cyclase activity | 4 | 0.01031 |
| BP | GO:0035743 | CD4-positive, alpha-beta T cell cytokine production | 4 | 0.01031 |
| BP | GO:1900017 | positive regulation of cytokine production involved in inflammatory response | 4 | 0.01031 |
| BP | GO:0007623 | circadian rhythm | 14 | 0.01034 |
| BP | GO:0032964 | collagen biosynthetic process | 6 | 0.01034 |
| BP | GO:0043949 | regulation of cAMP-mediated signaling | 6 | 0.01034 |
| BP | GO:0061756 | leukocyte adhesion to vascular endothelial cell | 6 | 0.01034 |
| BP | GO:0051098 | regulation of binding | 20 | 0.011196 |
| BP | GO:0003206 | cardiac chamber morphogenesis | 10 | 0.01129 |
| BP | GO:0045667 | regulation of osteoblast differentiation | 10 | 0.01129 |
| BP | GO:0032623 | interleukin-2 production | 6 | 0.011308 |
| BP | GO:0001867 | complement activation, lectin pathway | 3 | 0.011308 |
| BP | GO:0002863 | positive regulation of inflammatory response to antigenic stimulus | 3 | 0.011308 |
| BP | GO:0030157 | pancreatic juice secretion | 3 | 0.011308 |
| BP | GO:0044650 | adhesion of symbiont to host cell | 3 | 0.011308 |
| BP | GO:0051549 | positive regulation of keratinocyte migration | 3 | 0.011308 |
| BP | GO:0060453 | regulation of gastric acid secretion | 3 | 0.011308 |
| BP | GO:0072540 | T-helper 17 cell lineage commitment | 3 | 0.011308 |
| BP | GO:1902287 | semaphorin-plexin signaling pathway involved in axon guidance | 3 | 0.011308 |
| BP | GO:1905941 | positive regulation of gonad development | 3 | 0.011308 |
| BP | GO:0003298 | physiological muscle hypertrophy | 5 | 0.011308 |
| BP | GO:0003301 | physiological cardiac muscle hypertrophy | 5 | 0.011308 |
| BP | GO:0010661 | positive regulation of muscle cell apoptotic process | 5 | 0.011308 |
| BP | GO:0030224 | monocyte differentiation | 5 | 0.011308 |
| BP | GO:0061049 | cell growth involved in cardiac muscle cell development | 5 | 0.011308 |
| BP | GO:0071276 | cellular response to cadmium ion | 5 | 0.011308 |
| BP | GO:1903131 | mononuclear cell differentiation | 5 | 0.011308 |
| BP | GO:0043647 | inositol phosphate metabolic process | 7 | 0.011401 |
| BP | GO:0051937 | catecholamine transport | 7 | 0.011401 |
| BP | GO:0061035 | regulation of cartilage development | 7 | 0.011401 |
| BP | GO:0051952 | regulation of amine transport | 8 | 0.011449 |
| BP | GO:0060333 | interferon-gamma-mediated signaling pathway | 8 | 0.011449 |
| BP | GO:0001759 | organ induction | 4 | 0.011695 |
| BP | GO:0044321 | response to leptin | 4 | 0.011695 |
| BP | GO:1903429 | regulation of cell maturation | 4 | 0.011695 |
| BP | GO:0045620 | negative regulation of lymphocyte differentiation | 6 | 0.012078 |
| BP | GO:0051145 | smooth muscle cell differentiation | 7 | 0.012222 |
| BP | GO:0010737 | protein kinase A signaling | 5 | 0.012492 |
| BP | GO:0042092 | type 2 immune response | 5 | 0.012492 |
| BP | GO:2000249 | regulation of actin cytoskeleton reorganization | 5 | 0.012492 |
| BP | GO:2000279 | negative regulation of DNA biosynthetic process | 5 | 0.012492 |
| BP | GO:0003015 | heart process | 17 | 0.01281 |
| BP | GO:0002221 | pattern recognition receptor signaling pathway | 13 | 0.012829 |
| BP | GO:0051702 | interaction with symbiont | 8 | 0.012893 |
| BP | GO:0072080 | nephron tubule development | 8 | 0.012893 |
| BP | GO:1903036 | positive regulation of response to wounding | 7 | 0.01305 |
| BP | GO:0070228 | regulation of lymphocyte apoptotic process | 6 | 0.01305 |
| BP | GO:1904645 | response to amyloid-beta | 6 | 0.01305 |
| BP | GO:0006816 | calcium ion transport | 22 | 0.013219 |
| BP | GO:0048469 | cell maturation | 12 | 0.013253 |
| BP | GO:0010738 | regulation of protein kinase A signaling | 4 | 0.013379 |
| BP | GO:0043302 | positive regulation of leukocyte degranulation | 4 | 0.013379 |
| BP | GO:1904062 | regulation of cation transmembrane transport | 19 | 0.013382 |
| BP | GO:0051235 | maintenance of location | 18 | 0.013505 |
| BP | GO:0010660 | regulation of muscle cell apoptotic process | 8 | 0.013614 |
| BP | GO:0002714 | positive regulation of B cell mediated immunity | 5 | 0.013682 |
| BP | GO:0002891 | positive regulation of immunoglobulin mediated immune response | 5 | 0.013682 |
| BP | GO:0030501 | positive regulation of bone mineralization | 5 | 0.013682 |
| BP | GO:0050691 | regulation of defense response to virus by host | 5 | 0.013682 |
| BP | GO:0072210 | metanephric nephron development | 5 | 0.013682 |
| BP | GO:0030858 | positive regulation of epithelial cell differentiation | 7 | 0.013697 |
| BP | GO:0043627 | response to estrogen | 7 | 0.013697 |
| BP | GO:0045685 | regulation of glial cell differentiation | 7 | 0.013697 |
| BP | GO:0031098 | stress-activated protein kinase signaling cascade | 17 | 0.013697 |
| BP | GO:0002430 | complement receptor mediated signaling pathway | 3 | 0.013697 |
| BP | GO:0008228 | opsonization | 3 | 0.013697 |
| BP | GO:0010745 | negative regulation of macrophage derived foam cell differentiation | 3 | 0.013697 |
| BP | GO:0060340 | positive regulation of type I interferon-mediated signaling pathway | 3 | 0.013697 |
| BP | GO:0061469 | regulation of type B pancreatic cell proliferation | 3 | 0.013697 |
| BP | GO:1900272 | negative regulation of long-term synaptic potentiation | 3 | 0.013697 |
| BP | GO:1902285 | semaphorin-plexin signaling pathway involved in neuron projection guidance | 3 | 0.013697 |
| BP | GO:1903236 | regulation of leukocyte tethering or rolling | 3 | 0.013697 |
| BP | GO:1904995 | negative regulation of leukocyte adhesion to vascular endothelial cell | 3 | 0.013697 |
| BP | GO:2000194 | regulation of female gonad development | 3 | 0.013697 |
| BP | GO:0030168 | platelet activation | 11 | 0.013813 |
| BP | GO:0045840 | positive regulation of mitotic nuclear division | 6 | 0.013832 |
| BP | GO:0060760 | positive regulation of response to cytokine stimulus | 6 | 0.013832 |
| BP | GO:0060541 | respiratory system development | 13 | 0.014 |
| BP | GO:0007589 | body fluid secretion | 8 | 0.014119 |
| BP | GO:0060337 | type I interferon signaling pathway | 8 | 0.014119 |
| BP | GO:0061326 | renal tubule development | 8 | 0.014119 |
| BP | GO:0071357 | cellular response to type I interferon | 8 | 0.014119 |
| BP | GO:0046620 | regulation of organ growth | 9 | 0.014558 |
| BP | GO:0046916 | cellular transition metal ion homeostasis | 9 | 0.014558 |
| BP | GO:0006979 | response to oxidative stress | 23 | 0.014767 |
| BP | GO:0007618 | mating | 5 | 0.014806 |
| BP | GO:0042596 | fear response | 5 | 0.014806 |
| BP | GO:2000826 | regulation of heart morphogenesis | 5 | 0.014806 |
| BP | GO:0050769 | positive regulation of neurogenesis | 24 | 0.014809 |
| BP | GO:0035902 | response to immobilization stress | 4 | 0.01481 |
| BP | GO:0051350 | negative regulation of lyase activity | 4 | 0.01481 |
| BP | GO:0060343 | trabecula formation | 4 | 0.01481 |
| BP | GO:0072202 | cell differentiation involved in metanephros development | 4 | 0.01481 |
| BP | GO:1904754 | positive regulation of vascular associated smooth muscle cell migration | 4 | 0.01481 |
| BP | GO:1905523 | positive regulation of macrophage migration | 4 | 0.01481 |
| BP | GO:0051222 | positive regulation of protein transport | 19 | 0.014878 |
| BP | GO:0045446 | endothelial cell differentiation | 9 | 0.015193 |
| BP | GO:0030500 | regulation of bone mineralization | 7 | 0.015395 |
| BP | GO:0060411 | cardiac septum morphogenesis | 7 | 0.015395 |
| BP | GO:0046328 | regulation of JNK cascade | 12 | 0.015642 |
| BP | GO:1901655 | cellular response to ketone | 8 | 0.015696 |
| BP | GO:0008543 | fibroblast growth factor receptor signaling pathway | 9 | 0.015954 |
| BP | GO:0031663 | lipopolysaccharide-mediated signaling pathway | 6 | 0.015954 |
| BP | GO:0060043 | regulation of cardiac muscle cell proliferation | 6 | 0.015954 |
| BP | GO:0044058 | regulation of digestive system process | 5 | 0.016122 |
| BP | GO:0046006 | regulation of activated T cell proliferation | 5 | 0.016122 |
| BP | GO:0090279 | regulation of calcium ion import | 5 | 0.016122 |
| BP | GO:2001257 | regulation of cation channel activity | 12 | 0.016187 |
| BP | GO:0043900 | regulation of multi-organism process | 7 | 0.016322 |
| BP | GO:0051057 | positive regulation of small GTPase mediated signal transduction | 7 | 0.016322 |
| BP | GO:0061045 | negative regulation of wound healing | 7 | 0.016322 |
| BP | GO:0010838 | positive regulation of keratinocyte proliferation | 3 | 0.016332 |
| BP | GO:0045472 | response to ether | 3 | 0.016332 |
| BP | GO:0050872 | white fat cell differentiation | 3 | 0.016332 |
| BP | GO:0051547 | regulation of keratinocyte migration | 3 | 0.016332 |
| BP | GO:0051709 | regulation of killing of cells of other organism | 3 | 0.016332 |
| BP | GO:0071285 | cellular response to lithium ion | 3 | 0.016332 |
| BP | GO:0072216 | positive regulation of metanephros development | 3 | 0.016332 |
| BP | GO:0090713 | immunological memory process | 3 | 0.016332 |
| BP | GO:0010657 | muscle cell apoptotic process | 8 | 0.016377 |
| BP | GO:0051153 | regulation of striated muscle cell differentiation | 9 | 0.016533 |
| BP | GO:0002026 | regulation of the force of heart contraction | 4 | 0.016533 |
| BP | GO:0003416 | endochondral bone growth | 4 | 0.016533 |
| BP | GO:0072539 | T-helper 17 cell differentiation | 4 | 0.016533 |
| BP | GO:1903428 | positive regulation of reactive oxygen species biosynthetic process | 6 | 0.016968 |
| BP | GO:0062197 | cellular response to chemical stress | 19 | 0.017197 |
| BP | GO:0006826 | iron ion transport | 7 | 0.017219 |
| BP | GO:0043535 | regulation of blood vessel endothelial cell migration | 11 | 0.017242 |
| BP | GO:0034340 | response to type I interferon | 8 | 0.017242 |
| BP | GO:0002823 | negative regulation of adaptive immune response based on somatic recombination of immune receptors built from immunoglobulin superfamily domains | 5 | 0.017423 |
| BP | GO:0045773 | positive regulation of axon extension | 5 | 0.017423 |
| BP | GO:0000302 | response to reactive oxygen species | 14 | 0.017465 |
| BP | GO:0045444 | fat cell differentiation | 14 | 0.017465 |
| BP | GO:0030879 | mammary gland development | 10 | 0.017485 |
| BP | GO:0034599 | cellular response to oxidative stress | 17 | 0.017718 |
| BP | GO:0003170 | heart valve development | 6 | 0.01814 |
| BP | GO:0021872 | forebrain generation of neurons | 6 | 0.01814 |
| BP | GO:0046888 | negative regulation of hormone secretion | 6 | 0.01814 |
| BP | GO:0090303 | positive regulation of wound healing | 6 | 0.01814 |
| BP | GO:0003151 | outflow tract morphogenesis | 7 | 0.018199 |
| BP | GO:0051403 | stress-activated MAPK cascade | 16 | 0.018317 |
| BP | GO:0006367 | transcription initiation from RNA polymerase II promoter | 12 | 0.018473 |
| BP | GO:0001916 | positive regulation of T cell mediated cytotoxicity | 4 | 0.018478 |
| BP | GO:0010460 | positive regulation of heart rate | 4 | 0.018478 |
| BP | GO:0010575 | positive regulation of vascular endothelial growth factor production | 4 | 0.018478 |
| BP | GO:1990776 | response to angiotensin | 4 | 0.018478 |
| BP | GO:0007254 | JNK cascade | 13 | 0.018601 |
| BP | GO:0090276 | regulation of peptide hormone secretion | 13 | 0.018601 |
| BP | GO:0042692 | muscle cell differentiation | 20 | 0.018738 |
| BP | GO:0060047 | heart contraction | 16 | 0.018753 |
| BP | GO:1905521 | regulation of macrophage migration | 5 | 0.018837 |
| BP | GO:0021782 | glial cell development | 9 | 0.018837 |
| BP | GO:0070838 | divalent metal ion transport | 23 | 0.019124 |
| BP | GO:0001946 | lymphangiogenesis | 3 | 0.019128 |
| BP | GO:0002467 | germinal center formation | 3 | 0.019128 |
| BP | GO:0010455 | positive regulation of cell fate commitment | 3 | 0.019128 |
| BP | GO:0020027 | hemoglobin metabolic process | 3 | 0.019128 |
| BP | GO:0035635 | entry of bacterium into host cell | 3 | 0.019128 |
| BP | GO:0044406 | adhesion of symbiont to host | 3 | 0.019128 |
| BP | GO:0045346 | regulation of MHC class II biosynthetic process | 3 | 0.019128 |
| BP | GO:0060347 | heart trabecula formation | 3 | 0.019128 |
| BP | GO:0060841 | venous blood vessel development | 3 | 0.019128 |
| BP | GO:0072567 | chemokine (C-X-C motif) ligand 2 production | 3 | 0.019128 |
| BP | GO:0090594 | inflammatory response to wounding | 3 | 0.019128 |
| BP | GO:2000341 | regulation of chemokine (C-X-C motif) ligand 2 production | 3 | 0.019128 |
| BP | GO:2001028 | positive regulation of endothelial cell chemotaxis | 3 | 0.019128 |
| BP | GO:0021954 | central nervous system neuron development | 7 | 0.020232 |
| BP | GO:1902930 | regulation of alcohol biosynthetic process | 7 | 0.020232 |
| BP | GO:0016525 | negative regulation of angiogenesis | 12 | 0.020307 |
| BP | GO:0002715 | regulation of natural killer cell mediated immunity | 5 | 0.020313 |
| BP | GO:0043114 | regulation of vascular permeability | 5 | 0.020313 |
| BP | GO:0060443 | mammary gland morphogenesis | 5 | 0.020313 |
| BP | GO:2001222 | regulation of neuron migration | 5 | 0.020313 |
| BP | GO:0002717 | positive regulation of natural killer cell mediated immunity | 4 | 0.020355 |
| BP | GO:0007263 | nitric oxide mediated signal transduction | 4 | 0.020355 |
| BP | GO:0033032 | regulation of myeloid cell apoptotic process | 4 | 0.020355 |
| BP | GO:0050901 | leukocyte tethering or rolling | 4 | 0.020355 |
| BP | GO:2000727 | positive regulation of cardiac muscle cell differentiation | 4 | 0.020355 |
| BP | GO:0055008 | cardiac muscle tissue morphogenesis | 6 | 0.02041 |
| BP | GO:0051384 | response to glucocorticoid | 10 | 0.021174 |
| BP | GO:2000241 | regulation of reproductive process | 11 | 0.021262 |
| BP | GO:0001570 | vasculogenesis | 7 | 0.021262 |
| BP | GO:0014855 | striated muscle cell proliferation | 7 | 0.021262 |
| BP | GO:2000243 | positive regulation of reproductive process | 7 | 0.021262 |
| BP | GO:1904951 | positive regulation of establishment of protein localization | 19 | 0.021494 |
| BP | GO:2000181 | negative regulation of blood vessel morphogenesis | 12 | 0.02162 |
| BP | GO:0002027 | regulation of heart rate | 8 | 0.021783 |
| BP | GO:0042130 | negative regulation of T cell proliferation | 6 | 0.021783 |
| BP | GO:0046173 | polyol biosynthetic process | 6 | 0.021783 |
| BP | GO:0046686 | response to cadmium ion | 6 | 0.021783 |
| BP | GO:0072511 | divalent inorganic cation transport | 23 | 0.021826 |
| BP | GO:0050798 | activated T cell proliferation | 5 | 0.021895 |
| BP | GO:0031644 | regulation of nervous system process | 10 | 0.021897 |
| BP | GO:0055007 | cardiac muscle cell differentiation | 9 | 0.022196 |
| BP | GO:0002295 | T-helper cell lineage commitment | 3 | 0.022257 |
| BP | GO:0002830 | positive regulation of type 2 immune response | 3 | 0.022257 |
| BP | GO:0007567 | parturition | 3 | 0.022257 |
| BP | GO:0014061 | regulation of norepinephrine secretion | 3 | 0.022257 |
| BP | GO:0033008 | positive regulation of mast cell activation involved in immune response | 3 | 0.022257 |
| BP | GO:0043306 | positive regulation of mast cell degranulation | 3 | 0.022257 |
| BP | GO:0048521 | negative regulation of behavior | 3 | 0.022257 |
| BP | GO:0071380 | cellular response to prostaglandin E stimulus | 3 | 0.022257 |
| BP | GO:0072224 | metanephric glomerulus development | 3 | 0.022257 |
| BP | GO:1900451 | positive regulation of glutamate receptor signaling pathway | 3 | 0.022257 |
| BP | GO:2001224 | positive regulation of neuron migration | 3 | 0.022257 |
| BP | GO:0010758 | regulation of macrophage chemotaxis | 4 | 0.022403 |
| BP | GO:0031532 | actin cytoskeleton reorganization | 8 | 0.02259 |
| BP | GO:1902106 | negative regulation of leukocyte differentiation | 8 | 0.02259 |
| BP | GO:0071897 | DNA biosynthetic process | 12 | 0.022871 |
| BP | GO:0006940 | regulation of smooth muscle contraction | 6 | 0.022985 |
| BP | GO:0048645 | animal organ formation | 6 | 0.022985 |
| BP | GO:0098586 | cellular response to virus | 6 | 0.022985 |
| BP | GO:0042593 | glucose homeostasis | 14 | 0.022985 |
| BP | GO:0045844 | positive regulation of striated muscle tissue development | 7 | 0.023402 |
| BP | GO:0048636 | positive regulation of muscle organ development | 7 | 0.023402 |
| BP | GO:0043300 | regulation of leukocyte degranulation | 5 | 0.023402 |
| BP | GO:0045933 | positive regulation of muscle contraction | 5 | 0.023402 |
| BP | GO:0048483 | autonomic nervous system development | 5 | 0.023402 |
| BP | GO:0007599 | hemostasis | 18 | 0.023417 |
| BP | GO:0099177 | regulation of trans-synaptic signaling | 22 | 0.023495 |
| BP | GO:0033500 | carbohydrate homeostasis | 14 | 0.0236 |
| BP | GO:0002228 | natural killer cell mediated immunity | 6 | 0.0244 |
| BP | GO:0007585 | respiratory gaseous exchange by respiratory system | 6 | 0.0244 |
| BP | GO:0034394 | protein localization to cell surface | 6 | 0.0244 |
| BP | GO:0045669 | positive regulation of osteoblast differentiation | 6 | 0.0244 |
| BP | GO:1905207 | regulation of cardiocyte differentiation | 6 | 0.0244 |
| BP | GO:0001776 | leukocyte homeostasis | 7 | 0.024488 |
| BP | GO:1901863 | positive regulation of muscle tissue development | 7 | 0.024488 |
| BP | GO:0002828 | regulation of type 2 immune response | 4 | 0.024488 |
| BP | GO:0021772 | olfactory bulb development | 4 | 0.024488 |
| BP | GO:0032770 | positive regulation of monooxygenase activity | 4 | 0.024488 |
| BP | GO:0034368 | protein-lipid complex remodeling | 4 | 0.024488 |
| BP | GO:0034369 | plasma lipoprotein particle remodeling | 4 | 0.024488 |
| BP | GO:0045940 | positive regulation of steroid metabolic process | 4 | 0.024488 |
| BP | GO:0045987 | positive regulation of smooth muscle contraction | 4 | 0.024488 |
| BP | GO:0070229 | negative regulation of lymphocyte apoptotic process | 4 | 0.024488 |
| BP | GO:2000406 | positive regulation of T cell migration | 4 | 0.024488 |
| BP | GO:1903426 | regulation of reactive oxygen species biosynthetic process | 8 | 0.024513 |
| BP | GO:0002576 | platelet degranulation | 9 | 0.024678 |
| BP | GO:0002820 | negative regulation of adaptive immune response | 5 | 0.024963 |
| BP | GO:0009409 | response to cold | 5 | 0.024963 |
| BP | GO:0055023 | positive regulation of cardiac muscle tissue growth | 5 | 0.024963 |
| BP | GO:0070169 | positive regulation of biomineral tissue development | 5 | 0.024963 |
| BP | GO:0110151 | positive regulation of biomineralization | 5 | 0.024963 |
| BP | GO:0032970 | regulation of actin filament-based process | 20 | 0.02537 |
| BP | GO:0002643 | regulation of tolerance induction | 3 | 0.025408 |
| BP | GO:0003184 | pulmonary valve morphogenesis | 3 | 0.025408 |
| BP | GO:0016045 | detection of bacterium | 3 | 0.025408 |
| BP | GO:0032966 | negative regulation of collagen biosynthetic process | 3 | 0.025408 |
| BP | GO:0046851 | negative regulation of bone remodeling | 3 | 0.025408 |
| BP | GO:0048243 | norepinephrine secretion | 3 | 0.025408 |
| BP | GO:0060263 | regulation of respiratory burst | 3 | 0.025408 |
| BP | GO:0019218 | regulation of steroid metabolic process | 9 | 0.026775 |
| BP | GO:0006692 | prostanoid metabolic process | 5 | 0.026775 |
| BP | GO:0006693 | prostaglandin metabolic process | 5 | 0.026775 |
| BP | GO:0042059 | negative regulation of epidermal growth factor receptor signaling pathway | 5 | 0.026775 |
| BP | GO:0070231 | T cell apoptotic process | 5 | 0.026775 |
| BP | GO:0097720 | calcineurin-mediated signaling | 5 | 0.026775 |
| BP | GO:0003176 | aortic valve development | 4 | 0.026775 |
| BP | GO:0034367 | protein-containing complex remodeling | 4 | 0.026775 |
| BP | GO:0043304 | regulation of mast cell degranulation | 4 | 0.026775 |
| BP | GO:0060317 | cardiac epithelial to mesenchymal transition | 4 | 0.026775 |
| BP | GO:0097421 | liver regeneration | 4 | 0.026775 |
| BP | GO:0007179 | transforming growth factor beta receptor signaling pathway | 12 | 0.027252 |
| BP | GO:0072009 | nephron epithelium development | 8 | 0.028011 |
| BP | GO:0110053 | regulation of actin filament organization | 15 | 0.028112 |
| BP | GO:0043537 | negative regulation of blood vessel endothelial cell migration | 7 | 0.028454 |
| BP | GO:0001961 | positive regulation of cytokine-mediated signaling pathway | 5 | 0.028785 |
| BP | GO:0008038 | neuron recognition | 5 | 0.028785 |
| BP | GO:0010718 | positive regulation of epithelial to mesenchymal transition | 5 | 0.028785 |
| BP | GO:0030195 | negative regulation of blood coagulation | 5 | 0.028785 |
| BP | GO:0101023 | vascular endothelial cell proliferation | 5 | 0.028785 |
| BP | GO:1905562 | regulation of vascular endothelial cell proliferation | 5 | 0.028785 |
| BP | GO:0060038 | cardiac muscle cell proliferation | 6 | 0.028785 |
| BP | GO:2000573 | positive regulation of DNA biosynthetic process | 6 | 0.028785 |
| BP | GO:0060348 | bone development | 12 | 0.028986 |
| BP | GO:0010713 | negative regulation of collagen metabolic process | 3 | 0.029141 |
| BP | GO:0034755 | iron ion transmembrane transport | 3 | 0.029141 |
| BP | GO:0035313 | wound healing, spreading of epidermal cells | 3 | 0.029141 |
| BP | GO:0035994 | response to muscle stretch | 3 | 0.029141 |
| BP | GO:0061298 | retina vasculature development in camera-type eye | 3 | 0.029141 |
| BP | GO:0033006 | regulation of mast cell activation involved in immune response | 4 | 0.029335 |
| BP | GO:0008202 | steroid metabolic process | 17 | 0.029353 |
| BP | GO:0003012 | muscle system process | 22 | 0.029367 |
| BP | GO:0042058 | regulation of epidermal growth factor receptor signaling pathway | 7 | 0.02968 |
| BP | GO:0051495 | positive regulation of cytoskeleton organization | 13 | 0.030006 |
| BP | GO:0097191 | extrinsic apoptotic signaling pathway | 13 | 0.030006 |
| BP | GO:0021879 | forebrain neuron differentiation | 5 | 0.030719 |
| BP | GO:0035176 | social behavior | 5 | 0.030719 |
| BP | GO:0038084 | vascular endothelial growth factor signaling pathway | 5 | 0.030719 |
| BP | GO:1900047 | negative regulation of hemostasis | 5 | 0.030719 |
| BP | GO:1903587 | regulation of blood vessel endothelial cell proliferation involved in sprouting angiogenesis | 5 | 0.030719 |
| BP | GO:0030203 | glycosaminoglycan metabolic process | 10 | 0.030782 |
| BP | GO:0035051 | cardiocyte differentiation | 10 | 0.030782 |
| BP | GO:1903707 | negative regulation of hemopoiesis | 10 | 0.030782 |
| BP | GO:0003158 | endothelium development | 9 | 0.031001 |
| BP | GO:0001656 | metanephros development | 7 | 0.031065 |
| BP | GO:0033273 | response to vitamin | 7 | 0.031065 |
| BP | GO:0035821 | modulation of process of other organism | 8 | 0.031642 |
| BP | GO:0032868 | response to insulin | 15 | 0.031695 |
| BP | GO:0014706 | striated muscle tissue development | 19 | 0.031732 |
| BP | GO:0055067 | monovalent inorganic cation homeostasis | 10 | 0.031868 |
| BP | GO:0030431 | sleep | 4 | 0.031939 |
| BP | GO:0070232 | regulation of T cell apoptotic process | 4 | 0.031939 |
| BP | GO:0046579 | positive regulation of Ras protein signal transduction | 6 | 0.031994 |
| BP | GO:0051785 | positive regulation of nuclear division | 6 | 0.031994 |
| BP | GO:0003007 | heart morphogenesis | 14 | 0.03219 |
| BP | GO:0010883 | regulation of lipid storage | 5 | 0.03268 |
| BP | GO:0060421 | positive regulation of heart growth | 5 | 0.03268 |
| BP | GO:0072132 | mesenchyme morphogenesis | 5 | 0.03268 |
| BP | GO:0001696 | gastric acid secretion | 3 | 0.03268 |
| BP | GO:0002076 | osteoblast development | 3 | 0.03268 |
| BP | GO:0002862 | negative regulation of inflammatory response to antigenic stimulus | 3 | 0.03268 |
| BP | GO:0010759 | positive regulation of macrophage chemotaxis | 3 | 0.03268 |
| BP | GO:0043373 | CD4-positive, alpha-beta T cell lineage commitment | 3 | 0.03268 |
| BP | GO:0044320 | cellular response to leptin stimulus | 3 | 0.03268 |
| BP | GO:0048486 | parasympathetic nervous system development | 3 | 0.03268 |
| BP | GO:0051546 | keratinocyte migration | 3 | 0.03268 |
| BP | GO:0060602 | branch elongation of an epithelium | 3 | 0.03268 |
| BP | GO:0060749 | mammary gland alveolus development | 3 | 0.03268 |
| BP | GO:0061377 | mammary gland lobule development | 3 | 0.03268 |
| BP | GO:0070233 | negative regulation of T cell apoptotic process | 3 | 0.03268 |
| BP | GO:1900747 | negative regulation of vascular endothelial growth factor signaling pathway | 3 | 0.03268 |
| BP | GO:2000251 | positive regulation of actin cytoskeleton reorganization | 3 | 0.03268 |
| BP | GO:2000319 | regulation of T-helper 17 cell differentiation | 3 | 0.03268 |
| BP | GO:0021537 | telencephalon development | 14 | 0.03268 |
| BP | GO:0030183 | B cell differentiation | 9 | 0.033005 |
| BP | GO:0051052 | regulation of DNA metabolic process | 18 | 0.033408 |
| BP | GO:0007249 | I-kappaB kinase/NF-kappaB signaling | 15 | 0.03376 |
| BP | GO:0002532 | production of molecular mediator involved in inflammatory response | 7 | 0.033789 |
| BP | GO:0031623 | receptor internalization | 8 | 0.033967 |
| BP | GO:0006109 | regulation of carbohydrate metabolic process | 12 | 0.034103 |
| BP | GO:0055076 | transition metal ion homeostasis | 9 | 0.034296 |
| BP | GO:0030513 | positive regulation of BMP signaling pathway | 4 | 0.034407 |
| BP | GO:0097009 | energy homeostasis | 4 | 0.034407 |
| BP | GO:0045744 | negative regulation of G protein-coupled receptor signaling pathway | 5 | 0.034576 |
| BP | GO:0051703 | intraspecies interaction between organisms | 5 | 0.034576 |
| BP | GO:0051155 | positive regulation of striated muscle cell differentiation | 6 | 0.035376 |
| BP | GO:0051591 | response to cAMP | 7 | 0.035398 |
| BP | GO:0032872 | regulation of stress-activated MAPK cascade | 13 | 0.035979 |
| BP | GO:0046777 | protein autophosphorylation | 13 | 0.035979 |
| BP | GO:0010524 | positive regulation of calcium ion transport into cytosol | 5 | 0.036881 |
| BP | GO:1901185 | negative regulation of ERBB signaling pathway | 5 | 0.036881 |
| BP | GO:0002418 | immune response to tumor cell | 3 | 0.036881 |
| BP | GO:0002726 | positive regulation of T cell cytokine production | 3 | 0.036881 |
| BP | GO:0007252 | I-kappaB phosphorylation | 3 | 0.036881 |
| BP | GO:0007620 | copulation | 3 | 0.036881 |
| BP | GO:0032727 | positive regulation of interferon-alpha production | 3 | 0.036881 |
| BP | GO:0034138 | toll-like receptor 3 signaling pathway | 3 | 0.036881 |
| BP | GO:0036303 | lymph vessel morphogenesis | 3 | 0.036881 |
| BP | GO:0048670 | regulation of collateral sprouting | 3 | 0.036881 |
| BP | GO:0042493 | response to drug | 19 | 0.036881 |
| BP | GO:0070167 | regulation of biomineral tissue development | 7 | 0.036881 |
| BP | GO:0110149 | regulation of biomineralization | 7 | 0.036881 |
| BP | GO:0010976 | positive regulation of neuron projection development | 15 | 0.037023 |
| BP | GO:0034121 | regulation of toll-like receptor signaling pathway | 6 | 0.037024 |
| BP | GO:0043507 | positive regulation of JUN kinase activity | 6 | 0.037024 |
| BP | GO:2001259 | positive regulation of cation channel activity | 6 | 0.037024 |
| BP | GO:0002755 | MyD88-dependent toll-like receptor signaling pathway | 4 | 0.0371 |
| BP | GO:0031128 | developmental induction | 4 | 0.0371 |
| BP | GO:0071634 | regulation of transforming growth factor beta production | 4 | 0.0371 |
| BP | GO:0043087 | regulation of GTPase activity | 22 | 0.037431 |
| BP | GO:0001889 | liver development | 9 | 0.038013 |
| BP | GO:0050868 | negative regulation of T cell activation | 8 | 0.038079 |
| BP | GO:0050804 | modulation of chemical synaptic transmission | 21 | 0.038079 |
| BP | GO:0070302 | regulation of stress-activated protein kinase signaling cascade | 13 | 0.038716 |
| BP | GO:0015909 | long-chain fatty acid transport | 6 | 0.03897 |
| BP | GO:0032418 | lysosome localization | 6 | 0.03897 |
| BP | GO:0035924 | cellular response to vascular endothelial growth factor stimulus | 6 | 0.03897 |
| BP | GO:0022029 | telencephalon cell migration | 5 | 0.03897 |
| BP | GO:0032720 | negative regulation of tumor necrosis factor production | 5 | 0.03897 |
| BP | GO:0050819 | negative regulation of coagulation | 5 | 0.03897 |
| BP | GO:0007292 | female gamete generation | 9 | 0.039307 |
| BP | GO:1901222 | regulation of NIK/NF-kappaB signaling | 8 | 0.039556 |
| BP | GO:0048167 | regulation of synaptic plasticity | 11 | 0.04004 |
| BP | GO:1901184 | regulation of ERBB signaling pathway | 7 | 0.04004 |
| BP | GO:0030212 | hyaluronan metabolic process | 4 | 0.04004 |
| BP | GO:0032941 | secretion by tissue | 4 | 0.04004 |
| BP | GO:0060428 | lung epithelium development | 4 | 0.04004 |
| BP | GO:0090322 | regulation of superoxide metabolic process | 4 | 0.04004 |
| BP | GO:0097242 | amyloid-beta clearance | 4 | 0.04004 |
| BP | GO:1905314 | semi-lunar valve development | 4 | 0.04004 |
| BP | GO:0003281 | ventricular septum development | 6 | 0.040878 |
| BP | GO:0060415 | muscle tissue morphogenesis | 6 | 0.040878 |
| BP | GO:0002363 | alpha-beta T cell lineage commitment | 3 | 0.040952 |
| BP | GO:0030277 | maintenance of gastrointestinal epithelium | 3 | 0.040952 |
| BP | GO:0072574 | hepatocyte proliferation | 3 | 0.040952 |
| BP | GO:0072575 | epithelial cell proliferation involved in liver morphogenesis | 3 | 0.040952 |
| BP | GO:1902548 | negative regulation of cellular response to vascular endothelial growth factor stimulus | 3 | 0.040952 |
| BP | GO:0002712 | regulation of B cell mediated immunity | 5 | 0.041226 |
| BP | GO:0002889 | regulation of immunoglobulin mediated immune response | 5 | 0.041226 |
| BP | GO:0002832 | negative regulation of response to biotic stimulus | 7 | 0.041767 |
| BP | GO:0061008 | hepaticobiliary system development | 9 | 0.041978 |
| BP | GO:0071248 | cellular response to metal ion | 11 | 0.042441 |
| BP | GO:0014015 | positive regulation of gliogenesis | 6 | 0.042954 |
| BP | GO:0034765 | regulation of ion transmembrane transport | 22 | 0.043046 |
| BP | GO:0003156 | regulation of animal organ formation | 4 | 0.043153 |
| BP | GO:0019433 | triglyceride catabolic process | 4 | 0.043153 |
| BP | GO:0045777 | positive regulation of blood pressure | 4 | 0.043153 |
| BP | GO:0071604 | transforming growth factor beta production | 4 | 0.043153 |
| BP | GO:1903523 | negative regulation of blood circulation | 4 | 0.043153 |
| BP | GO:0038127 | ERBB signaling pathway | 9 | 0.043388 |
| BP | GO:0010717 | regulation of epithelial to mesenchymal transition | 7 | 0.043531 |
| BP | GO:0043547 | positive regulation of GTPase activity | 19 | 0.043593 |
| BP | GO:0002090 | regulation of receptor internalization | 5 | 0.043646 |
| BP | GO:0061005 | cell differentiation involved in kidney development | 5 | 0.043646 |
| BP | GO:1903556 | negative regulation of tumor necrosis factor superfamily cytokine production | 5 | 0.043646 |
| BP | GO:0010466 | negative regulation of peptidase activity | 14 | 0.044716 |
| BP | GO:0001895 | retina homeostasis | 6 | 0.044954 |
| BP | GO:0050688 | regulation of defense response to virus | 6 | 0.044954 |
| BP | GO:0071496 | cellular response to external stimulus | 16 | 0.045441 |
| BP | GO:0010888 | negative regulation of lipid storage | 3 | 0.045441 |
| BP | GO:0043369 | CD4-positive or CD8-positive, alpha-beta T cell lineage commitment | 3 | 0.045441 |
| BP | GO:0044342 | type B pancreatic cell proliferation | 3 | 0.045441 |
| BP | GO:0045655 | regulation of monocyte differentiation | 3 | 0.045441 |
| BP | GO:0060487 | lung epithelial cell differentiation | 3 | 0.045441 |
| BP | GO:0071157 | negative regulation of cell cycle arrest | 3 | 0.045441 |
| BP | GO:0072576 | liver morphogenesis | 3 | 0.045441 |
| BP | GO:0007173 | epidermal growth factor receptor signaling pathway | 8 | 0.045614 |
| BP | GO:0060537 | muscle tissue development | 19 | 0.046046 |
| BP | GO:0021885 | forebrain cell migration | 5 | 0.046208 |
| BP | GO:0001662 | behavioral fear response | 4 | 0.046276 |
| BP | GO:0010837 | regulation of keratinocyte proliferation | 4 | 0.046276 |
| BP | GO:0071260 | cellular response to mechanical stimulus | 6 | 0.047039 |
| BP | GO:0010522 | regulation of calcium ion transport into cytosol | 7 | 0.04719 |
| BP | GO:0006022 | aminoglycan metabolic process | 10 | 0.047202 |
| BP | GO:0050806 | positive regulation of synaptic transmission | 10 | 0.047202 |
| BP | GO:0010675 | regulation of cellular carbohydrate metabolic process | 9 | 0.047831 |
| BP | GO:0006352 | DNA-templated transcription, initiation | 13 | 0.048184 |
| BP | GO:0006636 | unsaturated fatty acid biosynthetic process | 5 | 0.048993 |
| BP | GO:0046456 | icosanoid biosynthetic process | 5 | 0.048993 |
| BP | GO:0001952 | regulation of cell-matrix adhesion | 8 | 0.049184 |
| BP | GO:0002312 | B cell activation involved in immune response | 6 | 0.049359 |
| BP | GO:2001233 | regulation of apoptotic signaling pathway | 19 | 0.04968 |
| BP | GO:0002209 | behavioral defense response | 4 | 0.04968 |
| BP | GO:0010613 | positive regulation of cardiac muscle hypertrophy | 4 | 0.04968 |
| BP | GO:0034142 | toll-like receptor 4 signaling pathway | 4 | 0.04968 |
| BP | GO:0043902 | positive regulation of multi-organism process | 4 | 0.04968 |
| BP | GO:0048713 | regulation of oligodendrocyte differentiation | 4 | 0.04968 |
| BP | GO:0051281 | positive regulation of release of sequestered calcium ion into cytosol | 4 | 0.04968 |
| BP | GO:0003205 | cardiac chamber development | 10 | 0.049849 |
| BP | GO:0007413 | axonal fasciculation | 3 | 0.049849 |
| BP | GO:0007617 | mating behavior | 3 | 0.049849 |
| BP | GO:0010226 | response to lithium ion | 3 | 0.049849 |
| BP | GO:0030539 | male genitalia development | 3 | 0.049849 |
| BP | GO:0032703 | negative regulation of interleukin-2 production | 3 | 0.049849 |
| BP | GO:0032799 | low-density lipoprotein receptor particle metabolic process | 3 | 0.049849 |
| BP | GO:0045649 | regulation of macrophage differentiation | 3 | 0.049849 |
| BP | GO:0048714 | positive regulation of oligodendrocyte differentiation | 3 | 0.049849 |
| BP | GO:0060445 | branching involved in salivary gland morphogenesis | 3 | 0.049849 |
| BP | GO:0060479 | lung cell differentiation | 3 | 0.049849 |
| BP | GO:0072215 | regulation of metanephros development | 3 | 0.049849 |
| BP | GO:0106030 | neuron projection fasciculation | 3 | 0.049849 |
| BP | GO:1903589 | positive regulation of blood vessel endothelial cell proliferation involved in sprouting angiogenesis | 3 | 0.049849 |
| CC | GO:0019814 | immunoglobulin complex | 132 | 6.96E-179 |
| CC | GO:0009897 | external side of plasma membrane | 102 | 6.26E-66 |
| CC | GO:0042571 | immunoglobulin complex, circulating | 54 | 1.22E-65 |
| CC | GO:0072562 | blood microparticle | 36 | 2.66E-22 |
| CC | GO:0034774 | secretory granule lumen | 30 | 3.35E-07 |
| CC | GO:0060205 | cytoplasmic vesicle lumen | 30 | 3.67E-07 |
| CC | GO:0031983 | vesicle lumen | 30 | 3.67E-07 |
| CC | GO:0062023 | collagen-containing extracellular matrix | 34 | 1.08E-06 |
| CC | GO:0005796 | Golgi lumen | 12 | 0.00081 |
| CC | GO:0042581 | specific granule | 14 | 0.003901 |
| CC | GO:0043020 | NADPH oxidase complex | 4 | 0.006097 |
| CC | GO:0035580 | specific granule lumen | 8 | 0.006705 |
| CC | GO:0045121 | membrane raft | 21 | 0.006782 |
| CC | GO:0098857 | membrane microdomain | 21 | 0.006782 |
| CC | GO:0098589 | membrane region | 21 | 0.010526 |
| CC | GO:0070820 | tertiary granule | 13 | 0.010897 |
| CC | GO:0030136 | clathrin-coated vesicle | 14 | 0.01627 |
| CC | GO:0031093 | platelet alpha granule lumen | 7 | 0.03771 |
| CC | GO:0030139 | endocytic vesicle | 18 | 0.039064 |
| CC | GO:0034358 | plasma lipoprotein particle | 5 | 0.039064 |
| CC | GO:1990777 | lipoprotein particle | 5 | 0.039064 |
| MF | GO:0030546 | signaling receptor activator activity | 143 | 6.08E-109 |
| MF | GO:0048018 | receptor ligand activity | 142 | 1.26E-108 |
| MF | GO:0003823 | antigen binding | 89 | 4.34E-94 |
| MF | GO:0034987 | immunoglobulin receptor binding | 54 | 1.43E-64 |
| MF | GO:0005125 | cytokine activity | 72 | 1.11E-54 |
| MF | GO:0005179 | hormone activity | 44 | 1.85E-36 |
| MF | GO:0008083 | growth factor activity | 48 | 2.74E-35 |
| MF | GO:0005126 | cytokine receptor binding | 54 | 3.66E-30 |
| MF | GO:0001664 | G protein-coupled receptor binding | 51 | 1.47E-25 |
| MF | GO:0008528 | G protein-coupled peptide receptor activity | 38 | 2.24E-25 |
| MF | GO:0001653 | peptide receptor activity | 38 | 5.93E-25 |
| MF | GO:0019955 | cytokine binding | 33 | 2.55E-21 |
| MF | GO:0008009 | chemokine activity | 22 | 9.04E-21 |
| MF | GO:0140375 | immune receptor activity | 30 | 3.81E-18 |
| MF | GO:0042379 | chemokine receptor binding | 23 | 4.38E-18 |
| MF | GO:0004896 | cytokine receptor activity | 25 | 8.05E-17 |
| MF | GO:0042562 | hormone binding | 21 | 7.39E-14 |
| MF | GO:0042277 | peptide binding | 36 | 1.58E-12 |
| MF | GO:0048020 | CCR chemokine receptor binding | 15 | 1.16E-11 |
| MF | GO:0017046 | peptide hormone binding | 15 | 4.24E-11 |
| MF | GO:0005539 | glycosaminoglycan binding | 29 | 7.06E-11 |
| MF | GO:0033218 | amide binding | 37 | 1.66E-10 |
| MF | GO:0070851 | growth factor receptor binding | 22 | 3.25E-10 |
| MF | GO:0045499 | chemorepellent activity | 11 | 8.49E-10 |
| MF | GO:0019838 | growth factor binding | 20 | 7.29E-09 |
| MF | GO:0005184 | neuropeptide hormone activity | 10 | 3.94E-08 |
| MF | GO:0030215 | semaphorin receptor binding | 9 | 4.58E-08 |
| MF | GO:0071855 | neuropeptide receptor binding | 10 | 1.05E-07 |
| MF | GO:0019199 | transmembrane receptor protein kinase activity | 14 | 2.59E-07 |
| MF | GO:0004879 | nuclear receptor activity | 11 | 1.04E-06 |
| MF | GO:0098531 | ligand-activated transcription factor activity | 11 | 1.04E-06 |
| MF | GO:0008201 | heparin binding | 19 | 1.35E-06 |
| MF | GO:0016500 | protein-hormone receptor activity | 7 | 2.24E-06 |
| MF | GO:0045236 | CXCR chemokine receptor binding | 7 | 2.24E-06 |
| MF | GO:0048306 | calcium-dependent protein binding | 13 | 3.55E-06 |
| MF | GO:0005024 | transforming growth factor beta-activated receptor activity | 6 | 4.79E-06 |
| MF | GO:0038187 | pattern recognition receptor activity | 7 | 1.41E-05 |
| MF | GO:0005501 | retinoid binding | 8 | 3.34E-05 |
| MF | GO:0019840 | isoprenoid binding | 8 | 3.95E-05 |
| MF | GO:0042056 | chemoattractant activity | 8 | 3.95E-05 |
| MF | GO:0050786 | RAGE receptor binding | 5 | 4.42E-05 |
| MF | GO:0004675 | transmembrane receptor protein serine/threonine kinase activity | 6 | 5.66E-05 |
| MF | GO:1901681 | sulfur compound binding | 21 | 5.94E-05 |
| MF | GO:0033612 | receptor serine/threonine kinase binding | 7 | 6.59E-05 |
| MF | GO:0016175 | superoxide-generating NAD(P)H oxidase activity | 5 | 0.000107 |
| MF | GO:0019956 | chemokine binding | 7 | 0.000157 |
| MF | GO:0017134 | fibroblast growth factor binding | 6 | 0.000168 |
| MF | GO:0050431 | transforming growth factor beta binding | 6 | 0.000168 |
| MF | GO:0005104 | fibroblast growth factor receptor binding | 6 | 0.000277 |
| MF | GO:0019841 | retinol binding | 5 | 0.000307 |
| MF | GO:0042923 | neuropeptide binding | 6 | 0.000338 |
| MF | GO:0042834 | peptidoglycan binding | 5 | 0.000554 |
| MF | GO:0030296 | protein tyrosine kinase activator activity | 5 | 0.000709 |
| MF | GO:0050664 | oxidoreductase activity, acting on NAD(P)H, oxygen as acceptor | 5 | 0.000709 |
| MF | GO:0004714 | transmembrane receptor protein tyrosine kinase activity | 8 | 0.001256 |
| MF | GO:1990782 | protein tyrosine kinase binding | 10 | 0.001701 |
| MF | GO:0061783 | peptidoglycan muralytic activity | 4 | 0.001734 |
| MF | GO:0070700 | BMP receptor binding | 4 | 0.001734 |
| MF | GO:0005160 | transforming growth factor beta receptor binding | 5 | 0.002089 |
| MF | GO:0070696 | transmembrane receptor protein serine/threonine kinase binding | 5 | 0.002089 |
| MF | GO:0048185 | activin binding | 4 | 0.002947 |
| MF | GO:0001637 | G protein-coupled chemoattractant receptor activity | 5 | 0.002947 |
| MF | GO:0004950 | chemokine receptor activity | 5 | 0.002947 |
| MF | GO:0030971 | receptor tyrosine kinase binding | 8 | 0.003404 |
| MF | GO:0038024 | cargo receptor activity | 8 | 0.003533 |
| MF | GO:0003953 | NAD+ nucleosidase activity | 4 | 0.003533 |
| MF | GO:0050135 | NAD(P)+ nucleosidase activity | 4 | 0.003533 |
| MF | GO:0061809 | NAD+ nucleotidase, cyclic ADP-ribose generating | 4 | 0.003533 |
| MF | GO:0016918 | retinal binding | 4 | 0.004458 |
| MF | GO:0004713 | protein tyrosine kinase activity | 11 | 0.004742 |
| MF | GO:0008188 | neuropeptide receptor activity | 6 | 0.005609 |
| MF | GO:0005164 | tumor necrosis factor receptor binding | 5 | 0.005973 |
| MF | GO:0032813 | tumor necrosis factor receptor superfamily binding | 6 | 0.007687 |
| MF | GO:0030414 | peptidase inhibitor activity | 13 | 0.007755 |
| MF | GO:0001972 | retinoic acid binding | 4 | 0.007839 |
| MF | GO:0005154 | epidermal growth factor receptor binding | 5 | 0.008526 |
| MF | GO:0004955 | prostaglandin receptor activity | 3 | 0.008526 |
| MF | GO:0005031 | tumor necrosis factor-activated receptor activity | 3 | 0.008526 |
| MF | GO:0017002 | activin-activated receptor activity | 3 | 0.008526 |
| MF | GO:0051428 | peptide hormone receptor binding | 4 | 0.00889 |
| MF | GO:0043394 | proteoglycan binding | 5 | 0.010567 |
| MF | GO:0004954 | prostanoid receptor activity | 3 | 0.01094 |
| MF | GO:0019864 | IgG binding | 3 | 0.01094 |
| MF | GO:0045309 | protein phosphorylated amino acid binding | 6 | 0.011182 |
| MF | GO:0051427 | hormone receptor binding | 12 | 0.011314 |
| MF | GO:0016493 | C-C chemokine receptor activity | 4 | 0.011733 |
| MF | GO:0033293 | monocarboxylic acid binding | 7 | 0.011801 |
| MF | GO:0019865 | immunoglobulin binding | 4 | 0.012902 |
| MF | GO:0019957 | C-C chemokine binding | 4 | 0.012902 |
| MF | GO:0004875 | complement receptor activity | 3 | 0.012902 |
| MF | GO:0005035 | death receptor activity | 3 | 0.012902 |
| MF | GO:0017154 | semaphorin receptor activity | 3 | 0.012902 |
| MF | GO:0036122 | BMP binding | 3 | 0.016268 |
| MF | GO:0003707 | steroid hormone receptor activity | 4 | 0.017022 |
| MF | GO:0001784 | phosphotyrosine residue binding | 5 | 0.017853 |
| MF | GO:0005178 | integrin binding | 10 | 0.018536 |
| MF | GO:0044548 | S100 protein binding | 3 | 0.019464 |
| MF | GO:0004953 | icosanoid receptor activity | 3 | 0.023612 |
| MF | GO:0001223 | transcription coactivator binding | 4 | 0.02412 |
| MF | GO:0004857 | enzyme inhibitor activity | 19 | 0.024315 |
| MF | GO:0005044 | scavenger receptor activity | 5 | 0.027185 |
| MF | GO:0043395 | heparan sulfate proteoglycan binding | 3 | 0.027378 |
| MF | GO:0061134 | peptidase regulator activity | 13 | 0.02824 |
| MF | GO:0030169 | low-density lipoprotein particle binding | 3 | 0.031971 |
| MF | GO:0004866 | endopeptidase inhibitor activity | 11 | 0.032568 |
| MF | GO:0001221 | transcription cofactor binding | 5 | 0.0364 |
| MF | GO:0016799 | hydrolase activity, hydrolyzing N-glycosyl compounds | 4 | 0.03928 |
| MF | GO:0061135 | endopeptidase regulator activity | 11 | 0.044184 |
| MF | GO:0050661 | NADP binding | 5 | 0.044688 |
| MF | GO:0004252 | serine-type endopeptidase activity | 10 | 0.047167 |
| MF | GO:0004867 | serine-type endopeptidase inhibitor activity | 7 | 0.048455 |
