# Supplementary material for: A Prognostic Risk Score Based on Hypoxia-, Immunity-, and Epithelialto-Mesenchymal Transition-Related Genes for the Prognosis and Immunotherapy Response of Lung Adenocarcinoma
Source: Front Cell Dev Biol. 2022 Jan 24;9:758777. doi: 10.3389/fcell.2021.758777 (PMC8819669; doi:10.3389/fcell.2021.758777)
Supplement: Supplementary file 10 [file Table11.DOCX]

| **Supplementary Table 11 \| KEGG pathway enrichment analysis of DEGs between high risk score group and low**  **risk score group** | | | |
| --- | --- | --- | --- |
| ID | Description | Count | qvalue |
| hsa04974 | Protein digestion and absorption | 13 | 0.007358691 |
| hsa04610 | Complement and coagulation cascades | 11 | 0.010988231 |
| hsa04512 | ECM-receptor interaction | 11 | 0.010988231 |
| hsa04640 | Hematopoietic cell lineage | 11 | 0.023573072 |
| hsa04972 | Pancreatic secretion | 11 | 0.024430258 |
| hsa00590 | Arachidonic acid metabolism | 8 | 0.032694486 |
| hsa04510 | Focal adhesion | 16 | 0.032874017 |
