# Supplementary material for: A Prognostic Risk Score Based on Hypoxia-, Immunity-, and Epithelialto-Mesenchymal Transition-Related Genes for the Prognosis and Immunotherapy Response of Lung Adenocarcinoma
Source: Front Cell Dev Biol. 2022 Jan 24;9:758777. doi: 10.3389/fcell.2021.758777 (PMC8819669; doi:10.3389/fcell.2021.758777)
Supplement: Supplementary file 11 [file Table6.DOCX]

|  |  |  |  |  |  |
| --- | --- | --- | --- | --- | --- |
|  | **Supplementary Table 6 \| GO enrichment analysis of EMT-DEGs** | | | | |
|  | Category | ID | Trem | Count | qvalue |
|  | BP | GO:0030198 | extracellular matrix organization | 37 | 8.63E-38 |
|  | BP | GO:0043062 | extracellular structure organization | 37 | 8.63E-38 |
|  | BP | GO:0032963 | collagen metabolic process | 14 | 2.41E-14 |
|  | BP | GO:0030199 | collagen fibril organization | 11 | 1.92E-13 |
|  | BP | GO:0001503 | ossification | 17 | 4.99E-10 |
|  | BP | GO:0031589 | cell-substrate adhesion | 16 | 5.63E-10 |
|  | BP | GO:0051216 | cartilage development | 13 | 5.63E-10 |
|  | BP | GO:0061448 | connective tissue development | 14 | 1.16E-09 |
|  | BP | GO:0009612 | response to mechanical stimulus | 12 | 1.66E-08 |
|  | BP | GO:0032964 | collagen biosynthetic process | 7 | 4.50E-07 |
|  | BP | GO:0007160 | cell-matrix adhesion | 11 | 6.01E-07 |
|  | BP | GO:0051271 | negative regulation of cellular component movement | 13 | 2.03E-06 |
|  | BP | GO:0016049 | cell growth | 14 | 2.39E-06 |
|  | BP | GO:0002062 | chondrocyte differentiation | 8 | 2.39E-06 |
|  | BP | GO:0010721 | negative regulation of cell development | 12 | 2.70E-06 |
|  | BP | GO:0001558 | regulation of cell growth | 13 | 2.70E-06 |
|  | BP | GO:0010810 | regulation of cell-substrate adhesion | 10 | 2.96E-06 |
|  | BP | GO:0035987 | endodermal cell differentiation | 6 | 3.90E-06 |
|  | BP | GO:2000146 | negative regulation of cell motility | 12 | 4.27E-06 |
|  | BP | GO:0001649 | osteoblast differentiation | 10 | 4.54E-06 |
|  | BP | GO:0022617 | extracellular matrix disassembly | 7 | 4.78E-06 |
|  | BP | GO:0001704 | formation of primary germ layer | 8 | 5.42E-06 |
|  | BP | GO:0052547 | regulation of peptidase activity | 13 | 6.33E-06 |
|  | BP | GO:0040013 | negative regulation of locomotion | 12 | 8.43E-06 |
|  | BP | GO:0001706 | endoderm formation | 6 | 8.44E-06 |
|  | BP | GO:0033690 | positive regulation of osteoblast proliferation | 4 | 9.69E-06 |
|  | BP | GO:0033688 | regulation of osteoblast proliferation | 5 | 1.04E-05 |
|  | BP | GO:0002063 | chondrocyte development | 5 | 1.36E-05 |
|  | BP | GO:0007596 | blood coagulation | 11 | 1.36E-05 |
|  | BP | GO:0007599 | hemostasis | 11 | 1.51E-05 |
|  | BP | GO:0050817 | coagulation | 11 | 1.51E-05 |
|  | BP | GO:0030336 | negative regulation of cell migration | 11 | 1.51E-05 |
|  | BP | GO:0007178 | transmembrane receptor protein serine/threonine kinase signaling pathway | 11 | 1.84E-05 |
|  | BP | GO:0033687 | osteoblast proliferation | 5 | 1.84E-05 |
|  | BP | GO:0007229 | integrin-mediated signaling pathway | 7 | 1.92E-05 |
|  | BP | GO:0043687 | post-translational protein modification | 11 | 1.93E-05 |
|  | BP | GO:0031214 | biomineral tissue development | 8 | 2.13E-05 |
|  | BP | GO:0110148 | biomineralization | 8 | 2.13E-05 |
|  | BP | GO:0050673 | epithelial cell proliferation | 12 | 2.13E-05 |
|  | BP | GO:0048771 | tissue remodeling | 8 | 3.98E-05 |
|  | BP | GO:0007492 | endoderm development | 6 | 4.54E-05 |
|  | BP | GO:0007517 | muscle organ development | 11 | 5.08E-05 |
|  | BP | GO:0007369 | gastrulation | 8 | 5.81E-05 |
|  | BP | GO:0071559 | response to transforming growth factor beta | 9 | 6.06E-05 |
|  | BP | GO:0030574 | collagen catabolic process | 5 | 6.32E-05 |
|  | BP | GO:0010712 | regulation of collagen metabolic process | 5 | 7.63E-05 |
|  | BP | GO:0031099 | regeneration | 8 | 8.42E-05 |
|  | BP | GO:0010466 | negative regulation of peptidase activity | 9 | 8.59E-05 |
|  | BP | GO:0046849 | bone remodeling | 6 | 8.75E-05 |
|  | BP | GO:0060348 | bone development | 8 | 8.84E-05 |
|  | BP | GO:0048608 | reproductive structure development | 11 | 9.50E-05 |
|  | BP | GO:0061458 | reproductive system development | 11 | 0.000102 |
|  | BP | GO:0002685 | regulation of leukocyte migration | 8 | 0.000111 |
|  | BP | GO:0001657 | ureteric bud development | 6 | 0.000122 |
|  | BP | GO:0072163 | mesonephric epithelium development | 6 | 0.000125 |
|  | BP | GO:0072164 | mesonephric tubule development | 6 | 0.000125 |
|  | BP | GO:0050768 | negative regulation of neurogenesis | 9 | 0.00014 |
|  | BP | GO:0030168 | platelet activation | 7 | 0.00015 |
|  | BP | GO:0001823 | mesonephros development | 6 | 0.00015 |
|  | BP | GO:0051346 | negative regulation of hydrolase activity | 11 | 0.000151 |
|  | BP | GO:0048705 | skeletal system morphogenesis | 8 | 0.000165 |
|  | BP | GO:0001667 | ameboidal-type cell migration | 11 | 0.000171 |
|  | BP | GO:0051961 | negative regulation of nervous system development | 9 | 0.000235 |
|  | BP | GO:0050974 | detection of mechanical stimulus involved in sensory perception | 4 | 0.000235 |
|  | BP | GO:0048660 | regulation of smooth muscle cell proliferation | 7 | 0.000242 |
|  | BP | GO:0033002 | muscle cell proliferation | 8 | 0.00025 |
|  | BP | GO:0048659 | smooth muscle cell proliferation | 7 | 0.000253 |
|  | BP | GO:0010812 | negative regulation of cell-substrate adhesion | 5 | 0.000265 |
|  | BP | GO:0030282 | bone mineralization | 6 | 0.000267 |
|  | BP | GO:0043589 | skin morphogenesis | 3 | 0.000291 |
|  | BP | GO:0071560 | cellular response to transforming growth factor beta stimulus | 8 | 0.000294 |
|  | BP | GO:1903034 | regulation of response to wounding | 7 | 0.000315 |
|  | BP | GO:0061035 | regulation of cartilage development | 5 | 0.000327 |
|  | BP | GO:0010951 | negative regulation of endopeptidase activity | 8 | 0.000334 |
|  | BP | GO:0031345 | negative regulation of cell projection organization | 7 | 0.000357 |
|  | BP | GO:0052548 | regulation of endopeptidase activity | 10 | 0.000357 |
|  | BP | GO:0035265 | organ growth | 7 | 0.000388 |
|  | BP | GO:0001935 | endothelial cell proliferation | 7 | 0.0005 |
|  | BP | GO:0010631 | epithelial cell migration | 9 | 0.000553 |
|  | BP | GO:0090132 | epithelium migration | 9 | 0.000582 |
|  | BP | GO:0045861 | negative regulation of proteolysis | 9 | 0.000582 |
|  | BP | GO:0048041 | focal adhesion assembly | 5 | 0.000582 |
|  | BP | GO:0090130 | tissue migration | 9 | 0.000636 |
|  | BP | GO:0072073 | kidney epithelium development | 6 | 0.000659 |
|  | BP | GO:0032965 | regulation of collagen biosynthetic process | 4 | 0.000733 |
|  | BP | GO:0007162 | negative regulation of cell adhesion | 8 | 0.000845 |
|  | BP | GO:0033273 | response to vitamin | 5 | 0.000845 |
|  | BP | GO:0050982 | detection of mechanical stimulus | 4 | 0.000845 |
|  | BP | GO:0061041 | regulation of wound healing | 6 | 0.000878 |
|  | BP | GO:0001837 | epithelial to mesenchymal transition | 6 | 0.000888 |
|  | BP | GO:0046851 | negative regulation of bone remodeling | 3 | 0.000888 |
|  | BP | GO:0070482 | response to oxygen levels | 9 | 0.000888 |
|  | BP | GO:1903035 | negative regulation of response to wounding | 5 | 0.000888 |
|  | BP | GO:0060349 | bone morphogenesis | 5 | 0.000913 |
|  | BP | GO:0010977 | negative regulation of neuron projection development | 6 | 0.000913 |
|  | BP | GO:0044706 | multi-multicellular organism process | 7 | 0.000913 |
|  | BP | GO:0010769 | regulation of cell morphogenesis involved in differentiation | 8 | 0.000933 |
|  | BP | GO:0090287 | regulation of cellular response to growth factor stimulus | 8 | 0.000933 |
|  | BP | GO:0045765 | regulation of angiogenesis | 9 | 0.000952 |
|  | BP | GO:0010718 | positive regulation of epithelial to mesenchymal transition | 4 | 0.001026 |
|  | BP | GO:0046850 | regulation of bone remodeling | 4 | 0.001026 |
|  | BP | GO:0060537 | muscle tissue development | 9 | 0.00103 |
|  | BP | GO:0010771 | negative regulation of cell morphogenesis involved in differentiation | 5 | 0.00103 |
|  | BP | GO:0007044 | cell-substrate junction assembly | 5 | 0.00106 |
|  | BP | GO:0010717 | regulation of epithelial to mesenchymal transition | 5 | 0.00106 |
|  | BP | GO:0060560 | developmental growth involved in morphogenesis | 7 | 0.001086 |
|  | BP | GO:0035988 | chondrocyte proliferation | 3 | 0.001089 |
|  | BP | GO:0032330 | regulation of chondrocyte differentiation | 4 | 0.00112 |
|  | BP | GO:0150115 | cell-substrate junction organization | 5 | 0.001178 |
|  | BP | GO:0048661 | positive regulation of smooth muscle cell proliferation | 5 | 0.001222 |
|  | BP | GO:0001655 | urogenital system development | 8 | 0.001276 |
|  | BP | GO:0045785 | positive regulation of cell adhesion | 9 | 0.001334 |
|  | BP | GO:0060350 | endochondral bone morphogenesis | 4 | 0.001336 |
|  | BP | GO:0032102 | negative regulation of response to external stimulus | 9 | 0.001431 |
|  | BP | GO:0034329 | cell junction assembly | 9 | 0.001444 |
|  | BP | GO:0034104 | negative regulation of tissue remodeling | 3 | 0.001582 |
|  | BP | GO:0007566 | embryo implantation | 4 | 0.001589 |
|  | BP | GO:0001933 | negative regulation of protein phosphorylation | 9 | 0.001657 |
|  | BP | GO:1901342 | regulation of vasculature development | 9 | 0.001657 |
|  | BP | GO:0031102 | neuron projection regeneration | 4 | 0.001657 |
|  | BP | GO:0003007 | heart morphogenesis | 7 | 0.00166 |
|  | BP | GO:0043200 | response to amino acid | 5 | 0.001782 |
|  | BP | GO:0050770 | regulation of axonogenesis | 6 | 0.002008 |
|  | BP | GO:0006936 | muscle contraction | 8 | 0.002161 |
|  | BP | GO:0030308 | negative regulation of cell growth | 6 | 0.002218 |
|  | BP | GO:0045927 | positive regulation of growth | 7 | 0.00231 |
|  | BP | GO:0032967 | positive regulation of collagen biosynthetic process | 3 | 0.002372 |
|  | BP | GO:0060343 | trabecula formation | 3 | 0.002372 |
|  | BP | GO:0090025 | regulation of monocyte chemotaxis | 3 | 0.002372 |
|  | BP | GO:0036293 | response to decreased oxygen levels | 8 | 0.002436 |
|  | BP | GO:0002548 | monocyte chemotaxis | 4 | 0.002486 |
|  | BP | GO:0007565 | female pregnancy | 6 | 0.002486 |
|  | BP | GO:0010714 | positive regulation of collagen metabolic process | 3 | 0.002581 |
|  | BP | GO:0001822 | kidney development | 7 | 0.002648 |
|  | BP | GO:0071230 | cellular response to amino acid stimulus | 4 | 0.002691 |
|  | BP | GO:1901888 | regulation of cell junction assembly | 6 | 0.002691 |
|  | BP | GO:0042326 | negative regulation of phosphorylation | 9 | 0.002744 |
|  | BP | GO:0007179 | transforming growth factor beta receptor signaling pathway | 6 | 0.002799 |
|  | BP | GO:0060485 | mesenchyme development | 7 | 0.002952 |
|  | BP | GO:0003206 | cardiac chamber morphogenesis | 5 | 0.002952 |
|  | BP | GO:0003231 | cardiac ventricle development | 5 | 0.002952 |
|  | BP | GO:0060351 | cartilage development involved in endochondral bone morphogenesis | 3 | 0.002995 |
|  | BP | GO:0072001 | renal system development | 7 | 0.002995 |
|  | BP | GO:0001101 | response to acid chemical | 5 | 0.002995 |
|  | BP | GO:0022604 | regulation of cell morphogenesis | 9 | 0.003247 |
|  | BP | GO:0030278 | regulation of ossification | 6 | 0.003429 |
|  | BP | GO:0098868 | bone growth | 3 | 0.003536 |
|  | BP | GO:0003281 | ventricular septum development | 4 | 0.003536 |
|  | BP | GO:0060415 | muscle tissue morphogenesis | 4 | 0.003536 |
|  | BP | GO:0071229 | cellular response to acid chemical | 4 | 0.003536 |
|  | BP | GO:0030500 | regulation of bone mineralization | 4 | 0.003668 |
|  | BP | GO:0060411 | cardiac septum morphogenesis | 4 | 0.003668 |
|  | BP | GO:0071295 | cellular response to vitamin | 3 | 0.003746 |
|  | BP | GO:0001889 | liver development | 5 | 0.003755 |
|  | BP | GO:0032355 | response to estradiol | 5 | 0.003755 |
|  | BP | GO:0061045 | negative regulation of wound healing | 4 | 0.003755 |
|  | BP | GO:0002687 | positive regulation of leukocyte migration | 5 | 0.003848 |
|  | BP | GO:0050921 | positive regulation of chemotaxis | 5 | 0.004052 |
|  | BP | GO:0061008 | hepaticobiliary system development | 5 | 0.004052 |
|  | BP | GO:0001960 | negative regulation of cytokine-mediated signaling pathway | 4 | 0.004229 |
|  | BP | GO:0045665 | negative regulation of neuron differentiation | 6 | 0.00427 |
|  | BP | GO:0048644 | muscle organ morphogenesis | 4 | 0.004584 |
|  | BP | GO:0048762 | mesenchymal cell differentiation | 6 | 0.004602 |
|  | BP | GO:0050920 | regulation of chemotaxis | 6 | 0.004602 |
|  | BP | GO:0048738 | cardiac muscle tissue development | 6 | 0.00479 |
|  | BP | GO:0070371 | ERK1 and ERK2 cascade | 7 | 0.004924 |
|  | BP | GO:0014910 | regulation of smooth muscle cell migration | 4 | 0.00506 |
|  | BP | GO:0071774 | response to fibroblast growth factor | 5 | 0.00506 |
|  | BP | GO:0000302 | response to reactive oxygen species | 6 | 0.00512 |
|  | BP | GO:0048846 | axon extension involved in axon guidance | 3 | 0.005132 |
|  | BP | GO:1902284 | neuron projection extension involved in neuron projection guidance | 3 | 0.005132 |
|  | BP | GO:0060420 | regulation of heart growth | 4 | 0.005132 |
|  | BP | GO:0060761 | negative regulation of response to cytokine stimulus | 4 | 0.005132 |
|  | BP | GO:0030509 | BMP signaling pathway | 5 | 0.00514 |
|  | BP | GO:0048754 | branching morphogenesis of an epithelial tube | 5 | 0.00514 |
|  | BP | GO:0048588 | developmental cell growth | 6 | 0.00514 |
|  | BP | GO:0034103 | regulation of tissue remodeling | 4 | 0.005945 |
|  | BP | GO:1902742 | apoptotic process involved in development | 3 | 0.006257 |
|  | BP | GO:0014909 | smooth muscle cell migration | 4 | 0.006381 |
|  | BP | GO:0031960 | response to corticosteroid | 5 | 0.006471 |
|  | BP | GO:0048545 | response to steroid hormone | 7 | 0.00654 |
|  | BP | GO:0070167 | regulation of biomineral tissue development | 4 | 0.006761 |
|  | BP | GO:0110149 | regulation of biomineralization | 4 | 0.006761 |
|  | BP | GO:0071772 | response to BMP | 5 | 0.006951 |
|  | BP | GO:0071773 | cellular response to BMP stimulus | 5 | 0.006951 |
|  | BP | GO:0045926 | negative regulation of growth | 6 | 0.006951 |
|  | BP | GO:2000027 | regulation of animal organ morphogenesis | 6 | 0.006951 |
|  | BP | GO:0006971 | hypotonic response | 2 | 0.006968 |
|  | BP | GO:0060346 | bone trabecula formation | 2 | 0.006968 |
|  | BP | GO:0070099 | regulation of chemokine-mediated signaling pathway | 2 | 0.006968 |
|  | BP | GO:0048638 | regulation of developmental growth | 7 | 0.006968 |
|  | BP | GO:0071674 | mononuclear cell migration | 4 | 0.006968 |
|  | BP | GO:0034614 | cellular response to reactive oxygen species | 5 | 0.007068 |
|  | BP | GO:0010470 | regulation of gastrulation | 3 | 0.007068 |
|  | BP | GO:0045124 | regulation of bone resorption | 3 | 0.007068 |
|  | BP | GO:0007584 | response to nutrient | 5 | 0.007173 |
|  | BP | GO:1990138 | neuron projection extension | 5 | 0.007326 |
|  | BP | GO:0003012 | muscle system process | 8 | 0.007359 |
|  | BP | GO:0001666 | response to hypoxia | 7 | 0.007369 |
|  | BP | GO:0031670 | cellular response to nutrient | 3 | 0.007369 |
|  | BP | GO:0050954 | sensory perception of mechanical stimulus | 5 | 0.007369 |
|  | BP | GO:0008585 | female gonad development | 4 | 0.007387 |
|  | BP | GO:0045807 | positive regulation of endocytosis | 4 | 0.007387 |
|  | BP | GO:0003205 | cardiac chamber development | 5 | 0.007451 |
|  | BP | GO:0060412 | ventricular septum morphogenesis | 3 | 0.007683 |
|  | BP | GO:0055024 | regulation of cardiac muscle tissue development | 4 | 0.007784 |
|  | BP | GO:0060840 | artery development | 4 | 0.007784 |
|  | BP | GO:0032060 | bleb assembly | 2 | 0.007784 |
|  | BP | GO:0090596 | sensory organ morphogenesis | 6 | 0.008368 |
|  | BP | GO:0022602 | ovulation cycle process | 3 | 0.008478 |
|  | BP | GO:0050919 | negative chemotaxis | 3 | 0.008478 |
|  | BP | GO:0046545 | development of primary female sexual characteristics | 4 | 0.008484 |
|  | BP | GO:0090288 | negative regulation of cellular response to growth factor stimulus | 5 | 0.008665 |
|  | BP | GO:0014812 | muscle cell migration | 4 | 0.008665 |
|  | BP | GO:0034446 | substrate adhesion-dependent cell spreading | 4 | 0.008665 |
|  | BP | GO:0030111 | regulation of Wnt signaling pathway | 7 | 0.008759 |
|  | BP | GO:0001959 | regulation of cytokine-mediated signaling pathway | 5 | 0.008773 |
|  | BP | GO:0030208 | dermatan sulfate biosynthetic process | 2 | 0.008825 |
|  | BP | GO:0071107 | response to parathyroid hormone | 2 | 0.008825 |
|  | BP | GO:0001936 | regulation of endothelial cell proliferation | 5 | 0.008825 |
|  | BP | GO:0090090 | negative regulation of canonical Wnt signaling pathway | 5 | 0.008825 |
|  | BP | GO:0002686 | negative regulation of leukocyte migration | 3 | 0.009058 |
|  | BP | GO:0061383 | trabecula morphogenesis | 3 | 0.009058 |
|  | BP | GO:0048639 | positive regulation of developmental growth | 5 | 0.009313 |
|  | BP | GO:0061138 | morphogenesis of a branching epithelium | 5 | 0.009313 |
|  | BP | GO:1904707 | positive regulation of vascular associated smooth muscle cell proliferation | 3 | 0.009479 |
|  | BP | GO:0001654 | eye development | 7 | 0.009555 |
|  | BP | GO:0071675 | regulation of mononuclear cell migration | 3 | 0.009842 |
|  | BP | GO:1900047 | negative regulation of hemostasis | 3 | 0.009842 |
|  | BP | GO:0030205 | dermatan sulfate metabolic process | 2 | 0.009842 |
|  | BP | GO:0061430 | bone trabecula morphogenesis | 2 | 0.009842 |
|  | BP | GO:1900025 | negative regulation of substrate adhesion-dependent cell spreading | 2 | 0.009842 |
|  | BP | GO:0150063 | visual system development | 7 | 0.009879 |
|  | BP | GO:0014706 | striated muscle tissue development | 7 | 0.009962 |
|  | BP | GO:0001938 | positive regulation of endothelial cell proliferation | 4 | 0.009962 |
|  | BP | GO:0043542 | endothelial cell migration | 6 | 0.009962 |
|  | BP | GO:0003179 | heart valve morphogenesis | 3 | 0.009962 |
|  | BP | GO:0010883 | regulation of lipid storage | 3 | 0.009962 |
|  | BP | GO:0031103 | axon regeneration | 3 | 0.009962 |
|  | BP | GO:0048260 | positive regulation of receptor-mediated endocytosis | 3 | 0.009962 |
|  | BP | GO:0060421 | positive regulation of heart growth | 3 | 0.009962 |
|  | BP | GO:0003279 | cardiac septum development | 4 | 0.010077 |
|  | BP | GO:0048880 | sensory system development | 7 | 0.010321 |
|  | BP | GO:0060419 | heart growth | 4 | 0.010321 |
|  | BP | GO:0050678 | regulation of epithelial cell proliferation | 7 | 0.010406 |
|  | BP | GO:0060759 | regulation of response to cytokine stimulus | 5 | 0.010483 |
|  | BP | GO:0046620 | regulation of organ growth | 4 | 0.010526 |
|  | BP | GO:0030207 | chondroitin sulfate catabolic process | 2 | 0.010668 |
|  | BP | GO:0050966 | detection of mechanical stimulus involved in sensory perception of pain | 2 | 0.010668 |
|  | BP | GO:0051146 | striated muscle cell differentiation | 6 | 0.011156 |
|  | BP | GO:0060828 | regulation of canonical Wnt signaling pathway | 6 | 0.011156 |
|  | BP | GO:1901654 | response to ketone | 5 | 0.011183 |
|  | BP | GO:0046660 | female sex differentiation | 4 | 0.011286 |
|  | BP | GO:0001763 | morphogenesis of a branching structure | 5 | 0.011341 |
|  | BP | GO:0018158 | protein oxidation | 2 | 0.011756 |
|  | BP | GO:0038065 | collagen-activated signaling pathway | 2 | 0.011756 |
|  | BP | GO:0045779 | negative regulation of bone resorption | 2 | 0.011756 |
|  | BP | GO:0050651 | dermatan sulfate proteoglycan biosynthetic process | 2 | 0.011756 |
|  | BP | GO:0070571 | negative regulation of neuron projection regeneration | 2 | 0.011756 |
|  | BP | GO:1900119 | positive regulation of execution phase of apoptosis | 2 | 0.011756 |
|  | BP | GO:0060541 | respiratory system development | 5 | 0.011776 |
|  | BP | GO:0010632 | regulation of epithelial cell migration | 6 | 0.01183 |
|  | BP | GO:0010811 | positive regulation of cell-substrate adhesion | 4 | 0.011893 |
|  | BP | GO:0048675 | axon extension | 4 | 0.012207 |
|  | BP | GO:0002688 | regulation of leukocyte chemotaxis | 4 | 0.012478 |
|  | BP | GO:0010633 | negative regulation of epithelial cell migration | 4 | 0.012478 |
|  | BP | GO:0070372 | regulation of ERK1 and ERK2 cascade | 6 | 0.01261 |
|  | BP | GO:0001952 | regulation of cell-matrix adhesion | 4 | 0.012754 |
|  | BP | GO:0060043 | regulation of cardiac muscle cell proliferation | 3 | 0.012807 |
|  | BP | GO:0050655 | dermatan sulfate proteoglycan metabolic process | 2 | 0.012807 |
|  | BP | GO:0061450 | trophoblast cell migration | 2 | 0.012807 |
|  | BP | GO:1901163 | regulation of trophoblast cell migration | 2 | 0.012807 |
|  | BP | GO:0050679 | positive regulation of epithelial cell proliferation | 5 | 0.013042 |
|  | BP | GO:0035904 | aorta development | 3 | 0.013237 |
|  | BP | GO:0072089 | stem cell proliferation | 4 | 0.013549 |
|  | BP | GO:0003170 | heart valve development | 3 | 0.013729 |
|  | BP | GO:0006027 | glycosaminoglycan catabolic process | 3 | 0.013729 |
|  | BP | GO:0043588 | skin development | 7 | 0.01387 |
|  | BP | GO:0006816 | calcium ion transport | 7 | 0.014004 |
|  | BP | GO:0010715 | regulation of extracellular matrix disassembly | 2 | 0.014061 |
|  | BP | GO:0010518 | positive regulation of phospholipase activity | 3 | 0.014077 |
|  | BP | GO:0055025 | positive regulation of cardiac muscle tissue development | 3 | 0.014077 |
|  | BP | GO:0070527 | platelet aggregation | 3 | 0.014077 |
|  | BP | GO:0045667 | regulation of osteoblast differentiation | 4 | 0.014271 |
|  | BP | GO:0007568 | aging | 6 | 0.014476 |
|  | BP | GO:0046622 | positive regulation of organ growth | 3 | 0.014476 |
|  | BP | GO:0051893 | regulation of focal adhesion assembly | 3 | 0.014476 |
|  | BP | GO:0090109 | regulation of cell-substrate junction assembly | 3 | 0.014476 |
|  | BP | GO:0030178 | negative regulation of Wnt signaling pathway | 5 | 0.014476 |
|  | BP | GO:0045453 | bone resorption | 3 | 0.015041 |
|  | BP | GO:1905049 | negative regulation of metallopeptidase activity | 2 | 0.015178 |
|  | BP | GO:0097529 | myeloid leukocyte migration | 5 | 0.015193 |
|  | BP | GO:0051235 | maintenance of location | 6 | 0.015279 |
|  | BP | GO:0008406 | gonad development | 5 | 0.015385 |
|  | BP | GO:0060389 | pathway-restricted SMAD protein phosphorylation | 3 | 0.015454 |
|  | BP | GO:0071496 | cellular response to external stimulus | 6 | 0.015591 |
|  | BP | GO:0045669 | positive regulation of osteoblast differentiation | 3 | 0.015978 |
|  | BP | GO:0150116 | regulation of cell-substrate junction organization | 3 | 0.015978 |
|  | BP | GO:0048732 | gland development | 7 | 0.016247 |
|  | BP | GO:2000647 | negative regulation of stem cell proliferation | 2 | 0.016409 |
|  | BP | GO:0006026 | aminoglycan catabolic process | 3 | 0.01642 |
|  | BP | GO:0050918 | positive chemotaxis | 3 | 0.01642 |
|  | BP | GO:0060562 | epithelial tube morphogenesis | 6 | 0.01642 |
|  | BP | GO:0045137 | development of primary sexual characteristics | 5 | 0.016676 |
|  | BP | GO:0032496 | response to lipopolysaccharide | 6 | 0.017053 |
|  | BP | GO:0009581 | detection of external stimulus | 4 | 0.017268 |
|  | BP | GO:0042698 | ovulation cycle | 3 | 0.017426 |
|  | BP | GO:0060038 | cardiac muscle cell proliferation | 3 | 0.017426 |
|  | BP | GO:0002544 | chronic inflammatory response | 2 | 0.017426 |
|  | BP | GO:0002689 | negative regulation of leukocyte chemotaxis | 2 | 0.017426 |
|  | BP | GO:0032495 | response to muramyl dipeptide | 2 | 0.017426 |
|  | BP | GO:0048670 | regulation of collateral sprouting | 2 | 0.017426 |
|  | BP | GO:0060231 | mesenchymal to epithelial transition | 2 | 0.017426 |
|  | BP | GO:0060070 | canonical Wnt signaling pathway | 6 | 0.017818 |
|  | BP | GO:0050771 | negative regulation of axonogenesis | 3 | 0.017861 |
|  | BP | GO:0009582 | detection of abiotic stimulus | 4 | 0.018032 |
|  | BP | GO:0006979 | response to oxidative stress | 7 | 0.01841 |
|  | BP | GO:0014823 | response to activity | 3 | 0.018416 |
|  | BP | GO:0042542 | response to hydrogen peroxide | 4 | 0.018498 |
|  | BP | GO:0044344 | cellular response to fibroblast growth factor stimulus | 4 | 0.018498 |
|  | BP | GO:0001502 | cartilage condensation | 2 | 0.018498 |
|  | BP | GO:0030502 | negative regulation of bone mineralization | 2 | 0.018498 |
|  | BP | GO:0061213 | positive regulation of mesonephros development | 2 | 0.018498 |
|  | BP | GO:0072574 | hepatocyte proliferation | 2 | 0.018498 |
|  | BP | GO:0072575 | epithelial cell proliferation involved in liver morphogenesis | 2 | 0.018498 |
|  | BP | GO:0010517 | regulation of phospholipase activity | 3 | 0.019429 |
|  | BP | GO:0010888 | negative regulation of lipid storage | 2 | 0.020106 |
|  | BP | GO:0072576 | liver morphogenesis | 2 | 0.020106 |
|  | BP | GO:0070838 | divalent metal ion transport | 7 | 0.020648 |
|  | BP | GO:0031100 | animal organ regeneration | 3 | 0.020648 |
|  | BP | GO:0048844 | artery morphogenesis | 3 | 0.020648 |
|  | BP | GO:0016202 | regulation of striated muscle tissue development | 4 | 0.021197 |
|  | BP | GO:0060193 | positive regulation of lipase activity | 3 | 0.021197 |
|  | BP | GO:0072091 | regulation of stem cell proliferation | 3 | 0.021197 |
|  | BP | GO:0002237 | response to molecule of bacterial origin | 6 | 0.021197 |
|  | BP | GO:0051043 | regulation of membrane protein ectodomain proteolysis | 2 | 0.02131 |
|  | BP | GO:1903077 | negative regulation of protein localization to plasma membrane | 2 | 0.02131 |
|  | BP | GO:2000050 | regulation of non-canonical Wnt signaling pathway | 2 | 0.02131 |
|  | BP | GO:0150076 | neuroinflammatory response | 3 | 0.021667 |
|  | BP | GO:0072511 | divalent inorganic cation transport | 7 | 0.021667 |
|  | BP | GO:0062197 | cellular response to chemical stress | 6 | 0.02198 |
|  | BP | GO:0001890 | placenta development | 4 | 0.022021 |
|  | BP | GO:0048592 | eye morphogenesis | 4 | 0.022021 |
|  | BP | GO:1901861 | regulation of muscle tissue development | 4 | 0.022021 |
|  | BP | GO:0060395 | SMAD protein signal transduction | 3 | 0.022115 |
|  | BP | GO:0007409 | axonogenesis | 7 | 0.022198 |
|  | BP | GO:0048634 | regulation of muscle organ development | 4 | 0.022198 |
|  | BP | GO:0003181 | atrioventricular valve morphogenesis | 2 | 0.022198 |
|  | BP | GO:0014850 | response to muscle activity | 2 | 0.022198 |
|  | BP | GO:0032331 | negative regulation of chondrocyte differentiation | 2 | 0.022198 |
|  | BP | GO:0033622 | integrin activation | 2 | 0.022198 |
|  | BP | GO:0048745 | smooth muscle tissue development | 2 | 0.022198 |
|  | BP | GO:0098743 | cell aggregation | 2 | 0.022198 |
|  | BP | GO:0090092 | regulation of transmembrane receptor protein serine/threonine kinase signaling pathway | 5 | 0.022225 |
|  | BP | GO:0071260 | cellular response to mechanical stimulus | 3 | 0.022272 |
|  | BP | GO:0048678 | response to axon injury | 3 | 0.022939 |
|  | BP | GO:0055021 | regulation of cardiac muscle tissue growth | 3 | 0.022939 |
|  | BP | GO:0051098 | regulation of binding | 6 | 0.022971 |
|  | BP | GO:0019915 | lipid storage | 3 | 0.023206 |
|  | BP | GO:0002092 | positive regulation of receptor internalization | 2 | 0.023206 |
|  | BP | GO:0045992 | negative regulation of embryonic development | 2 | 0.023206 |
|  | BP | GO:0046697 | decidualization | 2 | 0.023206 |
|  | BP | GO:0050927 | positive regulation of positive chemotaxis | 2 | 0.023206 |
|  | BP | GO:0061217 | regulation of mesonephros development | 2 | 0.023206 |
|  | BP | GO:0062149 | detection of stimulus involved in sensory perception of pain | 2 | 0.023206 |
|  | BP | GO:1904376 | negative regulation of protein localization to cell periphery | 2 | 0.023206 |
|  | BP | GO:0045862 | positive regulation of proteolysis | 6 | 0.023301 |
|  | BP | GO:0001894 | tissue homeostasis | 5 | 0.023957 |
|  | BP | GO:0014855 | striated muscle cell proliferation | 3 | 0.024459 |
|  | BP | GO:0048708 | astrocyte differentiation | 3 | 0.024459 |
|  | BP | GO:1900046 | regulation of hemostasis | 3 | 0.024459 |
|  | BP | GO:0050730 | regulation of peptidyl-tyrosine phosphorylation | 5 | 0.024467 |
|  | BP | GO:0003171 | atrioventricular valve development | 2 | 0.024469 |
|  | BP | GO:0030206 | chondroitin sulfate biosynthetic process | 2 | 0.024469 |
|  | BP | GO:0050926 | regulation of positive chemotaxis | 2 | 0.024469 |
|  | BP | GO:0034250 | positive regulation of cellular amide metabolic process | 4 | 0.025581 |
|  | BP | GO:0045844 | positive regulation of striated muscle tissue development | 3 | 0.025581 |
|  | BP | GO:0048636 | positive regulation of muscle organ development | 3 | 0.025581 |
|  | BP | GO:0110110 | positive regulation of animal organ morphogenesis | 3 | 0.025581 |
|  | BP | GO:0003416 | endochondral bone growth | 2 | 0.025803 |
|  | BP | GO:0018126 | protein hydroxylation | 2 | 0.025803 |
|  | BP | GO:0048668 | collateral sprouting | 2 | 0.025803 |
|  | BP | GO:0072337 | modified amino acid transport | 2 | 0.025803 |
|  | BP | GO:0034109 | homotypic cell-cell adhesion | 3 | 0.025946 |
|  | BP | GO:0050772 | positive regulation of axonogenesis | 3 | 0.025946 |
|  | BP | GO:1901863 | positive regulation of muscle tissue development | 3 | 0.025946 |
|  | BP | GO:1904705 | regulation of vascular associated smooth muscle cell proliferation | 3 | 0.026652 |
|  | BP | GO:1990874 | vascular associated smooth muscle cell proliferation | 3 | 0.026652 |
|  | BP | GO:0030307 | positive regulation of cell growth | 4 | 0.026813 |
|  | BP | GO:1905952 | regulation of lipid localization | 4 | 0.026813 |
|  | BP | GO:0003180 | aortic valve morphogenesis | 2 | 0.027005 |
|  | BP | GO:0006929 | substrate-dependent cell migration | 2 | 0.027005 |
|  | BP | GO:0043901 | negative regulation of multi-organism process | 2 | 0.027005 |
|  | BP | GO:0070098 | chemokine-mediated signaling pathway | 3 | 0.027089 |
|  | BP | GO:0042692 | muscle cell differentiation | 6 | 0.027815 |
|  | BP | GO:0006029 | proteoglycan metabolic process | 3 | 0.02849 |
|  | BP | GO:0070542 | response to fatty acid | 3 | 0.02849 |
|  | BP | GO:0001958 | endochondral ossification | 2 | 0.02849 |
|  | BP | GO:0036075 | replacement ossification | 2 | 0.02849 |
|  | BP | GO:0030324 | lung development | 4 | 0.029416 |
|  | BP | GO:0007548 | sex differentiation | 5 | 0.029416 |
|  | BP | GO:0042176 | regulation of protein catabolic process | 6 | 0.029722 |
|  | BP | GO:0030511 | positive regulation of transforming growth factor beta receptor signaling pathway | 2 | 0.029843 |
|  | BP | GO:0070168 | negative regulation of biomineral tissue development | 2 | 0.029843 |
|  | BP | GO:0110150 | negative regulation of biomineralization | 2 | 0.029843 |
|  | BP | GO:0120033 | negative regulation of plasma membrane bounded cell projection assembly | 2 | 0.029843 |
|  | BP | GO:1903846 | positive regulation of cellular response to transforming growth factor beta stimulus | 2 | 0.029843 |
|  | BP | GO:0030510 | regulation of BMP signaling pathway | 3 | 0.030391 |
|  | BP | GO:0061097 | regulation of protein tyrosine kinase activity | 3 | 0.030391 |
|  | BP | GO:0030323 | respiratory tube development | 4 | 0.030901 |
|  | BP | GO:1905330 | regulation of morphogenesis of an epithelium | 4 | 0.030901 |
|  | BP | GO:0045778 | positive regulation of ossification | 3 | 0.030901 |
|  | BP | GO:0060993 | kidney morphogenesis | 3 | 0.030901 |
|  | BP | GO:1904035 | regulation of epithelial cell apoptotic process | 3 | 0.030901 |
|  | BP | GO:0002828 | regulation of type 2 immune response | 2 | 0.030901 |
|  | BP | GO:0048710 | regulation of astrocyte differentiation | 2 | 0.030901 |
|  | BP | GO:0050650 | chondroitin sulfate proteoglycan biosynthetic process | 2 | 0.030901 |
|  | BP | GO:0061037 | negative regulation of cartilage development | 2 | 0.030901 |
|  | BP | GO:1905476 | negative regulation of protein localization to membrane | 2 | 0.030901 |
|  | BP | GO:0030177 | positive regulation of Wnt signaling pathway | 4 | 0.031446 |
|  | BP | GO:0072593 | reactive oxygen species metabolic process | 5 | 0.031446 |
|  | BP | GO:0003176 | aortic valve development | 2 | 0.032406 |
|  | BP | GO:0034694 | response to prostaglandin | 2 | 0.032406 |
|  | BP | GO:0061036 | positive regulation of cartilage development | 2 | 0.032406 |
|  | BP | GO:0070570 | regulation of neuron projection regeneration | 2 | 0.032406 |
|  | BP | GO:0050764 | regulation of phagocytosis | 3 | 0.032596 |
|  | BP | GO:1990868 | response to chemokine | 3 | 0.032596 |
|  | BP | GO:1990869 | cellular response to chemokine | 3 | 0.032596 |
|  | BP | GO:0051147 | regulation of muscle cell differentiation | 4 | 0.032609 |
|  | BP | GO:0002691 | regulation of cellular extravasation | 2 | 0.033688 |
|  | BP | GO:0003338 | metanephros morphogenesis | 2 | 0.033688 |
|  | BP | GO:0044319 | wound healing, spreading of cells | 2 | 0.033688 |
|  | BP | GO:0090505 | epiboly involved in wound healing | 2 | 0.033688 |
|  | BP | GO:1905048 | regulation of metallopeptidase activity | 2 | 0.033688 |
|  | BP | GO:0018149 | peptide cross-linking | 2 | 0.03545 |
|  | BP | GO:0035633 | maintenance of blood-brain barrier | 2 | 0.03545 |
|  | BP | GO:0090504 | epiboly | 2 | 0.03545 |
|  | BP | GO:0060191 | regulation of lipase activity | 3 | 0.035565 |
|  | BP | GO:1903076 | regulation of protein localization to plasma membrane | 3 | 0.036447 |
|  | BP | GO:0035909 | aorta morphogenesis | 2 | 0.036787 |
|  | BP | GO:0048333 | mesodermal cell differentiation | 2 | 0.036787 |
|  | BP | GO:0070306 | lens fiber cell differentiation | 2 | 0.036787 |
|  | BP | GO:0002042 | cell migration involved in sprouting angiogenesis | 3 | 0.036787 |
|  | BP | GO:1901890 | positive regulation of cell junction assembly | 3 | 0.036787 |
|  | BP | GO:0050731 | positive regulation of peptidyl-tyrosine phosphorylation | 4 | 0.036787 |
|  | BP | GO:1901136 | carbohydrate derivative catabolic process | 4 | 0.036787 |
|  | BP | GO:1905475 | regulation of protein localization to membrane | 4 | 0.036787 |
|  | BP | GO:0048511 | rhythmic process | 5 | 0.037184 |
|  | BP | GO:0002040 | sprouting angiogenesis | 4 | 0.037184 |
|  | BP | GO:0071897 | DNA biosynthetic process | 4 | 0.037184 |
|  | BP | GO:0042063 | gliogenesis | 5 | 0.03796 |
|  | BP | GO:0001893 | maternal placenta development | 2 | 0.03796 |
|  | BP | GO:0033280 | response to vitamin D | 2 | 0.03796 |
|  | BP | GO:0034405 | response to fluid shear stress | 2 | 0.03796 |
|  | BP | GO:1905332 | positive regulation of morphogenesis of an epithelium | 2 | 0.03796 |
|  | BP | GO:0048259 | regulation of receptor-mediated endocytosis | 3 | 0.03796 |
|  | BP | GO:0034599 | cellular response to oxidative stress | 5 | 0.039024 |
|  | BP | GO:0060326 | cell chemotaxis | 5 | 0.039393 |
|  | BP | GO:0010596 | negative regulation of endothelial cell migration | 3 | 0.039393 |
|  | BP | GO:0055017 | cardiac muscle tissue growth | 3 | 0.039393 |
|  | BP | GO:0010952 | positive regulation of peptidase activity | 4 | 0.039393 |
|  | BP | GO:1904994 | regulation of leukocyte adhesion to vascular endothelial cell | 2 | 0.039393 |
|  | BP | GO:1905314 | semi-lunar valve development | 2 | 0.039393 |
|  | BP | GO:1903532 | positive regulation of secretion by cell | 5 | 0.039947 |
|  | BP | GO:0032526 | response to retinoic acid | 3 | 0.040172 |
|  | BP | GO:0006939 | smooth muscle contraction | 3 | 0.040849 |
|  | BP | GO:0033138 | positive regulation of peptidyl-serine phosphorylation | 3 | 0.040849 |
|  | BP | GO:2000278 | regulation of DNA biosynthetic process | 3 | 0.040849 |
|  | BP | GO:0014912 | negative regulation of smooth muscle cell migration | 2 | 0.040849 |
|  | BP | GO:0060045 | positive regulation of cardiac muscle cell proliferation | 2 | 0.040849 |
|  | BP | GO:0072009 | nephron epithelium development | 3 | 0.041583 |
|  | BP | GO:0090100 | positive regulation of transmembrane receptor protein serine/threonine kinase signaling pathway | 3 | 0.041583 |
|  | BP | GO:0051149 | positive regulation of muscle cell differentiation | 3 | 0.042308 |
|  | BP | GO:0061387 | regulation of extent of cell growth | 3 | 0.042308 |
|  | BP | GO:0001662 | behavioral fear response | 2 | 0.042308 |
|  | BP | GO:0030204 | chondroitin sulfate metabolic process | 2 | 0.042308 |
|  | BP | GO:0042092 | type 2 immune response | 2 | 0.042308 |
|  | BP | GO:0045766 | positive regulation of angiogenesis | 4 | 0.0428 |
|  | BP | GO:0043434 | response to peptide hormone | 6 | 0.043149 |
|  | BP | GO:1903510 | mucopolysaccharide metabolic process | 3 | 0.04377 |
|  | BP | GO:0030100 | regulation of endocytosis | 4 | 0.04377 |
|  | BP | GO:0002209 | behavioral defense response | 2 | 0.04377 |
|  | BP | GO:0090184 | positive regulation of kidney development | 2 | 0.04377 |
|  | BP | GO:1902895 | positive regulation of pri-miRNA transcription by RNA polymerase II | 2 | 0.04377 |
|  | BP | GO:0043393 | regulation of protein binding | 4 | 0.044261 |
|  | BP | GO:0042596 | fear response | 2 | 0.045605 |
|  | BP | GO:1900117 | regulation of execution phase of apoptosis | 2 | 0.045605 |
|  | BP | GO:0014902 | myotube differentiation | 3 | 0.047357 |
|  | BP | GO:1904019 | epithelial cell apoptotic process | 3 | 0.047357 |
|  | BP | GO:0006509 | membrane protein ectodomain proteolysis | 2 | 0.047364 |
|  | BP | GO:1904037 | positive regulation of epithelial cell apoptotic process | 2 | 0.047364 |
|  | BP | GO:0021987 | cerebral cortex development | 3 | 0.048149 |
|  | BP | GO:0002683 | negative regulation of immune system process | 6 | 0.048939 |
|  | BP | GO:0051153 | regulation of striated muscle cell differentiation | 3 | 0.048939 |
|  | BP | GO:0001953 | negative regulation of cell-matrix adhesion | 2 | 0.048939 |
|  | BP | GO:0071364 | cellular response to epidermal growth factor stimulus | 2 | 0.048939 |
|  | BP | GO:0150077 | regulation of neuroinflammatory response | 2 | 0.048939 |
|  | CC | GO:0062023 | collagen-containing extracellular matrix | 35 | 1.23E-34 |
|  | CC | GO:0005788 | endoplasmic reticulum lumen | 20 | 7.69E-17 |
|  | CC | GO:0098644 | complex of collagen trimers | 7 | 9.73E-11 |
|  | CC | GO:0005581 | collagen trimer | 10 | 1.04E-10 |
|  | CC | GO:0005583 | fibrillar collagen trimer | 6 | 1.04E-10 |
|  | CC | GO:0098643 | banded collagen fibril | 6 | 1.04E-10 |
|  | CC | GO:0005604 | basement membrane | 9 | 9.75E-09 |
|  | CC | GO:0005925 | focal adhesion | 11 | 2.26E-05 |
|  | CC | GO:0030055 | cell-substrate junction | 11 | 2.42E-05 |
|  | CC | GO:0005614 | interstitial matrix | 3 | 0.000312 |
|  | CC | GO:0031258 | lamellipodium membrane | 3 | 0.001486 |
|  | CC | GO:0001725 | stress fiber | 4 | 0.002193 |
|  | CC | GO:0097517 | contractile actin filament bundle | 4 | 0.002193 |
|  | CC | GO:0030027 | lamellipodium | 6 | 0.002193 |
|  | CC | GO:0032432 | actin filament bundle | 4 | 0.003057 |
|  | CC | GO:0042641 | actomyosin | 4 | 0.003324 |
|  | CC | GO:0031252 | cell leading edge | 8 | 0.003397 |
|  | CC | GO:0005916 | fascia adherens | 2 | 0.006806 |
|  | CC | GO:0031089 | platelet dense granule lumen | 2 | 0.012899 |
|  | CC | GO:0009897 | external side of plasma membrane | 7 | 0.014013 |
|  | CC | GO:0042827 | platelet dense granule | 2 | 0.026431 |
|  | CC | GO:0031594 | neuromuscular junction | 3 | 0.02889 |
|  | CC | GO:0031256 | leading edge membrane | 4 | 0.043079 |
|  | CC | GO:0031091 | platelet alpha granule | 3 | 0.043779 |
|  | CC | GO:0008305 | integrin complex | 2 | 0.047863 |
|  | MF | GO:0005201 | extracellular matrix structural constituent | 23 | 3.47E-26 |
|  | MF | GO:0005178 | integrin binding | 16 | 1.71E-16 |
|  | MF | GO:0005539 | glycosaminoglycan binding | 17 | 1.12E-14 |
|  | MF | GO:0008201 | heparin binding | 15 | 3.33E-14 |
|  | MF | GO:1901681 | sulfur compound binding | 16 | 9.89E-13 |
|  | MF | GO:0050840 | extracellular matrix binding | 9 | 9.09E-11 |
|  | MF | GO:0030020 | extracellular matrix structural constituent conferring tensile strength | 8 | 2.19E-10 |
|  | MF | GO:0005518 | collagen binding | 9 | 4.71E-10 |
|  | MF | GO:0002020 | protease binding | 10 | 9.18E-09 |
|  | MF | GO:0019838 | growth factor binding | 9 | 1.53E-07 |
|  | MF | GO:0061134 | peptidase regulator activity | 10 | 1.06E-06 |
|  | MF | GO:0001968 | fibronectin binding | 5 | 1.56E-06 |
|  | MF | GO:0048407 | platelet-derived growth factor binding | 4 | 1.56E-06 |
|  | MF | GO:0043394 | proteoglycan binding | 5 | 6.40E-06 |
|  | MF | GO:0046332 | SMAD binding | 6 | 1.57E-05 |
|  | MF | GO:0004866 | endopeptidase inhibitor activity | 8 | 1.62E-05 |
|  | MF | GO:0030414 | peptidase inhibitor activity | 8 | 1.94E-05 |
|  | MF | GO:0061135 | endopeptidase regulator activity | 8 | 2.07E-05 |
|  | MF | GO:0004867 | serine-type endopeptidase inhibitor activity | 6 | 4.40E-05 |
|  | MF | GO:0005520 | insulin-like growth factor binding | 4 | 6.55E-05 |
|  | MF | GO:0043236 | laminin binding | 4 | 6.55E-05 |
|  | MF | GO:0019955 | cytokine binding | 6 | 0.000238 |
|  | MF | GO:0008191 | metalloendopeptidase inhibitor activity | 3 | 0.000346 |
|  | MF | GO:0004857 | enzyme inhibitor activity | 9 | 0.000353 |
|  | MF | GO:0048018 | receptor ligand activity | 10 | 0.000393 |
|  | MF | GO:0030546 | signaling receptor activator activity | 10 | 0.000412 |
|  | MF | GO:0005125 | cytokine activity | 7 | 0.000518 |
|  | MF | GO:0008083 | growth factor activity | 6 | 0.000518 |
|  | MF | GO:0005126 | cytokine receptor binding | 7 | 0.001198 |
|  | MF | GO:0004222 | metalloendopeptidase activity | 4 | 0.007949 |
|  | MF | GO:0031994 | insulin-like growth factor I binding | 2 | 0.008108 |
|  | MF | GO:0036122 | BMP binding | 2 | 0.008108 |
|  | MF | GO:0008022 | protein C-terminus binding | 5 | 0.008108 |
|  | MF | GO:0001846 | opsonin binding | 2 | 0.010244 |
|  | MF | GO:0016641 | oxidoreductase activity, acting on the CH-NH2 group of donors, oxygen as acceptor | 2 | 0.011025 |
|  | MF | GO:0043395 | heparan sulfate proteoglycan binding | 2 | 0.011025 |
|  | MF | GO:0016638 | oxidoreductase activity, acting on the CH-NH2 group of donors | 2 | 0.015728 |
|  | MF | GO:0031418 | L-ascorbic acid binding | 2 | 0.015728 |
|  | MF | GO:0072349 | modified amino acid transmembrane transporter activity | 2 | 0.015728 |
|  | MF | GO:0070851 | growth factor receptor binding | 4 | 0.015728 |
|  | MF | GO:0030021 | extracellular matrix structural constituent conferring compression resistance | 2 | 0.018319 |
|  | MF | GO:0017134 | fibroblast growth factor binding | 2 | 0.019076 |
|  | MF | GO:0050431 | transforming growth factor beta binding | 2 | 0.019076 |
|  | MF | GO:0030165 | PDZ domain binding | 3 | 0.025657 |
|  | MF | GO:0030246 | carbohydrate binding | 5 | 0.025913 |
|  | MF | GO:0004252 | serine-type endopeptidase activity | 4 | 0.025913 |
|  | MF | GO:0017147 | Wnt-protein binding | 2 | 0.029414 |
|  | MF | GO:0019956 | chemokine binding | 2 | 0.034483 |
|  | MF | GO:0008236 | serine-type peptidase activity | 4 | 0.034483 |
|  | MF | GO:0008237 | metallopeptidase activity | 4 | 0.035043 |
|  | MF | GO:0017171 | serine hydrolase activity | 4 | 0.035609 |
|  | MF | GO:0004175 | endopeptidase activity | 6 | 0.045243 |
|  | MF | GO:0016504 | peptidase activator activity | 2 | 0.04546 |
|  | **BP: Biological Process; CC: Cellular Component; MF: Molecular Function** | | |  | |
|  |  |  |  |  |  |
