# Supplementary material for: A Prognostic Risk Score Based on Hypoxia-, Immunity-, and Epithelialto-Mesenchymal Transition-Related Genes for the Prognosis and Immunotherapy Response of Lung Adenocarcinoma
Source: Front Cell Dev Biol. 2022 Jan 24;9:758777. doi: 10.3389/fcell.2021.758777 (PMC8819669; doi:10.3389/fcell.2021.758777)
Supplement: Supplementary file 12 [file Table10.DOCX]

| **Supplementary Table 10 \| GO enrichment analysis of DEGs between high risk score group and low risk score group** | | | | |
| --- | --- | --- | --- | --- |
| Category | ID | Description | Count | qvalue |
| BP | GO:0006959 | humoral immune response | 53 | 5.82E-15 |
| BP | GO:0003341 | cilium movement | 28 | 1.03E-09 |
| BP | GO:0002455 | humoral immune response mediated by circulating immunoglobulin | 26 | 2.33E-08 |
| BP | GO:0019730 | antimicrobial humoral response | 24 | 4.10E-08 |
| BP | GO:0006958 | complement activation, classical pathway | 24 | 6.14E-08 |
| BP | GO:0006956 | complement activation | 26 | 2.75E-07 |
| BP | GO:0002920 | regulation of humoral immune response | 22 | 5.54E-07 |
| BP | GO:0001578 | microtubule bundle formation | 19 | 5.79E-07 |
| BP | GO:0035082 | axoneme assembly | 16 | 5.79E-07 |
| BP | GO:0030449 | regulation of complement activation | 20 | 7.96E-07 |
| BP | GO:0002377 | immunoglobulin production | 27 | 1.24E-06 |
| BP | GO:0016064 | immunoglobulin mediated immune response | 28 | 1.49E-06 |
| BP | GO:0019724 | B cell mediated immunity | 28 | 1.84E-06 |
| BP | GO:0070286 | axonemal dynein complex assembly | 11 | 1.94E-06 |
| BP | GO:0070268 | cornification | 18 | 1.41E-05 |
| BP | GO:0002460 | adaptive immune response based on somatic recombination of immune receptors built from immunoglobulin superfamily domains | 35 | 1.47E-05 |
| BP | GO:0002440 | production of molecular mediator of immune response | 30 | 5.70E-05 |
| BP | GO:0002449 | lymphocyte mediated immunity | 33 | 9.49E-05 |
| BP | GO:0007018 | microtubule-based movement | 33 | 9.88E-05 |
| BP | GO:0031638 | zymogen activation | 12 | 9.88E-05 |
| BP | GO:0031639 | plasminogen activation | 8 | 0.000108 |
| BP | GO:0030198 | extracellular matrix organization | 34 | 0.000152 |
| BP | GO:0043062 | extracellular structure organization | 34 | 0.000154 |
| BP | GO:0031581 | hemidesmosome assembly | 6 | 0.000206 |
| BP | GO:0036158 | outer dynein arm assembly | 7 | 0.000304 |
| BP | GO:0007586 | digestion | 17 | 0.001032 |
| BP | GO:0001539 | cilium or flagellum-dependent cell motility | 15 | 0.002618 |
| BP | GO:0060285 | cilium-dependent cell motility | 15 | 0.002618 |
| BP | GO:0002429 | immune response-activating cell surface receptor signaling pathway | 35 | 0.003194 |
| BP | GO:0002757 | immune response-activating signal transduction | 35 | 0.003194 |
| BP | GO:0097529 | myeloid leukocyte migration | 21 | 0.003425 |
| BP | GO:0016485 | protein processing | 21 | 0.003478 |
| BP | GO:0019731 | antibacterial humoral response | 10 | 0.003478 |
| BP | GO:0002431 | Fc receptor mediated stimulatory signaling pathway | 16 | 0.004465 |
| BP | GO:0008544 | epidermis development | 34 | 0.005411 |
| BP | GO:0050853 | B cell receptor signaling pathway | 15 | 0.006763 |
| BP | GO:0002697 | regulation of immune effector process | 33 | 0.008742 |
| BP | GO:0002433 | immune response-regulating cell surface receptor signaling pathway involved in phagocytosis | 15 | 0.008742 |
| BP | GO:0038096 | Fc-gamma receptor signaling pathway involved in phagocytosis | 15 | 0.008742 |
| BP | GO:0036159 | inner dynein arm assembly | 5 | 0.01038 |
| BP | GO:0038094 | Fc-gamma receptor signaling pathway | 15 | 0.01038 |
| BP | GO:0060294 | cilium movement involved in cell motility | 13 | 0.01038 |
| BP | GO:0035987 | endodermal cell differentiation | 8 | 0.014014 |
| BP | GO:0043588 | skin development | 30 | 0.014014 |
| BP | GO:0006691 | leukotriene metabolic process | 7 | 0.014014 |
| BP | GO:0051604 | protein maturation | 23 | 0.017098 |
| BP | GO:1905039 | carboxylic acid transmembrane transport | 15 | 0.017098 |
| BP | GO:1903825 | organic acid transmembrane transport | 15 | 0.018016 |
| BP | GO:0038095 | Fc-epsilon receptor signaling pathway | 16 | 0.019169 |
| BP | GO:0070327 | thyroid hormone transport | 4 | 0.019795 |
| BP | GO:0031424 | keratinization | 19 | 0.022147 |
| BP | GO:0044706 | multi-multicellular organism process | 19 | 0.022978 |
| BP | GO:1903035 | negative regulation of response to wounding | 11 | 0.025236 |
| BP | GO:0030216 | keratinocyte differentiation | 23 | 0.027516 |
| BP | GO:0048875 | chemical homeostasis within a tissue | 4 | 0.027516 |
| BP | GO:0006898 | receptor-mediated endocytosis | 24 | 0.028004 |
| BP | GO:0030595 | leukocyte chemotaxis | 19 | 0.028004 |
| BP | GO:0042742 | defense response to bacterium | 25 | 0.028004 |
| BP | GO:0001704 | formation of primary germ layer | 13 | 0.028004 |
| BP | GO:0071621 | granulocyte chemotaxis | 13 | 0.028004 |
| BP | GO:0002576 | platelet degranulation | 13 | 0.032046 |
| BP | GO:0001706 | endoderm formation | 8 | 0.034634 |
| BP | GO:0010755 | regulation of plasminogen activation | 4 | 0.034634 |
| BP | GO:0098656 | anion transmembrane transport | 23 | 0.034634 |
| BP | GO:0006766 | vitamin metabolic process | 13 | 0.034634 |
| BP | GO:0031099 | regeneration | 17 | 0.034634 |
| BP | GO:0030277 | maintenance of gastrointestinal epithelium | 5 | 0.036315 |
| BP | GO:0097530 | granulocyte migration | 14 | 0.038534 |
| BP | GO:0006909 | phagocytosis | 26 | 0.041992 |
| BP | GO:0021988 | olfactory lobe development | 6 | 0.045956 |
| BP | GO:0006575 | cellular modified amino acid metabolic process | 16 | 0.048306 |
| BP | GO:0046456 | icosanoid biosynthetic process | 8 | 0.048306 |
| CC | GO:0019814 | immunoglobulin complex | 35 | 7.05E-16 |
| CC | GO:0062023 | collagen-containing extracellular matrix | 46 | 1.29E-09 |
| CC | GO:0005930 | axoneme | 23 | 2.91E-09 |
| CC | GO:0097014 | ciliary plasm | 23 | 3.09E-09 |
| CC | GO:0031514 | motile cilium | 26 | 3.76E-07 |
| CC | GO:0032838 | plasma membrane bounded cell projection cytoplasm | 25 | 4.41E-06 |
| CC | GO:0031225 | anchored component of membrane | 22 | 5.30E-06 |
| CC | GO:0099568 | cytoplasmic region | 26 | 3.31E-05 |
| CC | GO:0009897 | external side of plasma membrane | 35 | 4.67E-05 |
| CC | GO:0042599 | lamellar body | 6 | 0.000501 |
| CC | GO:0036157 | outer dynein arm | 5 | 0.00058 |
| CC | GO:0005858 | axonemal dynein complex | 6 | 0.001197 |
| CC | GO:0034774 | secretory granule lumen | 26 | 0.001428 |
| CC | GO:0016324 | apical plasma membrane | 28 | 0.001428 |
| CC | GO:0060205 | cytoplasmic vesicle lumen | 26 | 0.0015 |
| CC | GO:0030286 | dynein complex | 9 | 0.0015 |
| CC | GO:0031983 | vesicle lumen | 26 | 0.0015 |
| CC | GO:0005604 | basement membrane | 12 | 0.004193 |
| CC | GO:0045177 | apical part of cell | 30 | 0.00435 |
| CC | GO:0097729 | 9+2 motile cilium | 13 | 0.00435 |
| CC | GO:0031091 | platelet alpha granule | 11 | 0.004713 |
| CC | GO:0043256 | laminin complex | 4 | 0.008654 |
| CC | GO:0072562 | blood microparticle | 14 | 0.009024 |
| CC | GO:0005581 | collagen trimer | 10 | 0.011522 |
| CC | GO:0031904 | endosome lumen | 6 | 0.015515 |
| CC | GO:0005771 | multivesicular body | 8 | 0.016424 |
| CC | GO:0036126 | sperm flagellum | 11 | 0.026277 |
| CC | GO:0031526 | brush border membrane | 7 | 0.044747 |
| CC | GO:0001533 | cornified envelope | 6 | 0.048947 |
| CC | GO:0046658 | anchored component of plasma membrane | 7 | 0.048947 |
| CC | GO:0005921 | gap junction | 5 | 0.048947 |
| CC | GO:0042571 | immunoglobulin complex, circulating | 8 | 0.048947 |
| MF | GO:0005201 | extracellular matrix structural constituent | 23 | 1.10E-05 |
| MF | GO:0008201 | heparin binding | 22 | 2.48E-05 |
| MF | GO:0005539 | glycosaminoglycan binding | 26 | 2.48E-05 |
| MF | GO:1901681 | sulfur compound binding | 26 | 0.000199 |
| MF | GO:0003823 | antigen binding | 19 | 0.000642 |
| MF | GO:0004867 | serine-type endopeptidase inhibitor activity | 14 | 0.000642 |
| MF | GO:0050786 | RAGE receptor binding | 5 | 0.001626 |
| MF | GO:0048018 | receptor ligand activity | 34 | 0.00577 |
| MF | GO:0030546 | signaling receptor activator activity | 34 | 0.00577 |
| MF | GO:0030414 | peptidase inhibitor activity | 18 | 0.00577 |
| MF | GO:0004252 | serine-type endopeptidase activity | 16 | 0.013567 |
| MF | GO:0005179 | hormone activity | 13 | 0.014383 |
| MF | GO:0005504 | fatty acid binding | 7 | 0.014383 |
| MF | GO:0017171 | serine hydrolase activity | 17 | 0.014383 |
| MF | GO:0061134 | peptidase regulator activity | 19 | 0.01501 |
| MF | GO:0030020 | extracellular matrix structural constituent conferring tensile strength | 7 | 0.017197 |
| MF | GO:0004866 | endopeptidase inhibitor activity | 16 | 0.021397 |
| MF | GO:0008509 | anion transmembrane transporter activity | 25 | 0.021608 |
| MF | GO:0008236 | serine-type peptidase activity | 16 | 0.022877 |
| MF | GO:0031994 | insulin-like growth factor I binding | 4 | 0.022877 |
| MF | GO:0033293 | monocarboxylic acid binding | 9 | 0.022877 |
| MF | GO:0061135 | endopeptidase regulator activity | 16 | 0.02776 |
| MF | GO:0019865 | immunoglobulin binding | 5 | 0.03145 |
| MF | GO:0005178 | integrin binding | 13 | 0.036068 |
