# Supplementary material for: A Prognostic Risk Score Based on Hypoxia-, Immunity-, and Epithelialto-Mesenchymal Transition-Related Genes for the Prognosis and Immunotherapy Response of Lung Adenocarcinoma
Source: Front Cell Dev Biol. 2022 Jan 24;9:758777. doi: 10.3389/fcell.2021.758777 (PMC8819669; doi:10.3389/fcell.2021.758777)
Supplement: Supplementary file 13 [file Image1.pdf]

# Supplementary Figure 1 | The analysis of independent prognostic risk factors

A

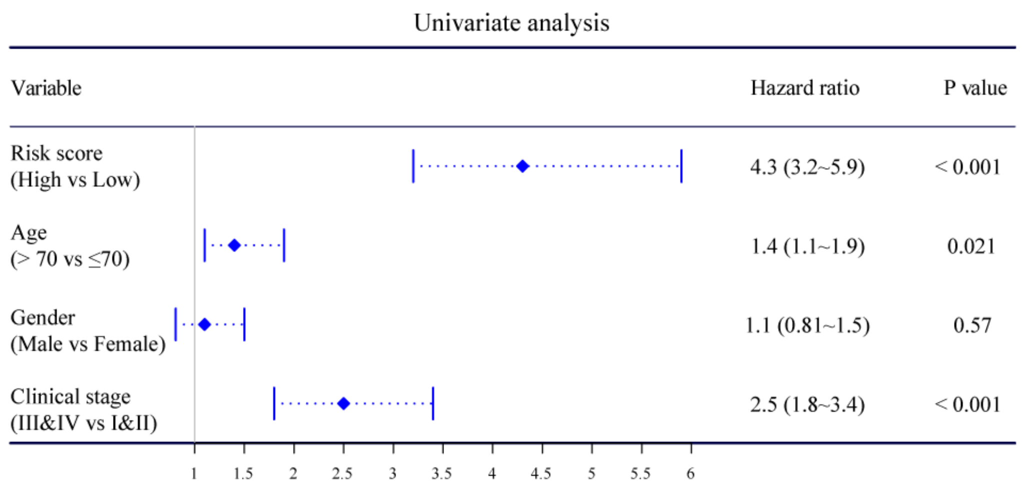


B

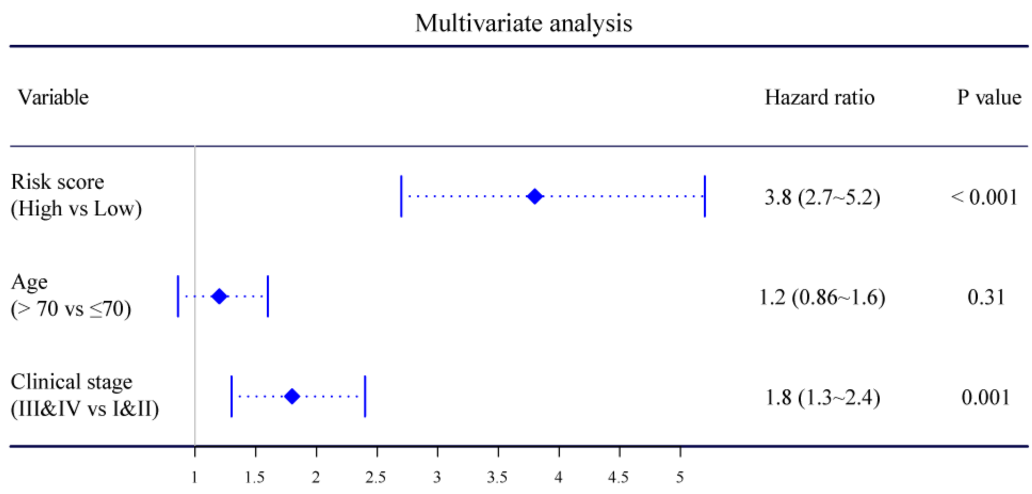


(A) Results of the univariate Cox regression analyses for OS in the TCGA cohort. (B) Results of the multivariate Cox regression analyses for OS in the TCGA cohort.
